# Olefin Dihydroxylation Using Nitroarenes as Photoresponsive Oxidants

**DOI:** 10.1002/anie.202214508

**Published:** 2023-01-17

**Authors:** Charlotte Hampton, Marco Simonetti, Daniele Leonori

**Affiliations:** ^1^ Department of Chemistry University of Manchester Oxford Road Manchester M13 9PL UK; ^2^ Institute of Organic Chemistry RWTH Aachen University Landoltweg 1 52056 Aachen Germany

**Keywords:** Dihydroxylation, Nitroarenes, Olefin Functionalization, Oxidation, Photochemistry

## Abstract

Vicinal diols are abundant among natural and synthetic molecules, and also represent valuable intermediates throughout organic synthesis. Olefin dihydroxylation is an effective strategy to access these derivatives owing to the broad range and availability of alkene feedstocks. OsO_4_ is among the most used reagents to achieve this transformation, yet its high toxicity and cost remain concerning. Herein, we present a mechanistically distinct strategy for olefin dihydroxylation using nitroarenes as photoresponsive oxidants. Upon purple LEDs irradiation, these species undergo a [3+2]‐photocycloaddition with a wide range of olefins to give stable 1,3,2‐dioxazolidine intermediates. These species can be accumulated in solution and then reduced in situ to the desired diols, utilising readily accessible and easy to handle solid reagents as H_2_ surrogates.

## Graphical abstract

Photoexcited nitroarenes ca be used, in conjunction with Pd‐mediated hydrogenation, as osmium surrogates for olefin dihydroxylation.

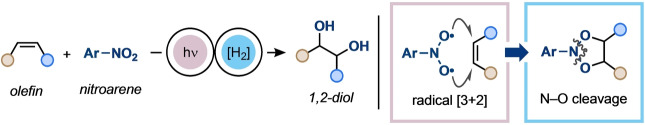

Vicinal diols are often encountered in the structure of many bioactive materials and are also routinely prepared as part of the manufacture of high‐value products by the pharmaceutical, agrochemical and fragrance industries (Scheme 1A).^[1]^

**Scheme 1:**
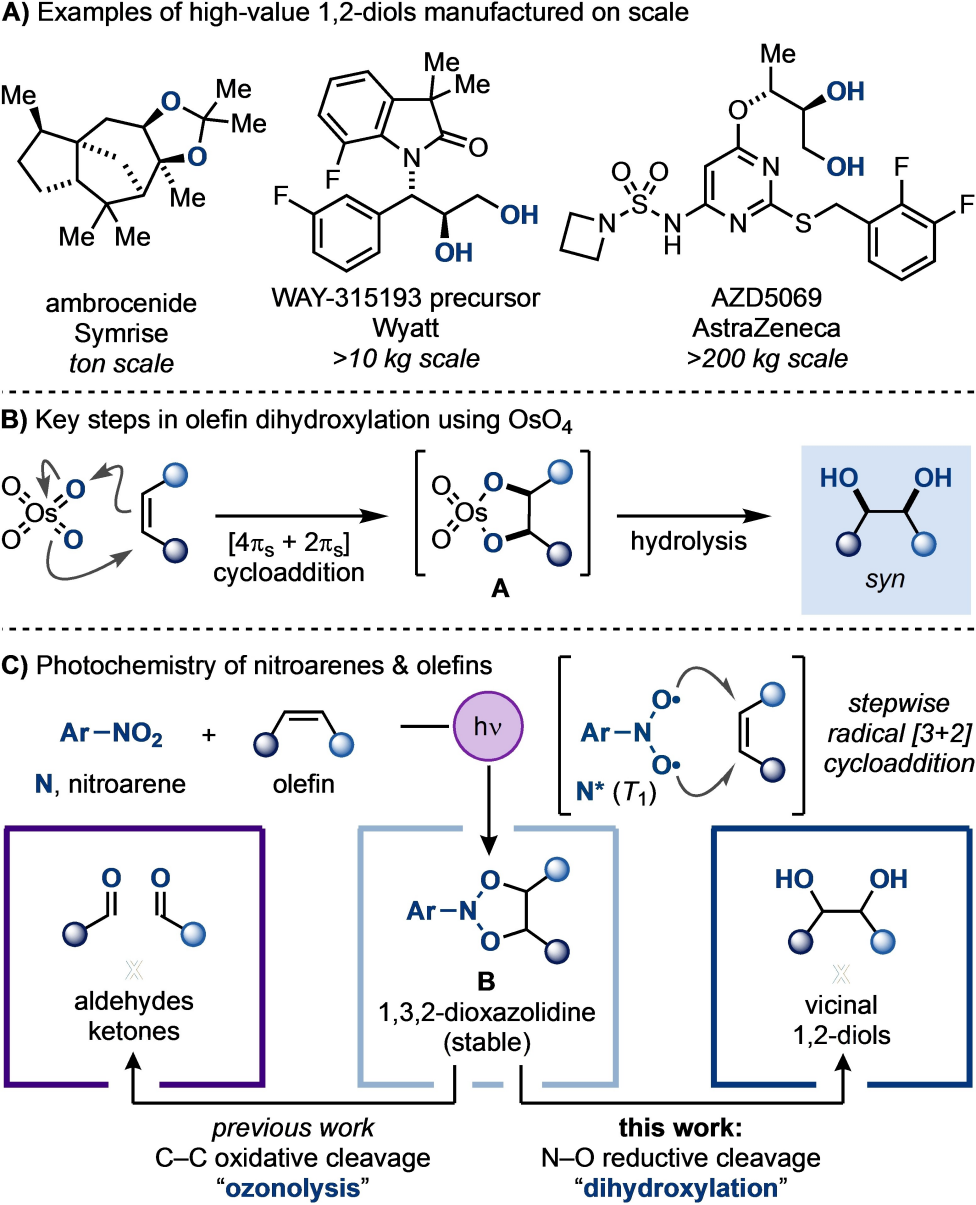
A) Vicinal diols are encountered in the structure of high‐value materials. B) Olefin dihydroxylation is generally achieved using OsO_4_. C) Photoexcited nitroarenes can be used to oxidise olefins and obtain either products of “ozonolysis” (previous work) or “dihydroxylation” (this work).

Olefin dihydroxylation with OsO_4_ is the most straightforward approach to access 1,2‐diols by converting alkene feedstocks into highly valuable oxygen‐enriched derivatives (Scheme 1B). This process features the stereospecific [4π_s_+2π_s_] cycloaddition of OsO_4_ across the C−Cπ bond delivering an intermediate osmate ester (**A**), from which hydrolysis gives the *syn*‐1,2‐diol (Scheme 1B). In order to be catalytic in [Os], these processes require the use of terminal oxidants, of which *N*‐methylmorpholine *N*‐oxide (Upjohn procedure) and K_3_Fe(CN)_6_ (Sharpless asymmetric dihydroxylation) are the most used.^[2]^ Despite the unquestionable power of OsO_4_ in delivering 1,2‐diols, its toxicity (aggravated by the fact that it sublimes at room temperature), as well as its high cost, continues to prompt the development of alternative strategies.^[3]^ While tremendous efforts have been made attempting to use other transition metal oxides (e.g. Ru, Fe, Mn, Pd, Mo, etc.) for this purpose,[^2d^, ^4^] these methods frequently display reduced scope and selectivity, and also can lead to undesired side products due to over‐oxidation. Methods based on stepwise olefin epoxidation followed by ring‐opening with *O*‐nucleophiles are often used to access *anti*‐1,2‐diols but they also pose challenges in terms of scope and functional group compatibility.^[5]^ More recently, mild and sustainable methods based on electrochemistry,^[6]^ electrophotochemistry^[7]^ and photocatalysis^[8]^ have been developed, but they are typically limited to the dihydroxylation of activated substrates such as styrenes. Additional strategies include those based on oxygen‐centred radical precursors[^5^, ^9^] and diborylations,^[10]^ as well as novel peroxide[^5^, ^11^] and hypervalent iodine[^5^, ^12^] systems. In some cases, unactivated olefins can be engaged but at the expense of functional group compatibility and reaction efficiency. Overall, a strategy able to convert olefins into vicinal diols using safe reagents under mild conditions, and which effectively engages a broad range of unactivated olefins remains a challenge in the field.

We recently became interested in the possibility of carrying out olefin dihydroxylation via a mechanistically distinct approach. Inspired by pioneering work from Büchi^[13]^ and De Mayo,^[14]^ our group^[15]^ and the one of Parasram^[16]^ have independently developed a general approach to carry out the ozonolysis‐style oxidative cleavage of olefins using visible light‐excited nitroarenes. This strategy is based on a stepwise radical [3+2]‐like cycloaddition between the triplet excited nitroarene *N and the olefin that delivers a relatively stable 1,3,2‐dioxazolidine species **B** (Scheme 1C). In our previous work we have demonstrated that these species can be effectively considered “*N*‐doped” ozonides and that they undergo efficient C−C σ‐bond cleavage leading to the corresponding aldehyde/ketone products. Herein, we report the development of a reductive protocol that, via the cleavage of their N−O bonds, provides an alternative approach for the general dihydroxylation of olefins.

The proposed mechanism for this strategy is depicted in Scheme 2A. Since nitroarenes have an absorption profile tailing in the visible range, purple LEDs irradiation can populate their singlet excited state (*S*_1_) and, following intersystem crossing (ISC), their long‐lived triplet state (*T*_1_). The *O*‐radical character of *T*_1_‐nitroarenes ensures favourable addition to olefins to produce an intermediate triplet biradical **C** that, upon ISC, cyclises to the 1,3,2‐dioxazolidine species **B**. To develop a successful and practical dihydroxylation strategy, accumulation of **B** in solution is essential, and conditions need to be identified to selectively cleave the two N−O bonds while suppressing the strong tendency of these species to react via C−C σ‐cleavage.^[15]^ It is important to note that De Mayo, in his pioneering works, demonstrated the reduction of such intermediates to the corresponding diols.^[14]^ However, this necessitated the use of stoichiometric PtO_2_/H_2_, as well as the prior isolation of **B** (generated by high‐energy irradiation at −78 °C using the olefin as the solvent), which hampered the adoption of his work as a method for olefin dihydroxylation.^[14b]^ Building on this seminal work,^[14b]^ one of the key findings of our previous contribution has been the realisation that upon placing highly electron withdrawing groups on the nitroarene, the corresponding cycloadduct **B** becomes a relatively stable and safe intermediate.^[15]^ We were therefore hopeful that this would have enabled us to identify conditions to generate **B**, accumulate it in situ and then subject it to a reductive protocol for diol synthesis.

**Scheme 2:**
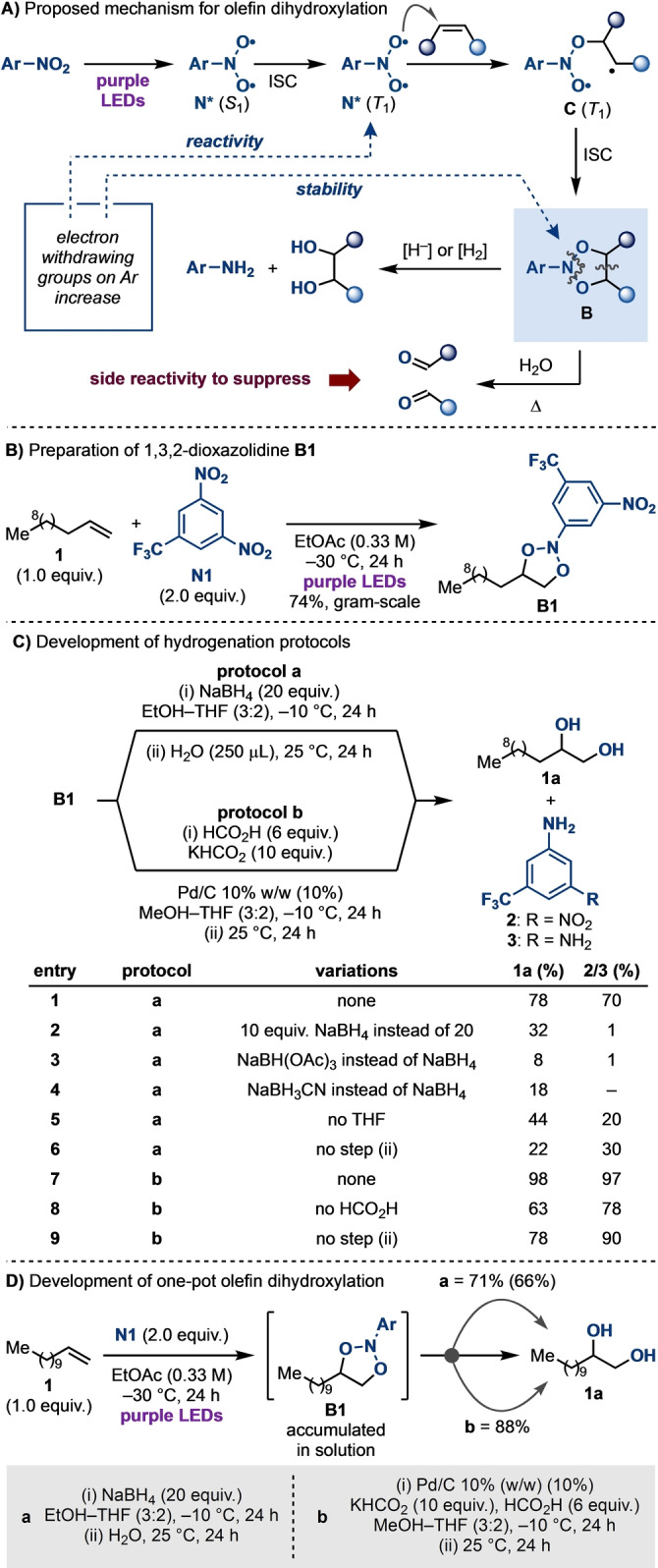
A) The proposed mechanism for olefin dihydroxylation involves cycloaddition between the photoexcited nitroarene and olefin, followed by reductive N−O bond cleavage. B) Preparation of **B1**. C) Optimisation of the reduction of **B1** to diol **1 a** and aniline **2**/**3**. D) One‐pot dihydroxylation of olefin **1** applying NaBH_4_ and Pd/C‐based conditions to in situ reductions of crude **B1**. Yields by quantitative ^1^H NMR. Yield in parentheses denotes isolated yield.

We started our investigation by converting olefin **1** into 1,3,2‐dioxazoline **B1**, a particularly stable derivative which we synthesised in high yield on gram scale, isolated by simple filtration, and stored for several months as a white solid (Scheme 2B). Pleasingly by treating **B1** with NaBH_4_ (20 equiv) in EtOH‐THF solvent at −10 °C, we immediately observed the formation of diol **1 a** in high yield (78 %), along with almost equimolar amounts of aniline **2** (70 %) (Scheme 2C, entry 1). Lowering the amount of NaBH_4_ was detrimental to reaction efficiency (entry 2), as was the use of other borohydrides (entries 3 and 4) or the removal of THF, which ensures full solubility of all components (entry 5).

Until **B1** is consumed, the reduction benefits from the low temperature to minimise the thermally‐induced C−C σ‐bond cleavage_,_ which would lead to the corresponding carbonyl products.^[15]^ Following consumption of **B1**, the reduction is most efficiently completed upon hydrolysis at room temperature, as indicated by only 22 % yield of **1 a** obtained without this final step (entry 7). Indeed, after step (i) ^1^H NMR analysis reveals a partially reduced intermediate species still compromising the aryl unit of **N1**, which subsequently undergoes efficient conversion to the diol in step (ii).^[17]^

Since the use of borohydride might be problematic with sensitive functionalities (e.g. carbonyls) in scope exploration, we also identified conditions for the conversion of **B1** to **1 a** using Pd/C with H_2_ generated in situ from KHCO_2_ and HCO_2_H (Scheme 2C, entry 7).^[18]^ This method has the advantage of obviating the use of gaseous H_2_ and delivered **1 a** and **3** in near‐quantitative yields.

A reduction in yield was observed upon exclusion of HCO_2_H, or without increasing the reduction temperature to 25 °C in step (ii)^[17]^ (entries 8 and 9).

Having developed two efficient protocols for the conversion of **B1** into **1 a**, we moved to evaluate the feasibility of a telescoped protocol where the accumulated **B1** would directly evolve into **1 a**. To simplify the experimental procedure as much as possible, it would be crucial to avoid any solvent‐switch or intermediate work‐up. Pleasingly, despite the initial photochemical step being run in EtOAc, the crude mixture could be subjected to both reduction protocols, **a** (NaBH_4_) and **b** (Pd/C), delivering **1 a** in 71 % and 88 % yield respectively (Scheme 2D). The higher yield and cleaner reduction profile observed under condition **b** led us to select the Pd/C‐based conditions for scope exploration purposes.^[19]^

With the optimised conditions in hand, we first applied our one‐pot dihydroxylation protocol to a series of terminal olefins containing different functional groups commonly encountered throughout organic synthesis (Scheme 3A). These substrates are challenging to engage in other non‐OsO_4_‐based methods but performed well under our conditions giving the desired products generally in good yields. This scope exploration demonstrated tolerance of electron withdrawing groups like nitrile (**4 a**) and acetyl (**5 a**), as well as fluoro, chloro, and bromo (**6 a**–**8 a**). This last example required the reduction to be run at lower temperature (−20 °C) to minimise dehalogenation. This unwanted reactivity was more difficult to by‐pass in the case of iodo‐derivative (**9 a**), which was obtained in a lower yield.

**Scheme 3:**
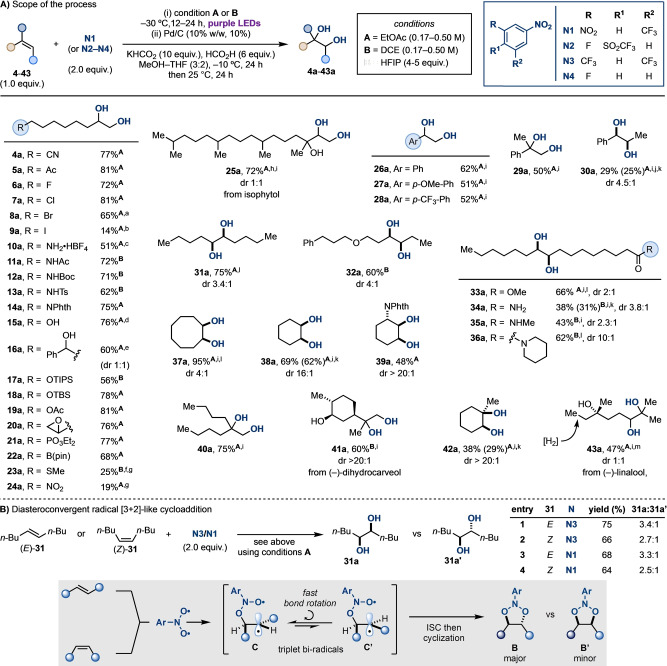
A) Scope of the one‐pot olefin dihydroxylation, see Supporting Information for full experimental conditions. ^a^−20 °C in *ii*). ^b^Olefin with 6 C linear chain in *i*). ^c^60 h irradiation in *i*). ^d^5.0 equiv HFIP added in *i*).^e^(+)‐1‐Phenylbut‐3‐en‐1‐ol with 4.0 equiv HFIP added in *i*). ^f^Perfluoro‐*tert‐*butanol used in place of HFIP in *i*). ^g^NaBH_4_ reduction used in *ii*). ^h^ **N2** used in *i*). ^i^KHCO_2_ (20 equiv), HCO_2_H (12 equiv), −20 °C in *ii*). ^j^6 h irradiation in *i*). ^k^1 atm H_2_ in *ii*) ^l^ **N3** used in *i*). ^m^ **N4** used in *i*). B) (*E*)‐ and (*Z*)‐**31** form a similar distribution of vicinal diol diastereomers. This convergent diastereoselectivity is rationalised by the stepwise nature of the photocycloaddition.

Nitrogen‐based functionalities were evaluated next and they were tolerated in the form of both free (**10 a**) and protected amine derivatives, which included amides (**11 a**), carbamates (**12 a**), tosylamides (**13 a**), and phthalimides (**14 a**). In the case of the free amine, prior protonation was required to insulate the amino group from unwanted side reactions (e.g. *N*‐oxidation). Similarly, both free alcohols (**15 a** and **16 a**) as well as protected silyl ethers (**17 a** and **18 a**) and esters (**19 a**) could be present. The successful formation of **16 a** highlights the compatibility of positions that are enthalpically activated for H‐atom transfer (HAT) by triplet nitroarenes.^[20]^ In this case, the incorporation of hexafluoroisopropanol (HFIP) as an additive during the photocycloaddition step deactivates the α‐O C(*sp* ^*3*^)−H by H‐bonding interaction with the OH group, which effectively minimises HAT by medium effects.[^15^, ^21^] Other functionalities of high synthetic value that were found compatible were epoxide (**20 a**), phosphonate (**21 a**), and pinacol boronic ester (**22 a**). In a few cases, specific functional groups, like thioether and nitro, performed well in the photocycloaddition but were not tolerated during the Pd/C reduction. Application of the NaBH_4_‐based conditions helped us circumvent this issue and access the corresponding diols (**23 a** and **24 a**), albeit in lower yields. The engagement of more complex and sterically hindered linear olefins in the dihydroxylation was demonstrated with isophytol **25**, a terpenoid alcohol produced on ton scale every year for application as a fragrance, that gave the 1,2,3‐triol **25 a** in 72 % yield.^[22]^

Having determined the functional group compatibility of the process and targeted challenging terminal olefin substrates, we evaluated the dihydroxylation of styrenes. Simple styrene as well as electron‐rich, electron‐poor, and α‐ and β‐substituted derivatives were all successfully engaged, giving **26 a**–**30 a** in good to moderate yields. In these cases, conducting the reduction at −20 °C and doubling the loading of KHCO_2_ and HCO_2_H was beneficial to speed up reduction of their corresponding 1,3,2‐dioxazolidine derivatives, which are likely more thermally labile. Likewise, increasing the hydrogen concentration also by the placement of a balloon of H_2_ further improved the yield of **30 a** from 25% to 29%.

Disubstituted olefins were evaluated next and they gave useful yields of the corresponding diols **31 a**–**36 a**. Despite **31 a** and **32 a** deriving from the dihydroxylation of (*E*)‐olefins and **33 a**–**36 a** from the functionalization of (*Z*)‐oleic acid derivatives, all products were obtained with similar dr favouring the *syn* isomer. This stereochemical outcome provides valuable insight into the mechanism of the photocycloaddition. For instance, *syn*‐diol **31 a** was obtained preferentially over *anti*‐diol **31 a′** from the reaction of nitroarene **N3** with both (*E*)‐ and (*Z*)‐5‐decene **31**. These observations can be rationalised by the fast bond rotation that equilibrates the triplet biradicals **C** and **C′** towards the less sterically hindered **C**.[^14^, ^15^, ^16^] ISC and cyclization selectively provide the cycloadduct **B** as the major component (Scheme 3B).^[23]^

The dihydroxylation process was then extended to cyclic olefins, providing the *syn* diols in high selectivity (**37 a** and **38 a**) and demonstrating strong substrate control when further stereochemical elements were present (1,2‐*anti*‐2,3‐*syn*‐diol **39 a**). This selectivity for the *cis*‐diol is in contrast to the previously reported 1 : 1 d.r. of **37 a** obtained applying alternative photocycloaddition and reduction conditions,^[24]^ suggesting the photocycloaddition and/or reduction conditions might impact the diastereoselectivity of the process.

High chemical yields were also obtained with geminal disubstituted olefins, as shown by the high‐yielding synthesis of **40 a** and of **41 a**, which derives from the acaricide monoterpenoid (−)‐dihydrocarveol. As a final element of scope exploration, we evaluated tri‐substituted olefins and found them compatible in both cyclic (**42 a**) and acyclic (**43 a**) settings, albeit in moderate yields. As previously discussed for styrene dihydroxylation (**26**–**30**), a reduction in yield can be rationalised on the basis of a competitive oxidative cleavage occurring in these substrates where hydrogenation is more difficult due to their increased steric bulk and for the decreased stability of the incipient 1,3,2‐dioxazolidine. Adding a balloon of H_2_ gas was found beneficial as, for example, it increased the yield for the formation of **42 a** from 29 to 38%.

In line with our findings on the oxidative cleavage processes,^[15]^ the dihydroxylation of (−)‐linalool **43** was selective for the tri‐substituted olefin over the terminal one, that however was hydrogenated during the reduction step. We believe the ability to tune the electronic properties of the nitroarenes will be a useful tool to increase the photocycloaddition selectivity, which will enable the selective dihydroxylation of a specific olefin over others in substrates containing more than one C−Cπ site.

Olefin dihydroxylation is generally approached using OsO_4_ or other highly oxidised metal‐systems. Herein, we have developed an alternative one‐pot strategy that uses nitroarenes as safe and easy‐to‐handle photoresponsive oxidants. The key 1,3,2‐dioxazolidine intermediates formed under our mild photochemical conditions are relatively stable, and this has enabled us to channel their reactivity towards N−O bond hydrogenation to the desired vicinal diols. The process requires straightforward equipment for low temperature reductions and uses convenient solid reagents as H_2_ surrogates. Overall, this reactivity enabled functionalization of a broad range of olefin classes and was tolerant of many valuable organic functionalities. We therefore hope that the benign reaction conditions and the generality of this strategy will render it a viable alternative to traditional approaches based on toxic reagents and highly oxidising conditions.

## Conflict of interest

The authors declare no conflict of interest.

## Supporting information

As a service to our authors and readers, this journal provides supporting information supplied by the authors. Such materials are peer reviewed and may be re‐organized for online delivery, but are not copy‐edited or typeset. Technical support issues arising from supporting information (other than missing files) should be addressed to the authors.

Supporting Information

## Data Availability

The data that support the findings of this study are available in the Supporting Information of this article.
