# Supplementary material for: Olefin Dihydroxylation Using Nitroarenes as Photoresponsive Oxidants
Source: Angew Chem Int Ed Engl. 2023 Jan 17;62(8):e202214508. doi: 10.1002/anie.202214508 (PMC10107662; doi:10.1002/anie.202214508)
Supplement: Supplementary file 1 — Supporting Information [file ANIE-62-0-s001.pdf]

Angewandte Chemie
Eine Zeitschrift der Gesellschaft Deutscher Chemiker

Supporting Information

Olefin Dihydroxylation Using Nitroarenes as Photoresponsive Oxidants

C. Hampton, M. Simonetti, D. Leonori**

Table of Contents

1 General Information 3

2 General Procedures 4

2.1 **GP1**: General Procedure for Olefin Dihydroxylation using $NaBH_4$ Reduction 4

2.2 **GP2**: General Procedure for Olefin Dihydroxylation using Pd/C, $KHCO_2$, and HCO_2H Reduction 4

3 Optimisation for 1,3,2-Dioxazolidine **B1** Reduction 7

3.1 **GP3**: General Procedure for Optimisation of 1,3,2-Dioxazolidine **B1** Reduction using Borohydride 7

3.2 **GP4**: General Procedure for Optimisation of 1,3,2-Dioxazolidine **B1** Reduction using Pd/C 8

3.3 Alternative Reductants 9

4 Reduction of 1,3,2-Dioxazolidine **B1**: Sequential N–O Cleavage 10

4.1 Reduction Using Borohydride 10

4.2 Reduction Using Pd/C 11

5 Effect of Olefin Substitution on 1,3,2-Dioxazolidine Reduction 12

6 Unsuccessful Substrates 14

7 Characterisation 15

7.1 Characterisation of Vicinal Diols 15

7.2 Characterisation of Anilines 28

8 NMR Spectra 30

8.1 NMR Spectra of Vicinal Diols 30

8.2 NMR Spectra of Anilines 77

9 References 85

1 General Information

All air and moisture sensitive reactions were carried out under nitrogen atmosphere using standard Schlenk manifold technique. All required fine chemicals were purchased from Acros (Fisher), Aldrich (Merck), Alfa Aesar (Fisher), Fluorochem, Generon, Manchester Organics, MP Biomedicals, and TCI, and used without further purification unless otherwise stated. All dry solvents were bought from Acros as 99.8% purity and degassed with 3 freeze-pump-thaw cycles prior to use. 1,3,2-Dioxazolidines **B1**, **B2**, and **B3** and olefins **4**, **6**, **10**–**14**, **18**, **19**, **21**–**24**, **32**, **35**, **36**, **39** were synthesised according to literature procedure.[1,2] ^{1}H and ^{13}C Nuclear Magnetic Resonance (NMR) spectra were acquired at various field strengths as indicated. ^{1}H NMR are referenced to the residual solvent peak at 7.26 ppm ($CDCl_3$), 1.94 ppm (CD_3CN), 3.31 ppm (CD_3OD), or 7.16 ppm (C_6D_6). ^{13}C NMR are referenced to the residual solvent peak at 77.0 ppm ($CDCl_3$), 1.32 ppm (CD_3CN), 49.00 ppm (CD_3OD), or 128.06 ppm (C_6D_6). ^{1}H NMR coupling constants are reported in Hertz and refer to apparent multiplicities and not true coupling constants. Data are reported as follows: chemical shift, integration, multiplicity (br = broad signal, s = singlet, d = doublet, t = triplet, q = quadruplet, p = pentet, m = multiplet, dd = doublet of doublets, etc).^{1}H NMR chemical shifts of O–H and N–H signals is prone to variation dependent on solvent, concentration etc and are typically broad or not observed due to H/D exchange. Unless stated otherwise, diastereomeric ratios of diol products determined by ^{1}H NMR using C**H**OH signals. High-resolution mass spectra were obtained using a JEOL JMS-700 spectrometer or a Fissions VG Trio 2000 quadrupole mass spectrometer. Spectra were obtained using atmospheric-pressure chemical ionization (APCI), atmospheric solids analysis probe (ASAP), or electrospray (ES) techniques. Analytical TLC: aluminium backed plates pre-coated (0.25 mm) with Merck Silica Gel 60 F254. Compounds were visualised by exposure to UV-light or by dipping the plates in *p*-anisaldehyde or permanganate stains followed by heating. Flash column chromatography was performed using Merck Silica Gel 60 (40–63 μm). All mixed solvent eluents are reported as v/v solutions. All one-pot olefin dihydroxylations were conducted in CEM 10 mL glass microwave tubes with Schlenk adaptation and sealed using a Supelco butyl rubber stopper (SKU-27232) with aluminium open centre crimp seal (SKU-27099-U). Photocycloadditions run using Huber TC45E immersion cooler in a Huber 220B (20 L) insulated stainless steel bath filled with MeOH and equipped with a mechanical stirrer, which was placed on two magnetic stirring plates. Light source is Kessil PR160L-390nm LEDs. Reductions run using Huber Ministat 230 circulating chiller unit connected to a custom-made hollow aluminium block which was placed on one magnetic stirring plate, fitted with aluminium vial holders made in-house, and used MeOH as the thermal fluid.

2 General Procedures

2.1 GP1: General Procedure for Olefin Dihydroxylation using $NaBH_4$ Reduction

Unless stated otherwise, an oven dried Schlenk microwave vial equipped with a stirring bar was charged with the olefin (0.2 mmol, 1 equiv.), if solid, and 3,5-dinitrobenzotrifluoride **N1** (94 mg, 0.4 mmol, 2 equiv.). The vial was capped and connected to the Schlenk line, and 3 cycles of vacuum/nitrogen were run. The olefin (1 equiv.) was added, if liquid. Solvent A (EtOAc or CH_2Cl_2; 0.6 mL) and the additive (perfluoro-*tert*-butylalcohol; 110 µL, 0.8 mmol, 4 equiv.), if applicable, were added. The cap was replaced under a positive pressure of nitrogen, the vial was disconnected from the Schlenk line, and the mixture was cooled to −30 °C. The LEDs (390 nm) were switched on to 100% intensity, and the mixture gently stirred under irradiation at −30 °C for 24 h. Next, the light was turned off and the vials placed at −10 °C. The cap was removed and cold EtOH:THF 3:2 (2 mL, −10 °C) was added, followed by slow addition of $NaBH_4$ (151 mg, 20 equiv.). The cap was replaced and the mixture was vigorously stirred for 24 h at −10 °C. Next, H_2O (1 mL) was added, and the vial transferred to a heating block at 25 °C where it was vigorously stirred for a further 24 h. The crude mixture was then concentrated under reduced pressure. Next, brine (2 mL) and EtOAc (20 mL) were added, the layers were separated, and the aqueous layer was further extracted with EtOAc (2 × 20 mL). The combined organic layers were filtered over SiO_2, and the filter cake washed with EtOAc. The filtrate was concentrated under reduced pressure. Purification by silica gel chromatography furnished the desired products.

2.2 GP2: General Procedure for Olefin Dihydroxylation using Pd/C, $KHCO_2$, and HCO_2H Reduction

Unless stated otherwise, an oven dried Schlenk microwave vial equipped with a stirring bar was charged with the olefin (0.2 mmol, 1 equiv.), if solid, and 3,5-dinitrobenzotrifluoride **N1** (94 mg, 0.4 mmol, 2 equiv.). The vial was capped and connected to the Schlenk line, and 3 cycles of vacuum/nitrogen were run. The olefin (1 equiv.) was added, if liquid. Solvent A (EtOAc or DCE; 0.6 mL) and the additive (1,1,1,3,3,3-hexafluoro-2-propanol (HFIP); 2−5 equiv.), if applicable, were added. The cap was replaced under a positive pressure of nitrogen, the vial was disconnected from the Schlenk line, and the mixture was cooled to −30 °C. The LEDs (390 nm) were switched on to 100% intensity, and the mixture gently stirred under irradiation at −30 °C for 24 h. Next, the light was turned off and the vials placed at −10 °C. The cap was removed and cold MeOH:THF 3:2 (4 mL, −10 °C) was added, followed by $KHCO_2$ (168 mg, 2 mmol, 10 equiv.) and palladium on activated charcoal (Pd/C; 10% Pd basis; 10% wt with respect to theoretical 100% yield of intermediate 1,3,2-dioxazolidine). The cap was replaced and the vial was connected to the Schlenk line and 3 cycles of vacuum/nitrogen were run as quickly as possible. The vial under N_2 was disconnected from the line. Then HCO_2H (45 µL, 1.2 mmol, 6 equiv.) was added under vigorous stirring, which was maintained for 24 h at −10 °C. The vials were transferred to a heating block at 25 °C and stirred for a further 24 h. Then the mixture was filtered over Celite, washed with THF, and concentrated under reduced pressure. Purification by silica gel chromatography furnished the desired products.

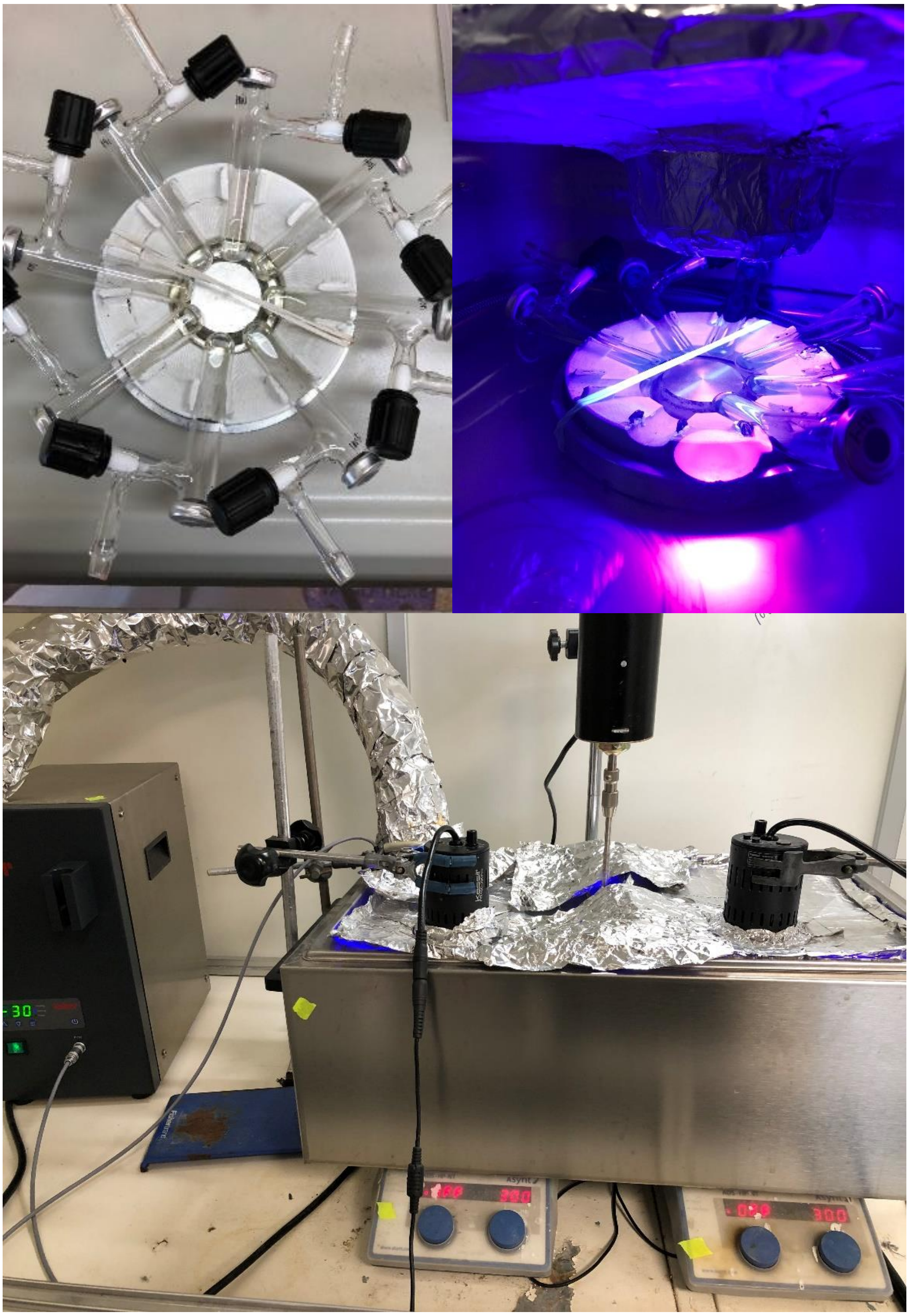

Figure S1

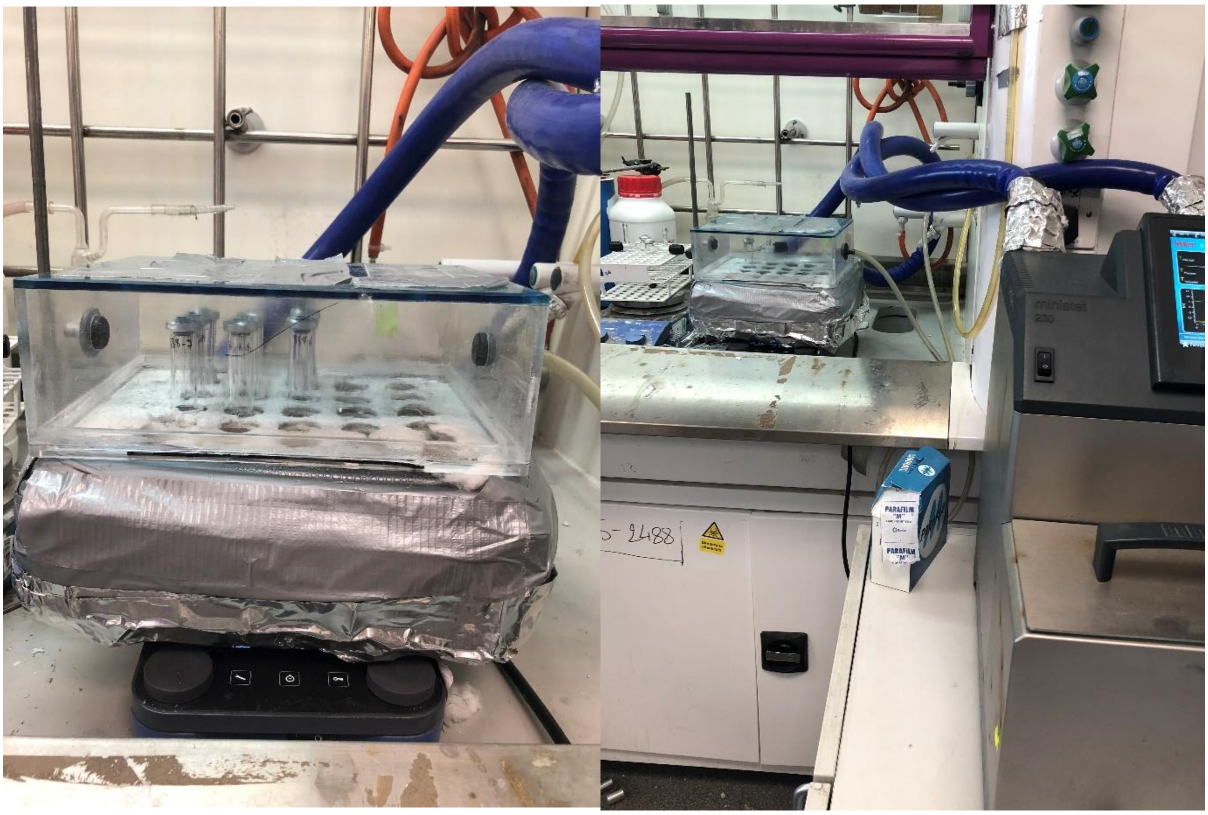

Figure S2

3 Optimisation for 1,3,2-Dioxazolidine B1 Reduction

3.1 GP3: General Procedure for Optimisation of 1,3,2-Dioxazolidine B1 Reduction using Borohydride

Unless stated otherwise, an oven dried microwave vial equipped with a stirring bar was charged with 4-decyl-2-(3-nitro-5-(trifluoromethyl)phenyl)-1,3,2-dioxazolidine **B1** (10.1 mg, 0.05 mmol, 1.0 equiv.) and placed –10 °C. The given solvent was also cooled to –10 °C, then was added to the reaction vial. Next, the hydride source was slowly added and the vial immediately sealed. The mixture was stirred vigorously for 24 h at the given temperature. Then, if required, H_2O (250 μL) was added and the mixture stirred at 25 °C for a further 24 h. The crude mixture was concentrated under reduced pressure, and a stock solution containing *p*-tolyl ether internal standard (0.1 M in EtOAc, 0.25 mL, 0.5 equiv.) was added. Next, brine (1 mL) and EtOAc (10 mL) were added, the layers were separated, and the aqueous layer was further extracted with EtOAc (2 × 10 mL). The combined organic layers were filtered over SiO_2, and the SiO_2 washed with EtOAc. The filtrate was concentrated under reduced pressure. $CDCl_3$ (0.6 mL) was added and the sample transferred into an NMR tube for quantitative analysis.

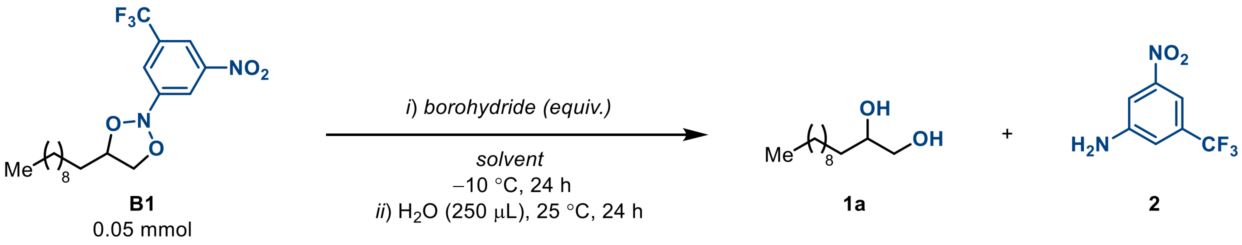

entry	*borohydride*	(equiv.)	*solvent*	*ii)*	**1a** (%)	**2** (%)
1	$NaBH_4$	20	MeOH	H_2O, 24 h	2	0
2	$NaBH_4$	20	EtOH	H_2O, 24 h	44	20
3	$NaBH_4$	20	EtOH:THF 4:1	H_2O, 24 h	68	58
4	$NaBH_4$	20	EtOH:THF 3:2	H_2O, 24 h	78	70
5	$NaBH_4$	20	THF	H_2O, 24 h	40	27
6	$NaBH_4$	10	EtOH:THF 3:2	H_2O, 24 h	32	1
7	$NaBH_4$	5	EtOH:THF 3:2	H_2O, 24 h	27	3
8	$NaBH(OAc)_3$	20	EtOH:THF 3:2	H_2O, 24 h	8	1
9	$NaBH_3CN$	20	EtOH:THF 3:2	H_2O, 24 h	18	0
10	$NaBH_4$	20	EtOH:THF 3:2	H_2O, 1 h	30	13
11	$NaBH_4$	20	EtOH:THF 3:2	H_2O, 80 °C, 1 h	24	7
12	$NaBH_4$	20	EtOH:THF 3:2	None	22	30
13[b]	$NaBH_4$	20	EtOH:THF 3:2	H_2O, 24 h	72	66
14[c]	$NaBH_4$	20	EtOH:THF 3:2	H_2O, 24 h	71(66)	70

Table S1. [b]EtOAc (150 μL) added to solvent. [c]Crude **B1** deriving from photocycloaddition between olefin **1** (1.0 equiv.) and nitroarene **N1** (2.0 equiv.) in EtOAc (0.33 M). Yield in parentheses denotes isolated yield.

3.2 GP4: General Procedure for Optimisation of 1,3,2-Dioxazolidine B1 Reduction using Pd/C

Unless stated otherwise, an oven dried microwave vial equipped with a stirring bar was charged with 4-decyl-2-(3-nitro-5-(trifluoromethyl)phenyl)-1,3,2-dioxazolidine **B1** (10.1 mg, 0.05 mmol, 1.0 equiv.) and placed at the stated temperature. The given solvent was cooled to this temperature, then was added to the reaction vial (1 mL). Next, the palladium on activated charcoal (Pd/C, 10% Pd basis, 10% wt) and the given formate (equiv.) was added. The vial was sealed, connected to the Schlenk line via a needle through the septum, and 3 cycles of vac/N_2 were run. Next, the mixture was stirred vigorously whilst HCO_2H (equiv.) was added. The mixture was then left to stir vigorously for the given time at the stated temperature then, if necessary, for a further 24 h at 25 °C. Next, a stock solution containing *p*-tolyl ether as internal standard (0.1 M in EtOAc, 0.25 mL, 0.5 equiv.) was added, and the mixture was filtered over Celite. The filter cake was washed with THF (30 mL), and the filtrate concentrated under reduced pressure. $CDCl_3$ (0.6 mL) was added and the sample transferred into an NMR tube for quantitative analysis.

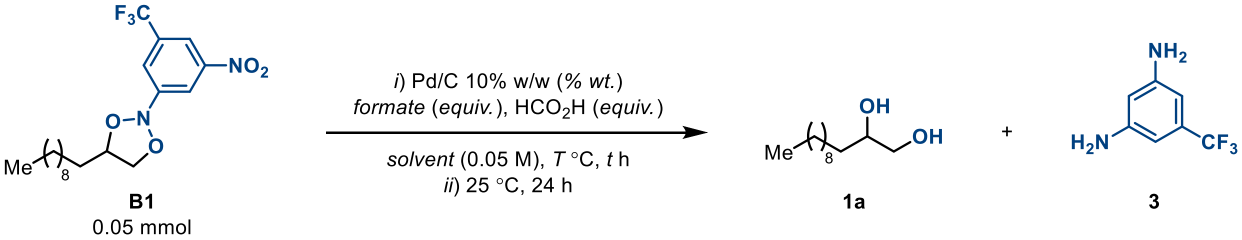

entry	Pd/C (% wt)	*formate*	(equiv.)	HCO_2H (equiv.)	*solvent*	*T* / °C	*t* / h	*ii)*	**1a** (%)	**3** (%)
1	10	$KHCO_2$	10	6	EtOH:THF 3:2	–10	16	×	67	81
2	10	NH_4HCO_2	10	6	EtOH:THF 3:2	–10	16	×	76	88
3	10	NH_4HCO_2	10	6	EtOH:THF 3:2	–20	16	×	43	25
4	10	$KHCO_2$	10	6	EtOH:THF 3:2	–20	16	×	64	78
5	10	$KHCO_2$	10	12	EtOH:THF 3:2	–20	16	×	52	48
6	10	$KHCO_2$	20	6	EtOH:THF 3:2	–20	16	×	28	11
7	10	$KHCO_2$	20	12	EtOH:THF 3:2	–20	16	×	70	59
8	10	$KHCO_2$	10	3	EtOH:THF 3:2	–10	16	×	62	68
9	10	$KHCO_2$	10	12	EtOH:THF 3:2	–10	16	×	11	86
10	10	$KHCO_2$	10	18	EtOH:THF 3:2	–10	16	×	11	85
11	10	$KHCO_2$	10	6	MeOH:THF 3:2	–10	16	×	76	92
12	10	$KHCO_2$	10	6	*i*-PrOH:THF 3:2	–10	16	×	53	23
13	10	$KHCO_2$	10	6	THF	–10	16	×	61	3
14	10	$KHCO_2$	10	6	MeOH:THF 3:2	–10	24	×	78	90
15	10	$KHCO_2$	10	6	MeOH:THF 3:2	–10	8	×	21	1
16	10	$KHCO_2$	10	6	MeOH:THF 3:2	–10	24	✓	98	97
17	10	$KHCO_2$	10	0	MeOH:THF 3:2	–10	24	✓	63	78
18	10	$KHCO_2$	10	6	MeOH	–10	24	✓	57	91
19	10	$KHCO_2$	10	6	EtOH:THF 3:2	–10	24	✓	75	82
20	20	$KHCO_2$	10	6	EtOH:THF 3:2	–10	24	✓	91	92
21[b]	10	$KHCO_2$	10	6	MeOH:THF 3:2	–10	24	✓	91	86
22[c]	10	$KHCO_2$	10	6	MeOH:THF 3:2	–10	24	✓	92	80
23[d]	10	$KHCO_2$	10	6	MeOH:THF 3:2	–10	24	✓	88	91

Table S2. [b]EtOAc (150 µL) added to solvent. [c]DCE (150 µL) added to solvent. [d]Crude **B1**. deriving from photocycloaddition between olefin **1** (1.0 equiv.) and nitroarene **N1** (2.0 equiv.) in EtOAc (0.33 M).

3.3 Alternative Reductants

A series of alternative reducing agents were preliminarily screened to assess their potential in conversion of **B1** into **1a**.

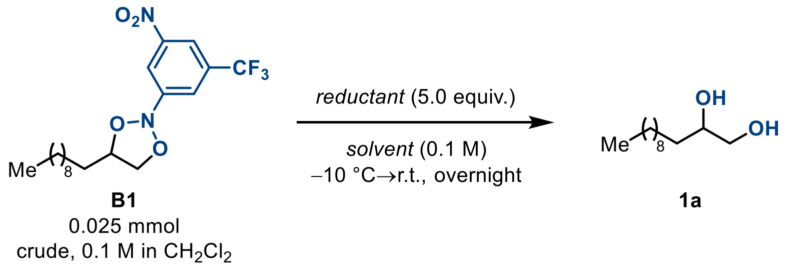

entry	*reductant*	*solvent*	**1a** (%)
1[b]	$(OEt)_2MeSiH$	CH_2Cl_2	0
2[b]	Ph_2SiH_2	CH_2Cl_2	0
3[b]	$(TMS)_3SiH$	CH_2Cl_2	0
4[b]	$PhMe_2SiH$	CH_2Cl_2	0
5[b]	Ph_3SiH	CH_2Cl_2	0
6[b]	$PhSiH_3$	CH_2Cl_2	0
7[b]	SMe_2	CH_2Cl_2	0
8[c]	BH_3-THF	THF	0
9[c]	BH_3-SMe_2	THF	trace
10[c,d]	BH_3-SMe_2	THF	22

Table S3. [a]Step *i*) of **GP3** was followed with the above modifications. In all cases, complex mixtures of nitroarene derivatives were observed. [b]At the end of the reaction GC-MS samples were prepared by dilution with EtOAc. [c]Isolated solid **B1** used, and H_2O was added at the end of the reaction before performing the workup described in **GP3** to give crude ^{1}H NMR yield using *p*-tolyl ether internal standard. [d]Additional step performed adding MeOH (1 mL), H_2O (1 mL), and K_2CO_3 (5.0 equiv.) overnight at room temperature.

In all cases, complex mixtures of products and minimal conversion towards **1a** was observed. No further optimisation was carried out using these reducing agents, and improved results may be possible upon further investigation.

4 Reduction of 1,3,2-Dioxazolidine B1: Sequential N–O Cleavage

4.1 Reduction Using Borohydride

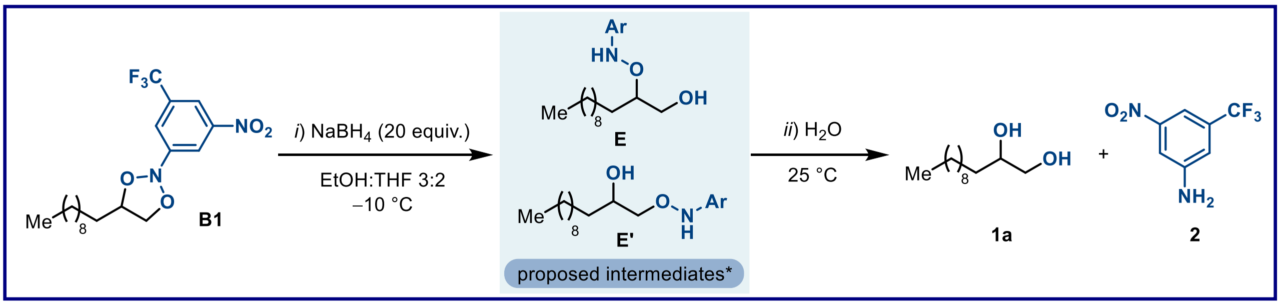

Scheme S1

During optimisation, ^{1}H NMR spectra obtained at the end of step *i*) displayed signals which could correspond to intermediate **E**/**E'**, denoting cleavage of one N–O bond of **B1** (Figure S3a). Indeed, after step *i*), **B1** was completely consumed and **E**/**E'** was the major species present alongside low levels of desired diol **1a** and aniline **2**, in addition to other unknown nitroarene derivatives (Figure S3b). It was therefore observed that further reduction of **E**/**E'** at 25 °C in step *ii*) in the presence of water increased efficiency of conversion to **1a** and **2**.

*Whilst ^{1}H NMR data are could be in accordance with intermediate **E**/**E'**, alternative structures, such as those involving boron complexation, have not been ruled out at this stage.

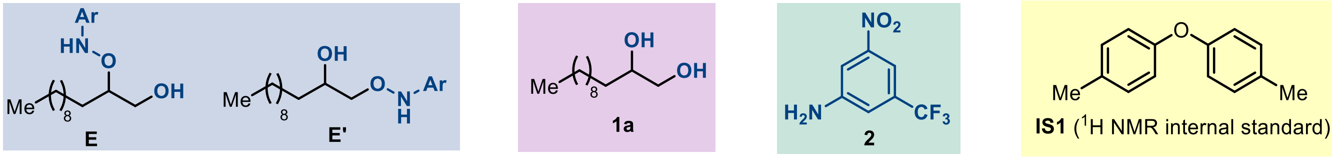

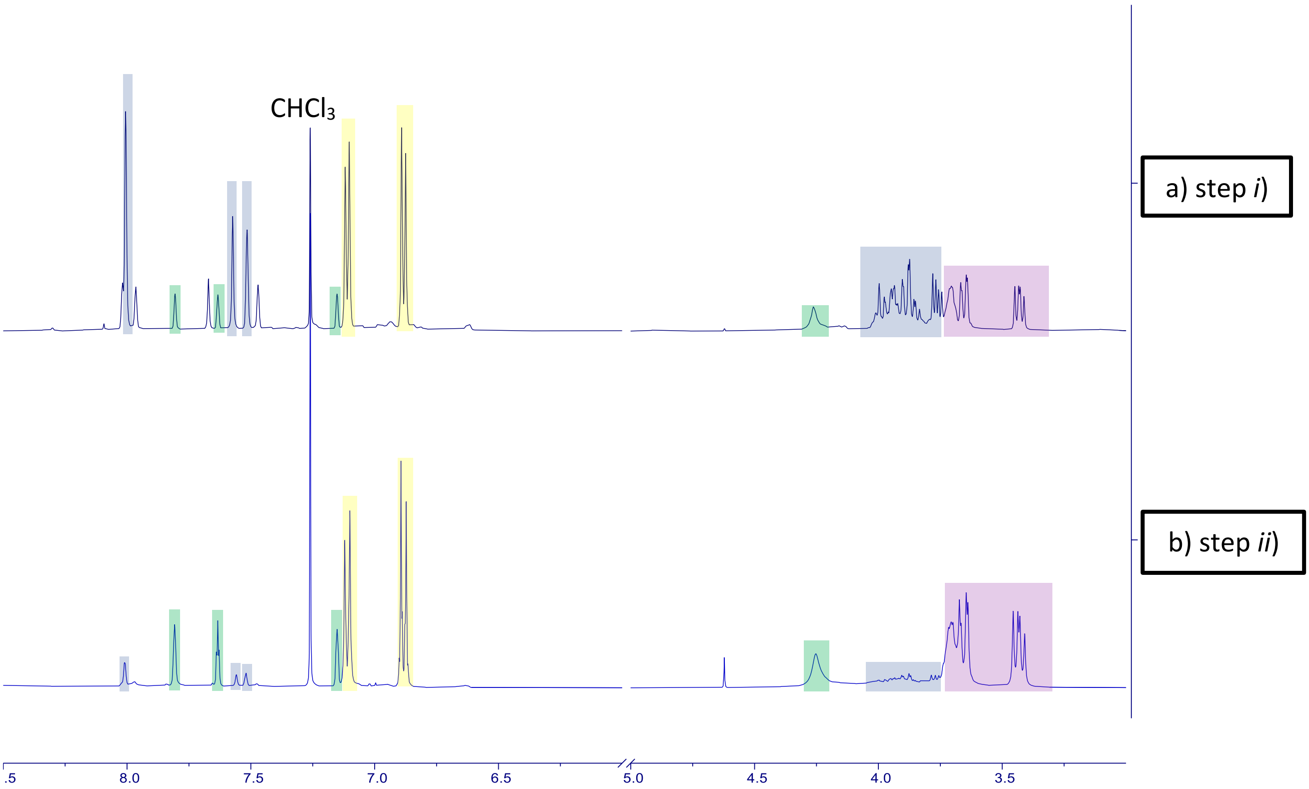

Figure S3

4.2 Reduction Using Pd/C

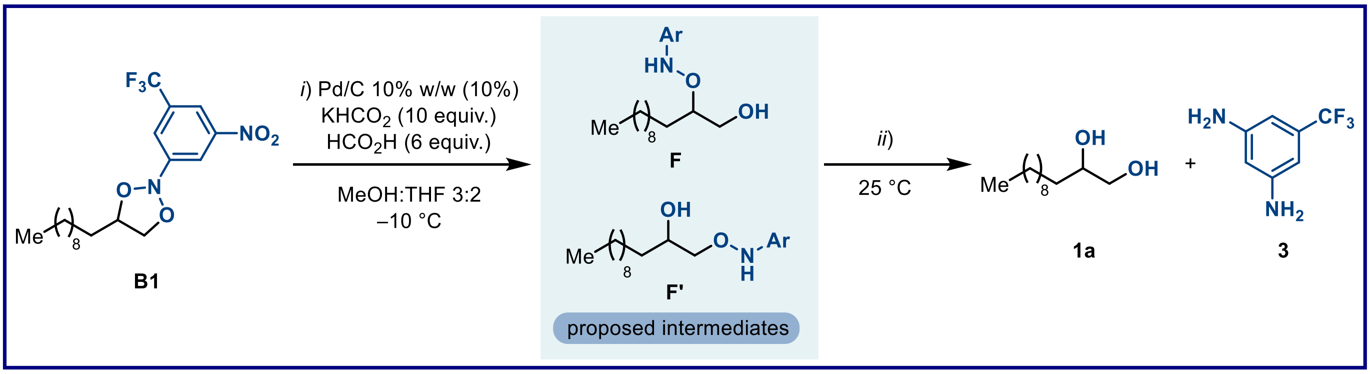

Scheme S2

Upon reduction of **B1** using Pd/C conditions, comparable proposed intermediates to those using $NaBH_4$ are observed. Therefore in these conditions it is similarly proposed that in *i*) at low temperature, **B1** is reduced to **F**/**F'** by cleavage of one N–O bond (Figure S4a) and in step *ii*) further reduction of **F**/**F'** generates diol **1a** and bis-aniline **3** (Figure S4b).

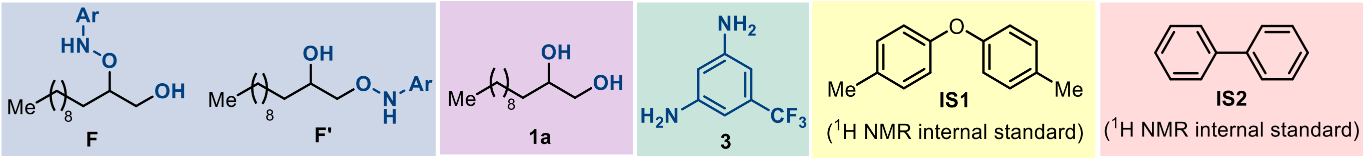

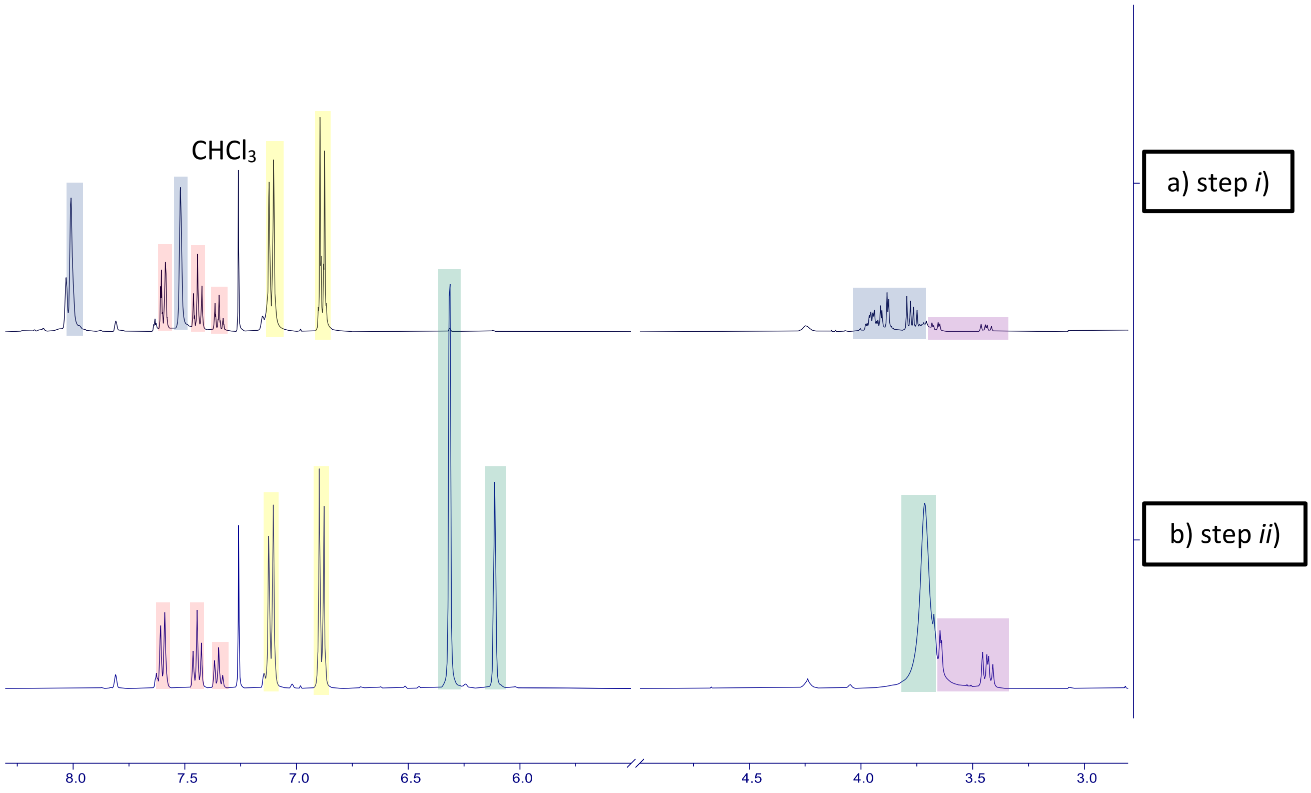

Figure S4

5 Effect of Olefin Substitution on 1,3,2-Dioxazolidine Reduction

In addition to model substrate **B1**, representative examples of 1,3,2-dioxazolidines deriving from increasingly substituted olefins were subjected to $NaBH_4$ and Pd/C reductions.

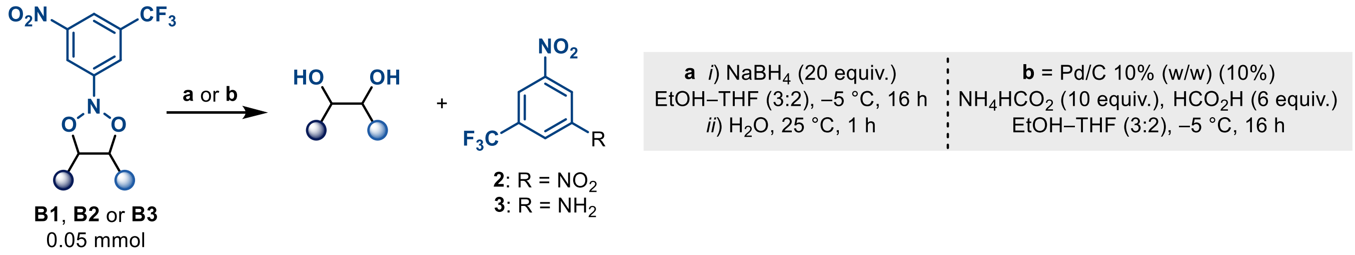

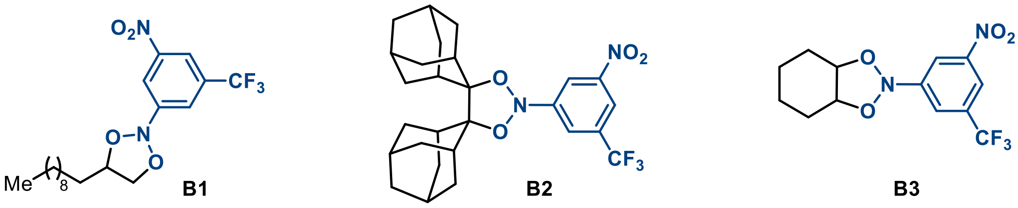

entry	B	reduction	diol (%)	dr	**2**/**3** (%)
1	**B1**	**a**	51	-	31
2	**B1**	**b**	77	-	76
3	**B2**	**a**	24	-	7
4	**B2**	**b**	70	-	47
5	**B3**	**a**	20	>16:1	4
6	**B3**	**b**	28	>16:1	51

Table S4. [a]**GP3** and **GP4** were followed with the above modifications. Crude ^{1}H NMR yield using *p*-tolyl ether internal standard.

Additionally, the following substrates were subjected to one-pot dihydroxylations using both the $NaBH_4$ and Pd/C reductions.

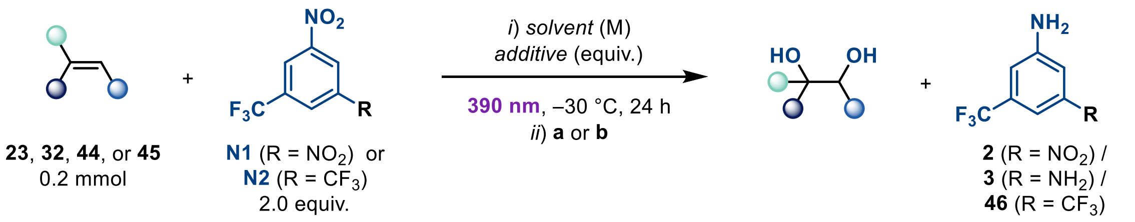

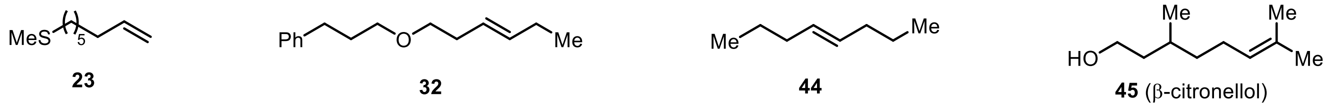

**a** *i*) $NaBH_4$ (20 equiv.) EtOH–THF (3:2), –20 °C, 24 h *ii*) H_2O, 25 °C, 24 h	**b** = *i*) Pd/C 10% (w/w) (10%) $KHCO_2$ (20 equiv.), HCO_2H (12 equiv.) MeOH–THF (3:2), –20 °C, 24 h *ii*) 25 °C, 24 h

entry	olefin	N	*solvent*	(M)	*additive*	(equiv.)	*ii*)	diol (%)	dr	**2/3/46** (%)
1[b]	**23**	**N1**	CH_2Cl_2	0.17	PFTB	4.0	**a**	25[c]	-	-
2[b]	**23**	**N1**	DCE	0.17	PFTB	4.0	**b**	0	-	0
3[d]	**32**	**N2**	CH_2Cl_2	0.5	HFIP	4.0	**a**	27	ND	ND
4[d,e]	**32**	**N2**	DCE	0.5	HFIP	4.0	**b**	67	3.0:1	41
5	**44**	**N1**	EtOAc	0.33	-	-	**a**	28	2.5:1	25
6	**44**	**N1**	EtOAc	0.33	-	-	**b**	67	2.5:1	57
7	**45**	**N1**	CH_2Cl_2	0.25	HFIP	2.0	**a**	0	-	10
8	**45**	**N1**	DCE	0.25	HFIP	2.0	**b**	25	ND	114

Table S5. [a]**GP1** and **GP2** were followed with the above modifications and without product isolation. Crude ^1H NMR yields using *p*-tolyl ether internal standard. ND = not determined: dr of diol and/or yield of **2**/**3**/**46** indistinguishable by ^1H NMR. [b]10 equiv. $KHCO_2$, 6.0 equiv. HCO_2H at –10 °C in *ii*). [c]Isolated yield (as seen in Scheme 3A). [d]0.1 mmol nitroarene, 2.0 equiv. olefin, 6 h irradiation in *i*). [e]H_2 balloon added in *ii*). [f]Expected bis-aniline **3** not detected despite high conversion to diol.

Olefin **23** proved incompatible with the Pd/C reduction, possibly as a result of catalyst poisoning by the thioether[3]

Bis-substituted olefins such as **32** and **44** show marked improvement upon using Pd/C versus $NaBH_4$. This is also seen in tris-substituted **46**, where indeed no desired diol was formed using $NaBH_4$.

Therefore $NaBH_4$ consistently performed worse upon increased olefin substitution/steric bulk in both reductions of isolated 1,3,2-dioxazolidines **B** and in one-pot olefin dihydroxylations.

6 Unsuccessful Substrates

The following substrates were subjected to one-pot olefin dihydroxylations, and not studied further due to minimal desired reactivity.

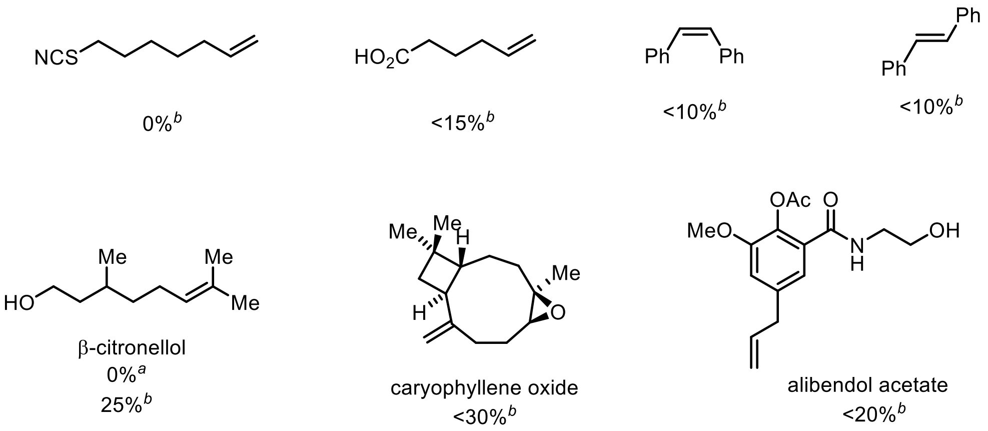

Scheme S3. ^{1}H-NMR Yields of crude reactions run with 2 equiv. of $ArNO_2$ and 1 equiv. of olefin. Yields estimated without product isolation and could therefore be incorrect. [a]Reduction using $NaBH_4$. [b]Reduction using Pd/C.

7 Characterisation

7.1 Characterisation of Vicinal Diols

Dodecane-1,2-diol (1a)

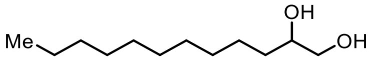

GP1 was followed using 1-dodecene **1** (45 μL, 99% purity) in EtOAc. Dodecane-1,2-diol **2** was isolated by silica gel chromatography (EtOAc in *n*-hexane, 40%) as a white solid (27 mg, 66%).

^{1}H NMR ($CDCl_3$, 400 MHz) δ 3.72–3.62 (m, 2H), 3.42 (1H, dd, *J* = 10.4, 7.9 Hz), 2.58 (2H, br s, O–H), 1.45–1.22 (18H, m), 0.87 (3H, t, *J* = 7.0 Hz); ^{13}C NMR (126 MHz, $CDCl_3$) 72.5, 67.0, 33.3, 32.0, 29.8, 29.8 (2C), 29.7, 29.5, 25.7, 22.8, 14.2.

Data in accordance with literature.[4] Commercially available CAS 1119-87-5.

8,9-Dihydroxynonanenitrile (4a)

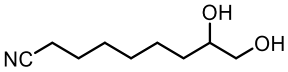

GP2 was followed using non-8-enenitrile **4** (33 μL) in EtOAc. 8,9-Dihydroxynonanenitrile **4a** was isolated by silica gel chromatography (EtOAc in CH_2Cl_2, 0–50%) as a yellow oil (26 mg, 77%).

^{1}H NMR (500 MHz, $CDCl_3$) δ 3.69–3.59 (2H, m), 3.40 (1H, dd, *J* = 11.0, 7.8 Hz), 2.86 (2H, br s, O–H), 2.33 (2H, t, *J* = 7.1 Hz), 1.64 (2H, app p), 1.48–1.31 (8H, m); ^{13}C NMR (101 MHz, $CDCl_3$) δ 120.0, 72.2, 66.8, 33.0, 28.8, 28.6, 25.3, 25.3, 17.2; HRMS (ESI^{+}): found MNa+ 194.1148, $[C_9H_{18}NO_2Na]^+$ requires 194.1152.

9,10-Dihydroxydecan-2-one (5a)

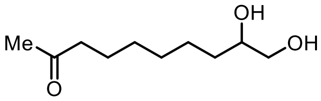

GP2, was followed using dec-9-en-2-one **5** (39 μL) in EtOAc (0.4 mL). 9,10-Dihydroxydecan-2-one **5a** was isolated by silica gel chromatography (MeOH in CH_2Cl_2, 6%) as a colourless oil (30 mg, 81%).

^{1}H NMR (400 MHz, C_6D_6) δ 3.59–3.52 (1H, m), 3.46 (1H, dd, *J* = 11.0, 3.2 Hz), 3.31 (1H, dd, *J* = 10.9, 7.5 Hz), 2.58 (2H, br s, O–H), 1.94 (2H, t, *J* = 7.3 Hz), 1.67 (3H, s), 1.49–1.09 (10H, m); ^{13}C NMR (101 MHz, C_6D_6) δ 206.9, 72.4, 67.1, 43.4, 33.5, 29.8, 29.4 (2C), 25.8, 23.9; HRMS (ESI^{+}): found MNa+ 211.1296, $[C_{10}H_{20}O_3Na]^+$ requires 211.1305.

8-Fluorooctane-1,2-diol (6a)

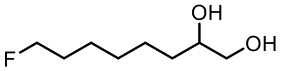

GP2 was followed using 8-fluorooct-1-ene **6** (30 μL) in EtOAc (0.4 mL). 8-Fluorooctane-1,2-diol **6a** was isolated by silica gel chromatography (EtOAc in *n*-hexane, 50%) as a yellow oil (24 mg, 72%).

^{1}H NMR (400 MHz, $CDCl_3$) δ 4.44 (2H, dt, *J* = 47.4, 6.1 Hz), 3.74–3.64 (2H, m), 3.44 (1H, dd, *J* = 10.9, 7.6 Hz), 2.03 (2H, br s, O–H), 1.76–1.63 (2H, dm, *J* = 25.1 Hz), 1.50–1.33 (8H, m); ^{13}C NMR (101 MHz, $CDCl_3$) δ 84.3 (d, *J* = 164.1 Hz), 72.4, 67.0, 33.2, 30.5 (d, *J* = 19.5 Hz), 29.4, 25.6, 25.3 (d, *J* = 5.4 Hz); ^{19}F NMR (376 MHz, $CDCl_3$) δ –218.2 (tt, *J* = 47.3, 25.2 Hz); ^{19}F {^{1}H} (376 MHz, $CDCl_3$) δ –218.2; HRMS (ESI^{+}): found MNa+ 187.1103, $[C_8H_{17}FO_2Na]^+$ requires 187.1105.

8-Chlorooctane-1,2-diol (7a)

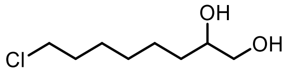

GP2 was followed using 8-chlorooct-1-ene **7** (29 mg) in EtOAc. 8-Chlorooctane-1,2-diol **7a** was isolated by silica gel chromatography (EtOAc in *n*-hexane, 70%) as a pale yellow oil (29 mg, 81%).

^{1}H NMR (500 MHz, $CDCl_3$) δ 3.73–3.68 (1H, m), 3.66 (1H, dd, *J* = 11.0, 2.7 Hz), 3.53 (2H, t, *J* = 6.7 Hz), 3.43 (1H, dd, *J* = 11.0, 7.6 Hz), 2.10 (2H, br s, O–H), 1.80–1.74 (2H, m), 1.49–1.31 (8H, m); ^{13}C NMR (101 MHz, $CDCl_3$) δ 72.4, 67.0, 45.2, 33.2, 32.6, 29.0, 26.9, 25.5; HRMS (ESI$^+$): found MNa+ 203.0804, $[C_8H_{17}ClO_2Na]^+$ requires 203.0809.

8-Bromooctane-1,2-diol (8a)

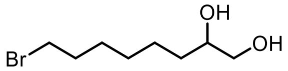

GP2 was followed using 8-bromooct-1-ene **8** (34 µL, 0.2 mmol) in EtOAc. Then –20 °C reduction. 8-Bromooctane-1,2-diol **8a** was isolated by silica gel chromatography (EtOAc in *n*-hexane, 50%) as a yellow oil (29 mg, 65%).

^{1}H NMR (500 MHz, $CDCl_3$) δ 3.74–3.69 (1H, m), 3.66 (1H, dd, *J* = 10.9, 3.0 Hz), 3.44 (1H, dd, *J* = 10.9, 7.6 Hz), 3.41 (2H, t, *J* = 6.8 Hz), 2.07–1.82 (4H, m, 2 × C–H; 2 × O–H), 1.50–1.31 (8H, m); ^{13}C NMR (101 MHz, $CDCl_3$) δ 72.3, 67.0, 34.1, 33.2, 32.8, 28.9, 28.2, 25.5.

Data in accordance with literature.[5]

6-Iodohexane-1,2-diol (9a)

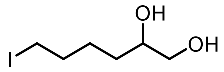

GP2 was followed using 6-iodohex-1-ene **9** (42 mg) in EtOAc. 6-Iodohexane-1,2-diol **9a** was isolated by silica gel chromatography (MeOH in CH_2Cl_2, 6%) as a yellow oil (7 mg, 14%).

^{1}H NMR (500 MHz, $CDCl_3$) δ 3.76–3.70 (1H, m), 3.68 (1H, dd, *J* = 10.9, 3.0 Hz), 3.46 (1H, dd, *J* = 10.9, 7.5 Hz), 3.20 (2H, t, *J* = 6.9 Hz), 1.91–1.81 (2H, m), 1.63–1.52 (4H, m, 2 × C–H, 2 × O–H), 1.51–1.44 (2H, m); ^{13}C NMR (126 MHz, $CDCl_3$) δ 72.1, 66.9, 33.5, 32.1, 26.7, 6.8; HRMS (ESI$^+$): found MNa+ 266.9847, $[C_6H_{13}IO_2Na]^+$ requires 266.9852.

7,8-Dihydroxyoctan-1-aminium tetrafluoroborate (10a)

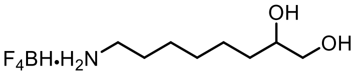

GP2 was followed using oct-7-en-1-aminium tetrafluoroborate **10** (43 mg) in EtOAc (1.2 mL) and 60 h irradiation. The crude residue obtained after filtration over Celite was concentrated under reduced pressure. Et_2O (50 mL) and HCl (10 mL, 1M) were added, and the layers separated. The organic layer was extracted with HCl (1M, 3 × 10 mL). Azeotrope with toluene was used to remove H_2O by concentration under reduced pressure to give 7,8-dihydroxyoctan-1-aminium tetrafluoroborate **10a** as an off-white solid (25 mg, 51%).

^{1}H NMR (400 MHz, D_2O) δ 3.76–3.68 (1H, m), 3.61 (1H, dd, *J* = 11.7, 3.7 Hz), 3.48 (1H, dd, *J* = 11.7, 7.0 Hz), 3.02 (2H, t, *J* = 7.6 Hz), 1.72–1.65 (2H, m), 1.53–1.33 (8H, m), N–H, O–H not observed; ^{13}C NMR (126 MHz, D_2O) δ 72.0, 65.6, 39.7, 32.2, 28.2, 26.7, 25.5, 24.5; HRMS (ESI$^+$): found M+ 162.1491, $[C_8H_{20}NO_2]^+$ requires 162.1489.

N-(7,8-Dihydroxyoctyl)acetamide (11a)

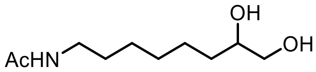

GP2 was followed using *N*-(oct-7-en-1-yl)acetamide **11** (34 mg) in DCE (0.4 mL) with HFIP (84 μL, 0.8 mmol, 4.0 equiv.). *N*-(7,8-Dihydroxyoctyl)acetamide **11a** was isolated by silica gel chromatography (MeOH in CH_2Cl_2, 6%) as a pale yellow solid (29 mg, 72%).

^{1}H NMR (400 MHz, $CDCl_3$) δ 5.55 (1H, br s, N–H), 3.73–3.67 (1H, m), 3.64 (1H, dd, *J* = 11.0, 3.1 Hz), 3.43 (1H, dd, *J* = 11.0, 7.6 Hz), 3.23 (2H, app tdd, *J* = 6.9, 5.7, 1.3 Hz), 2.26 (2H, br s, O–H), 1.97 (3H, s), 1.53–1.47 (2H, m), 1.46–1.30 (8H, m); ^{13}C NMR (101 MHz, $CDCl_3$) δ 170.2, 72.1, 66.9, 39.5, 33.0, 29.4, 29.0, 26.6, 25.3, 23.4; HRMS (ESI$^+$): found MNa+ 226.1403, $[C_{10}H_{22}NO_3Na]^+$ requires 226.1414.

***tert*-Butyl (7,8-dihydroxyoctyl)carbamate (12a)**

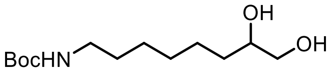

GP2 was followed using *tert*-butyl oct-7-en-1-ylcarbamate **12** (48 μL) in DCE (0.4 mL) with HFIP (84 μL, 0.8 mmol, 4.0 equiv.). *tert*-Butyl (7,8-dihydroxyoctyl)carbamate **12a** was isolated by silica gel chromatography (EtOAc in *n*-hexane, 20%) as a pale yellow solid (37 mg, 71%).

^{1}H NMR (500 MHz, $CDCl_3$) δ 4.50 (1H, br s, N–H), 3.74–3.63 (2H, m), 3.47–3.41 (1H, m), 3.13–3.07 (2H, m), 1.90 (2H, br s, O–H), 1.49–1.42 (13H, m), 1.36–1.30 (6H, m); ^{13}C NMR (126 MHz, $CDCl_3$) δ 156.2, 79.3, 72.3, 67.0, 40.6, 33.2, 30.1, 29.3, 28.6, 26.7, 25.5; HRMS (ESI$^+$): found MNa+ 284.1819, $[C_{13}H_{27}NO_4Na]^+$ requires 284.1832.

***N*-(7,8-Dihydroxyoctyl)-4-methylbenzenesulfonamide (13a)**

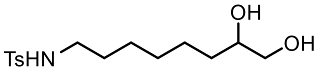

GP2 was followed using 4-methyl-*N*-(oct-7-en-1-yl)benzenesulfonamide (56 mg) **13** in DCE (0.4 mL) with HFIP (84 μL, 0.8 mmol, 4.0 equiv.). *N*-(7,8-Dihydroxyoctyl)-4-methylbenzenesulfonamide **13a** was isolated by silica gel chromatography (MeOH in CH_2Cl_2, 6%) as a yellow oil (39 mg, 62%).

^{1}H NMR (400 MHz, $CDCl_3$) δ 7.73 (2H, app d, *J* = 8.0 Hz), 7.30 (2H, app d, *J* = 7.9 Hz), 4.83 (1H, br s, N–H), 3.71–3.59 (2H, m), 3.41 (1H, dd, *J* = 10.0, 8.0 Hz), 2.90 (2H, t, *J* = 6.4 Hz), 2.54 (2H, br s, O–H), 2.42 (3H, s), 1.48–1.36 (4H, m), 1.31–1.23 (6H, m); ^{13}C NMR (101 MHz, $CDCl_3$) δ 143.5, 137.0, 129.8, 127.2, 72.3, 66.9, 43.2, 33.0, 29.5, 29.0, 26.4, 25.4, 21.6; HRMS (ESI$^+$): found MNa+ 338.1385, $[C_{15}H_{25}NO_4SNa]^+$ requires 338.1397.

2-(7,8-Dihydroxyoctyl)isoindoline-1,3-dione (14a)

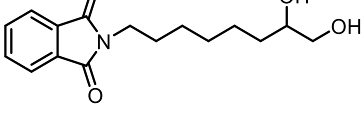

GP2 was followed using 2-(oct-7-en-1-yl)isoindoline-1,3-dione **14** (51 mg) in EtOAc (0.4 mL). 2-(7,8-Dihydroxyoctyl)isoindoline-1,3-dione **14a** was isolated by silica gel chromatography (EtOAc in *n*-hexane, 70%) as an off-white solid (44 mg, 75%).

^{1}H NMR (400 MHz, $CDCl_3$) δ 7.85–7.80 (2H, m), 7.72–7.67 (2H, m), 3.73–3.61 (4H, m), 3.42 (1H, dd, *J* = 10.9, 7.6 Hz), 2.44 (2H, br s, O–H), 1.70–1.62 (2H, m), 1.45–1.30 (8H, m); ^{13}C NMR (126 MHz, $CDCl_3$) δ

168.6, 134.0, 132.2, 123.3, 72.3, 66.9, 38.0, 33.1, 29.1, 28.5, 26.7, 25.4; HRMS (ESI$^+$): found MNa+ 314.1357, $[C_{16}H_{21}NO_4Na]^+$ requires 314.1363.

Octane-1,2,8-triol (15a)

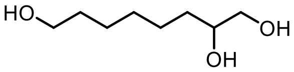

GP2 was followed using oct-7-en-1-ol **15** (31 µL) in EtOAc with HFIP (105 µL, 1.0 mmol, 5.0 equiv.). Octane-1,2,8-triol **15a** was isolated by silica gel chromatography (MeOH in CH_2Cl_2, 6%) as a yellow oil (25 mg, 76%).

^{1}H NMR ($CDCl_3$, 400 MHz) δ 3.75–3.68 (1H, m), 3.67–3.63 (3H, m), 3.44 (1H, dd, *J* = 11.0, 7.6 Hz), 1.76 (3H, br s, O–H), 1.61–1.54 (2H, m), 1.49–1.31 (8H, m); ^{13}C NMR (101 MHz, $CDCl_3$) δ 72.4, 67.0, 63.1, 33.2, 32.8, 29.5, 25.8, 25.6; HRMS (ESI$^+$): found MNa+ 185.1143, $[C_8H_{18}O_3Na]^+$ requires 185.1148.

4-Phenylbutane-1,2,4-triol (16a)

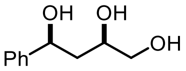

17a'

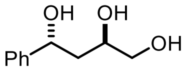

17a''

GP2 was followed using 1-phenylbut-3-en-1-ol **16** (31 µL) in EtOAc (0.4 mL) with HFIP (84 µL, 0.8 mmol, 4.0 equiv.). 4-Phenylbutane-1,2,4-triol **16a’** and **16a’’** were isolated by silica gel chromatography (MeOH in CH_2Cl_2, 6%) (22 mg, 60% total, d.r. 1:1) both as colourless oils.

16a’: ^{1}H NMR ($CDCl_3$, 400 MHz,) δ 7.39–7.27 (5H, m), 4.99 (1H, dd, *J* = 10.0, 2.7 Hz), 4.08–4.01 (1H, m), 3.65 (1H, dd, *J* = 11.1, 2.4 Hz), 3.51 (1H, dd, *J* = 11.1, 6.4 Hz), 1.94 (1H, app dt, *J* = 14.5, 9.8 Hz), 1.77 (1H, app dt, *J* = 14.6, 2.8 Hz), O–H not observed; ^{13}C NMR (101 MHz, $CDCl_3$) δ 144.3, 128.8, 128. 0, 125.8, 74.8, 72.4, 66.9, 41.6.

Data in accordance with literature.[6]

16a’’: ^{1}H NMR (400 MHz, $CDCl_3$) δ 7.37–7.27 (5H, m), 5.04 (1H, dd, *J* = 8.0, 3.5 Hz), 4.03–3.94 (1H, m), 3.67–3.59 (1H, m), 3.56–3.49 (1H, m), 1.94–1.79 (2H, m), O–H not observed; ^{13}C NMR (101 MHz, $CDCl_3$) δ 144.3, 128.7, 127.7, 125.7, 71.7, 69.6, 66.9, 41.1.

Data in accordance with literature.[7]

8-((Triisopropylsilyl)oxy)octane-1,2-diol (17a)

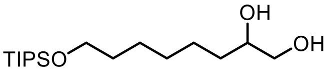

GP2 was followed using triisopropyl(oct-7-en-1-yloxy)silane **17** (57 mg) in DCE (0.4 mL) with HFIP (84 µL, 0.8 mmol, 4.0 equiv.). 8-((Triisopropylsilyl)oxy)octane-1,2-diol **17a** was isolated by silica gel chromatography (EtOAc in *n*-hexane, 40%) as a yellow oil (36 mg, 56%).

^{1}H NMR (400 MHz, $CDCl_3$) δ 3.67–3.57 (4H, m), 3.43 (1H, dd, *J* = 10.9, 7.6 Hz), 1.51–1.44 (2H, m), 1.40–1.24 (8H, m), 1.02–0.95 (21H, m), O–H not observed; ^{13}C NMR (101 MHz, $CDCl_3$) δ 72.4, 67.0, 63.6, 33.3, 33.1, 29.6, 25.9, 25.7, 18.2, 12.2; HRMS (ESI$^+$): found MNa+ 341.2481, $[C_{17}H_{38}O_3SiNa]^+$ requires 341.2482.

8-((*tert*-Butyldimethylsilyl)oxy)octane-1,2-diol (18a)

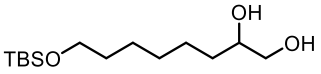

GP2 was followed using *tert*-butyldimethyl(oct-7-en-1-yloxy)silane **18** (48 mg) in EtOAc. 8-((*tert*-Butyldimethylsilyl)oxy)octane-1,2-diol **18a** was isolated by silica gel chromatography (EtOAc in *n*-hexane, 70%) as a yellow oil (43 mg, 78%).

^{1}H NMR (400 MHz, $CDCl_3$) δ 3.79–3.67 (1H, m), 3.66 (1H, dd, *J* = 11.0, 2.9 Hz), 3.60 (2H, t, *J* = 6.5 Hz), 3.44 (1H, dd, *J* = 10.9, 7.6 Hz), 1.55–1.47 (2H, m), 1.47–4.31 (8H, m), 0.89 (9H, s), 0.04 (6H, s), O–H not observed; ^{13}C NMR (101 MHz, $CDCl_3$) δ 72.4, 67.0, 63.4, 33.3, 32.9, 29.6, 26.1, 25.9, 25.7, 18.5, –5.1; HRMS (ESI$^+$): found MNa+ 299.2006, $[C_{14}H_{32}O_3SiNa]^+$ requires 299.2013.

7,8-Dihydroxyoctyl Acetate (19a)

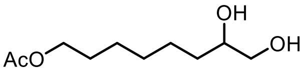

GP2 was followed using oct-7-en-1-yl acetate **19** (34 mg) in EtOAc. 7,8-Dihydroxyloctyl acetate **19a** was isolated by silica gel chromatography (MeOH in CH_2Cl_2, 6%) as a yellow oil (33 mg, 81%).

^{1}H NMR (400 MHz, $CDCl_3$) δ 4.04 (2H, t, *J* = 6.7 Hz), 3.72–3.62 (2H, m), 3.43 (1H, dd, *J* = 10.3, 7.7 Hz), 2.29 (2H, br s, O–H), 2.04 (3H, s), 1.65–1.58 (2H, m), 1.49–1.31 (8H, m); ^{13}C NMR (101 MHz, $CDCl_3$) δ 171.5, 72.3, 67.0, 64.7, 33.2, 29.3, 28.6, 25.9, 25.5, 21.2; HRMS (ESI$^+$): found MNa+ 227.1248, $[C_{10}H_{20}O_4Na]^+$ requires 227.1254.

7-(Oxiran-2-yl)octane-1,2-diol (20a)

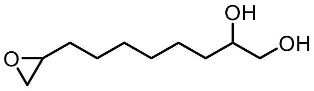

GP2 was followed using 2-(oct-7-en-1-yl)oxirane **20** (36 µL) in EtOAc (0.4 mL). 7-(Oxiran-2-yl)octane-1,2-diol **20a** was isolated by silica gel chromatography (MeOH in CH_2Cl_2, 6%) as a yellow oil (29 mg, 76%).

^{1}H NMR (500 MHz, $CDCl_3$) δ 3.74–3.66 (1H, m), 3.65 (1H, dd, *J* = 11.0, 3.0 Hz), 3.43 (1H, dd, *J* = 11.0, 7.6 Hz), 2.92–2.88 (1H, m), 2.74 (1H, dd, *J* = 5.1, 3.9 Hz), 2.46 (1H, dd, *J* = 5.0, 2.7 Hz), 2.15 (2H, br s, O–H), 1.57–1.49 (2H, m), 1.48–1.41 (5H, m), 1.38–1.30 (5H, m); ^{13}C NMR (101 MHz, $CDCl_3$) δ 72.4, 67.0, 52.6, 47.3, 33.2, 32.6, 29.6, 29.4, 26.0, 25.6.
Data in accordance with literature.[8]

Diethyl (7,8-Dihydroxyoctyl)phosphonate (21a)

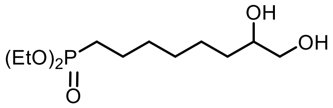

GP2 was followed using diethyl oct-7-en-1-ylphosphonate **21** (47 mg) in EtOAc. Diethyl (7,8-dihydroxyoctyl)phosphonate **21a** was isolated by silica gel chromatography (MeOH in CH_2Cl_2, 6%) as a yellow oil (43 mg, 77%).

^{1}H NMR (400 MHz, $CDCl_3$) δ 4.14–4.00 (4H, m), 3.72–3.65 (1H, m), 3.62 (1H, dd, *J* = 11.0, 2.9 Hz), 3.42 (1H, dd, *J* = 11.0, 7.5 Hz), 2.71 (2H, br s, O–H), 1.75–1.66 (2H, m), 1.64–1.53 (2H, m), 1.48–1.33 (8H, m), 1.31 (6H, t, *J* = 7.1 Hz); ^{13}C NMR (101 MHz, $CDCl_3$) δ 72.3, 67.0, 61.7 (d, *J* = 6.5 Hz), 33.2, 30.5 (d, *J* = 16.5 Hz), 29.1, 25.7 (d, *J* = 140.4 Hz), 25.4, 22.3 (d, *J* = 5.2 Hz), 16.6 (d, *J* = 6.0 Hz); ^{31}P NMR (162 MHz,

$CDCl_3$) δ 32.9–32.4 (m); ^{31}P {^{1}H} NMR (162 MHz, $CDCl_3$) δ 32.7; HRMS (ESI$^+$): found MNa+ 305.1483, $[C_{12}H_{27}O_5PNa]^+$ requires 305.1488.

8-(4,4,5,5-Tetramethyl-1,3,2-dioxaborolan-2-yl)octane-1,2-diol (22a)

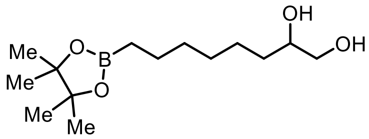

GP2 was followed using 4,4,5,5-tetramethyl-2-(oct-7-en-1-yl)-1,3,2-dioxaborolane **22** (47 mg) in EtOAc. 8-(4,4,5,5-Tetramethyl-1,3,2-dioxaborolan-2-yl)octane-1,2-diol **22a** was isolated by silica gel chromatography (EtOAc, 100%) as a colourless oil (37 mg, 68%).

^{1}H NMR (400 MHz, $CDCl_3$) δ 3.75–3.67 (1H, m), 3.65 (1H, dd, J = 11.0, 3.0 Hz), 3.43 (1H, dd, J = 10.9, 7.6 Hz), 1.93 (2H, br s, O–H), 1.46–1.28 (10H, m), 1.24 (12H, s), 0.76 (2H, t, J = 7.7 Hz); ^{13}C NMR (101 MHz, CD_3OD) δ 84.3, 73.3 (d, J = 1.0 Hz), 67.4, 34.5 (d, J = 2.0 Hz), 33.5 (d, J = 25.0 Hz), 30.7 (d, J = 9.1 Hz), 26.7 (d, J = 3.2 Hz), 25.1 (2C), 24.9 (d, J = 4.6 Hz); HRMS (ESI$^+$): found MNa+ 295.2048, $[C_{14}H_{29}BO_4Na]^+$ requires 295.2051.

8-(Methylthio)octane-1,2-diol (23a)

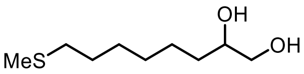

GP1 was followed using methyl(oct-7-en-1-yl)sulfane **23** (37 μL) in CH_2Cl_2 (1.2 mL) with PFTB (112 μL, 0.8 mmol, 4.0 equiv.). 8-(Methylthio)octane-1,2-diol **23a** was isolated by silica gel chromatography as a yellow oil (10 mg, 25%).

^{1}H NMR (400 MHz, $CDCl_3$) δ 3.74–3.62 (2H, m), 3.47–3.41 (1H, m), 2.48 (2H, t, J = 7.4 Hz), 2.09 (3H, s), 1.59 (2H, app p, J = 7.1 Hz), 1.47–1.31 (8H, m), O–H not observed; ^{13}C NMR (101 MHz, $CDCl_3$) δ 72.3, 66.9, 34.3, 33.1, 29.2, 29.1, 28.7, 25.4, 15.6; HRMS (ESI$^+$): found MNa+ 215.1068, $[C_9H_{20}O_2SNa]^+$ requires 215.1076.

8-Nitrooctane-1,2-diol (24a)

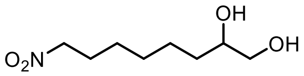

GP1 was followed using 8-nitrooct-1-ene **24** (33 μL) in EtOAc. 8-Nitrooctane-1,2-diol **24a** was isolated by silica gel chromatography (EtOAc, 100%) as a colourless oil (7 mg, 19%).

^{1}H NMR (400 MHz, $CDCl_3$) δ 4.38 (2H, t, J = 7.0 Hz), 3.74–3.62 (2H, m), 3.44 (1H, dd, J = 10.4, 7.9 Hz), 2.07–1.97 (3H, m, 2 × C–H, 1 × O–H), 1.84 (1H, br s, O–H), 1.51–1.34 (8H, m); ^{13}C NMR (101 MHz, $CDCl_3$) δ 75.8, 72.2, 67.0, 33.0, 29.0, 27.4, 26.3, 25.4; HRMS (ESI$^+$): found MNa+ 214.1048, $[C_8H_{17}NO_4Na]^+$ requires 214.1050.

3,7,11,15-Tetramethylhexadecane-1,2,3-triol (25a)

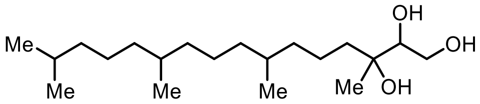

GP2 was followed using isophytol **25** (37 μL, 95% purity, 0.1 mmol, 1.0 equiv.) and 2-fluoro-4-nitro-1-((trifluoromethyl)sulfonyl)benzene **N2** (55 mg, 0.2 mmol, 2.0 equiv.) in EtOAc (0.4 mL). Then $KHCO_2$ (168 mg, 2.0 mmol, 20 equiv.) and HCO_2H (46 μL, 1.2 mmol, 12 equiv.) at –20 °C. 3,7,11,15-Tetramethylhexadecane-1,2,3-triol **25a** was isolated by silica gel chromatography (EtOAc in *n*-hexane, 60%) as a colourless oil (24 mg, 72%, d.r. 1.5:1).

Mixture of diastereomers (major, **M** + minor, **m**) ^1H NMR (400 MHz, $CDCl_3$) δ 3.77 (2H, app t, *J* = 4.8 Hz), 3.53–3.47 (1H, app dtd, *J* = 13.3, 4.9, 1.5 Hz), 1.57–1.04 (24H, m), 0.88–0.83 (12H, m), O–H not observed; ^{13}C NMR (101 MHz, $CDCl_3$) δ 76.3 (**M**), 75.3 (**m**), 74.9 (**M**), 74.7 (**m**), 63.6 (**m**), 63.4 (**M**), 39.9, 39.5, 38.6, 37.9, 37.8, 37.8, 37.7, 37.6, 37.5, 37.5, 37.4, 32.9, 28.1, 25.0, 24.9, 24.6, 23.8, 23.8, 22.9, 22.8, 22.5, 22.5, 21.2, 21.1, 19.9, 19.8, 19.8, 19.7. d.r. evaluated by ^{13}C NMR **C**H_2OH signals at 63.6 ppm (**m**) and 63.4 ppm (**M**).

Data in accordance with literature.[9]

1-Phenylethane-1,2-diol (26a)

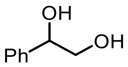

GP2 was followed using styrene **26** (24 μL, 95% purity) in EtOAc (0.4 mL) and 12 h irradiation. Then $KHCO_2$ (336 mg, 4 mmol, 20 equiv.) and HCO_2H (91 µL, 2.4 mmol, 12 equiv.) at –20 °C. 1-Phenylethane-1,2-diol **26a** was isolated by silica gel chromatography (EtOAc in *n*-hexane, 50%) as an off-white solid (17 mg, 62%).

^1H NMR (400 MHz, $CDCl_3$) δ 7.39–7.28 (5H, m), 4.84 (1H, dd, *J* = 8.1, 3.4 Hz), 3.81–3.75 (1H, m), 3.68 (1H, dd, *J* = 11.1, 8.3 Hz), 2.49 (1H, br s, O–H), 2.03 (1H, br s, O–H); ^{13}C NMR (101 MHz, $CDCl_3$) δ 140.6, 128.7, 128.2, 126.2, 74.8, 68.3.
Data in accordance with literature.[10]

1-(4-Methoxyphenyl)ethane-1,2-diol (27a)

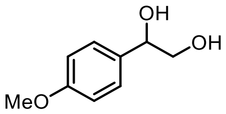

GP2 was followed using 1-methoxy-4-vinylbenzene **27** (28 μL, 96% purity) in EtOAc and 12 h irradiation. Then $KHCO_2$ (336 mg, 4 mmol, 20 equiv.) and HCO_2H (91 µL, 2.4 mmol, 12 equiv.) at –20 °C. 1-(4-Methoxyphenyl)ethane-1,2-diol **27a** was isolated by silica gel chromatography (MeOH in CH_2Cl_2, 6%) as a light yellow oil (17 mg, 51%).

^1H NMR (400 MHz, $CDCl_3$) δ 7.31–7.28 (2H, m), 6.92–6.88 (2H, m), 4.78 (1H, dd, *J* = 8.1, 3.7 Hz), 3.81 (3H, s), 3.73 (1H, dd, *J* = 11.2, 3.8 Hz), 3.66 (1H, dd, *J* = 11.2, 8.1 Hz), O–H not observed; ^{13}C NMR (101 MHz, $CDCl_3$) δ 159.5, 132.6, 127.4, 114.0, 74.3, 68.1, 55.3.
Data in accordance with literature.[10]

1-(4-(Trifluoromethyl)phenyl)ethane-1,2-diol (28a)

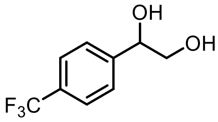

GP2 was followed using 1-(trifluoromethyl)-4-vinylbenzene **28** (31 µL, 96% purity) in EtOAc and 12 h irradiation. Then $KHCO_2$ (336 mg, 4 mmol, 20 equiv.) and HCO_2H (91 µL, 2.4 mmol, 12 equiv.) at –20 °C. 1-(4-(Trifluoromethyl)phenyl)ethane-1,2-diol **28a** was isolated by silica gel chromatography (MeOH in CH_2Cl_2, 6%) as a white solid (21 mg, 52%).

^{1}H NMR (400 MHz, CD_3CN) δ 7.68–7.64 (2H, m), 7.57–7.54 (2H, m), 4.75 (1H, dd, *J* = 7.3, 4.3 Hz), 3.68–3.59 (2H, m, 1 × C–H, 1 × O–H), 3.50 (1H, dd, *J* = 11.2, 7.2 Hz), 2.99 (1H, br s, O–H); ^{13}C NMR (101 MHz, $CDCl_3$) δ 148.0 (q, *J* = 1.2 Hz), 129.7 (q, *J* = 31.9 Hz), 127.9, 125.9 (q, *J* = 3.9 Hz), 125.6 (q, *J* = 271 Hz), 74.5, 68.2, ^{19}F NMR (471 MHz, CD_3CN) –62.9.

Data in accordance with literature.[10]

2-Phenylpropane-1,2-diol (29a)

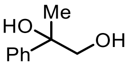

GP2 was followed using prop-1-en-2-ylbenzene **29** (27 µL, 95% purity) in EtOAc (0.4 mL) and 12 h irradiation. Then $KHCO_2$ (336 mg, 4 mmol, 20 equiv.) and HCO_2H (91 µL, 2.4 mmol, 12 equiv.) at –20 °C. 2-Phenylpropane-1,2-diol **29a** was isolated by silica gel chromatography (EtOAc in *n*-hexane, 50%) as an off-white solid (15 mg, 50%).

^{1}H NMR (400 MHz, $CDCl_3$) δ 7.48–7.45 (2H, m), 7.40–7.35 (2H, m), 7.30–7.27 (1H, m), 3.81 (1H, d, *J* = 11.1 Hz), 3.64 (1H, d, *J* = 10.7 Hz), 2.56 (1H, br s, O–H), 1.77 (1H, br s, O–H), 1.54 (3H, s); ^{13}C NMR (101 MHz, $CDCl_3$) 145.1, 128.6, 127.4, 125.2, 75.0, 71.3, 26.2.

Data in accordance with literature.[10]

1-Phenylpropane-1,2-diol (30a)

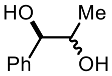

GP2 was followed using (*E*)-prop-1-en-2-ylbenzene **30** (26 µL) in EtOAc and 6 h irradiation. Then $KHCO_2$ (336 mg, 4.0 mmol, 20 equiv.) at –20 °C. Following 3 cycles of vacuum/nitrogen, the vial was placed under vacuum, disconnected from the line as quickly as possible, and a balloon of H_2 was added via a needle through the septum. Then HCO_2H (91 µL, 2.4 mmol, 12 equiv.) was added. 1-Phenylpropane-1,2-diol **30a** was isolated by silica gel chromatography (EtOAc in *n*-hexane, 50%) as a colourless oil (9 mg, 29%, *syn*:*anti* 4.5:1). Analysis of crude ^{1}H NMR spectrum shows *syn*:*anti* 8.7:1, thus the distribution isolated is likely not reflective of true d.r. in this case.

^{1}H NMR (400 MHz, $CDCl_3$) δ 7.39–7.29 (5H, m, *syn*+*anti*), 4.69 (1H, app dd, *J* = 4.2, 3.3 Hz, *anti*), 4.39 (1H, app dd, *J* = 7.3, 2.8 Hz, *syn*), 4.07–4.40 (1H, m, *anti*), 3.88 (1H, app ddq, *J* = 10.3, 6.2, 3.2 Hz, *syn*), 2.55 (1H, d, *J* = 3.2 Hz, *syn*, O–H), 2.41 (1H, d, *J* = 3.2 Hz, *syn*, O–H), 2.30 (1H, d, *J* = 3.3 Hz, *anti*, O–H), 1.82 (1H, d, *J* = 4.9 Hz, *anti*, O–H), 1.10 (3H, d, *J* = 6.4 Hz, *anti*), 1.08 (3H, d, *J* = 6.3 Hz, *syn*); ^{13}C NMR (126 MHz, CD_3CN) δ 143.3 (*syn*), 143.1 (*anti*), 129.0 (*syn*), 128.8 (*anti*), 128.4 (*syn*), 128.0 (*anti*), 127.9 (*syn*), 127.7 (*anti*), 79.5 (*syn*), 78.1 (*anti*), 72.5 (*syn*), 71.8 (*anti*), 19.3 (*syn*), 18.0 (*anti*).

Data in accordance with literature.[11]

Following **GP2** using (*E*)-prop-1-en-2-ylbenzene **30** (26 μL) in EtOAc and 6 h irradiation, then $KHCO_2$ (336 mg, 4.0 mmol, 20 equiv.) and HCO_2H (91 μL, 2.4 mmol, 12 equiv.) at –20 °C gave **30a** (25%, *syn*:*anti* 8.3:1; yield evaluated by quantitative ^{1}H NMR).

Decane-5,6-diol (31a)

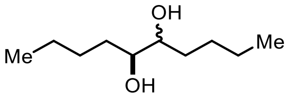

GP2 was followed using (*E*)-dec-5-ene (38 μL) **31** and 1-nitro-3,5-bis(trifluoromethyl)benzene **N3** (68 µL, added at the same stage as **31**) in EtOAc. Decane-5,6-diol **31a** was isolated by silica gel chromatography (EtOAc in CH_2Cl_2, 20%) as an off-white solid (26 mg, 75%, *syn*:*anti* 3.4:1).

^{1}H NMR (400 MHz, $CDCl_3$) δ 3.60 (2H, app p, *J* = 3.5 Hz, *anti*), 3.43–3.38 (2H, m, *syn*), 2.06 (2H, br s, *syn+anti*), 1.56–1.28 (12H, m, *syn+anti*), 0.91 (6H, t, *J* = 7.0 Hz, *syn+anti*); ^{13}C NMR (126 MHz, $CDCl_3$) δ 74.8 (*anti*), 74.7 (*syn*), 33.5 (*syn*), 31.0 (*anti*), 28.4 (*anti*), 28.0 (*syn*), 22.9 (*syn+anti*), 14.2 (*syn+anti*). Data in accordance with literature.[12,13]

1-(3-Phenylpropoxy)hexane-3,4-diol (32a)

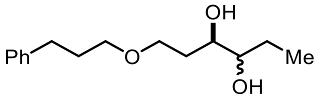

GP2 was followed using (*E*)-(3-(hex-3-en-1-yloxy)propyl)benzene **32** (44 mg) in DCE (0.8 mL) with HFIP (84 μL, 0.8 mmol, 4.0 equiv). 1-(3-Phenylpropoxy)hexane-3,4-diol **32a** was isolated by silica gel chromatography (EtOAc in CH_2Cl_2, 20%) as a yellow oil (30 mg, 60%, *syn*:*anti* 4.0:1).

^{1}H NMR (500 MHz, $CDCl_3$) δ 7.30–7.27 (2H+2H, m, *syn+anti*), 7.21–7.17 (3H+3H, m, *syn+anti*), 3.77 (1H, ddd, *J* = 8.9, 4.0, 2.6 Hz, *anti*), 3.71–3.61 (3H+2H, m, *syn+anti*), 3.54 (1H, app dt, *J* = 8.5, 4.3 Hz, *anti*), 3.49–3.44 (2H+2H, m, *syn+anti*), 3.37 (1H, app dt, *J* = 8.6, 4.5 Hz, *syn*), 2.69–2.66 (2H+2H, m, *syn+anti*), 1.94–1.83 (3H+3H, m, *syn+anti*), 1.78–1.68 (1H+1H, m, *syn+anti*), 1.61–1.44 (2H+2H, m, *syn+anti*), 1.00 (3H, t, *J* = 7.4 Hz, *anti*), 1.00 (3H, t, *J* = 7.5 Hz, *syn*), O–H not observed; ^{13}C NMR (126 MHz, $CDCl_3$) δ 141.8 (*syn*), 141.7 (*anti*), 128.6 (*syn+anti*), 128.5 (*syn+anti*), 126.0 (*syn+anti*), 75.8 (*syn*), 75.7 (*anti*), 74.5 (*anti*), 73.7 (*syn*), 70.7 (*syn+anti*), 69.9 (*anti*), 69.4 (*syn*), 33.3 (*syn*), 32.4 (*syn+anti*), 31.3 (*syn+anti*), 30.2 (*anti*), 26.5 (*syn*), 25.3 (*anti*), 10.5 (*anti*), 10.3 (*syn*); HRMS (ESI$^+$): found MNa+ 275.1611, $[C_{15}H_{24}O_3Na]^+$ requires 275.1618.

Methyl 9,10-Dihydroxyoctadecanoate (33a)

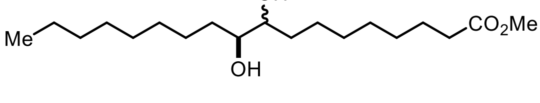

GP2 was followed using methyl oleate **33** (34 μL, 99% purity, 0.1 mmol, 1.0 equiv.) and 1-nitro-3,5-bis(trifluoromethyl)benzene **N3** (34 µL, added at the same stage as **33**) in EtOAc (0.4 mL) and 16 h irradiation. Then $KHCO_2$ (168 mg, 2 mmol, 20 equiv.) and HCO_2H (45 µL, 1.2 mmol, 12 equiv.) at –20 °C. Methyl 9,10-dihydroxyoctadecanoate **33a** was isolated by silica gel chromatography (EtOAc in CH_2Cl_2, 10%) as an off-white solid (22 mg, 66%, *syn:anti* 2.0:1).

^{1}H NMR (500 MHz, $CDCl_3$) δ 3.66 (3H+3H, s, *syn+anti*), 3.60–3.56 (2H, m, *anti*), 3.41–3.36 (2H, m, *syn*), 2.29 (2H+2H, t, *J* = 7.5 Hz, *syn+anti*), 2.09 (2H+2H, br s, *syn+anti*, O–H), 1.61 (2H+2H, app p, *J* = 7.4 Hz, *syn+anti*), 1.52–1.39 (6H+6H, m, *syn+anti*), 1.35–1.26 (18H+18H, m, *syn+anti*), 0.87 (3H+3H, t, *J* = 6.8 Hz, *syn+anti*); ^{13}C NMR (126 MHz, $CDCl_3$) δ 174.5 (*syn+anti*), 74.8 (*anti*), 74.8 (*anti*), 74.7 (*syn*), 74.6

(*syn*), 51.6 (*syn+anti*), 34.2 (*syn+anti*), 33.8 (*syn*), 33.7 (*syn*), 32.0 (*syn+anti*), 31.4 (*anti*), 31.3 (*anti*), 29.8 (*syn+anti*), 29.7 (*syn+anti*), 29.6 (*syn+anti*), 29.4 (*syn+anti*), 29.3 (*syn+anti*), 29.2 (*syn+anti*), 26.2 (*anti*), 26.1 (*anti*), 25.8 (*syn*), 25.7 (*syn*), 25.0 (*syn+anti*), 22.8 (*syn+anti*), 14.2 (*syn+anti*).

Data in accordance with literature.[14]

9,10-Dihydroxyoctadecanamide (34a)

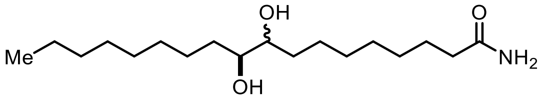

GP2 was followed using oleamide **34** (56 mg) in DCE (0.8 mL) with HFIP (84 µL, 0.8 mmol, 4.0 equiv). Then $KHCO_2$ (336 mg, 4.0 mmol, 20 equiv.) at –20 °C. Following 3 cycles of vacuum/nitrogen, the vial was placed under vacuum, disconnected from the line as quickly as possible, and a balloon of H_2 was added via a needle through the septum. Then HCO_2H (91 µL, 2.4 mmol, 12 equiv.). 9,10-Dihydroxyoctadecanamide **34a** was isolated by silica gel chromatography (MeOH in CH_2Cl_2, 6%) as an off-white solid (24 mg, 38%, *syn:anti* 3.8:1).

^{1}H NMR (400 MHz, $CDCl_3$) δ 5.37 (2H+2H, br s, *syn+anti,* N–H), 3.62–3.56 (2H, m, *anti*), 3.42–3.37 (2H, m, *syn*), 2.22 (2H+2H, t, *J* = 7.6 Hz, *syn+anti*), 2.06 (2H+2H, br s, *syn+anti*, O–H), 1.67–1.27 (26H+26H, m, *syn+anti*), 0.88 (3H+3H, t, *J* = 6.8 Hz, *syn+anti*); ^{13}C NMR (101 MHz, $CDCl_3$) δ 175.6 (*syn+anti*), 74.9 (*anti*), 74.8 (*anti*), 74.7 (*syn*), 74.6 (*syn*), 36.0 (*syn+anti*), 33.8 (*syn+anti*), 33.7 (*syn+anti*), 32.0 (*syn+anti*), 29.8 (*syn+anti*), 29.7 (*syn+anti*), 29.5 (*syn+anti*), 29.4 (*syn+anti*), 29.3 (*syn+anti*), 29.2 (*syn+anti*), 25.8 (*syn+anti*), 25.6 (*syn+anti*), 25.5 (*syn+anti*), 22.8 (*syn+anti*), 14.3 (*syn+anti*); HRMS (ESI$^+$): found MNa+ 338.2657, $[C_{18}H_{37}NO_3Na]^+$ requires 338.2666.

Following **GP2** using oleamide **34** (56 mg) in DCE (0.8 mL) with HFIP (84 µL, 0.8 mmol, 4.0 equiv). Then $KHCO_2$ (336 mg, 4.0 mmol, 20 equiv.) and HCO_2H (91 µL, 2.4 mmol, 12 equiv.) at –20 °C gave **34a** (31%, *syn:anti* 1.9:1; yield evaluated by quantitative ^{1}H NMR).

9,10-Dihydroxy-*N*-methyloctadecanamide (35a)

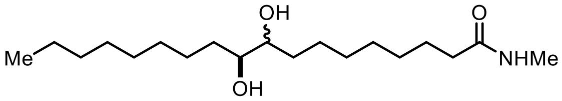

GP2 was followed using *N*-methyloleamide **35** (59 mg) in DCE (0.8 mL) with HFIP (84 µL, 0.8 mmol, 4.0 equiv). Then $KHCO_2$ (336 mg, 4 mmol, 20 equiv.) and HCO_2H (91 µL, 2.4 mmol, 12 equiv.) at –20 °C. 9,10-Dihydroxy-*N*-methyloctadecanamide **35a** was isolated by silica gel chromatography (MeOH in CH_2Cl_2, 6%) as an off-white solid (28 mg, 43%, *syn:anti* 4.9:1). Analysis of crude ^{1}H NMR spectrum shows *syn*:*anti* 2.3:1, thus the distribution isolated is likely not reflective of true d.r. in this case.

^{1}H NMR (500 MHz, $CDCl_3$) δ 5.47 (1H, br s, *syn+anti*, N–H), 3.62–3.57 (2H, m, *anti*), 3.42–3.37 (2H, m, *syn*), 2.81 (3H+3H, d, *J* = 4.8 Hz, *syn+anti*), 2.16 (2H+2H, t, *J* = 7.6 Hz, *syn+anti*), 1.62 (2H+2H, app p, *J* = 7.2 Hz, *syn+anti*), 1.52–1.27 (24H+24H, *syn+anti)*, 0.88 (3H+3H, t, *J* = 7.3 Hz, *syn+anti*), O–H not observed; ^{13}C NMR (101 MHz, $CDCl_3$) δ 173.9 (*syn+anti*), 74.9 (*anti*), 74.8 (*anti*), 74.7 (*syn*), 74.6 (*syn*), 36.8 (*syn+anti*), 33.8 (*syn*), 33.7 (*syn*), 32.1 (*anti*), 32.0 (*syn*), 31.4 (*anti*), 31.3 (*anti*), 29.8 (*syn+anti*) 29.7 (*syn+anti*), 29.5 (*syn+anti*), 29.4 (*syn+anti*), 29.3 (*syn+anti*), 29.2 (*syn+anti*), 26.4 (*syn*), 26.2 (*anti*), 26.0 (*anti*), 25.8 (*syn+anti*), 25.8 (*syn+anti*), 25.6 (*syn*), 22.8 (*syn+anti*), 14.3 (*syn+anti*); HRMS (ESI$^+$): found MNa+ 352.2808, $[C_{19}H_{39}NO_3Na]^+$ requires 352.2822.

9,10-Dihydroxy-1-(piperidin-1-yl)octadecan-1-one (36a)

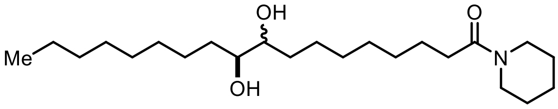

GP2 was followed using (*Z*)-1-(piperidin-1-yl)octadec-9-en-1-one **36** (70 mg) in DCE (0.8 mL) with HFIP (84 µL, 0.8 mmol, 4.0 equiv). 9,10-Dihydroxy-1-(piperidin-1-yl)octadecan-1-one **36a** was isolated by silica gel chromatography (MeOH in CH_2Cl_2, 6%) as an off-white solid (48 mg, 62%, *syn:anti* 10:1). d.r. determined by ^{13}C NMR using **C**HOH signals at approximately 75 ppm.

^{1}H NMR (400 MHz, $CDCl_3$) δ 3.62–3.36 (6H+6H, m, *syn+anti*), 2.32–2.28 (2H+2H, m, *syn+anti*), 1.67–1.26 (32H+32H, m, *syn+anti*), 0.91–0.84 (3H+3H, m, *syn+anti*), O–H not observed; ^{13}C NMR (101 MHz, $CDCl_3$) δ 171.6 (*syn+anti*), 74.9 (*anti*), 74.8 (*anti*), 74.7 (*syn*), 74.6 (*syn*), 46.9 (*syn+anti*), 42.8 (*syn+anti*), 33.8 (*syn+anti*), 33.7 (*syn+anti*), 33.5 (*syn+anti*), 32.0 (*syn+anti*), 29.7 (*syn+anti*), 29.5 (*syn+anti*), 29.4 (*syn+anti*), 29.4 (*syn+anti*), 29.4 (*syn+anti*), 26.7 (*syn+anti*), 25.8 (*syn+anti*), 25.7 (*syn+anti*), 25.6 (*syn+anti*), 25.5 (*syn+anti*), 24.7 (*syn+anti*), 22.8 (*syn+anti*), 14.3 (*syn+anti*); HRMS (ESI$^+$): found MNa+ 406.3287, $[C_{23}H_{45}NO_3Na]^+$ requires 406.3292. Additional ^{13}C NMR signals due to amide rotamers.[15]

Cyclooctane-1,2-diol (37a)

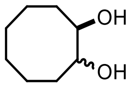

GP2 was followed using (*Z*)-cyclooctene **37** (14 µL, 95% purity) and 1-nitro-3,5-bis(trifluoromethyl)benzene **N3** (34 µL, added at the same stage as **37**) in EtOAc (0.4 mL) and 16 h irradiation. Then $KHCO_2$ (168 mg, 2 mmol, 20 equiv.) and HCO_2H (45 µL, 1.2 mmol, 12 equiv.) at –20 °C. Cyclooctane-1,2-diol **37a** was isolated by silica gel chromatography (EtOAc in CH_2Cl_2, 40%) as a white solid (14 mg, 95%, *cis*:*trans* 4.0:1).

^{1}H NMR (500 MHz, $CDCl_3$, *cis+trans*) δ 3.92–3.86 (2H, m, *cis*), 3.61–3.55 (2H, m, *trans*), 2.51 (2H, br s, *trans*, O–H), 2.33 (2H, br s, *cis*, O–H), 1.94–1.82 (2H+2H, m, *cis+trans*), 1.73–1.62 (4H+4H, m, *cis+trans*), 1.57–1.43 (6H+6H, m, *cis+trans*); ^{13}C NMR (126 MHz, $CDCl_3$) δ 76.4 (*trans*), 73.3 (*cis*), 32.1 (*trans*), 30.2 (*cis*), 26.4 (*cis+trans*), 23.9 (*cis*), 23.8 (*trans*).
Data in accordance with literature.[18]

Cyclohexane-1,2-diol (38a)

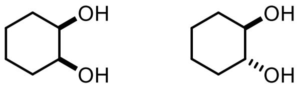

39a' **39a"**

GP2 was followed using cyclohexene **38** (21 µL, 99% purity) in EtOAc and 12 h irradiation. Then $KHCO_2$ (336 mg, 4.0 mmol, 20 equiv.) at –20 °C. Following 3 cycles of vacuum/nitrogen, the vial was placed under vacuum, disconnected from the line as quickly as possible, and a balloon of H_2 was added via a needle through the septum. Then, HCO_2H (91 µL, 2.4 mmol, 12 equiv.). *cis*-Cyclohexane-1,2-diol **38a'** and *trans*-cyclohexane-1,2-diol **38a''** were isolated by silica gel chromatography (EtOAc in CH_2Cl_2, 40%), each as a white solid (16 mg, 69%, *cis*:*trans* 16:1).

38a': ^{1}H NMR (500 MHz, $CDCl_3$) δ 3.80–3.76 (2H, m), 1.96 (2H, br s, O–H), 1.80–1.73 (2H, m), 1.65– 1.52 (4H, m), 1.35–1.28 (2H, m); ^{13}C NMR (126 MHz, $CDCl_3$) δ 70.8, 30.1, 21.6.

38a'': ^{1}H NMR (500 MHz, $CDCl_3$) δ 3.38–3.33 (2H, m), 2.31 (2H, br s, O–H), 2.00–1.95 (2H, m), 1.73–1.67 (2H, m), 1.30–1.22 (4H, m); ^{13}C NMR (126 MHz, $CDCl_3$) δ 76.0, 33.0, 24.5.

Data in accordance with literature.[16]

Following **GP2** using cyclohexene **38** (21 µL, 99% purity) in EtOAc and 12 h irradiation, then $KHCO_2$ (336 mg, 4.0 mmol, 20 equiv.) and HCO_2H (91 µL, 2.4 mmol, 12 equiv.) at –20 °C gave **38a** (62%, *cis*:*trans* 15:1, yield and d.r. evaluated by quantitative ^{1}H NMR).

2-(2,3-Dihydroxycyclohexyl)isoindoline-1,3-dione (39a)

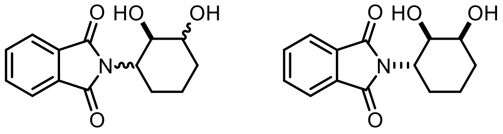

40a **40a'**

GP2 was followed using 2-(2,3-dihydroxycyclohexyl)isoindoline-1,3-dione **39** (23 mg) in EtOAc (0.5 mL). Then $KHCO_2$ (84 mg, 1 mmol, 10 equiv.) and HCO_2H (23 µL, 0.6 mmol, 6 equiv.). 2-(2,3-Dihydroxycyclohexyl)isoindoline-1,3-dione **39a** was isolated as a mixture of diastereomers by silica gel chromatography (EtOAc in *n*-hexane, 40%) as an off-white solid (13 mg, 48%, d.r. >20:1). Further purification via preparative thin layer chromatography (EtOAc in *n*-hexane, 40%) provided a few mg of major diastereomer **40a'** for characterisation.

39a: Mixture of diastereomers (major, **M** + minor, **m**) ^{1}H NMR (500 MHz, $CDCl_3$) δ 7.87 (2H, app dd, *J* = 5.4, 3.1 Hz, **m**), 7.83 (2H, app dd, *J* = 5.3, 3.1 Hz, **M**), 7.76 (2H, app dd, *J* = 5.4, 3.1 Hz, **m**), 7.70 (2H, app dd, *J* = 5.4, 3.0 Hz, **M**), 4.43 (1H, app td, *J* = 10.8, 3.9 Hz, **M**+**m**), 4.36 (1H, dd, *J* = 10.6, 2.9 Hz, **M**+**m**), 4.21 (1H, br s, **M**), 4.15 (1H, br s, **m**), 2.13 (1H, app qd, *J* = 13.2, 3.1 Hz, **M**+**m**), 2.01–1.96 (1H, m, **M**+**m**), 1.86–1.75 (2H, m, **M**+**m**), 1.66–1.58 (2H, m, **M**+**m**), O–H not observed; ^{13}C NMR (126 MHz, $CDCl_3$) δ 169.1 (**M**+**m**), 134.6 (**m**), 134.1 (**M**), 132.1 (**M**+**m**), 123.8 (**m**), 123.3 (**M**), 70.8 (**M**+**m**), 70.4 (**M**+**m**), 51.8 (**M**+**m**), 31.0 (**M**+**m**), 28.6 (**M**+**m**), 19.0 (**M**+**m**).

39a': ^{1}H NMR (500 MHz, $CDCl_3$) δ 7.83 (2H, app dd, *J* = 5.2, 3.1 Hz), 7.70 (2H, app dd, *J* = 5.2, 3.0 Hz), 4.47–4.39 (1H, app td, *J* = 10.9, 3.7 Hz), 4.36 (1H, dd, *J* = 10.7, 2.4 Hz), 4.21 (1H, br s), 2.19 (1H, app qd, *J* = 13.0, 2.9 Hz), 2.01–1.96 (1H, m), 1.86–1.76 (2H, m), 1.66–1.58 (2H, m), O–H not observed; ^{13}C NMR (126 MHz, $CDCl_3$) δ 169.1, 134.1, 132.1, 123.3, 70.8, 70.4, 51.8, 31.0, 28.6, 19.0; HRMS (ESI$^+$): found MNa+ 284.0888, $[C_{14}H_{15}NO_4Na]^+$ requires 284.0893.

2-Butylhexane-1,2-diol (40a)

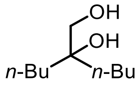

GP2 was followed using 5-methylenenonane **40** (38 µL, 94% purity) in EtOAc. Then $KHCO_2$ (336 mg, 4 mmol, 20 equiv.) and HCO_2H (91 µL, 2.4 mmol, 12 equiv.) at –20 °C. 2-Butylhexane-1,2-diol **40a** was isolated by silica gel chromatography (EtOAc in *n*-hexane, 30%) as a yellow oil (26 mg, 75%).

^{1}H NMR (400 MHz, $CDCl_3$) δ 3.45 (2H, s), 2.05 (1H, br s, O–H), 1.84 (1H, br s, O–H), 1.52–1.40 (4H, m), 1.37–1.21 (8H, m), 0.91 (6H, t, *J* = 7.0 Hz); ^{13}C NMR (126 MHz, $CDCl_3$) δ 74.9, 68.3, 35.7, 25.8, 23.5, 14.2.

Data in accordance with literature.[17]

2-((1*R*,3*R*,4*R*)-3-Hydroxy-4-methylcyclohexyl)propane-1,2-diol (41a)

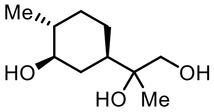

GP2 was followed using (–)-dihydrocarveol **41** (35 µL, 95% purity) in DCE with HFIP (42 µL, 0.4 mmol, 2.0 equiv.) and 12 h irradiation. Then $KHCO_2$ (336 mg, 4.0 mmol, 20 equiv.) and HCO_2H (91 µL, 2.4 mmol, 12 equiv.) at –20 °C. 2-((1*R*,3*R*,4*R*)-3-Hydroxy-4-methylcyclohexyl)propane-1,2-diol **41a** was isolated by silica gel chromatography (EtOAc, 100%) as a colourless oil (23 mg, 60%).

^{1}H NMR (400 MHz, $CDCl_3$) δ 3.58 (1H, d, *J* = 10.9 Hz), 3.44 (1H, d, *J* = 10.9 Hz), 3.16 (1H, app td, *J* = 10.2, 4.0 Hz), 1.99–1.92 (1H, dm, *J* = 11.8 Hz), 1.87 (1H, br s, O–H), 1.83–1.76 (2H, m), 1.67–1.51 (4H, m, 2 × C–H, 2 × O–H), 1.33–1.23 (2H, m), 1.15–1.08 (4H, m), 1.02 (3H, d, *J* = 6.4 Hz); ^{13}C NMR (101 MHz, $CDCl_3$) δ 76.4, 74.3, 68.4, 43.4, 40.2, 36.8, 33.0, 25.8, 20.3, 18.3; HRMS (ESI$^+$): found MNa+ 211.1307, $[C_{10}H_{20}O_3Na]^+$ requires 211.1305.

***cis*-1-Methylcyclohexane-1,2-diol (42a)**

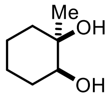

GP2 was followed using 1-methylcyclohex-1-ene **43** (24 µL, 90% purity) in EtOAc and 12 h irradiation. Then $KHCO_2$ (336 mg, 4.0 mmol, 20 equiv.) at –20 °C. Following 3 cycles of vacuum/nitrogen, the vial was placed under vacuum, disconnected from the line as quickly as possible, and a balloon of H_2 was added via a needle through the septum. Then, HCO_2H (91 µL, 2.4 mmol, 12 equiv.). *cis*-1-Methylcyclohexane-1,2-diol **42a** was isolated by silica gel chromatography (EtOAc in *n*-hexane, 50%) as a white solid (10 mg, 38%).

^{1}H NMR (400 MHz, CD_3CN) δ 3.26 (1H, ddd, *J* = 9.5, 5.6, 4.0 Hz), 2.72 (1H, d, *J* = 5.7 Hz, O–H), 2.45, (1H, s, O–H), 1.68–1.43 (5H, m), 1.37–1.19 (3H, m), 1.14 (3H, s); ^{13}C NMR (101 MHz, CD_3CN) δ 75.2, 71.8, 37.9, 31.2, 27.1, 24.3, 22.4.

Data in accordance with literature.[18]

Following **GP2** using 1-methylcyclohex-1-ene **42** (24 µL, 90% purity) in EtOAc and 12 h irradiation, then $KHCO_2$ (336 mg, 4.0 mmol, 20 equiv.) and HCO_2H (91 µL, 2.4 mmol, 12 equiv.) at –20 °C gave **42a** (29%, yield evaluated by quantitative ^{1}H NMR).

2,6-Dimethyloctane-2,3,6-triol (43a)

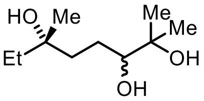

GP2 was followed using (–)-linalool **43** (38 µL, 95% purity) and 1-fluoro-3-nitrobenzene **N4** (43 µL, added at the same stage as **43**) in EtOAc (1.2 mL) and 12 h irradiation. Then $KHCO_2$ (336 mg, 4.0 mmol, 20 equiv.) and HCO_2H (91 µL, 2.4 mmol, 12 equiv.) at –20 °C. 2,6-Dimethyloctane-2,3,6-triol **43a** was isolated by silica gel chromatography (MeOH in CH_2Cl_2, 6%) as a pale yellow oil (18 mg, 47%, d.r. 1.0:1).

Mixture of diastereomers, ^{1}H NMR (400 MHz, $CDCl_3$) δ 3.36 (1H, ddd, *J* = 10.5, 7.0, 1.8 Hz), 1.79–1.66 (1H, m), 1.63–1.48 (4H, m), 1.46–1.32 (1H, m), 1.21 (3H, s), 1.18–1.16 (6H, m), 0.90 (3H, app q, *J* = 7.4 Hz), O–H not observed; ^{13}C NMR (101 MHz, $CDCl_3$) δ 79.3, 79.2, 73.3, 73.3, 73.1, 73.0, 38.4, 38.3, 35.5, 34.4, 26.8, 26.7, 26.7, 26.1, 25.9, 25.8, 23.5, 23.4, 8.5, 8.4; HRMS (ESI$^+$): found MNa+ 213.1463, $[C_{10}H_{22}O_3Na]^+$ requires 213.1461.

7.2 Characterisation of Anilines

Material for isolation acquired from selected dihydroxylation reactions. The ^{1}H NMR yield of aniline was in accordance with the yield of the relevant diol. All anilines were isolated by silica gel chromatography (EtOAc in hexane 20−50%).

3-Nitro-5-(trifluoromethyl)aniline (2)

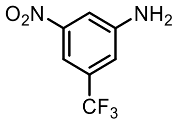

^{1}H NMR (400 MHz, $CDCl_3$) δ 7.82 (1H, s),7.64 (1H, app t, *J* = 2.1 Hz), 7.16 (1H, s), 4.23 (2H, br s, N–H); ^{19}F {^{1}H} NMR (376 MHz, $CDCl_3$) δ –63.2.

Off-white solid. Data in accordance with literature.[19] Commercially available CAS 401-94-5.

5-(Trifluoromethyl)benzene-1,3-diamine (3)

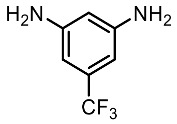

^{1}H NMR (400 MHz, $CDCl_3$) δ 6.33–6.31 (2H, m), 6.12 (1H, s), 3.72 (4H, br s, N–H); ^{19}F {^{1}H} NMR (376 MHz, $CDCl_3$) δ –63.3.

Off-white solid. Data in accordance with literature.[20] Commercially available CAS 368-53-6.

3,5-Bis(trifluoromethyl)aniline (46)

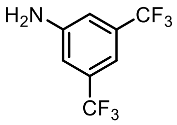

^{1}H NMR (400 MHz, $CDCl_3$) δ 7.21 (1H, s), 7.03 (2H, s), 4.01 (2H, br s, N–H); ^{13}C NMR (101 MHz, $CDCl_3$) δ 147.5, 132.7 (q, *J* = 32.8 Hz), 123.6 (q, *J* = 272.6 Hz), 114.3 (q, *J* = 4.0 Hz), 111.7 (app dt, *J* = 7.5, 3.7 Hz); ^{19}F NMR (471 MHz, $CDCl_3$) δ –63.3.

Colourless oil. Data in accordance with literature.[21] Commercially available CAS 325-74-5.

3-Fluoro-4-((trifluoromethyl)sulfonyl)aniline (47)

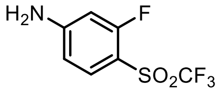

^{1}H NMR (400 MHz, $CDCl_3$) δ 7.66 (1H, dd, *J* = 8.8, 7.6 Hz), 6.52 (1H, dd, *J* = 12.0, 2.2 Hz), 6.43 (1H, dd, *J* = 12.0, 2.2 Hz), 4.68 (2H, br s, N–H); ^{13}C NMR (101 MHz, $CDCl_3$) δ 163.4 (d, *J* = 261.2 Hz), 156.6 (d, *J* = 12.3 Hz), 135.2, 120.1 (q, *J* = 325.2 Hz), 110.5 (d, *J* = 2.1 Hz), 105.8–105.6 (dm, *J* = 13.8), 101.8 (d, *J* = 24.5 Hz); ^{19}F {^{1}H} NMR (376 MHz, $CDCl_3$) δ –79.3 (d, *J* = 7.9 Hz), –103.7 (q, *J* = 7.8 Hz); ^{19}F NMR (376 MHz, $CDCl_3$) δ –79.3 (d, *J* = 7.9 Hz), –103.7 (app dp, *J* = 12.0, 7.8 Hz); HRMS (ESI$^+$): found MNa+ 265.9871, $[C_7H_5F_4NO_2SNa]^+$ requires 265.9869.

White solid.

3-Fluoroaniline (48)

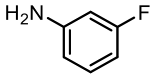

^{1}H NMR (400 MHz, $CDCl_3$) δ 7.08 (1H, app td, *J* = 8.1, 6.6 Hz), 6.47–6.41 (2H, m), 6.38 (1H, app dt, *J* = 10.9, 2.3 Hz), 3.75 (2H, br s, N–H); ^{13}C NMR (101 MHz, $CDCl_3$) δ 164.0 (d, *J* = 243.2 Hz), 148.3 (d, *J* = 10.7 Hz), 130.6 (d, *J* = 10.2 Hz), 110.8 (d, *J* = 2.5 Hz), 105.2 (d *J* = 21.3 Hz), 102.2 (d, *J* = 24.5 Hz); ^{19}F NMR (376 MHz, $CDCl_3$) δ –113.2 (ddd, *J* = 10.9, 8.9, 6.7 Hz).

Yellow oil. Data in accordance with literature.[22] Commercially available CAS 372-19-0.

8 NMR Spectra

8.1 NMR Spectra of Vicinal Diols

1a – ^{1}H NMR ($CDCl_3$, 500 MHz)

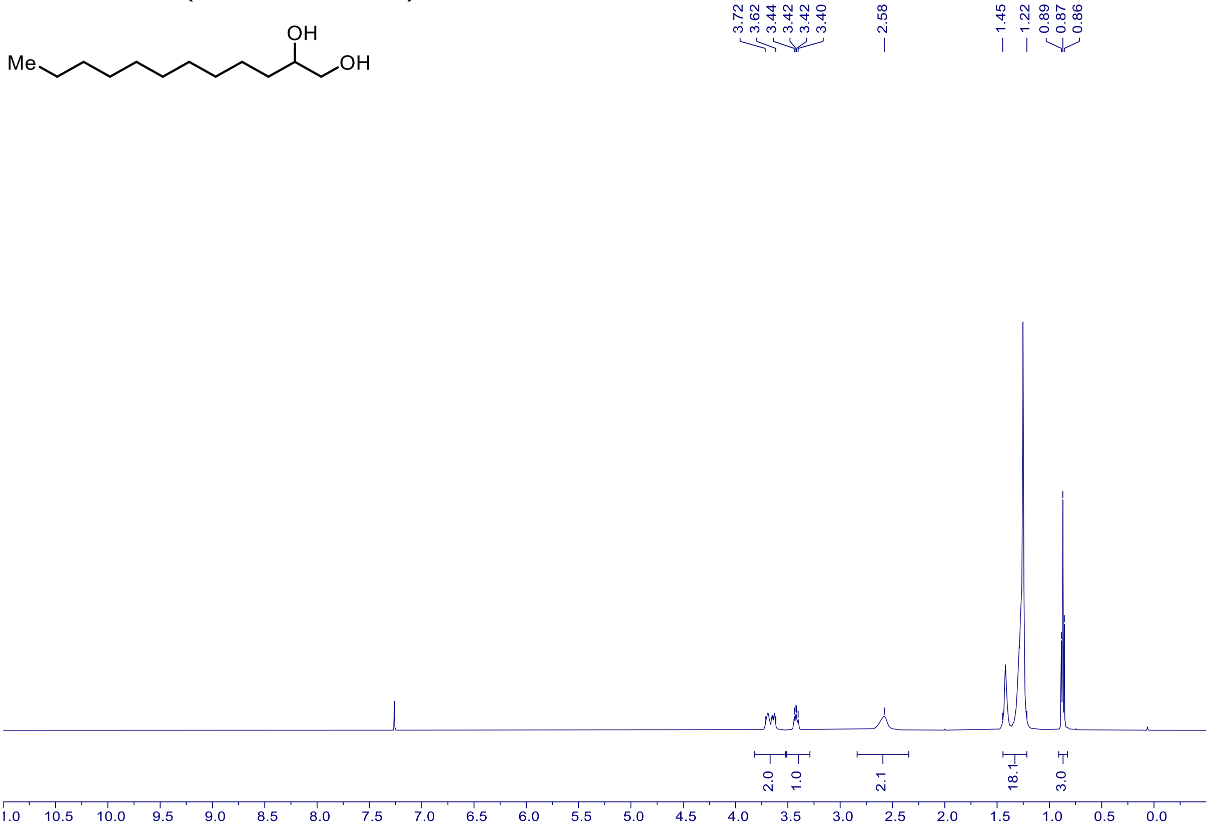

1a – ^{13}C NMR ($CDCl_3$, 126 MHz)

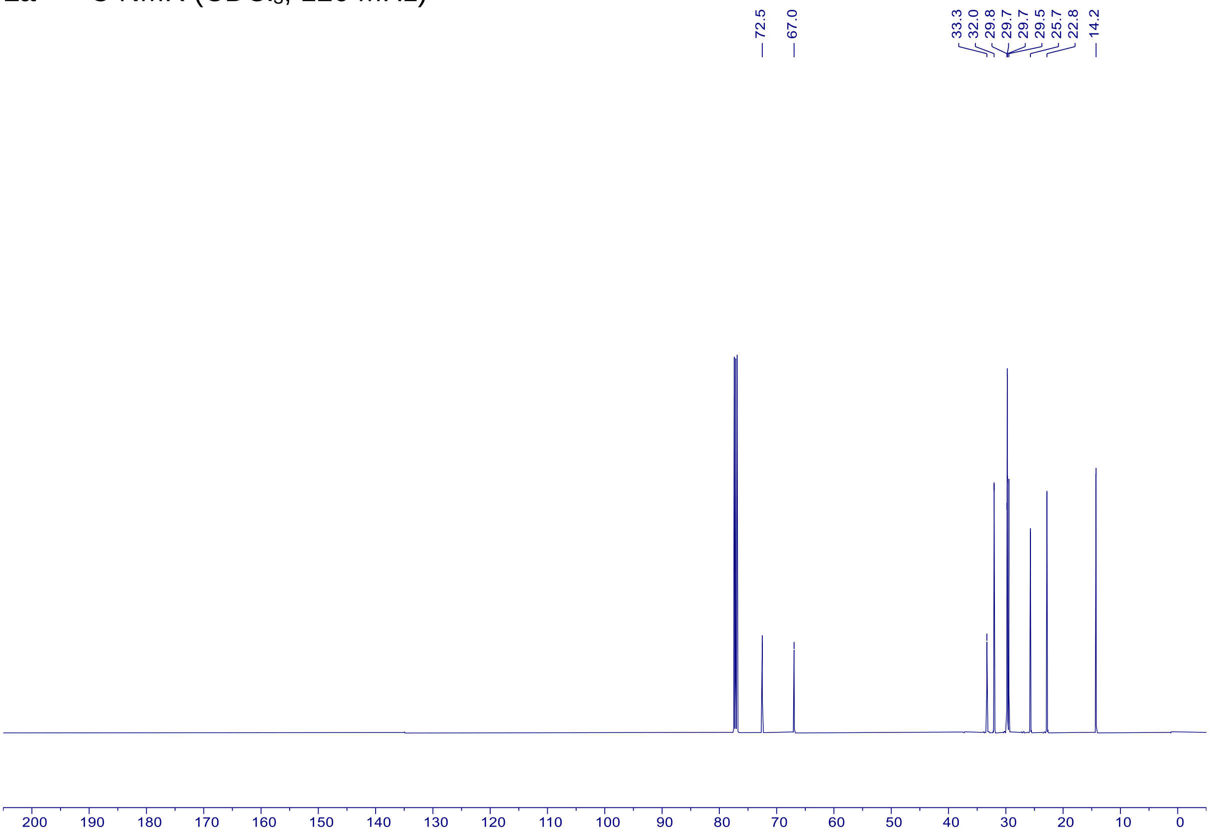

4a – ^{1}H NMR ($CDCl_3$, 500 MHz)

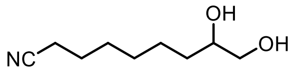

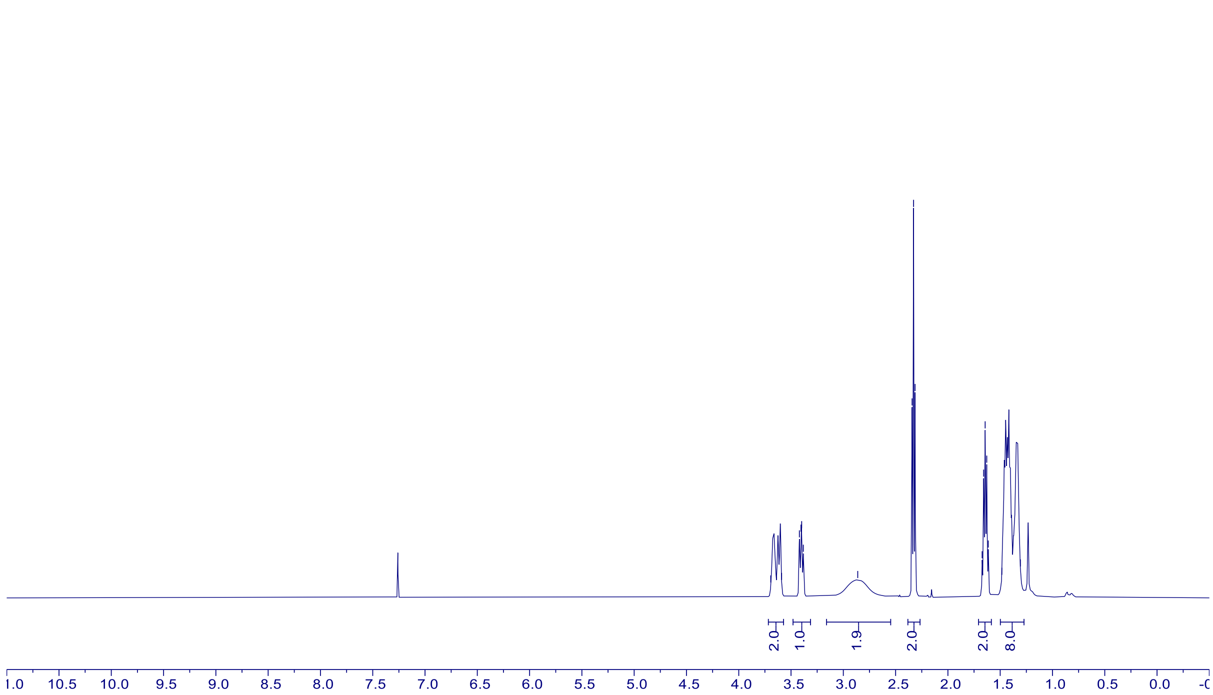

4a – ^{13}C NMR ($CDCl_3$, 101 MHz)

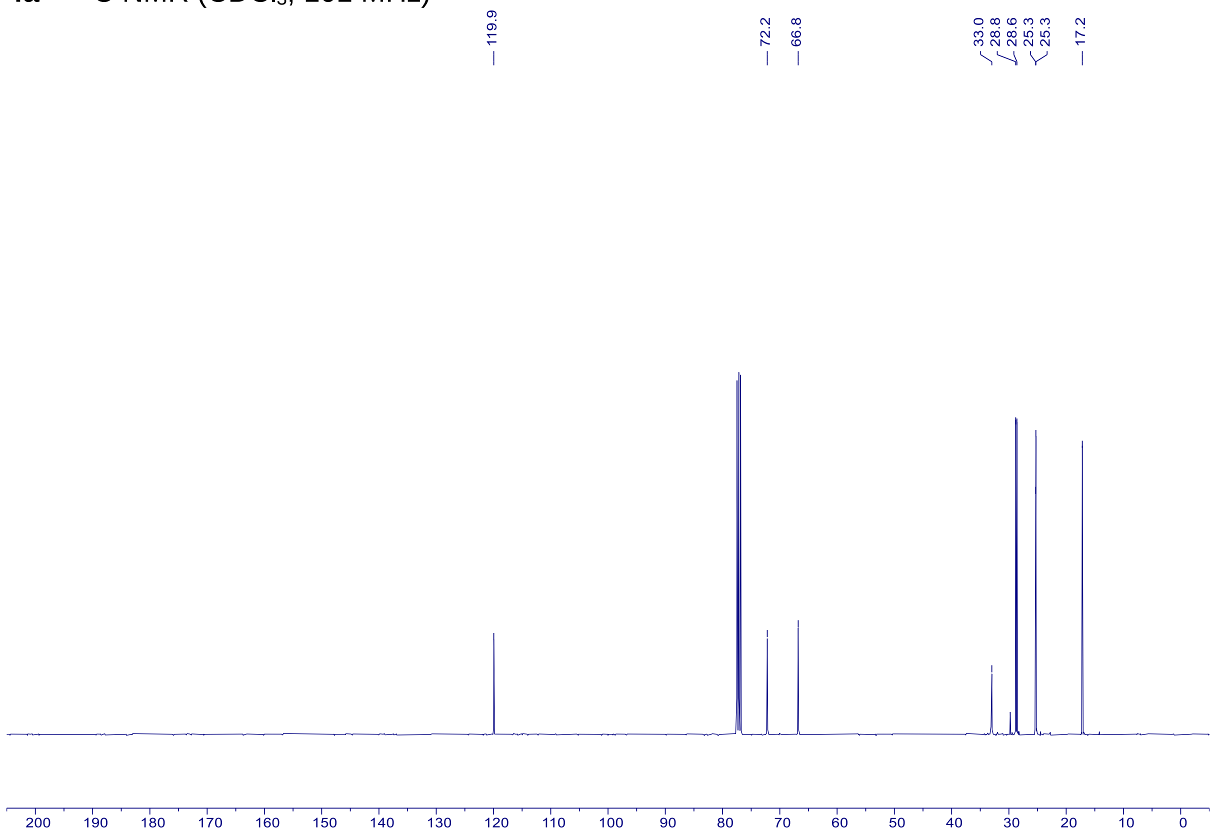

5a – ^{1}H NMR (C_6D_6, 400 MHz)

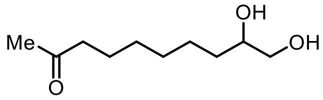

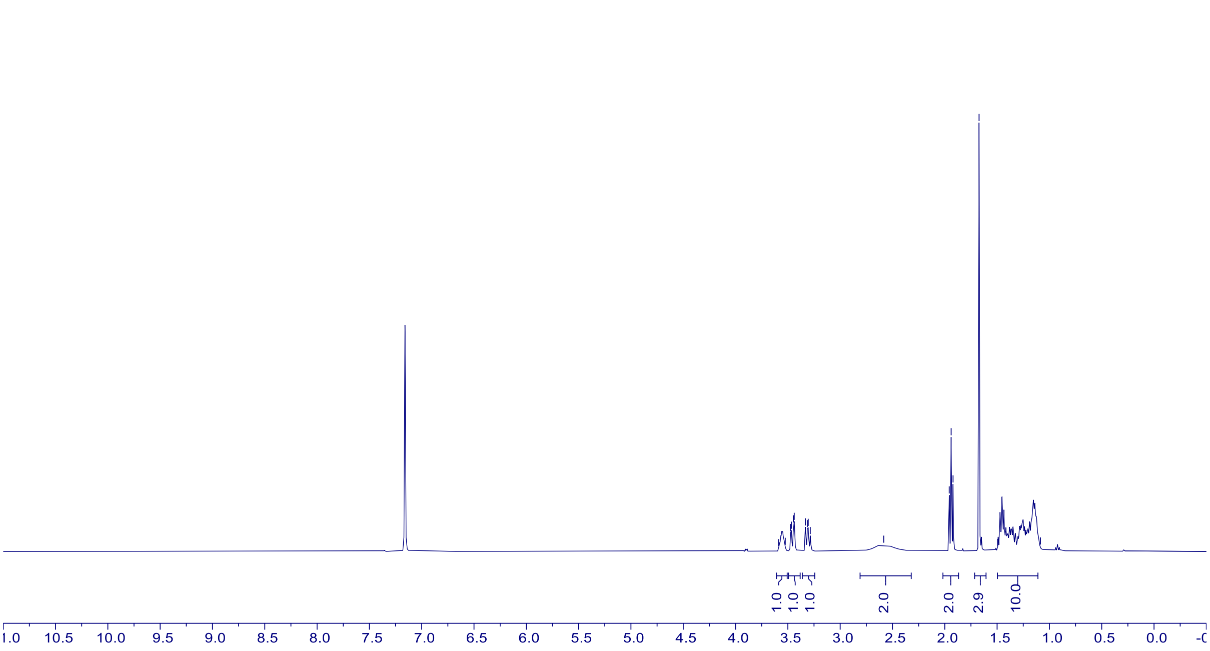

5a – ^{13}C NMR (C_6D_6, 101 MHz)

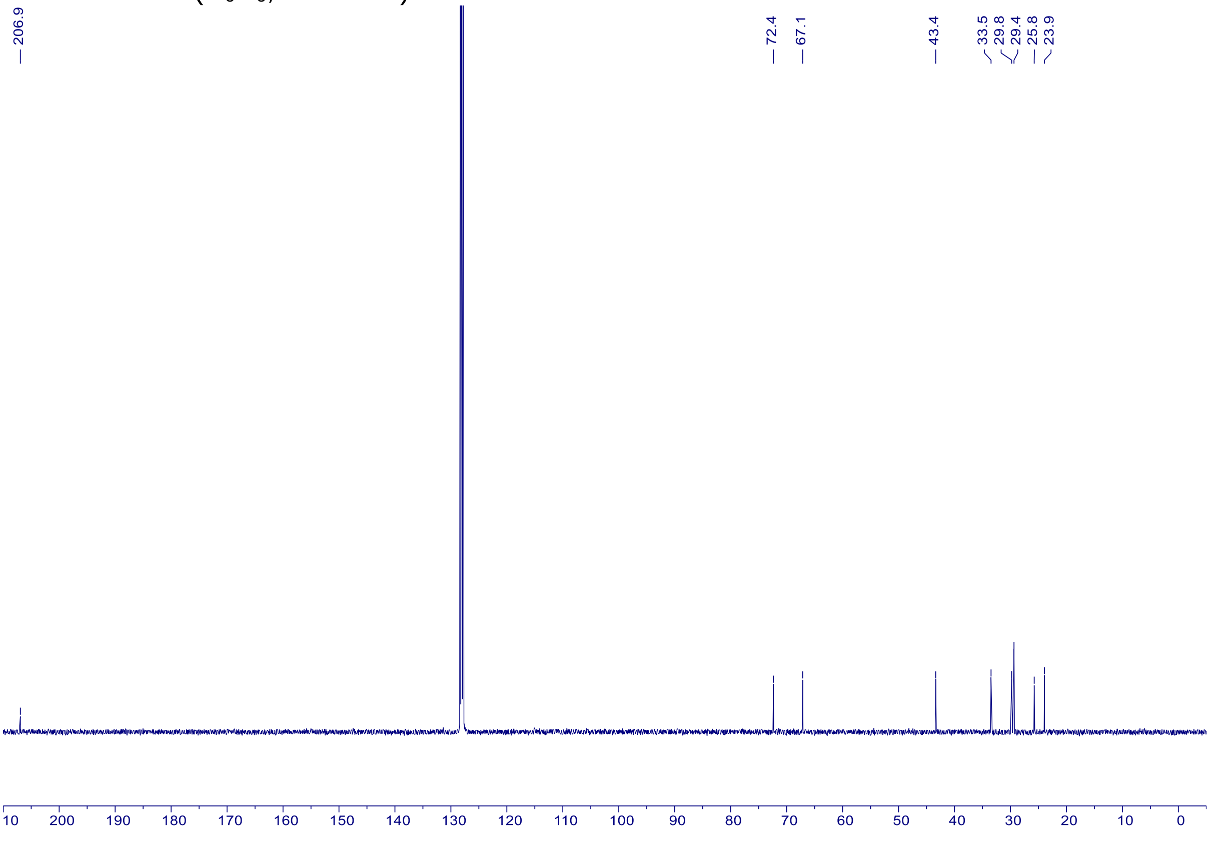

6a – ^{1}H NMR ($CDCl_3$, 400 MHz)

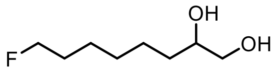

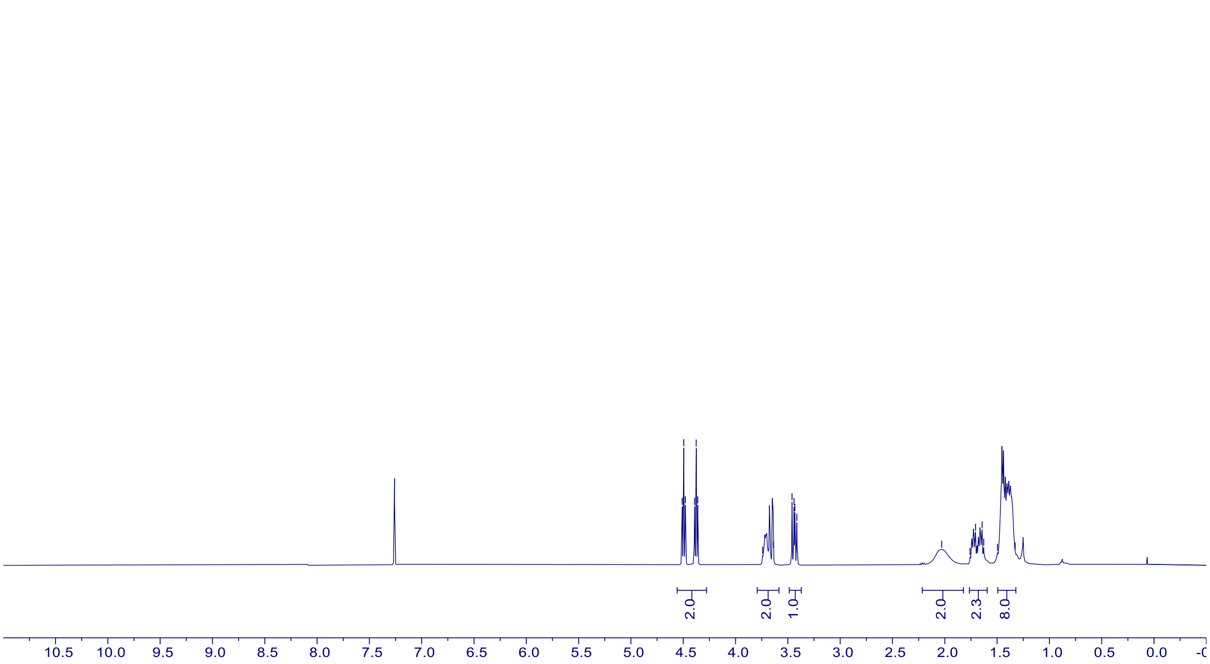

6a – ^{13}C NMR ($CDCl_3$, 101 MHz)

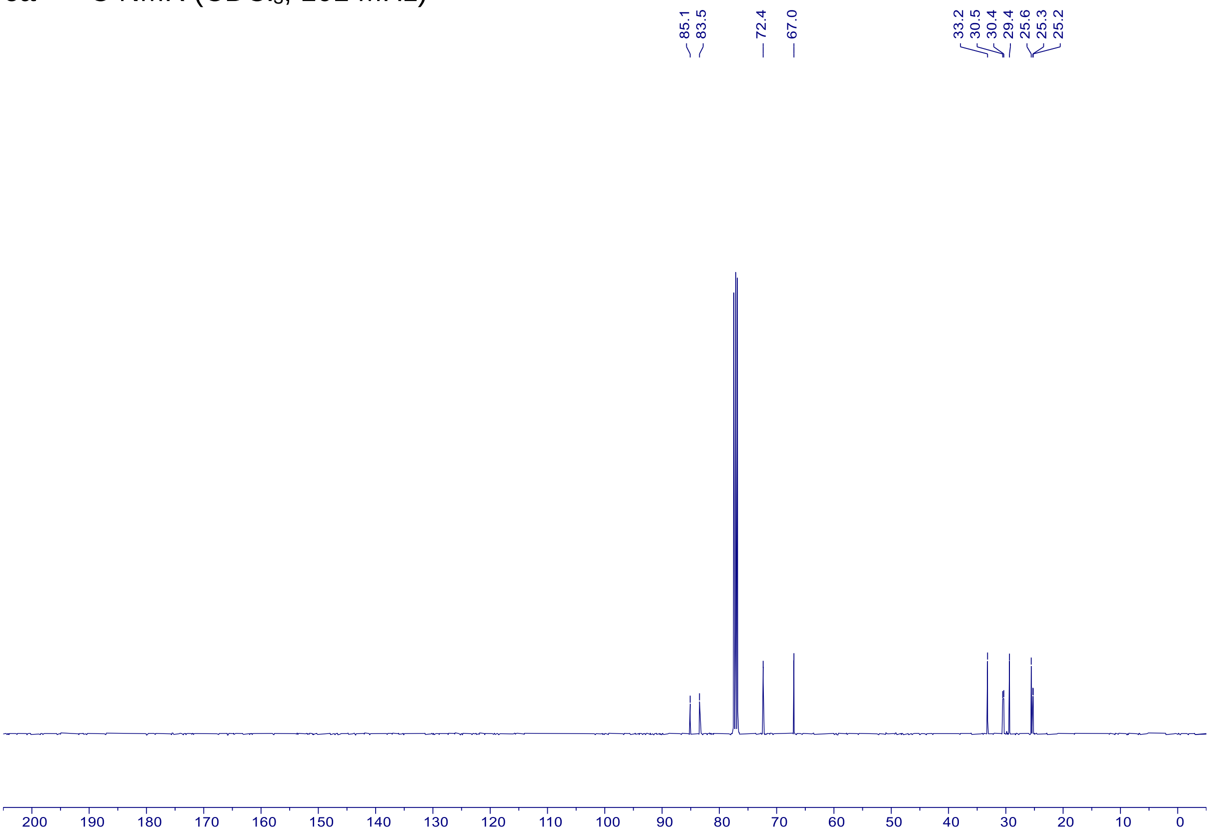

6a – ^{19}F NMR ($CDCl_3$, 376 MHz)

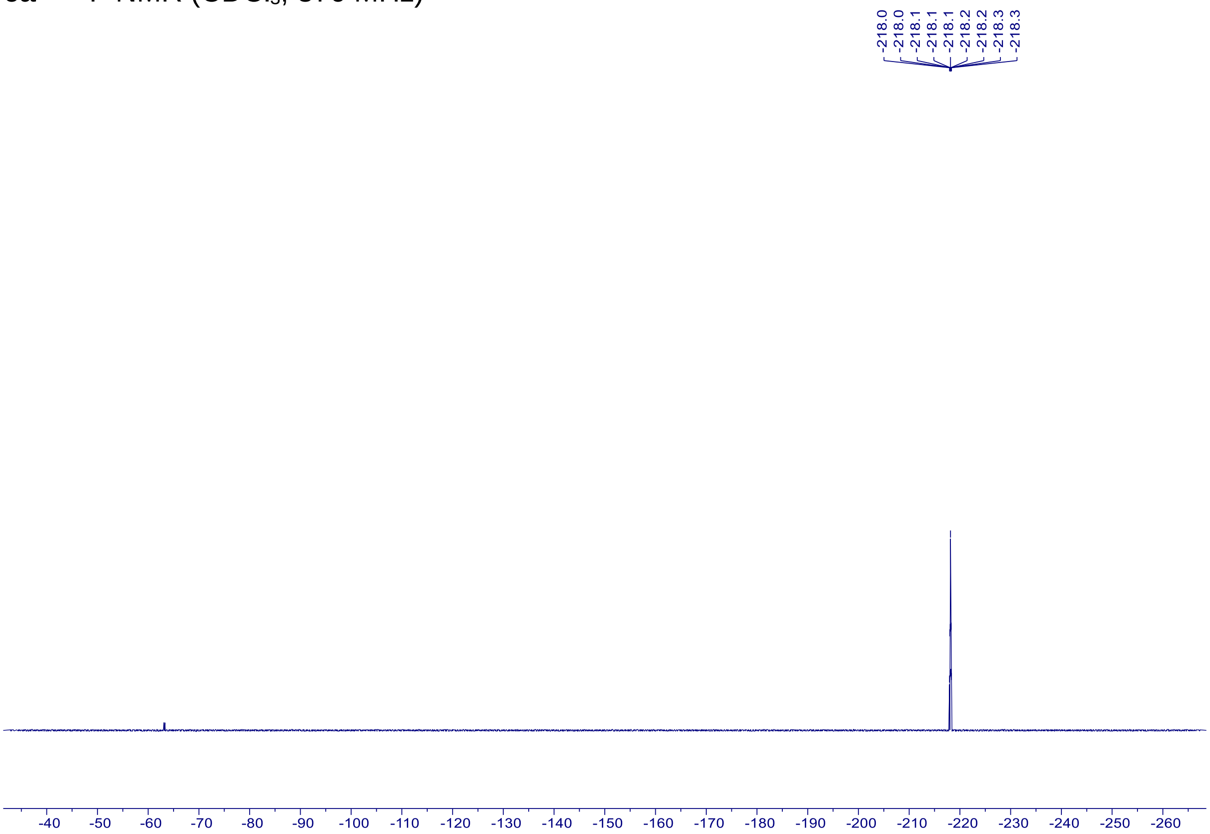

6a – ^{19}F {^{1}H} NMR ($CDCl_3$, 376 MHz)

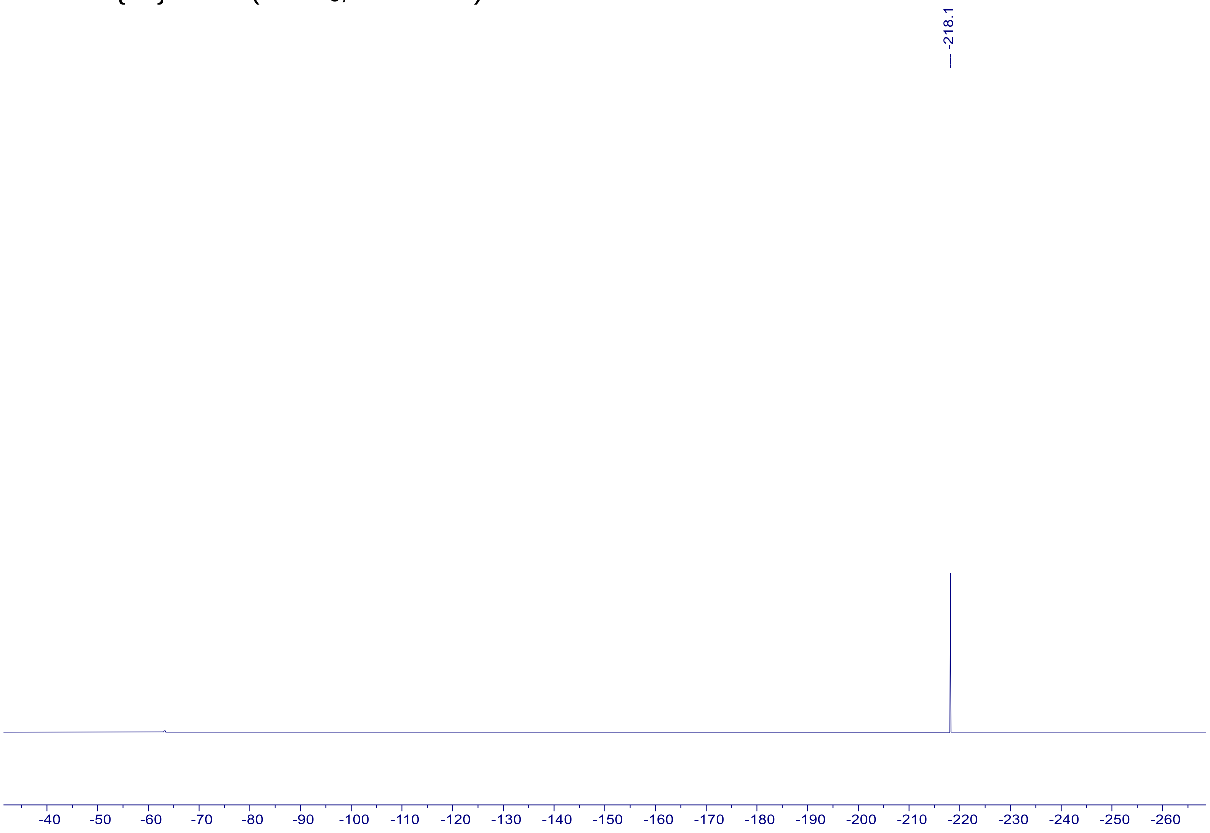

7a – ^{1}H NMR ($CDCl_3$, 500 MHz)

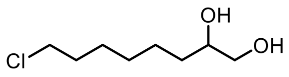

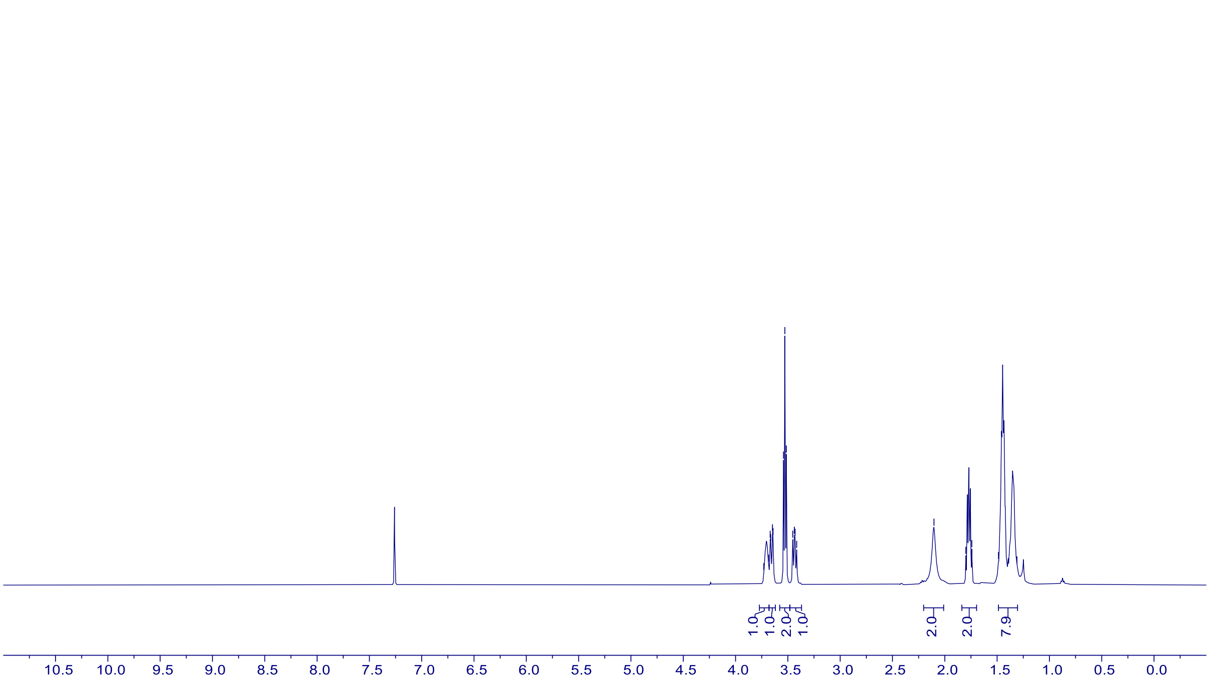

7a – ^{13}C NMR ($CDCl_3$, 101 MHz)

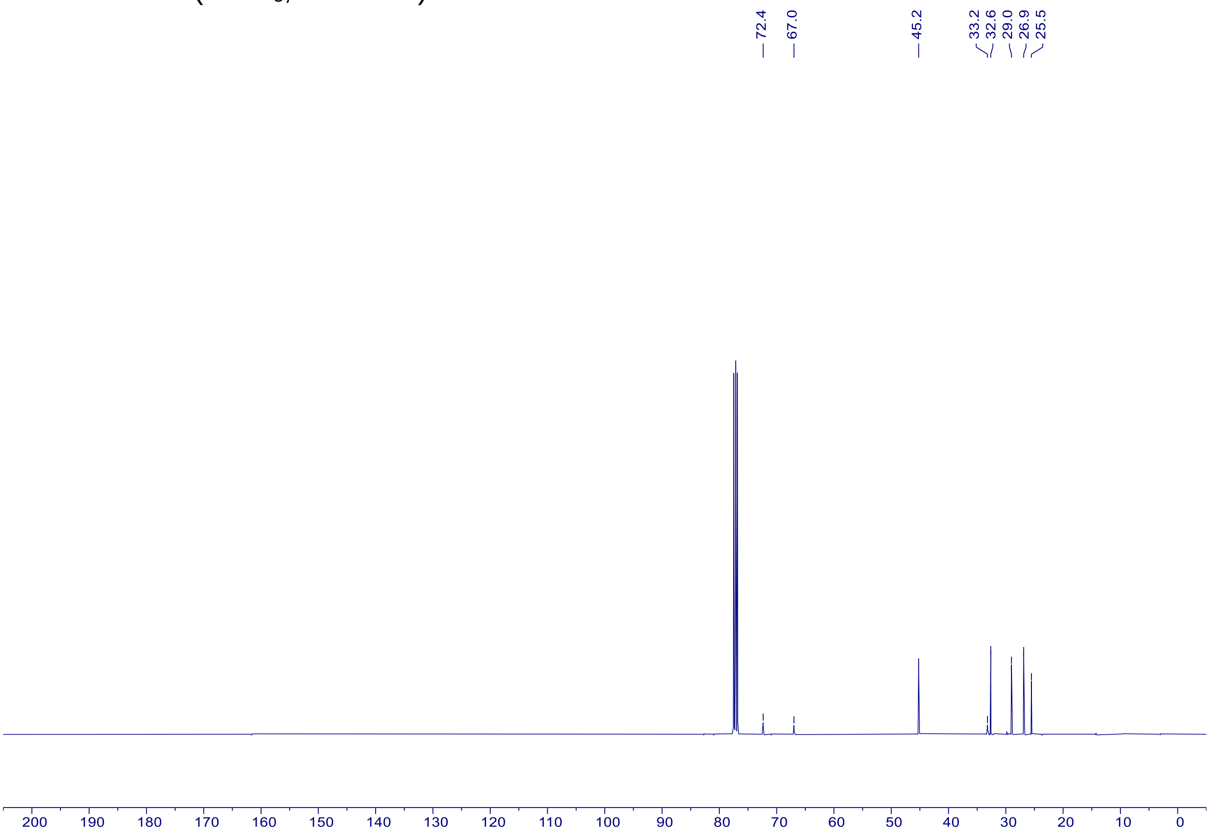

8a – ^{1}H NMR ($CDCl_3$, 400 MHz)

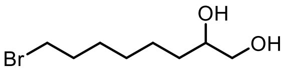

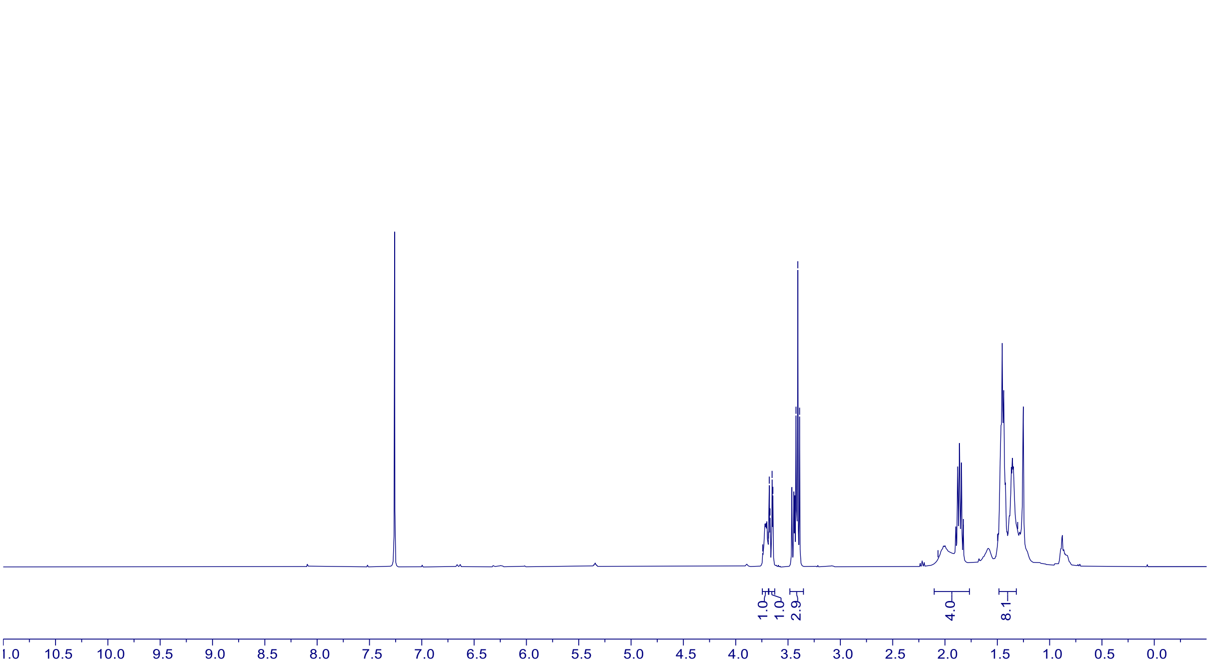

8a – ^{13}C NMR ($CDCl_3$, 101 MHz)

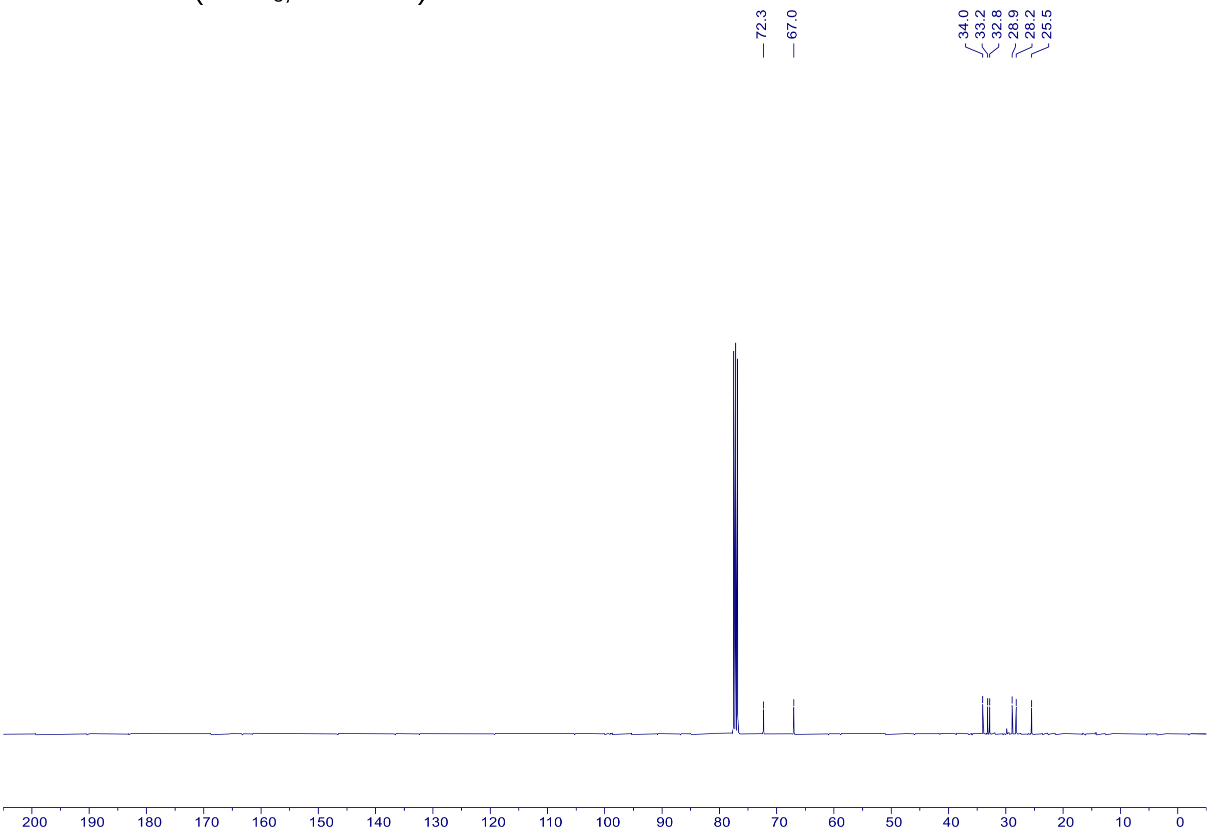

9a – ^{1}H NMR ($CDCl_3$, 500 MHz)

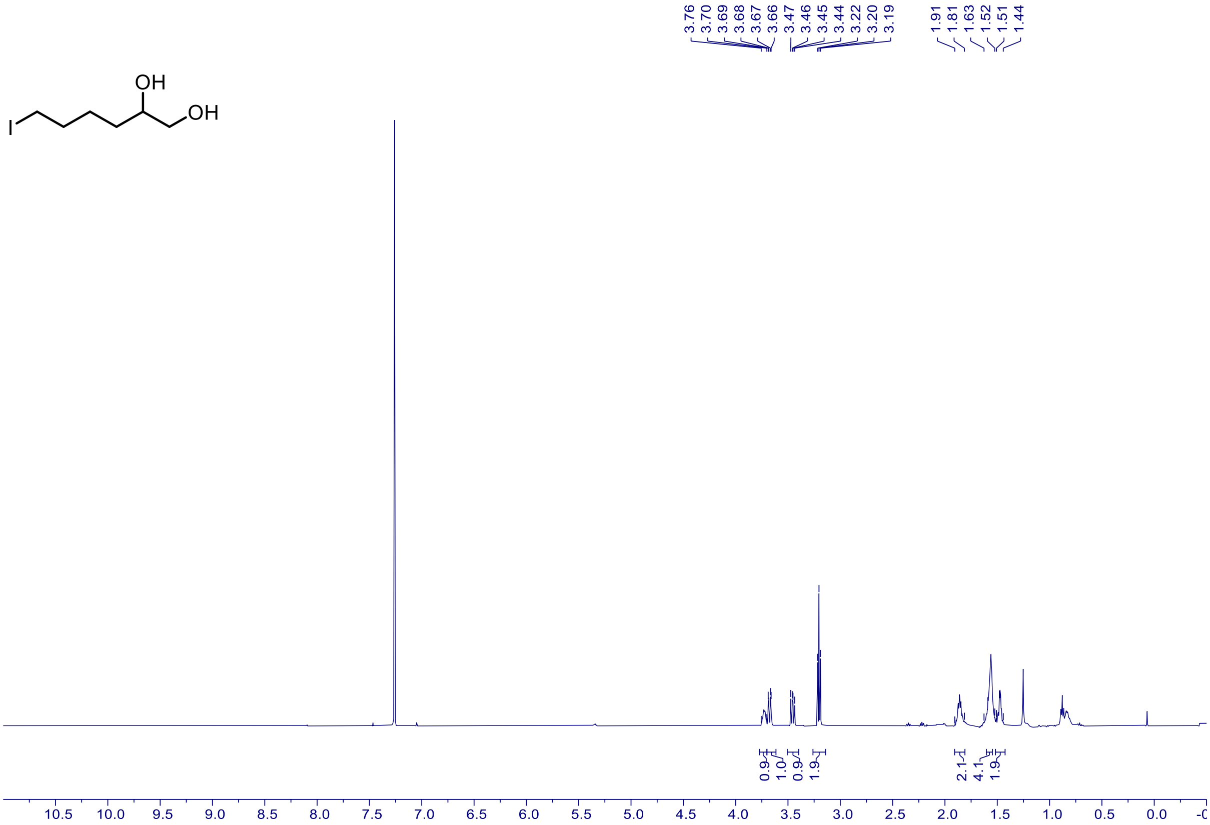

9a – ^{13}C NMR ($CDCl_3$, 126 MHz)

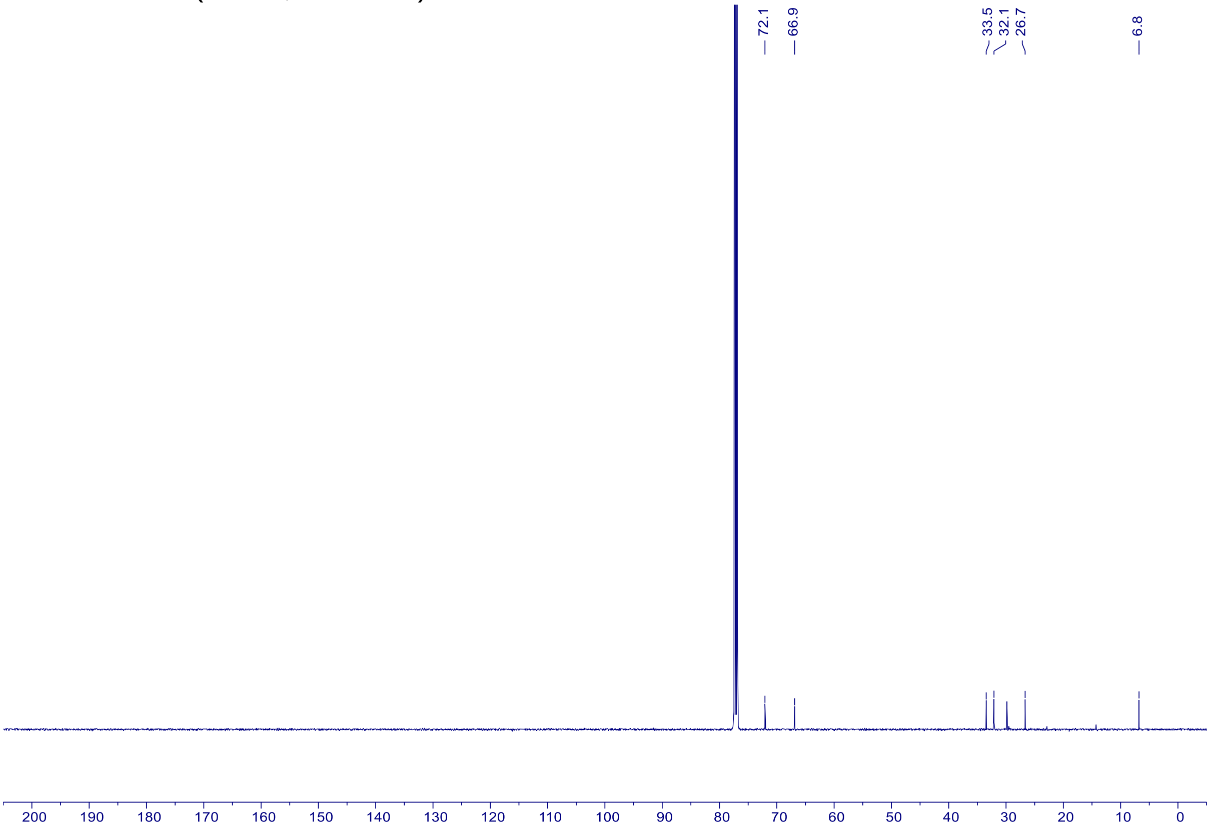

10a – ^{1}H NMR (D_2O, 400 MHz)

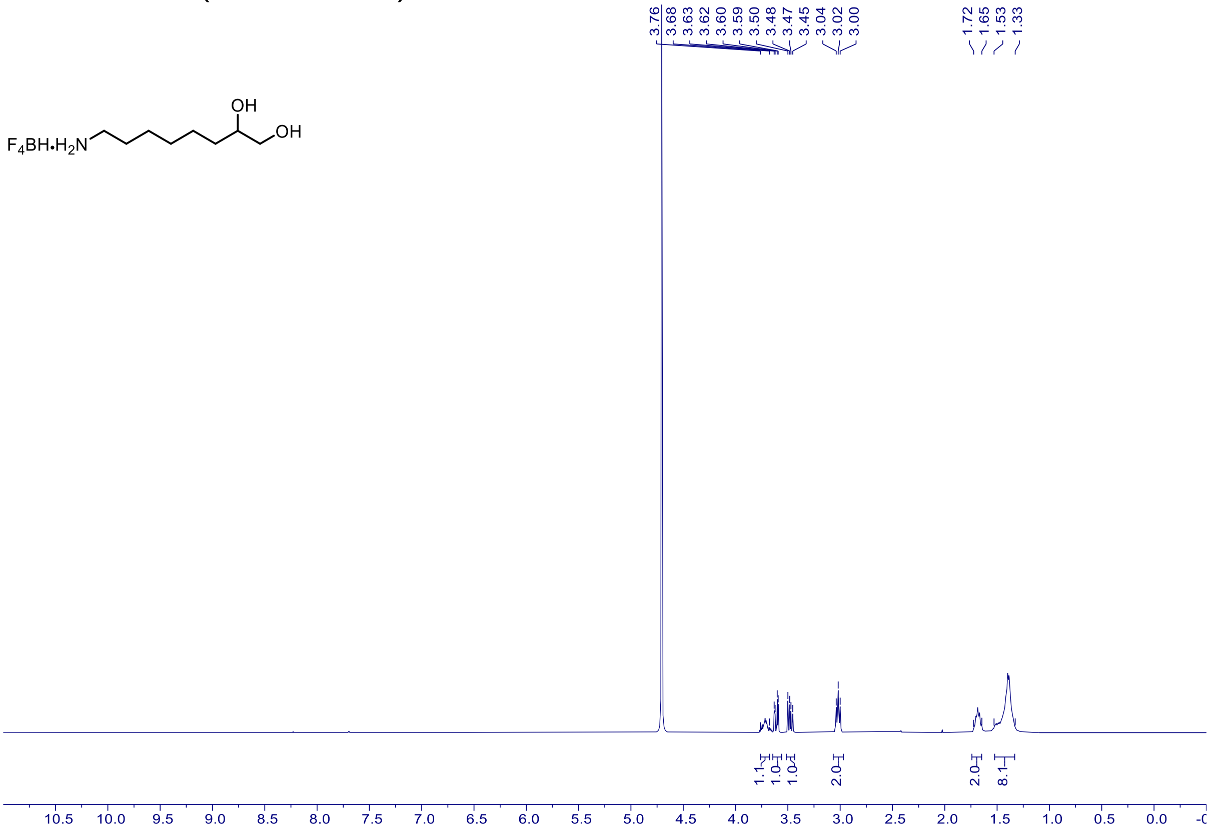

10a – ^{13}C NMR (D_2O, 126 MHz)

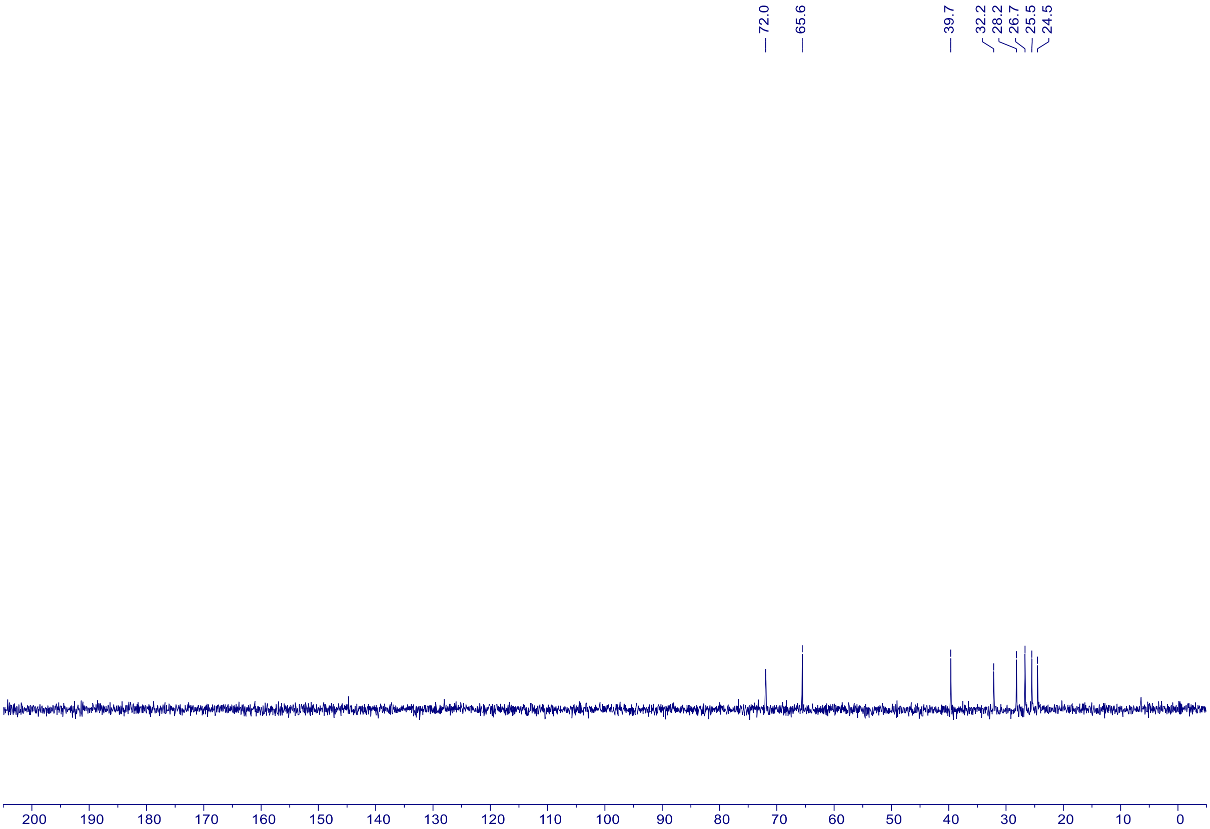

11a – ^{1}H NMR ($CDCl_3$, 400 MHz)

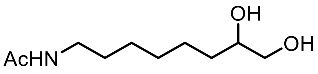

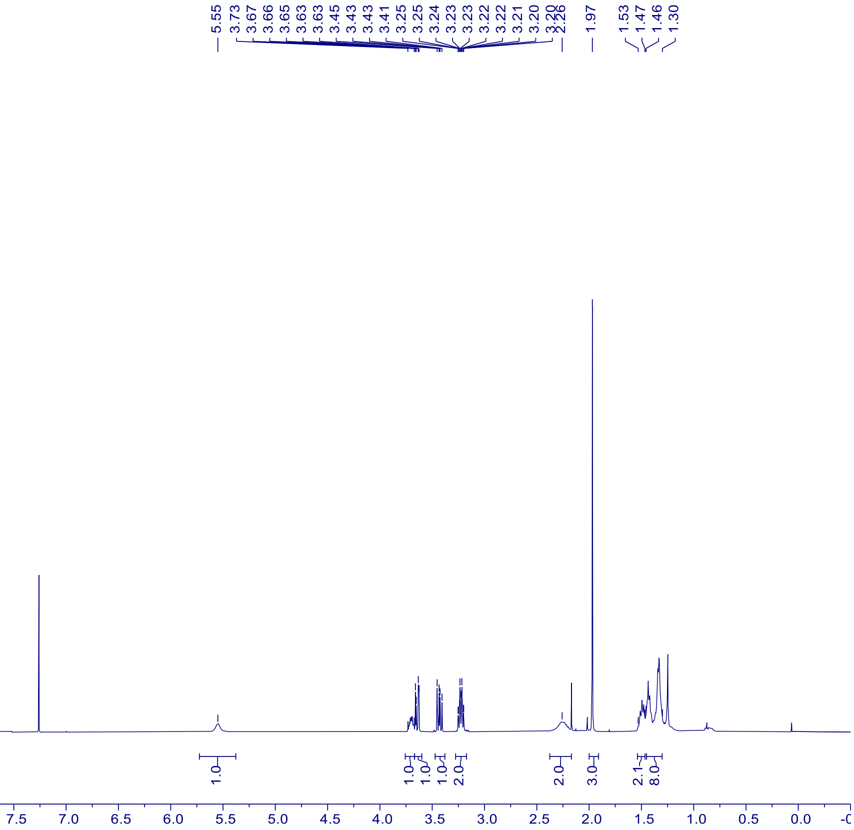

11a – ^{13}C NMR ($CDCl_3$, 101 MHz)

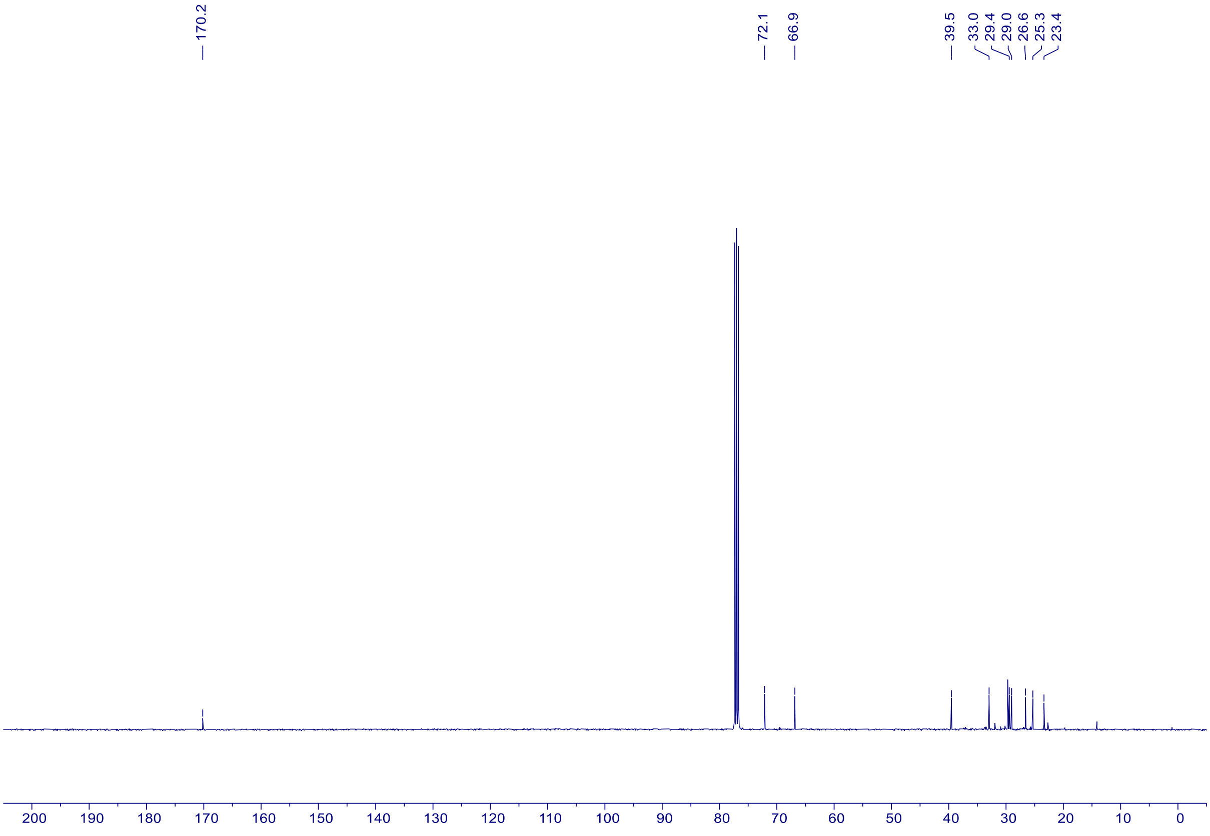

12a – ^{1}H NMR ($CDCl_3$, 500 MHz)

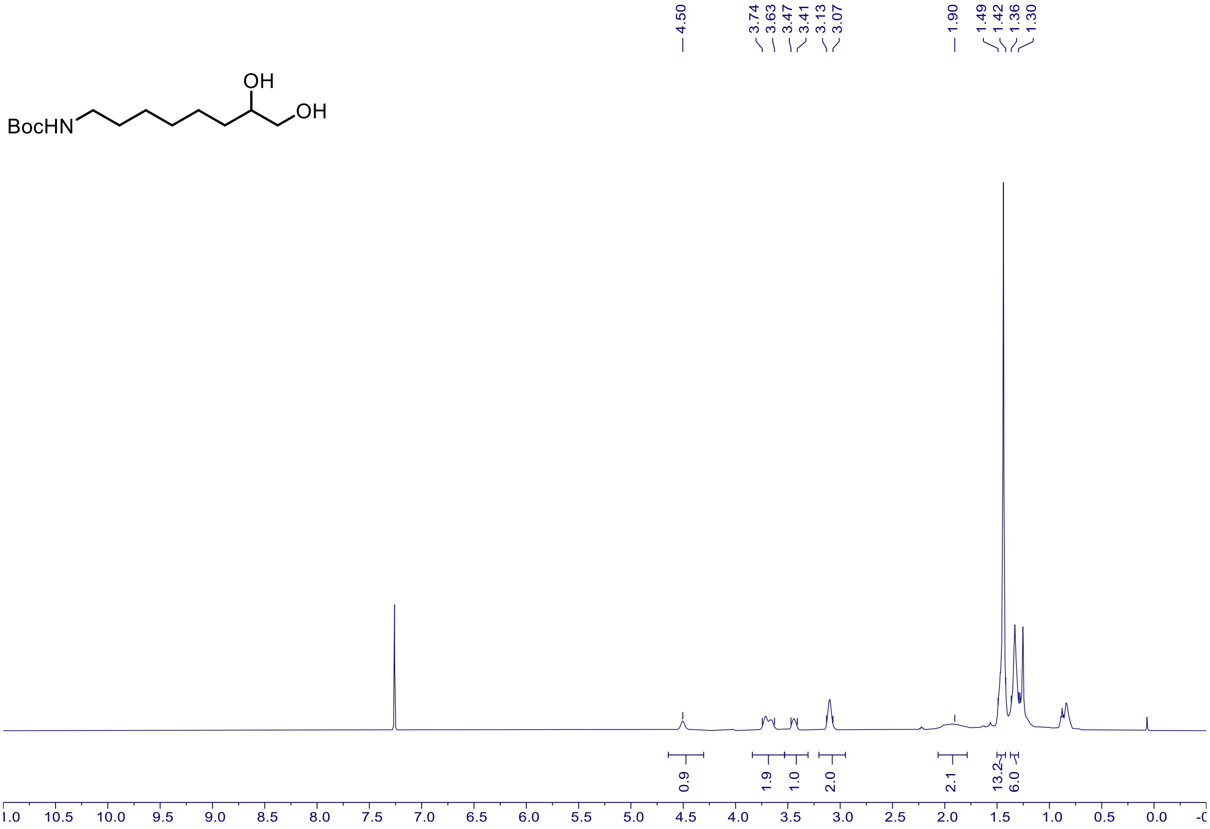

12a – ^{13}C NMR ($CDCl_3$, 126 MHz)

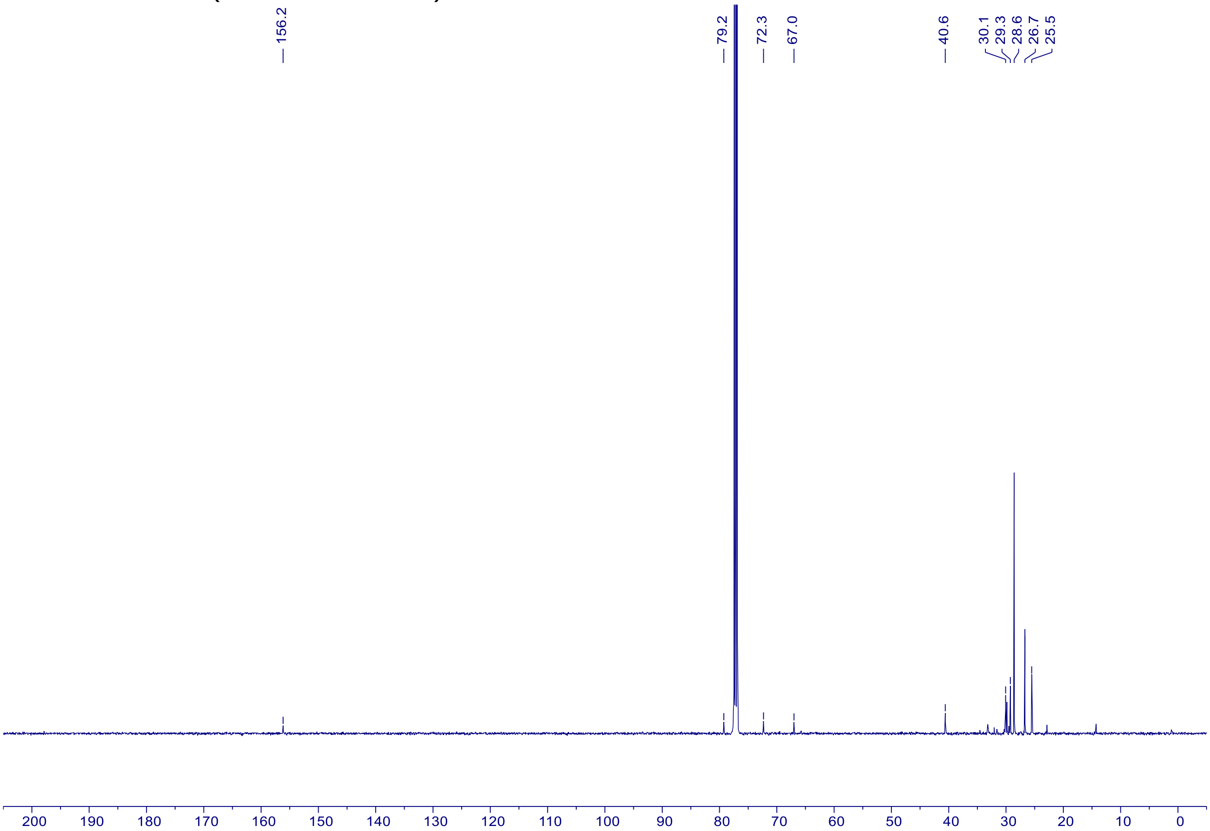

13a – ^{1}H NMR ($CDCl_3$, 400 MHz)

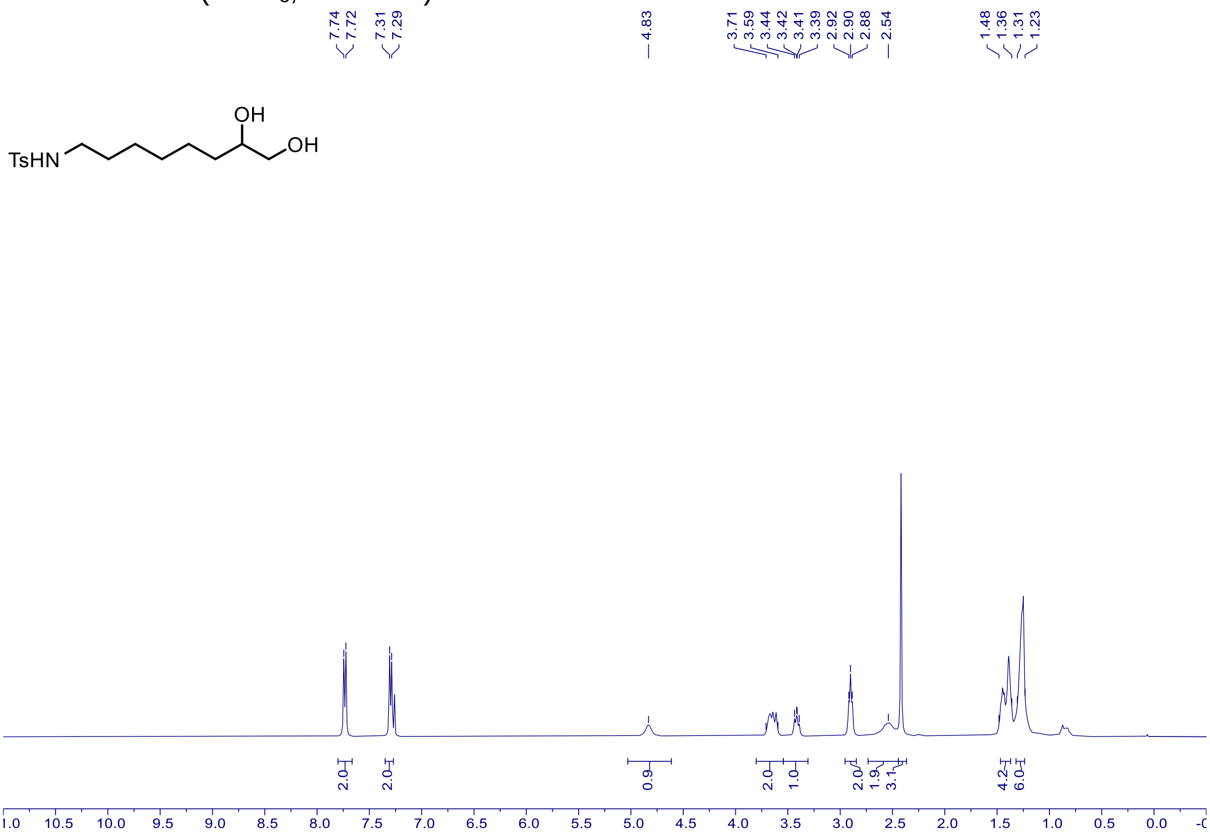

13a – ^{13}C NMR ($CDCl_3$, 101 MHz)

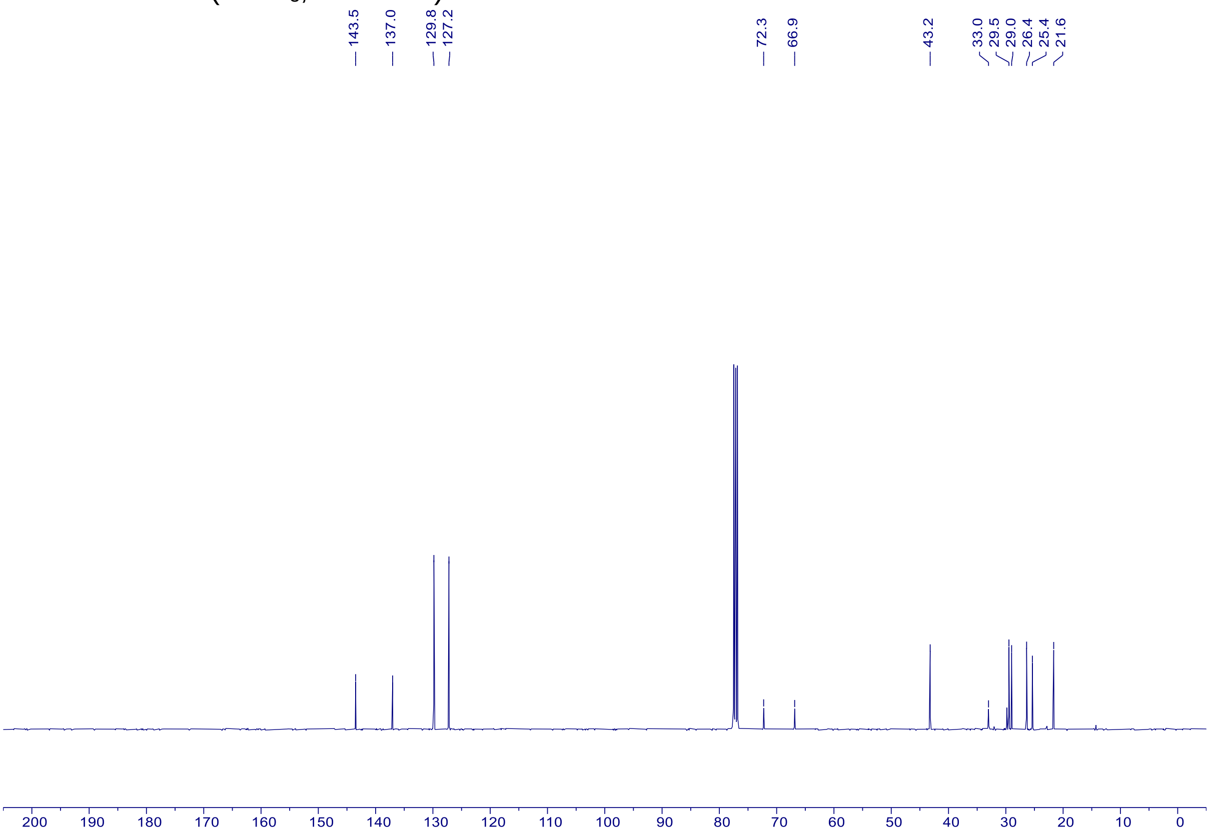

14a – ^{1}H NMR ($CDCl_3$, 400 MHz)

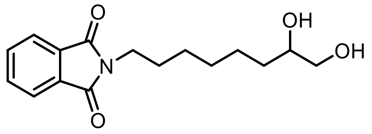

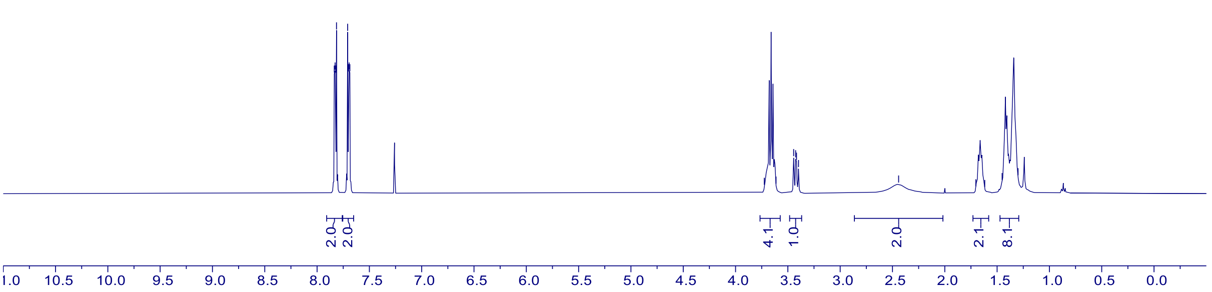

14a – ^{13}C NMR ($CDCl_3$, 101 MHz)

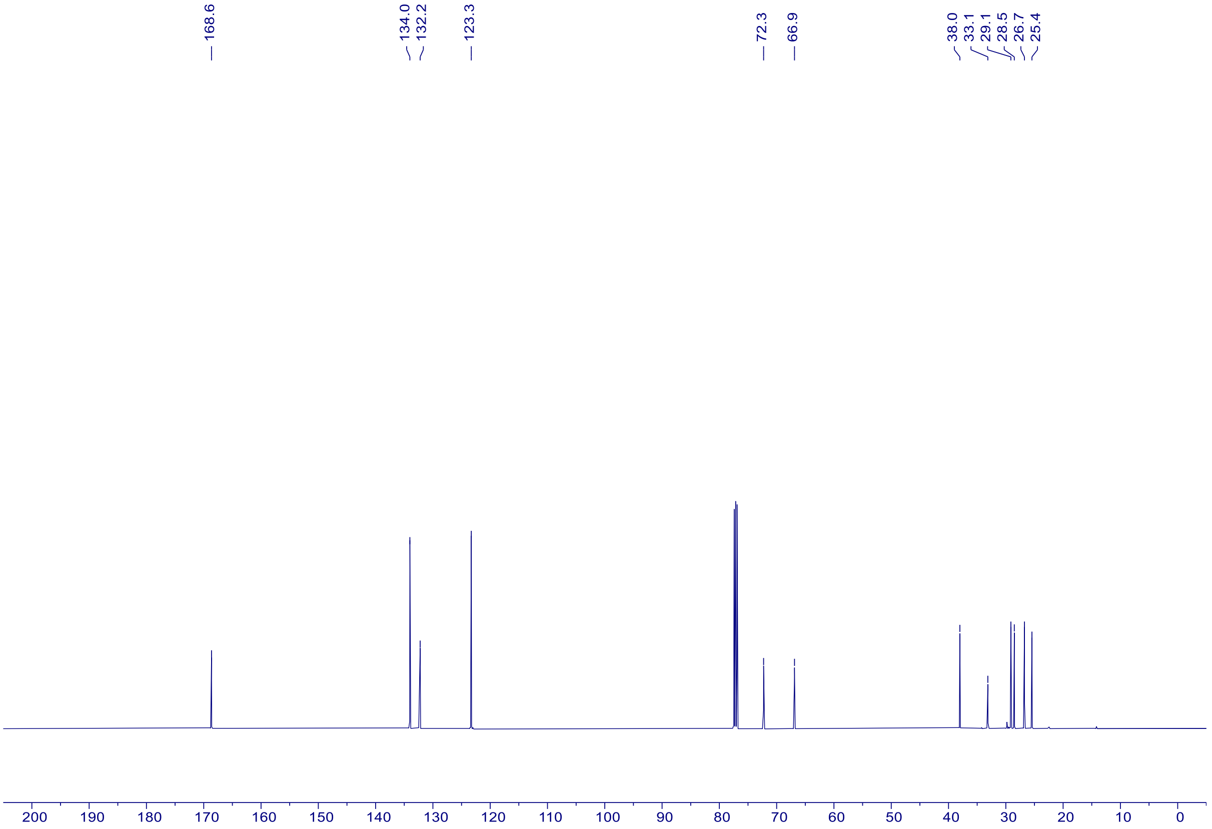

15a – ^{1}H NMR ($CDCl_3$, 400 MHz)

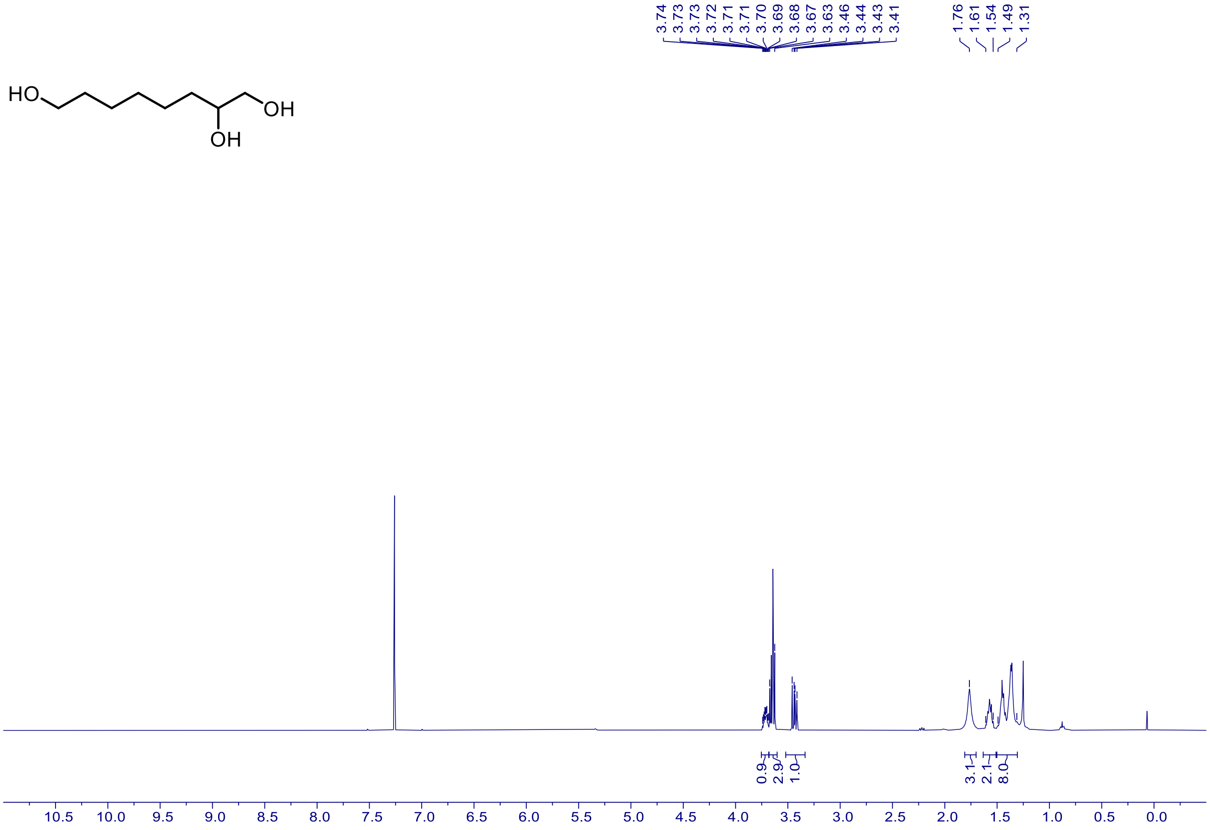

15a – ^{13}C NMR ($CDCl_3$, 101 MHz)

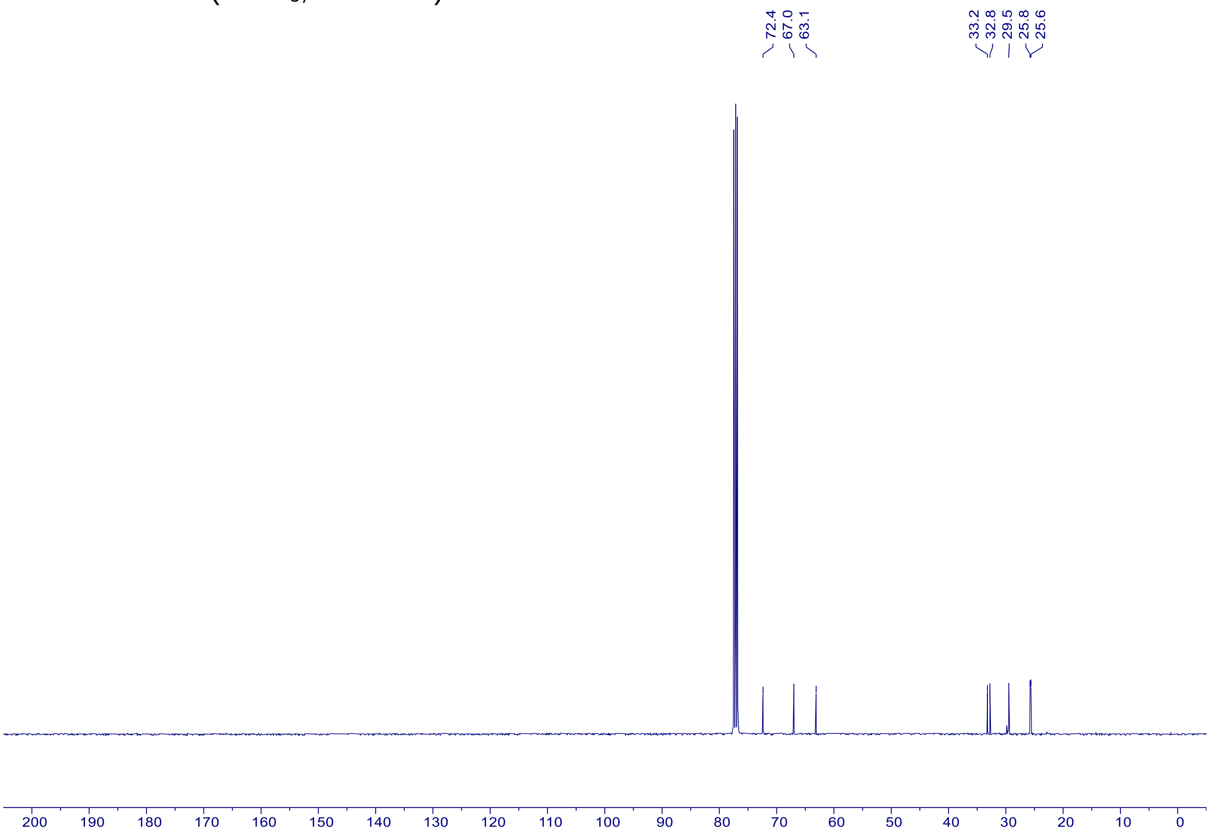

16a’ – ^{1}H NMR ($CDCl_3$, 400 MHz)

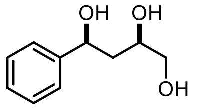

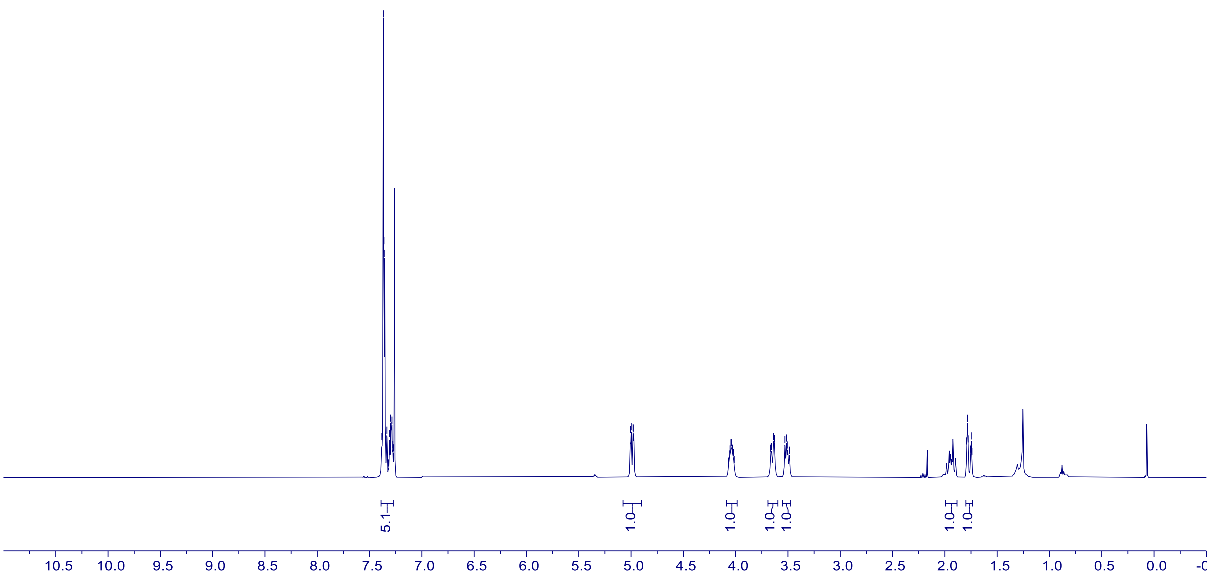

16a’ – ^{13}C NMR ($CDCl_3$, 101 MHz)

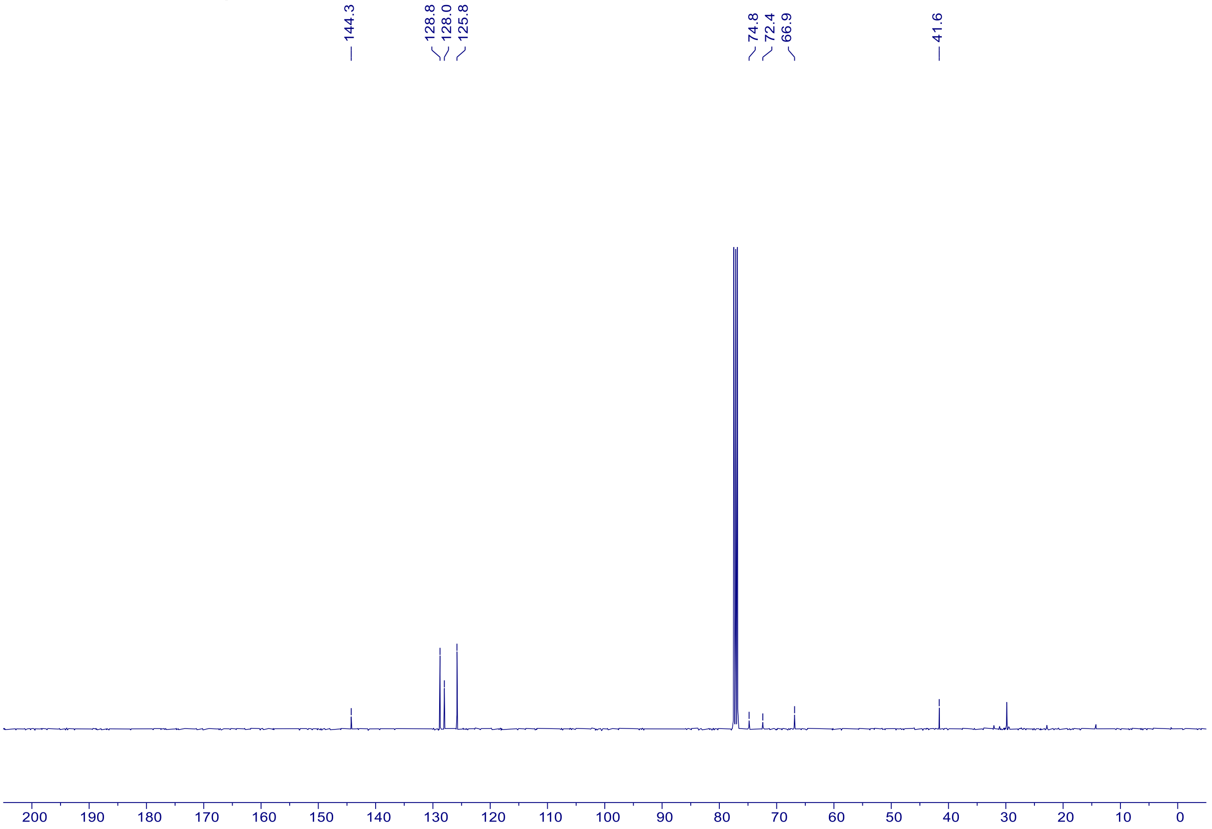

16a'' – ^{1}H NMR ($CDCl_3$, 400 MHz)

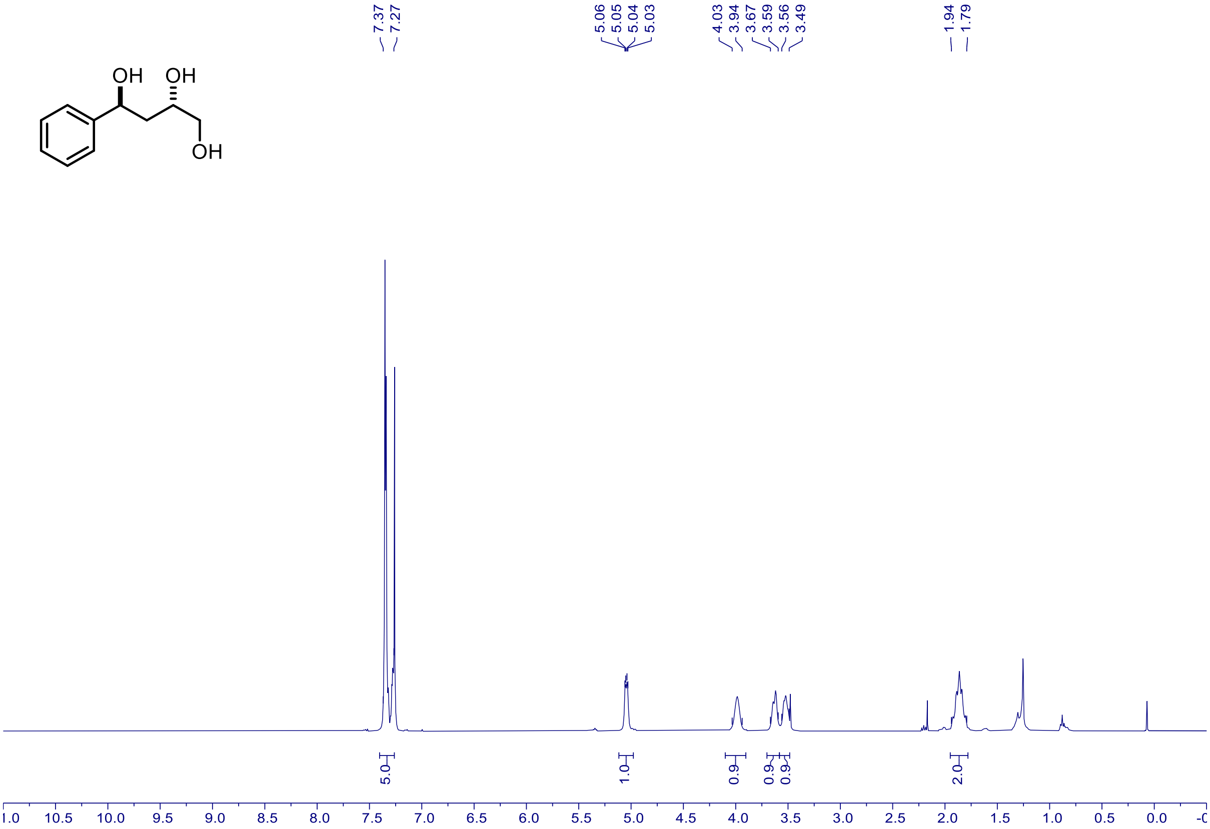

16a'' – ^{13}C NMR ($CDCl_3$, 101 MHz)

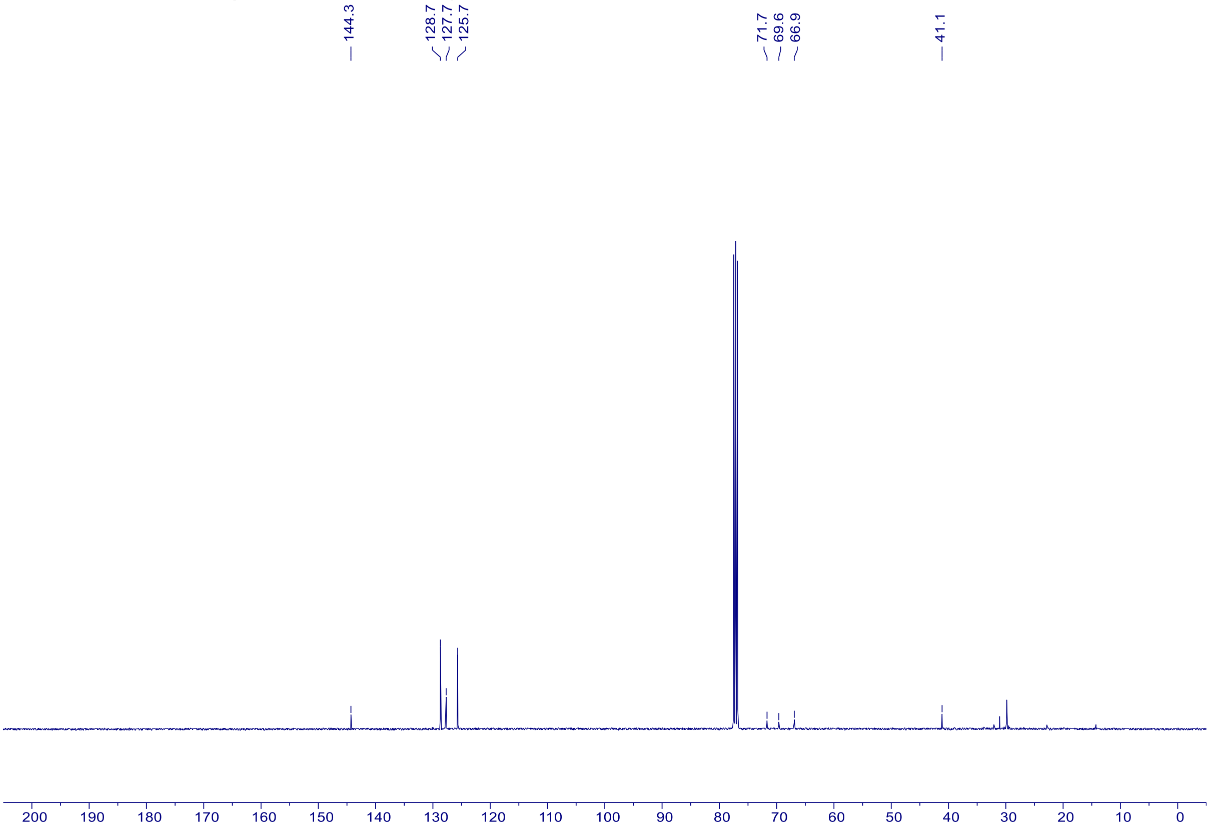

17a – ^{1}H NMR ($CDCl_3$, 400 MHz)

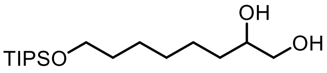

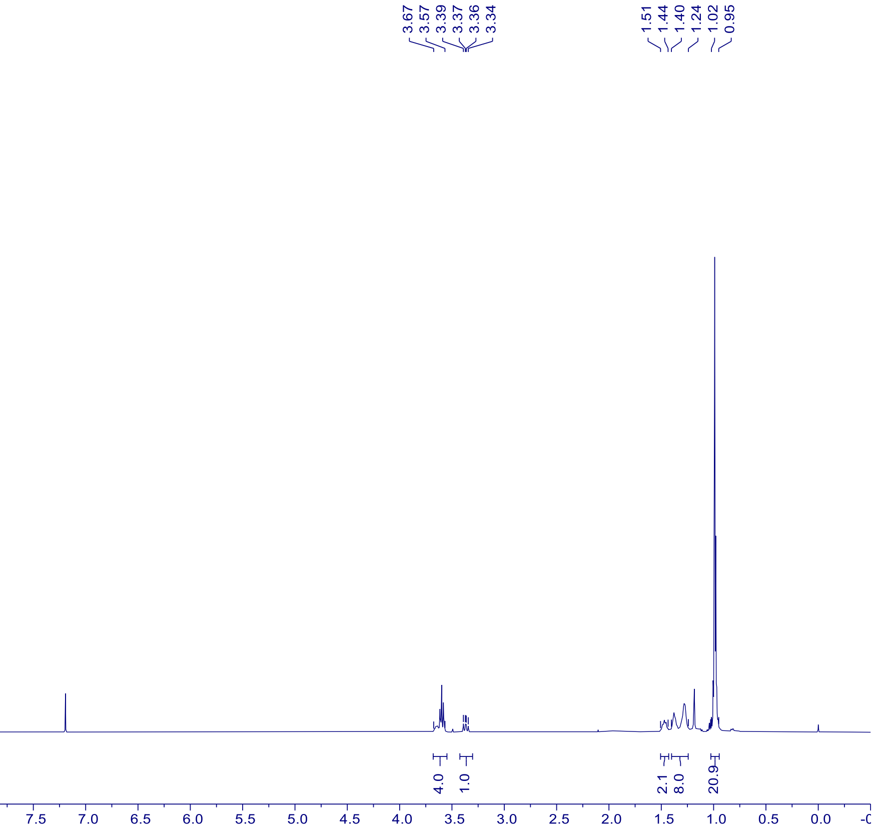

17a – ^{13}C NMR ($CDCl_3$, 101 MHz)

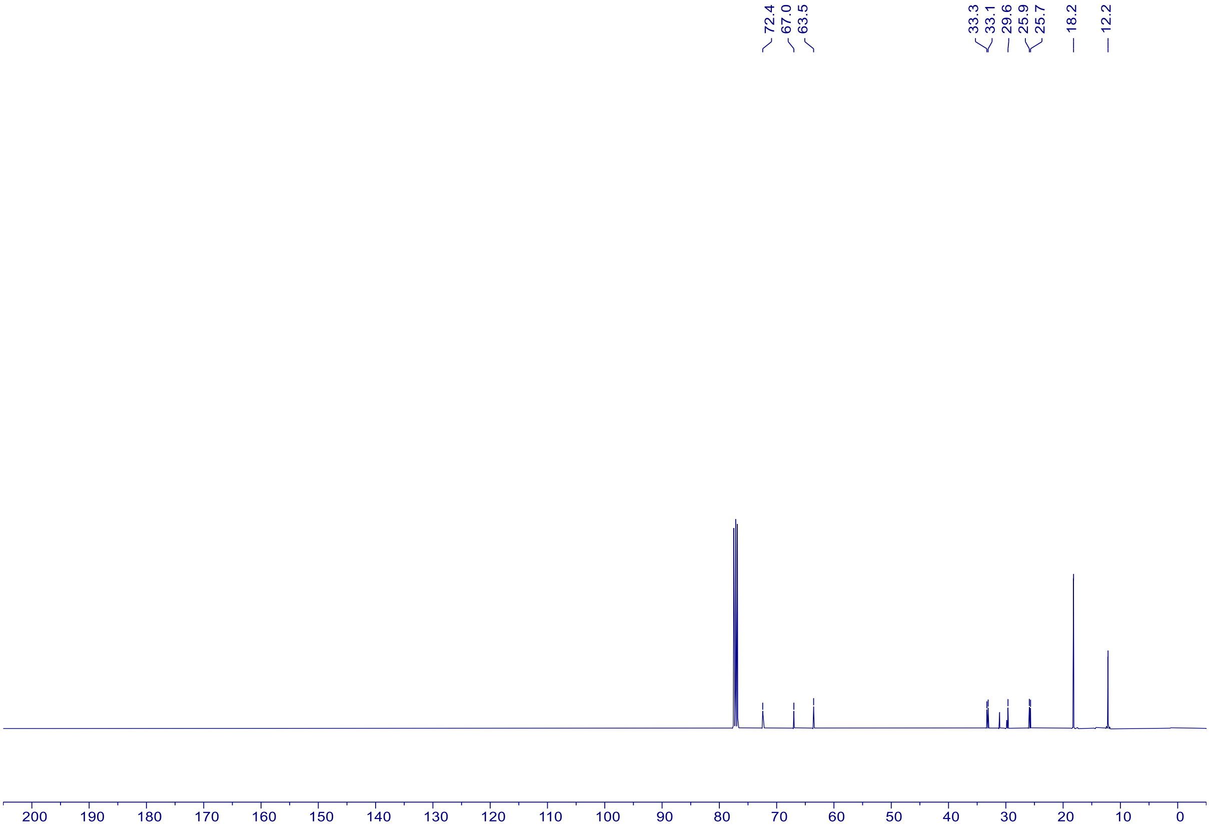

18a – ^{1}H NMR (CDCl$_3$, 400 MHz)

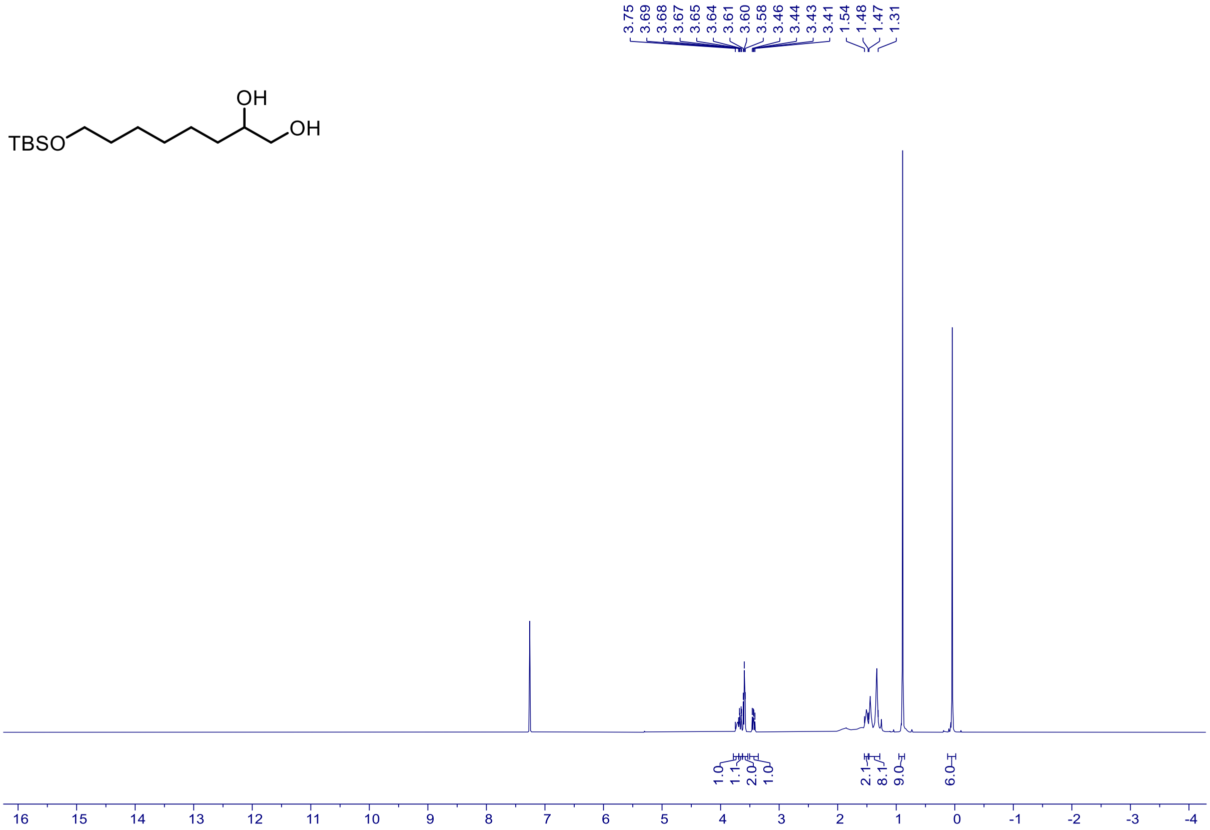

18a – ^{13}C NMR (CDCl$_3$, 101 MHz)

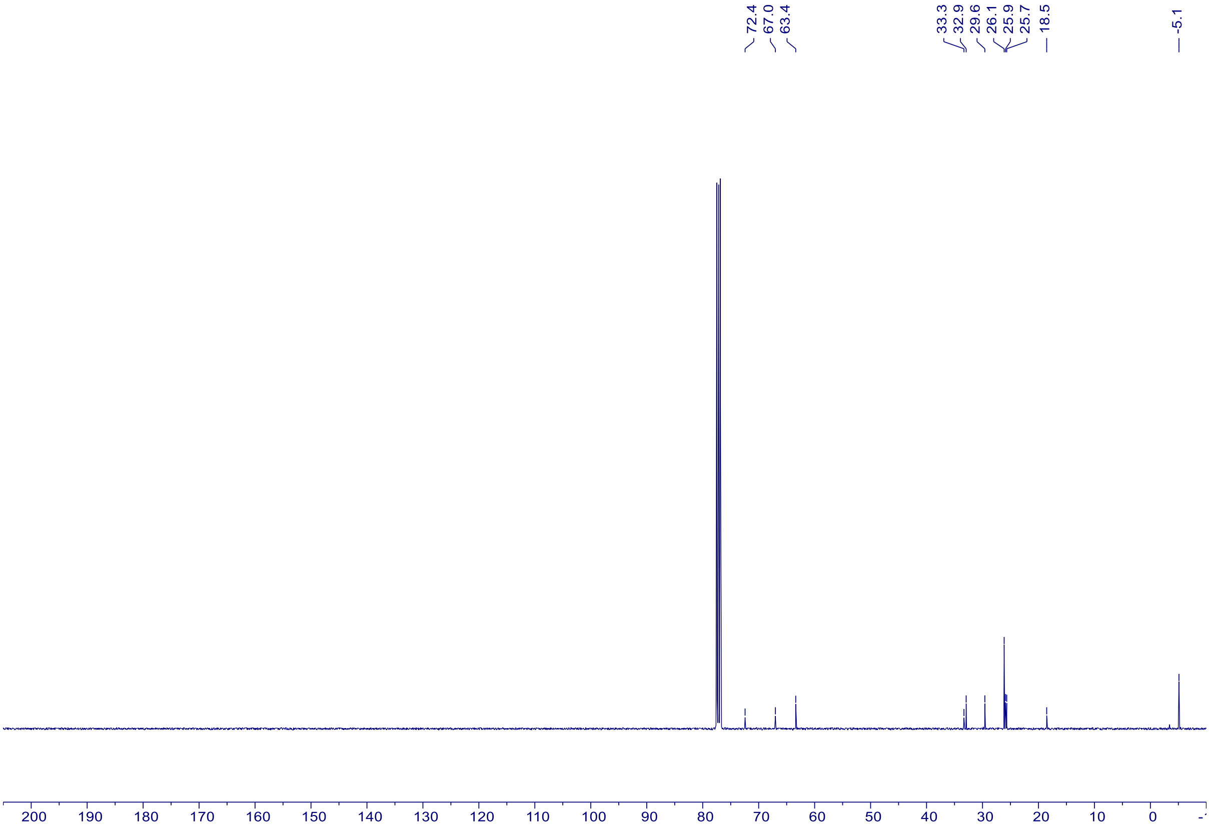

19a – ^{1}H NMR ($CDCl_3$, 400 MHz)

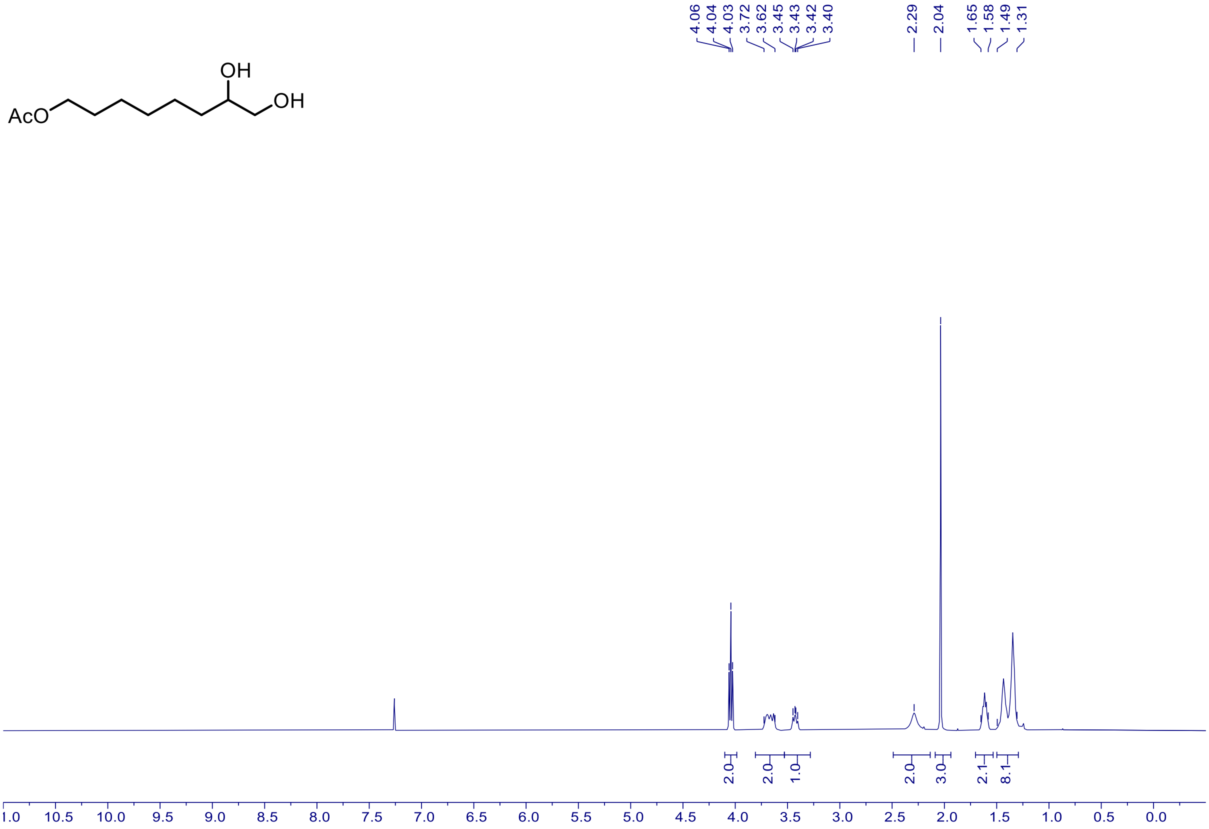

19a – ^{13}C NMR ($CDCl_3$, 101 MHz)

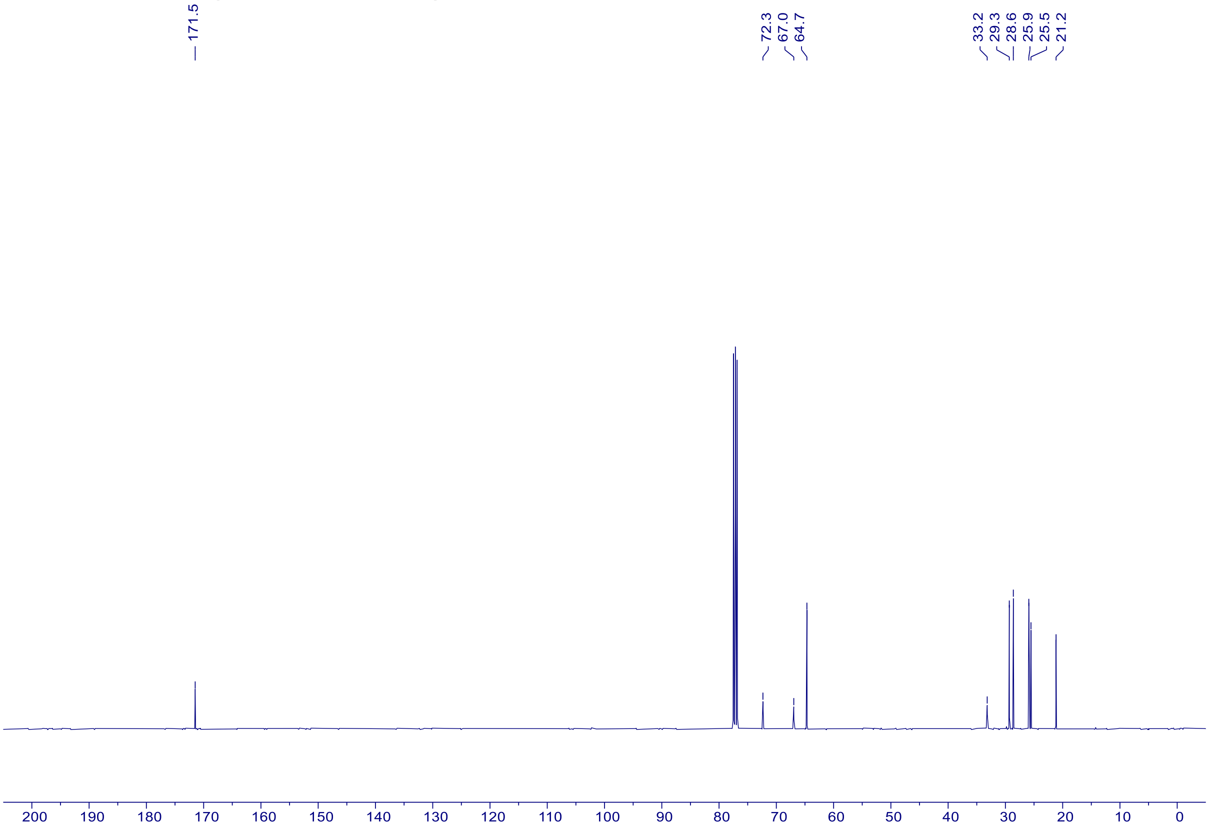

20a – ^{1}H NMR ($CDCl_3$, 400 MHz)

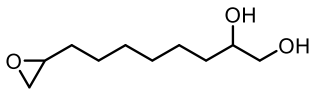

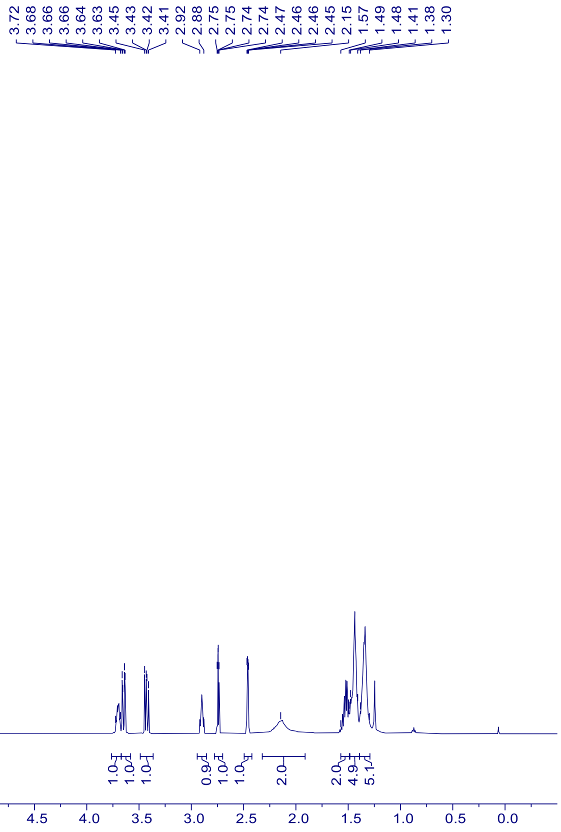

20a – ^{13}C NMR ($CDCl_3$, 101 MHz)

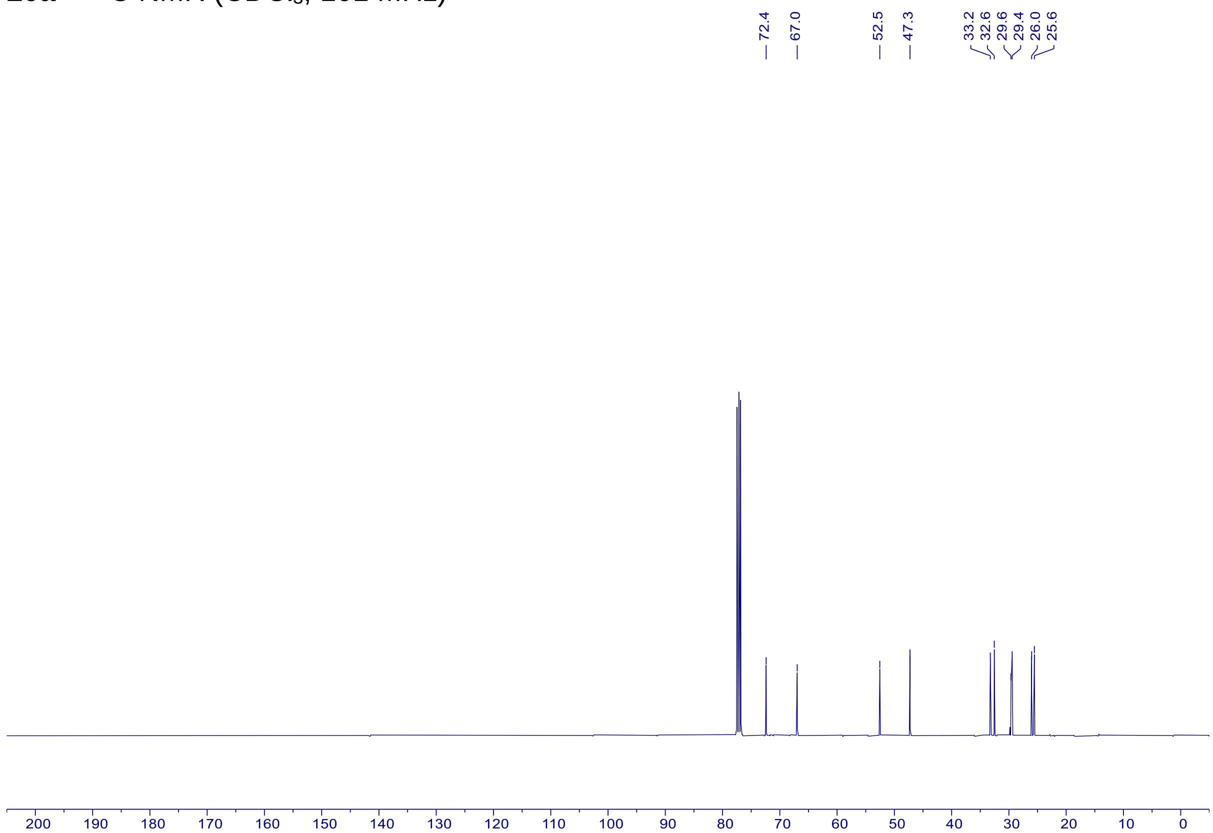

21a – ^{1}H NMR ($CDCl_3$, 400 MHz)

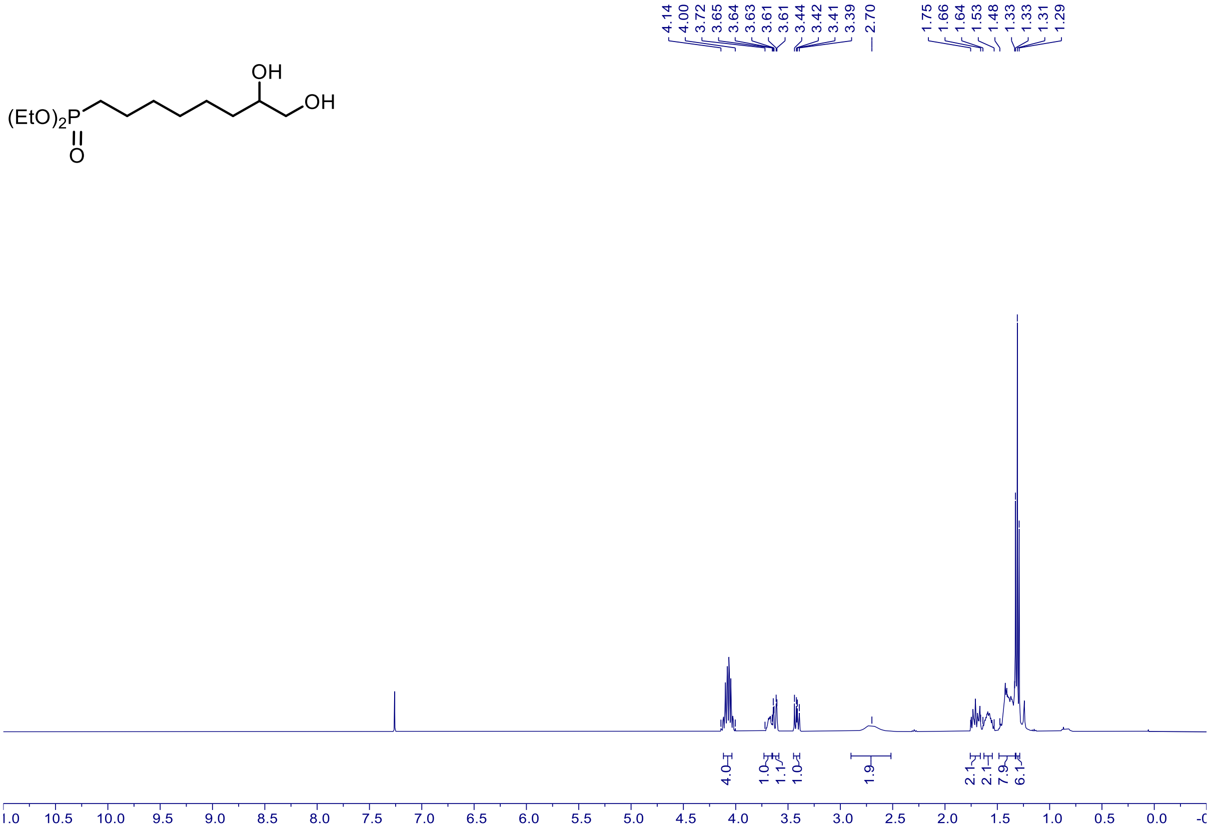

21a – ^{13}C NMR ($CDCl_3$, 101 MHz)

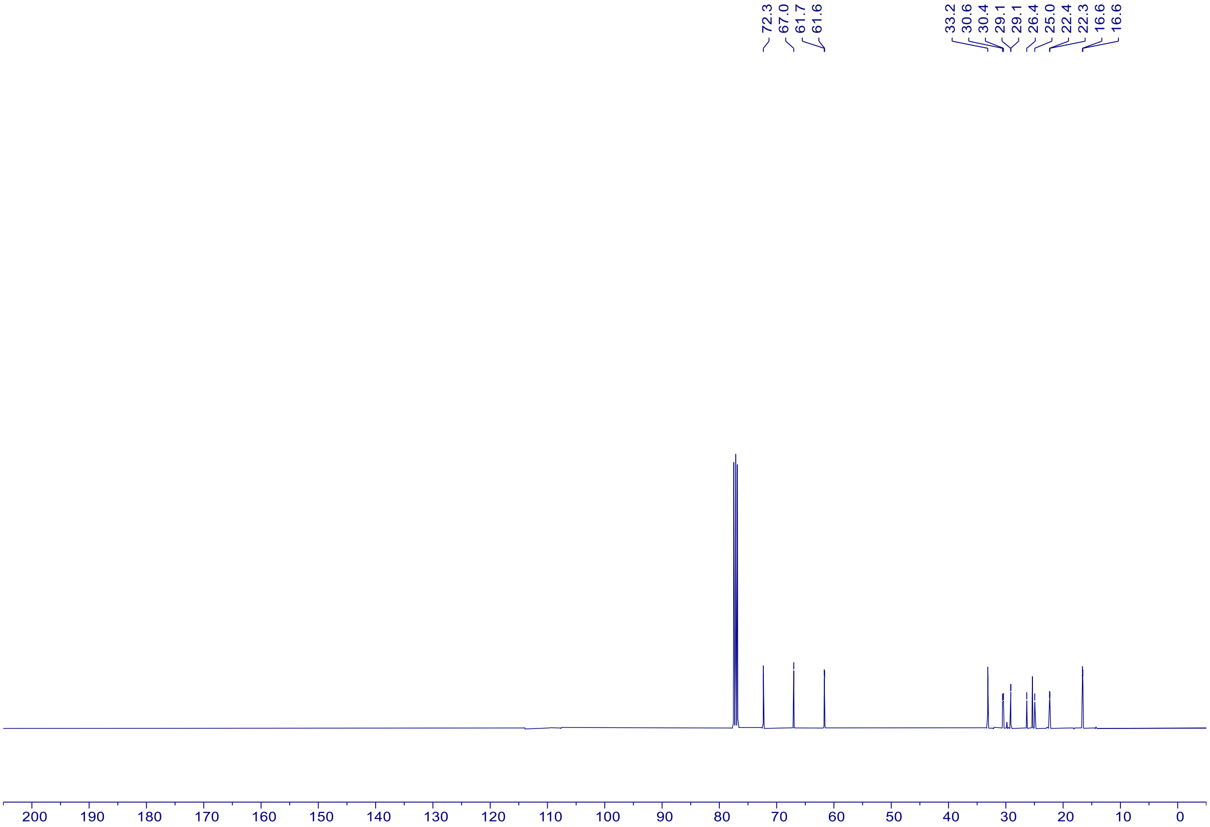

21a – ^{31}P NMR ($CDCl_3$, 162 MHz)

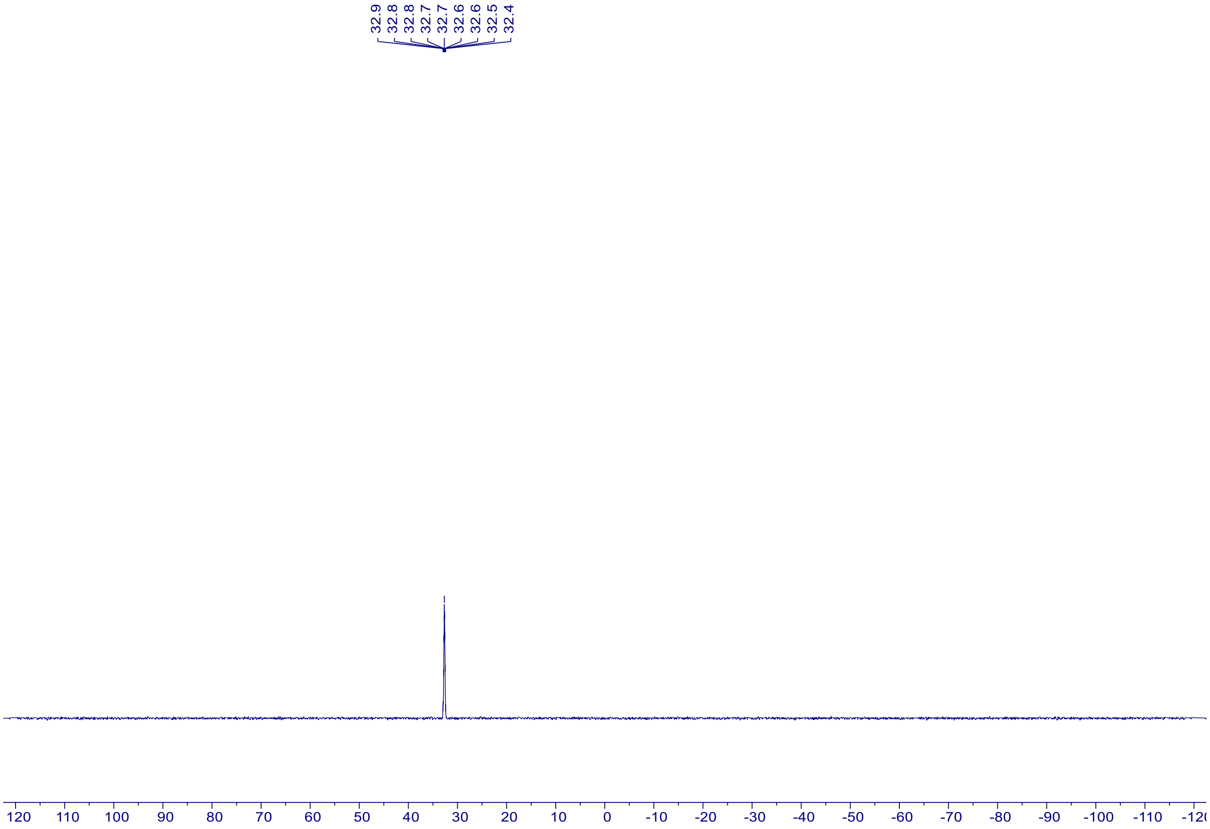

21a – ^{31}P {^{1}H} NMR ($CDCl_3$, 162 MHz)

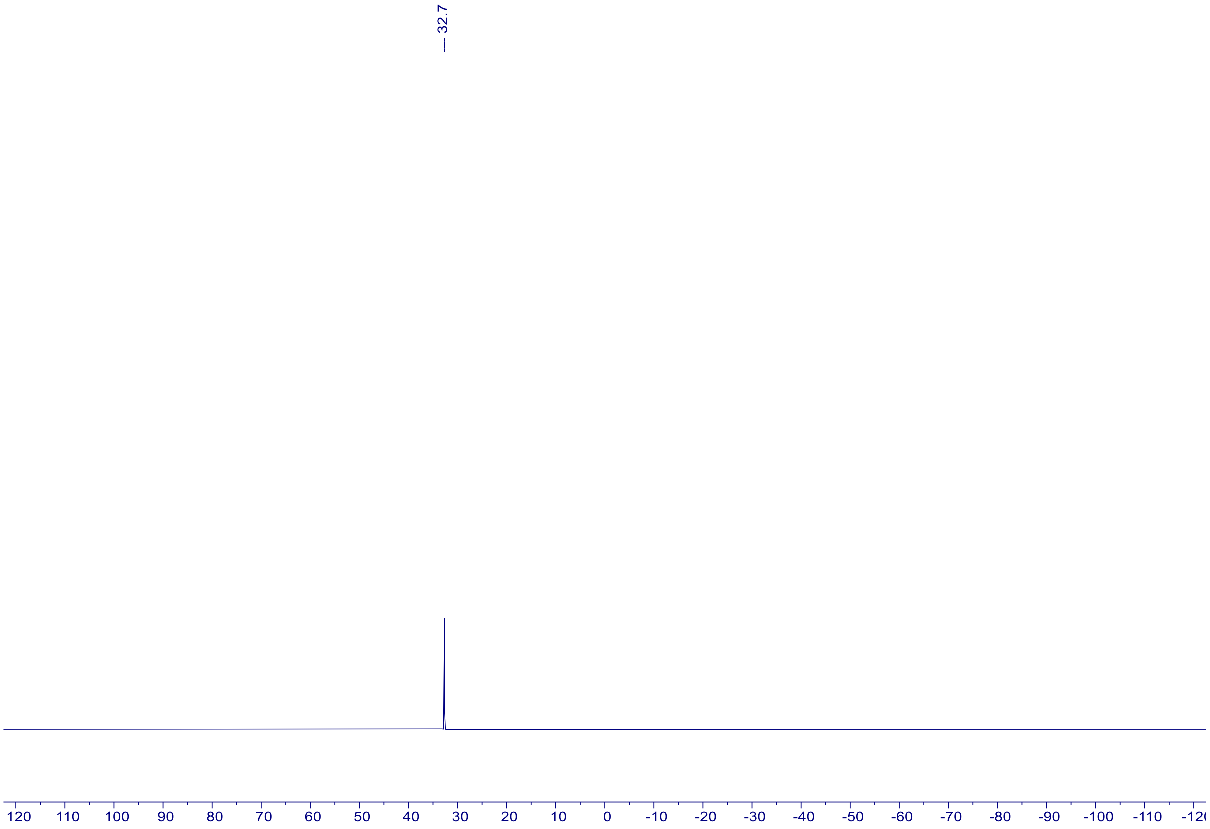

22a – ^{1}H NMR ($CDCl_3$, 400 MHz)

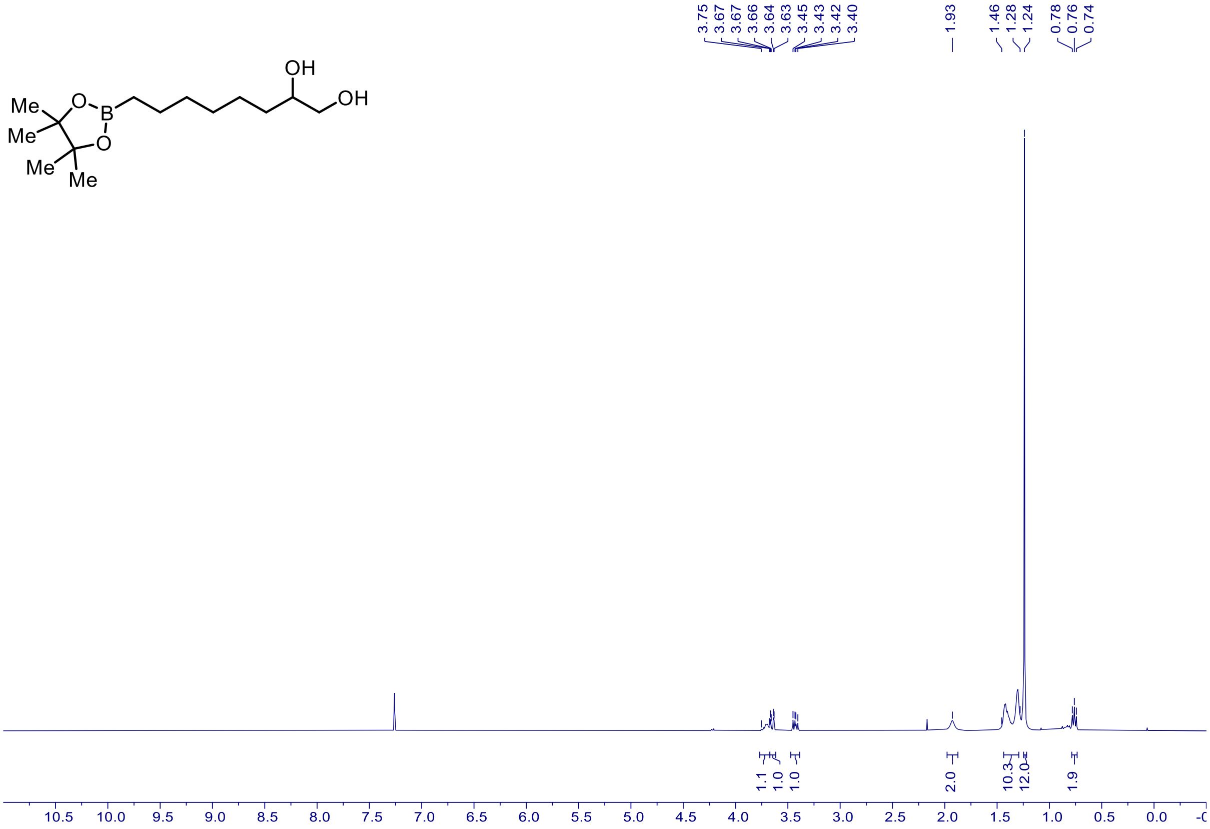

22a – ^{13}C NMR (CH_3OD, 101 MHz)

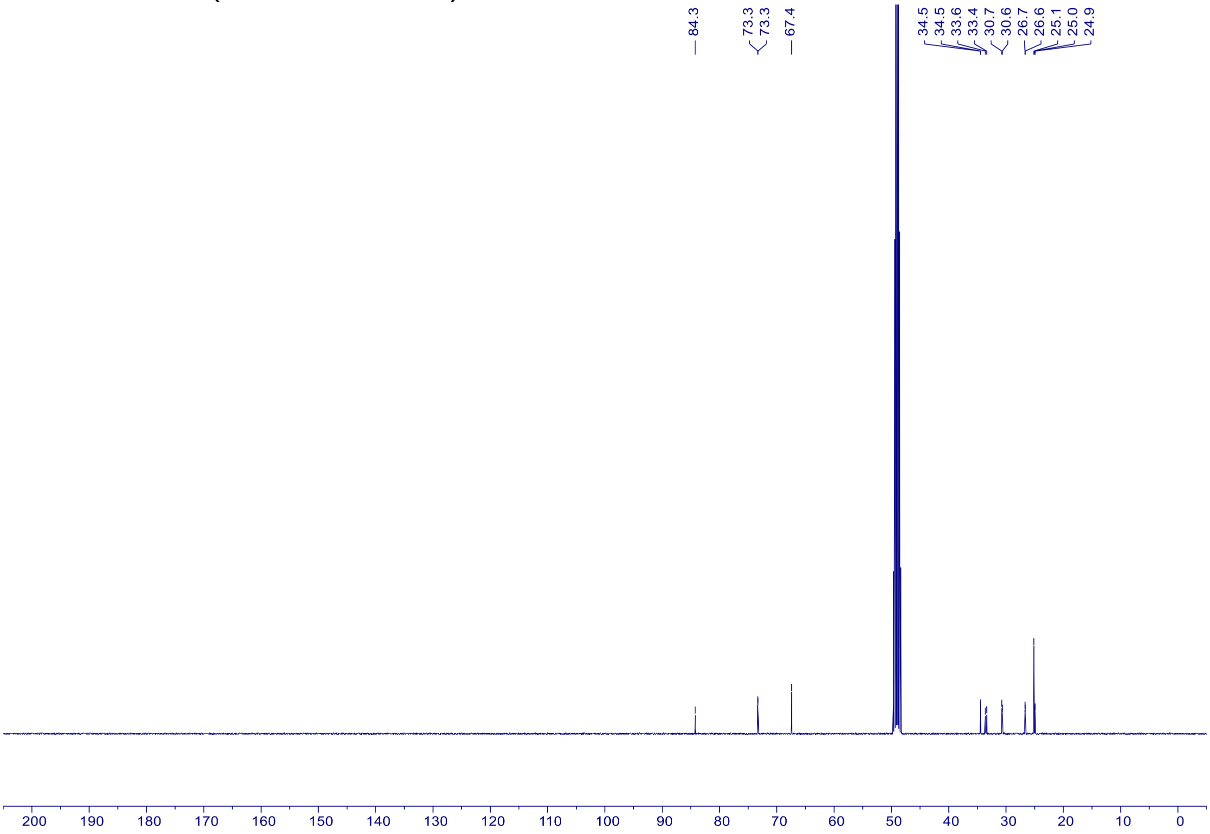

23a – ^{1}H NMR ($CDCl_3$, 400 MHz)

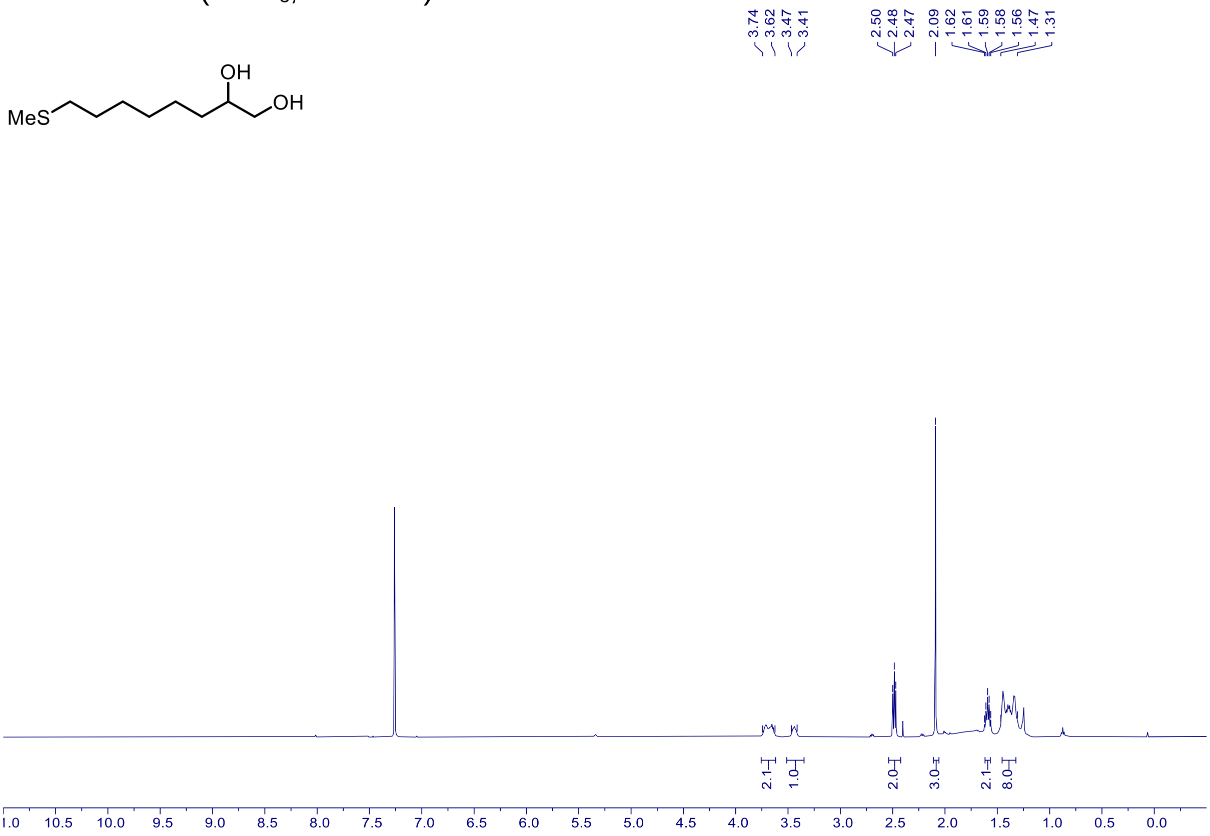

23a – ^{13}C NMR ($CDCl_3$, 101 MHz)

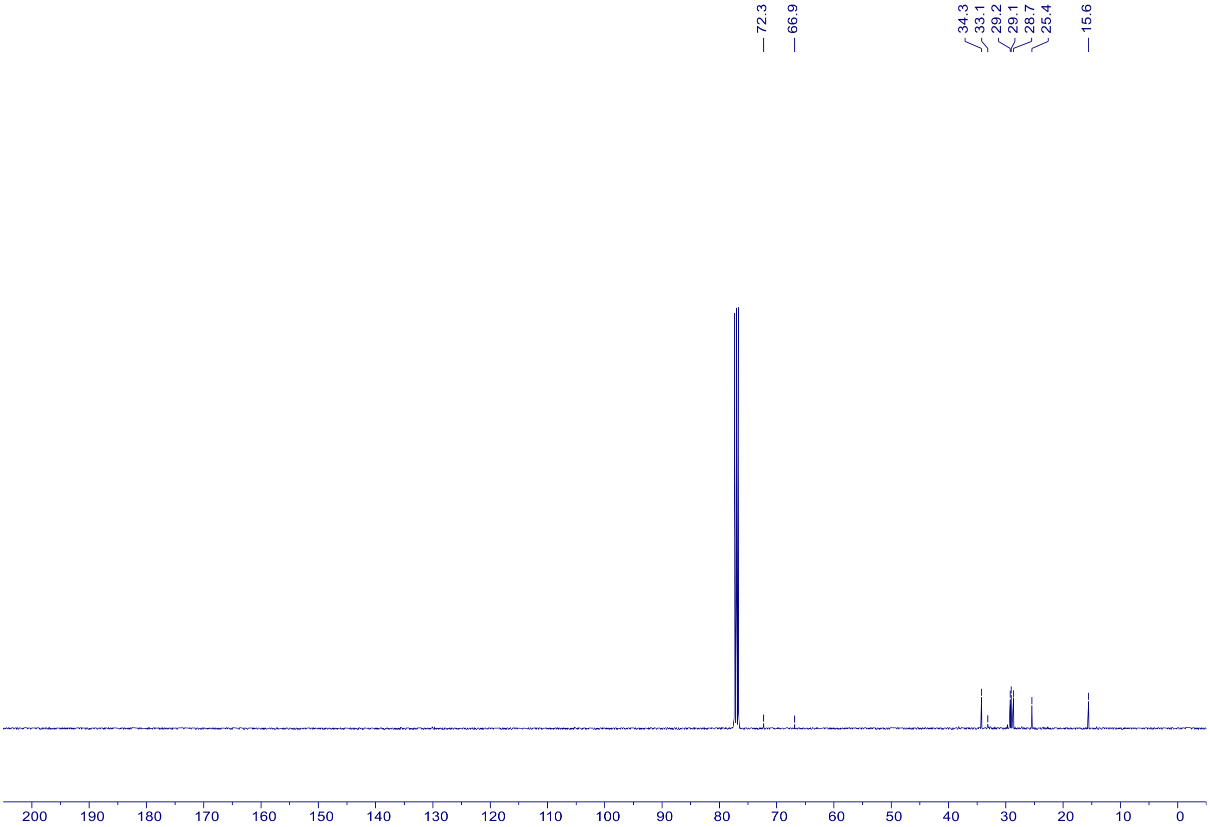

24a – ^{1}H NMR ($CDCl_3$, 400 MHz)

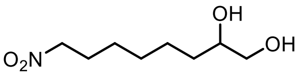

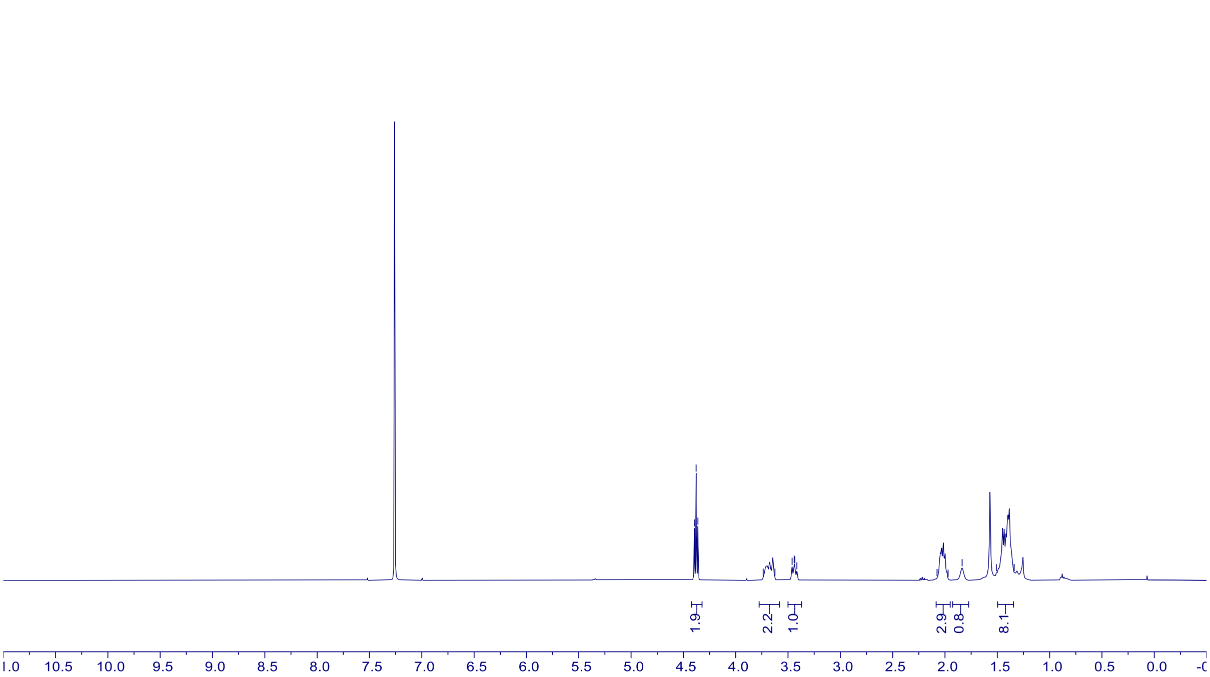

24a – ^{13}C NMR ($CDCl_3$, 101 MHz)

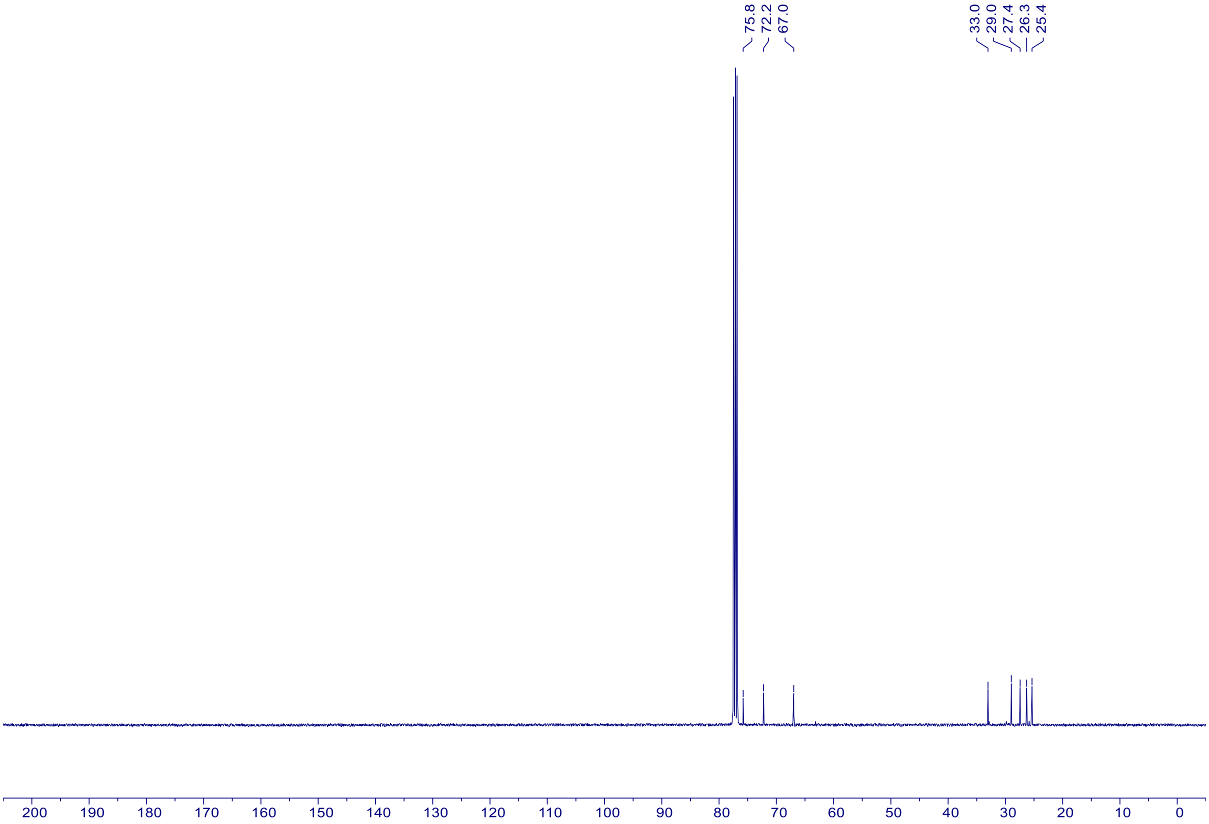

25a – ^{1}H NMR ($CDCl_3$, 400 MHz)

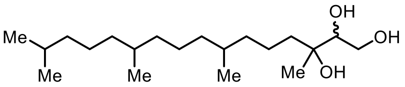

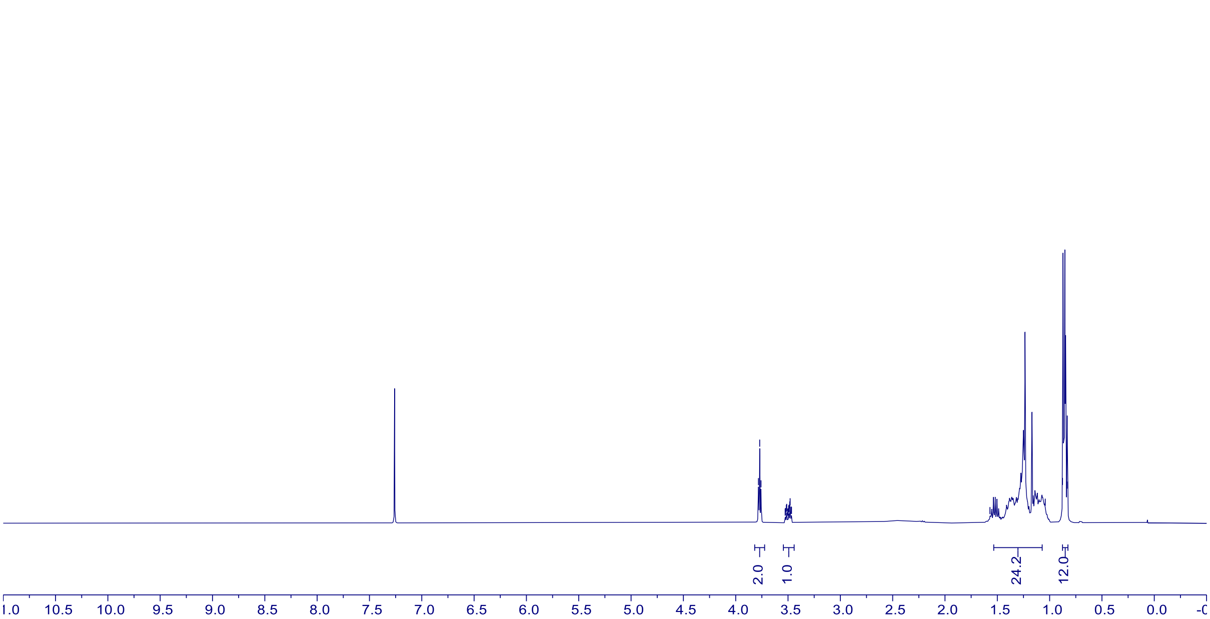

25a – ^{13}C NMR ($CDCl_3$, 101 MHz)

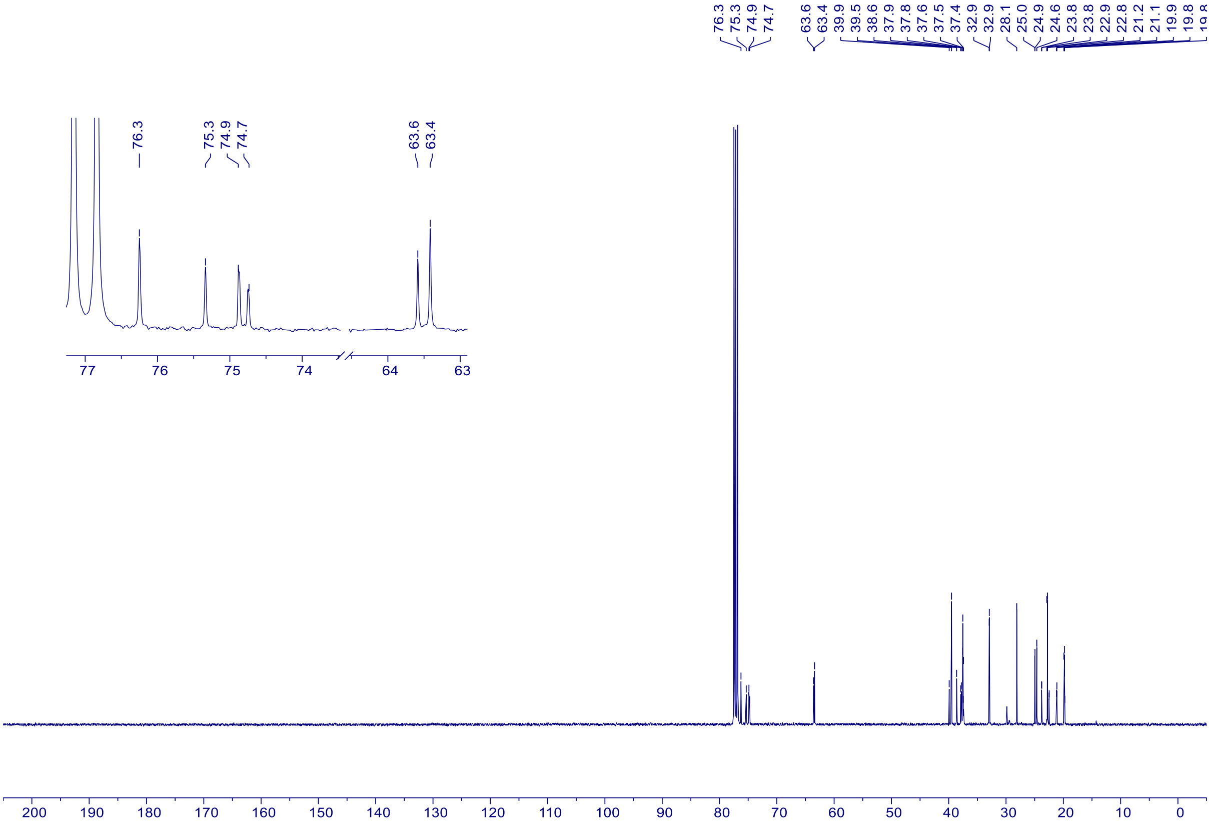

26a – ^{1}H NMR ($CDCl_3$, 400 MHz)

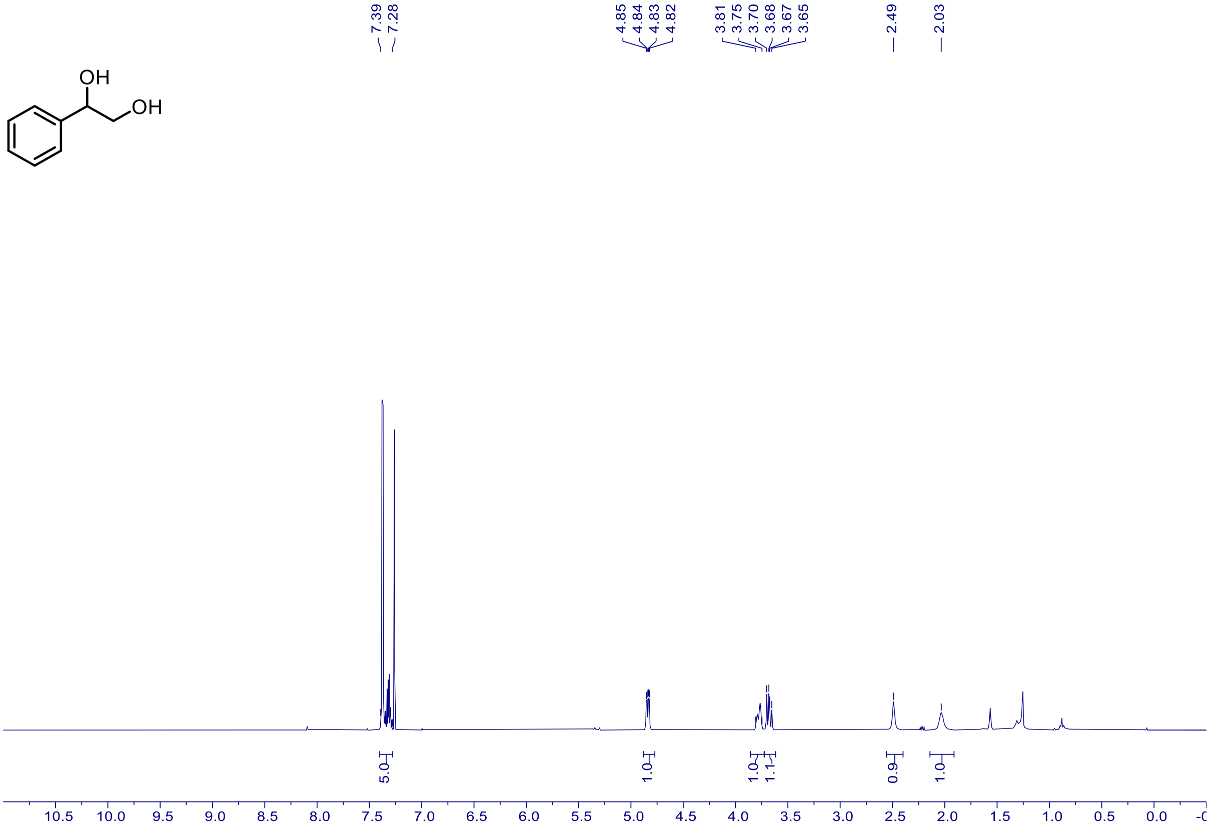

26a – ^{13}C NMR ($CDCl_3$, 101 MHz)

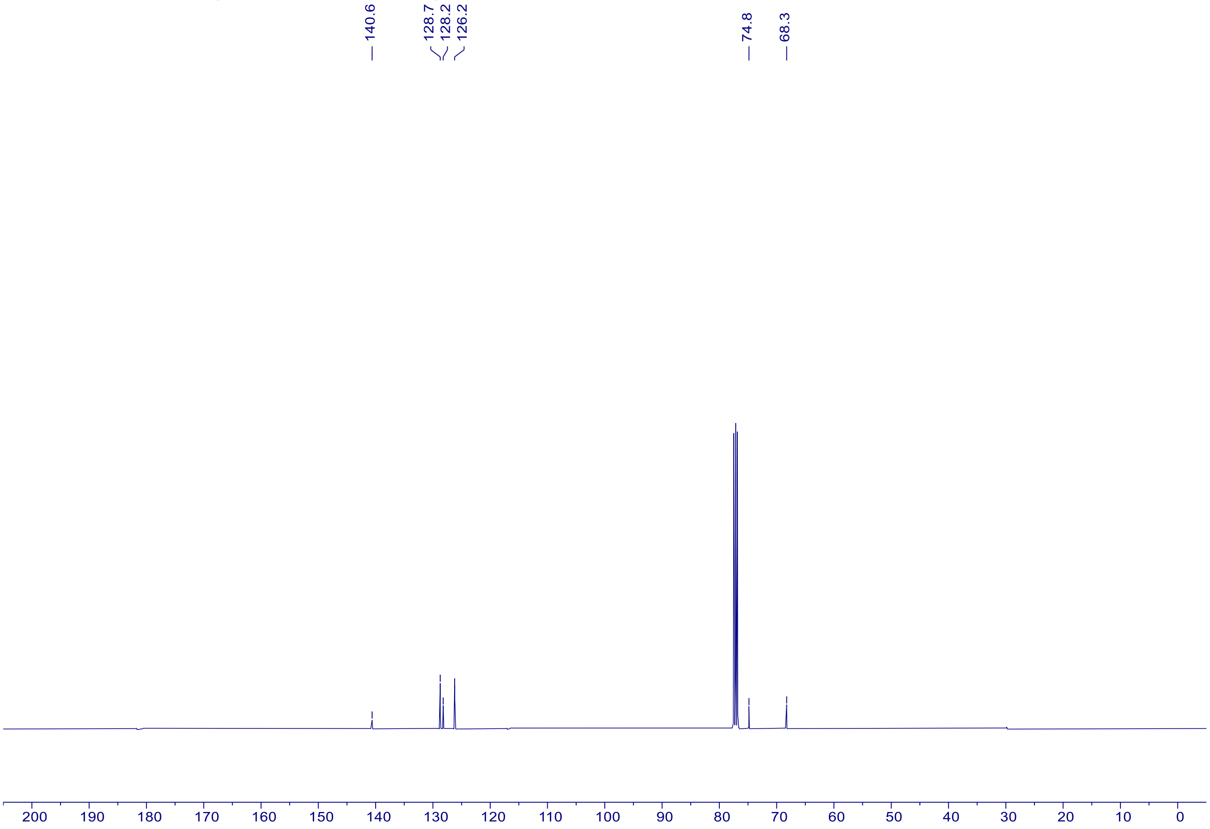

27a – ^{1}H NMR ($CDCl_3$, 400 MHz)

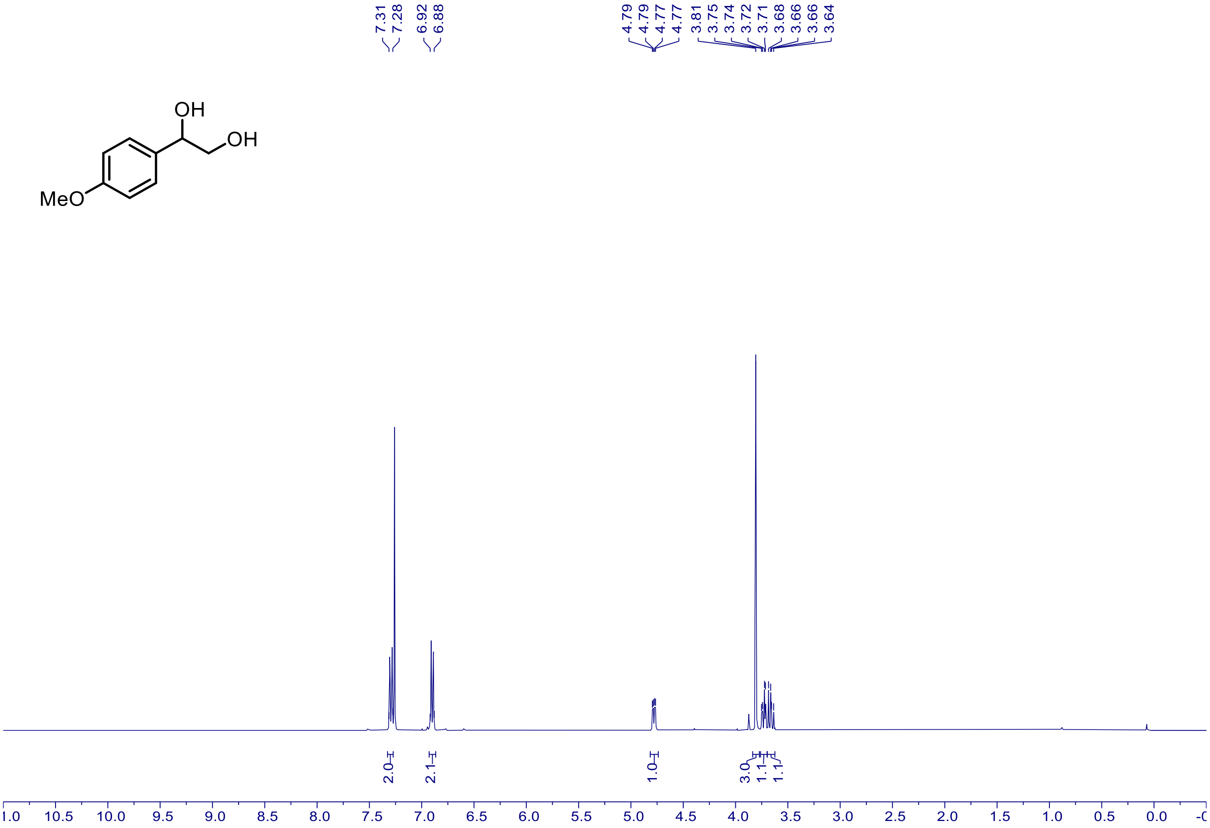

27a – ^{13}C NMR ($CDCl_3$, 101 MHz)

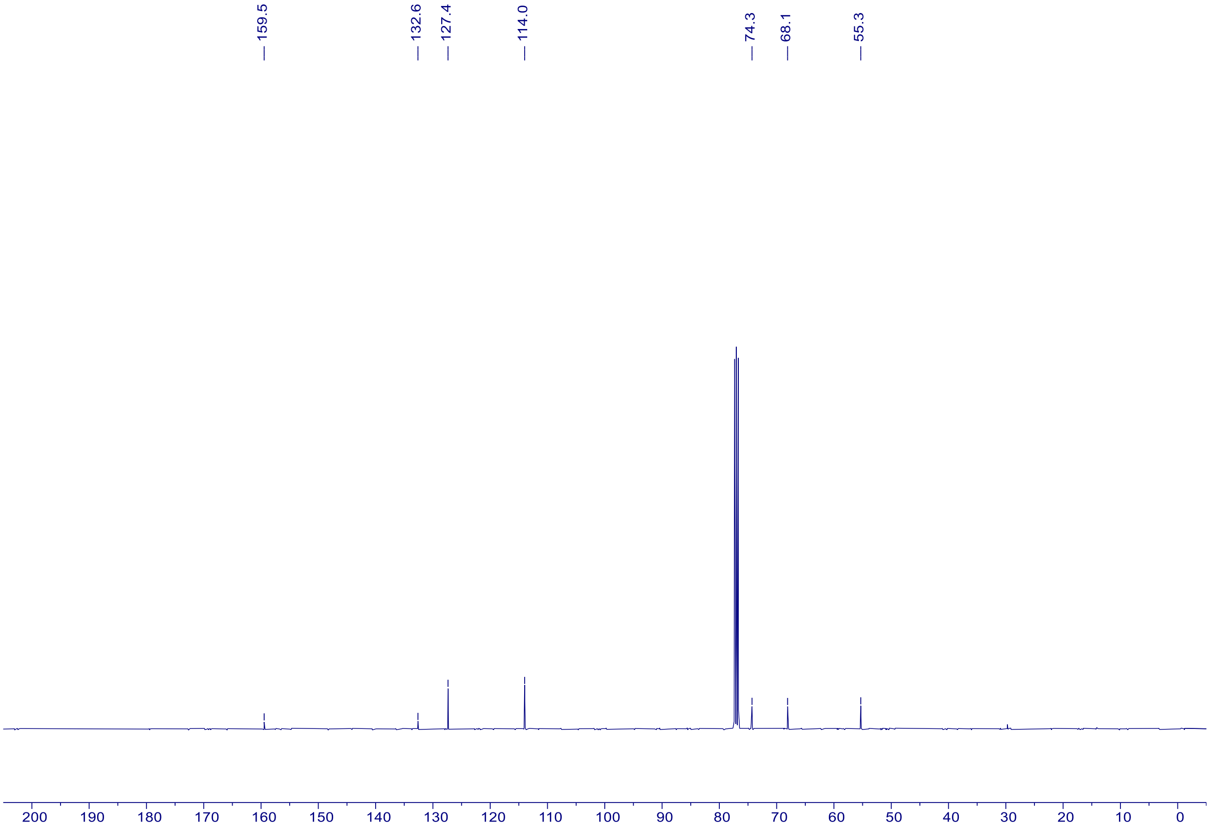

28a – ^{1}H NMR (CD_3CN, 400 MHz)

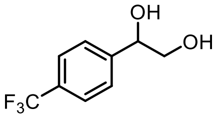

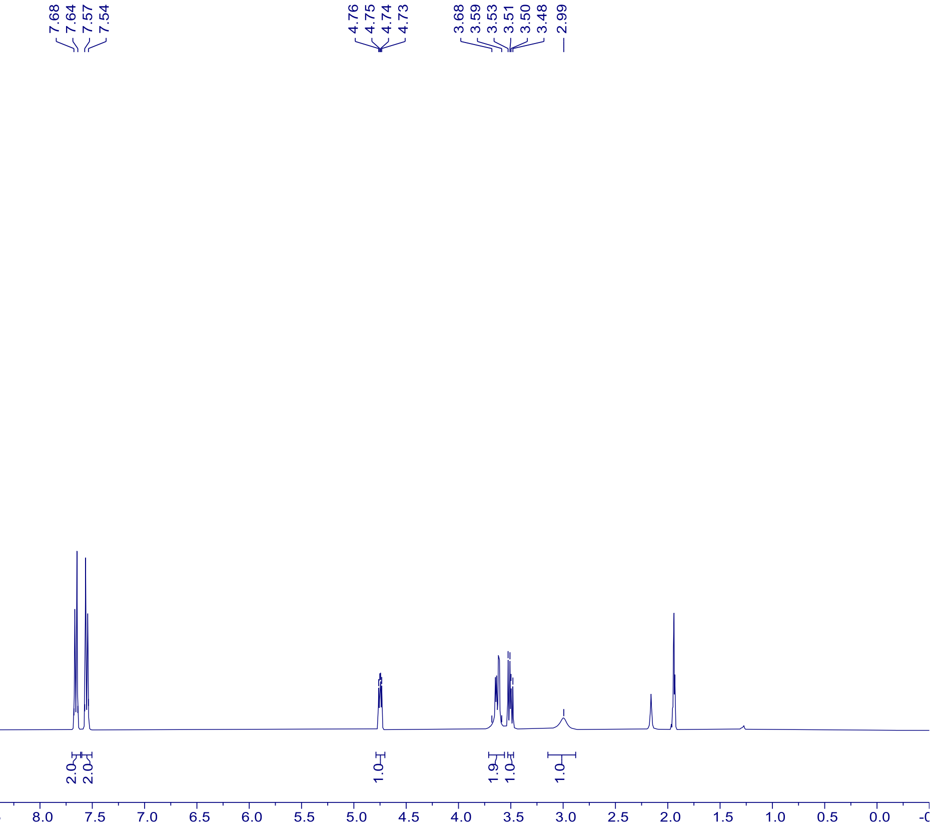

28a – ^{13}C NMR ($CDCl_3$, 101 MHz)

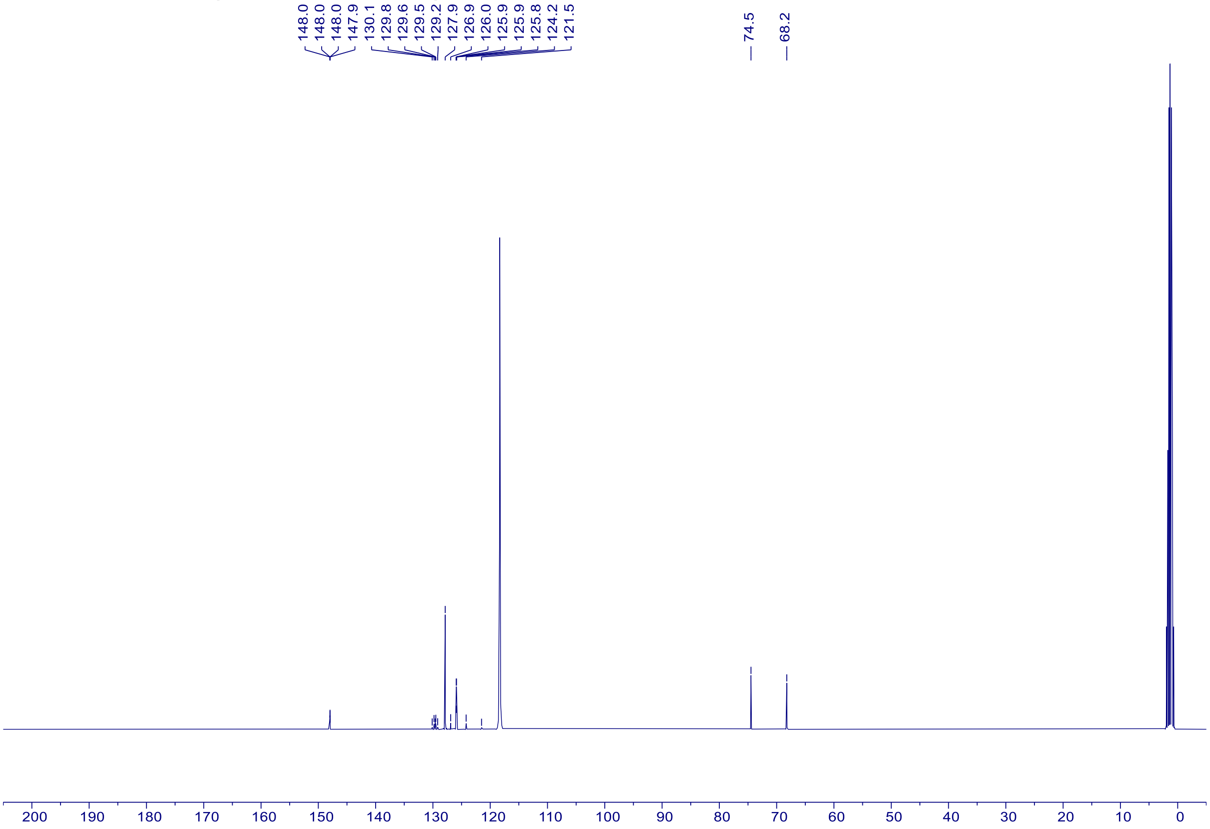

28a – ^{19}F NMR (CD_3CN, 471 MHz)

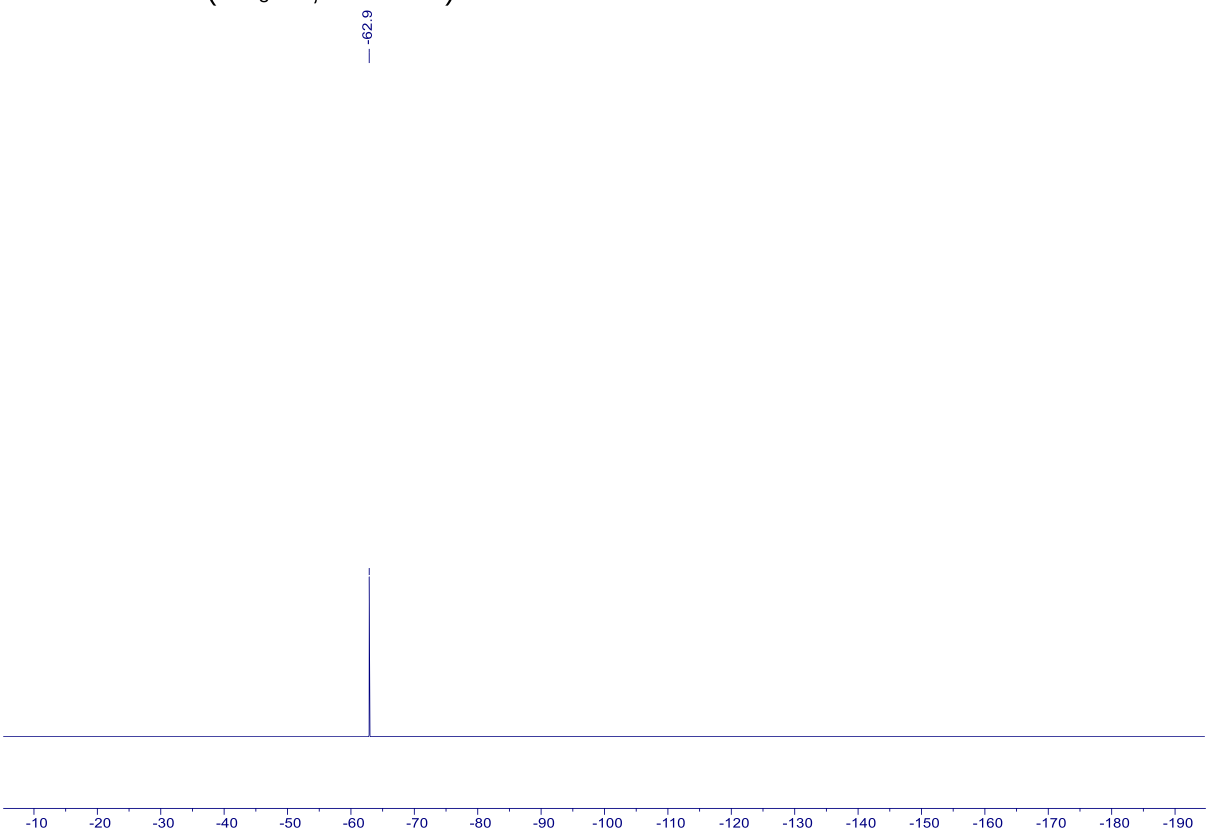

29a – ^{1}H NMR ($CDCl_3$, 400 MHz)

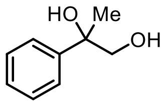

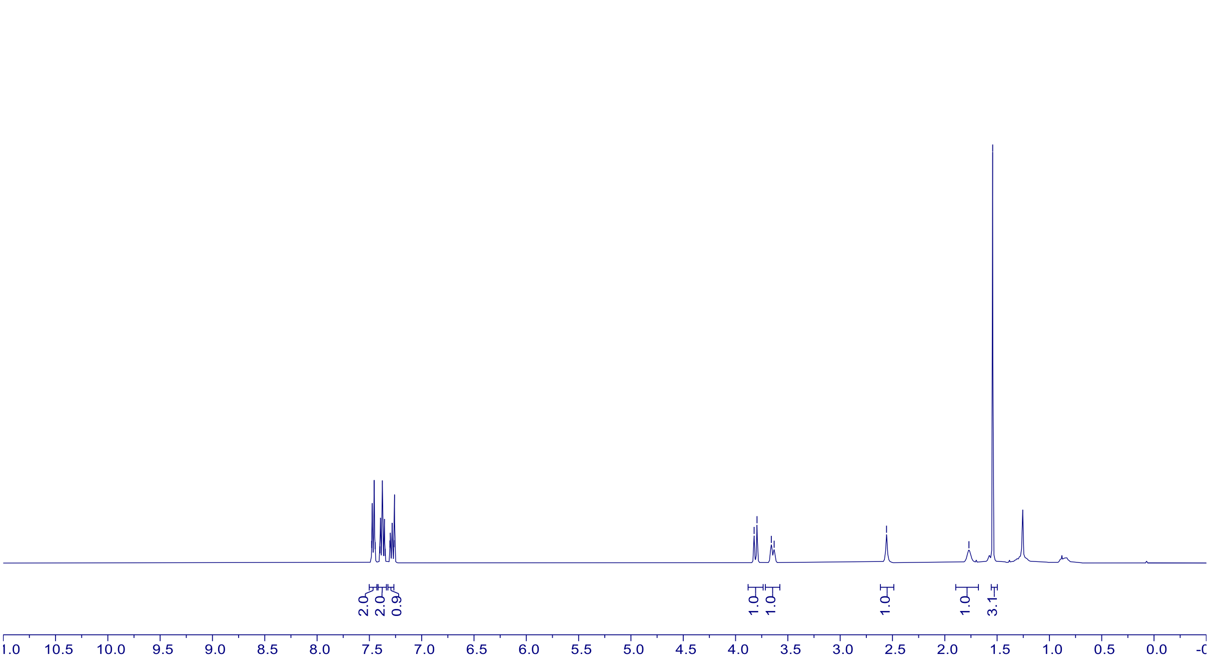

29a – ^{13}C NMR ($CDCl_3$, 101 MHz)

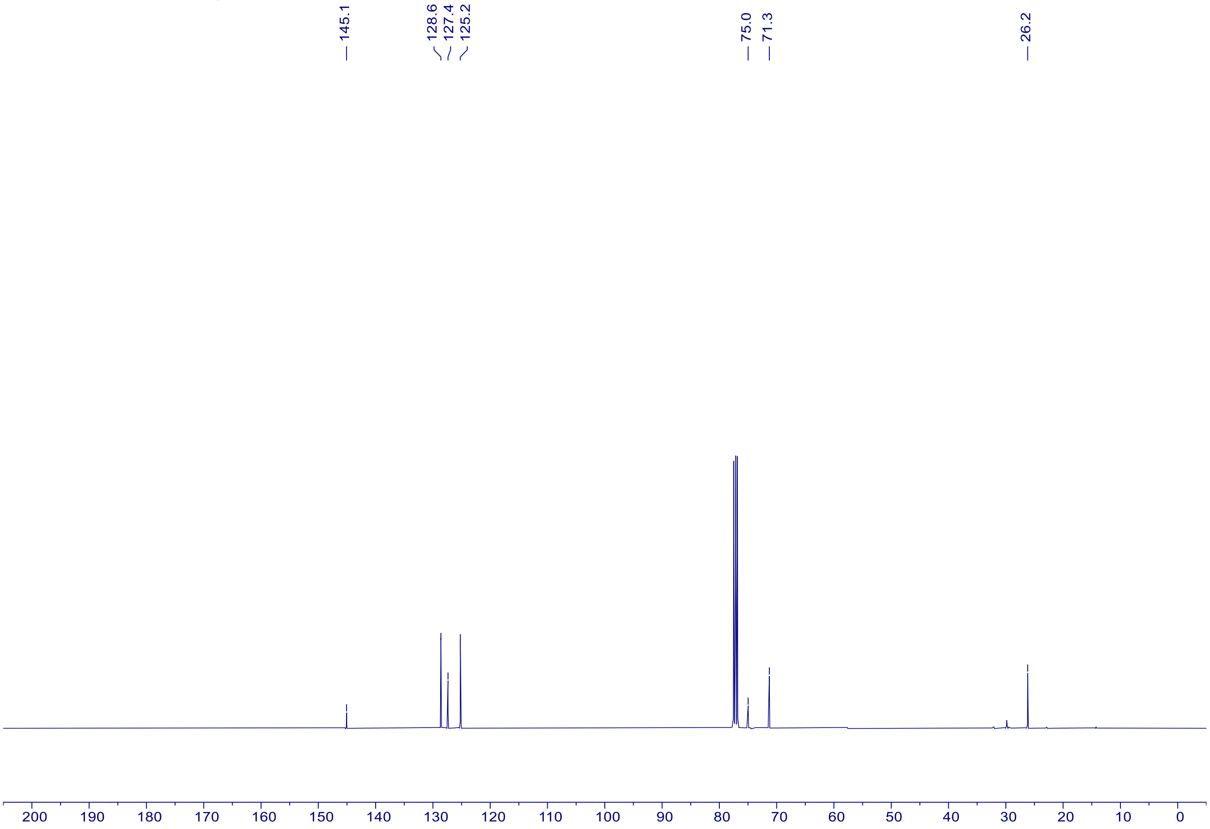

30a – ^{1}H NMR ($CDCl_3$, 400 MHz)

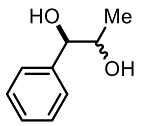

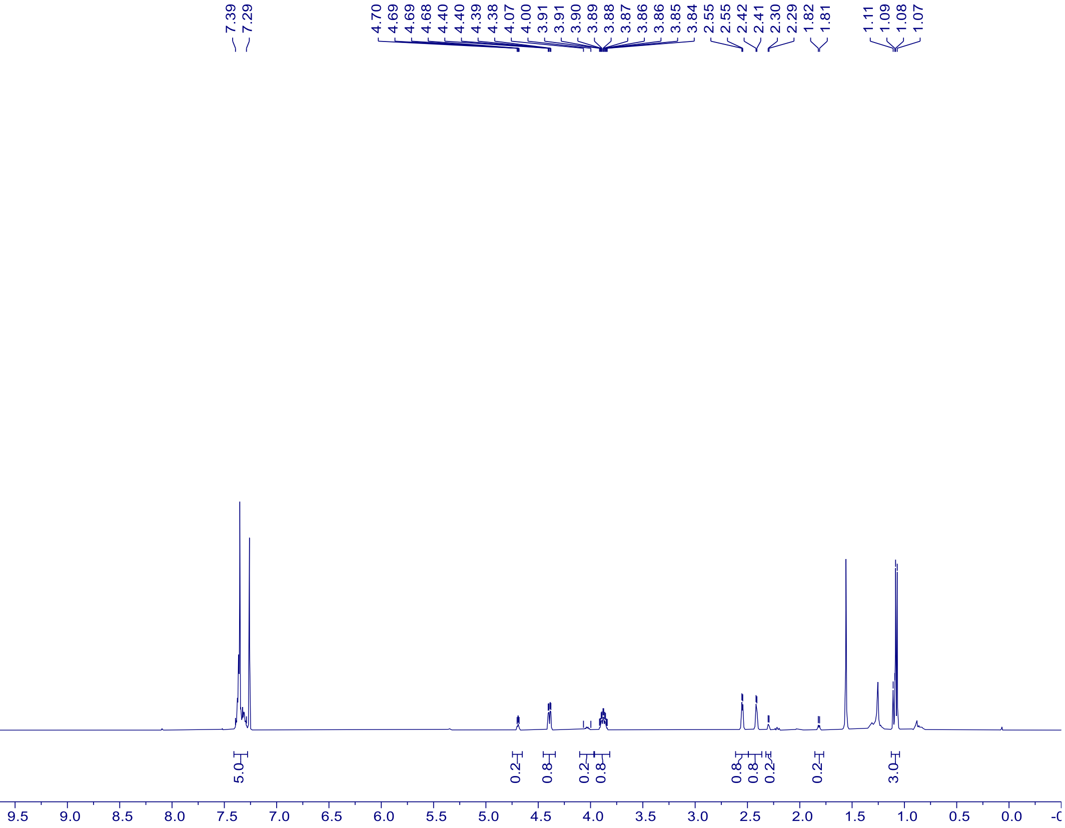

30a – ^{13}C NMR (CD_3CN, 101 MHz)

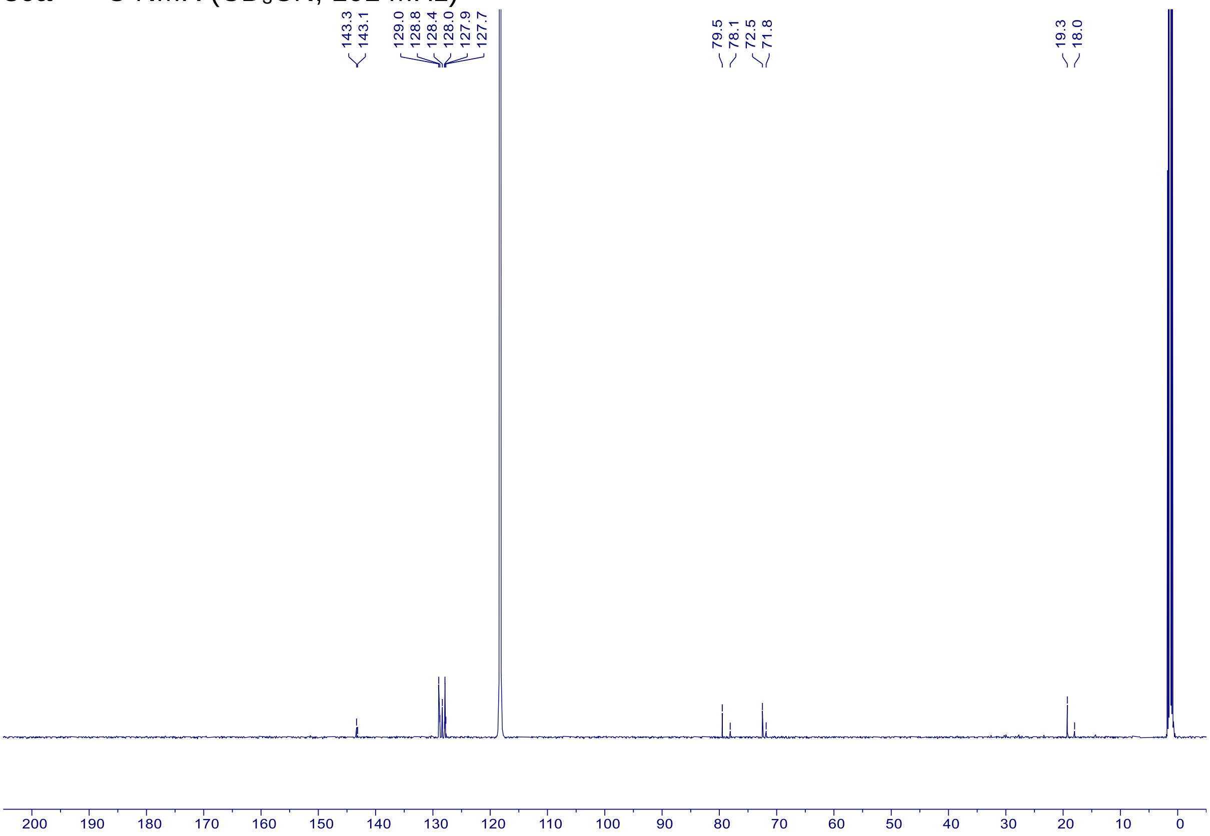

31a – ^{1}H NMR ($CDCl_3$, 400 MHz)

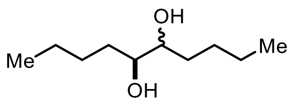

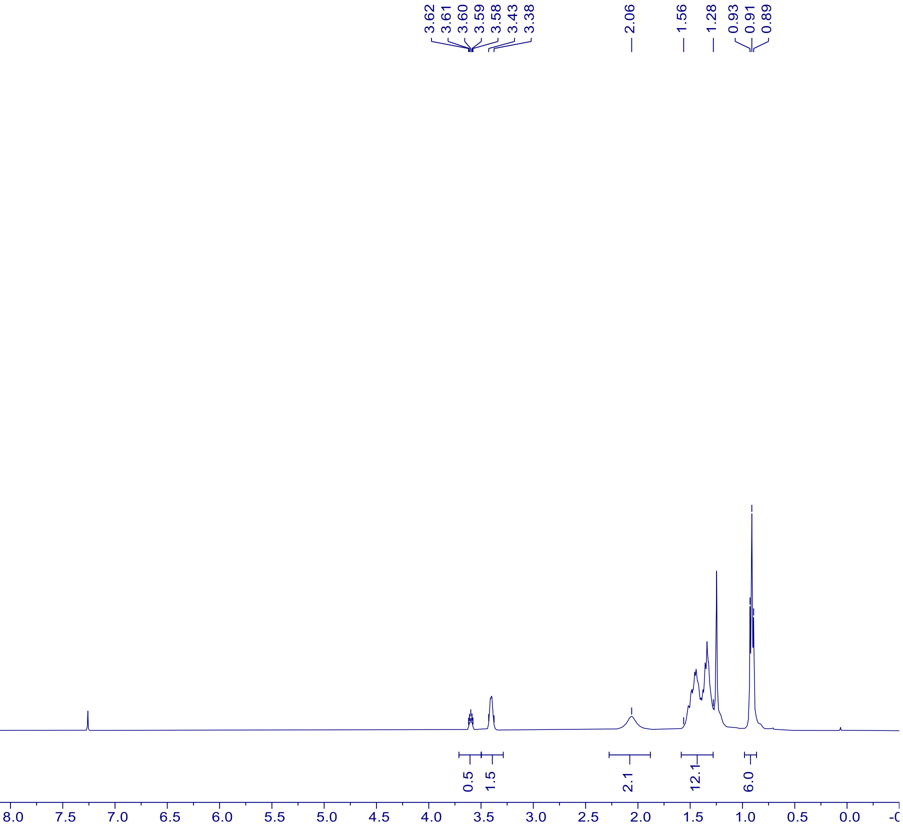

31a – ^{13}C NMR ($CDCl_3$, 101 MHz)

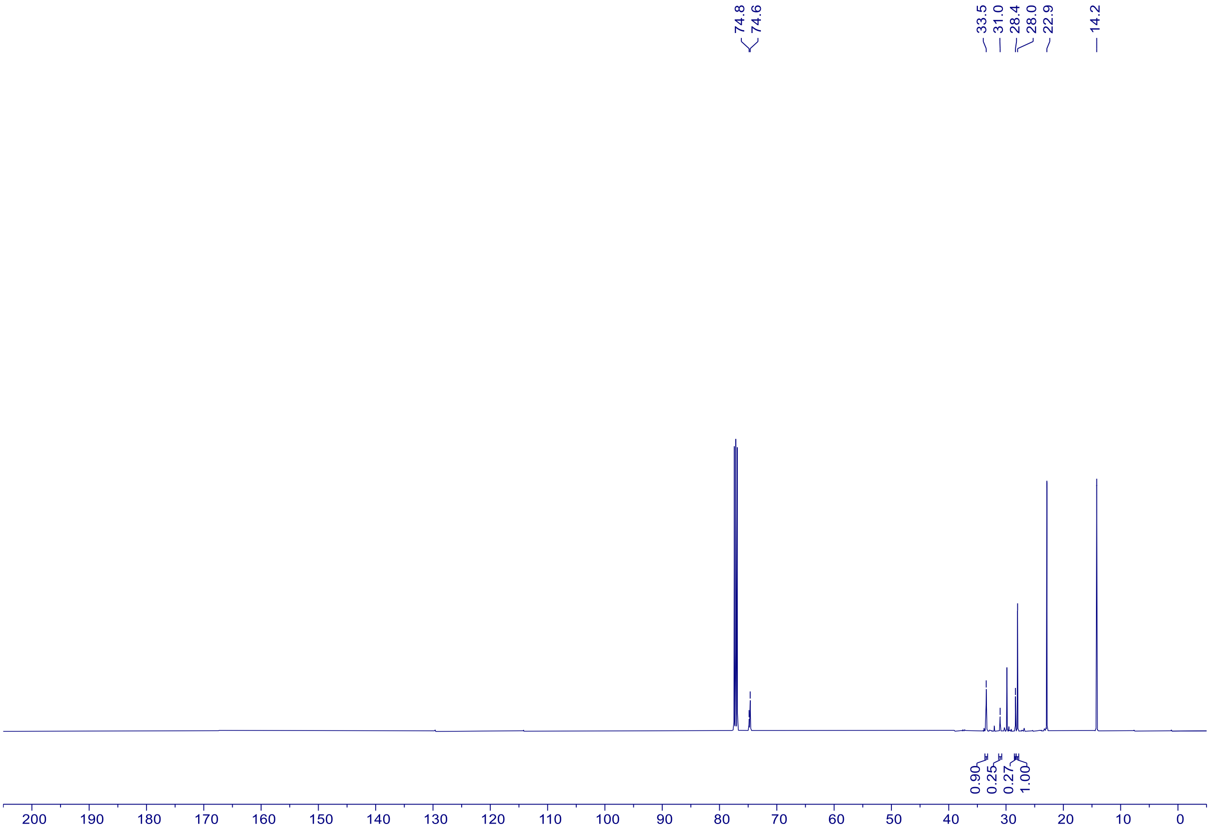

32a – ^{1}H NMR ($CDCl_3$, 500 MHz)

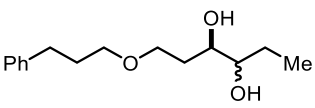

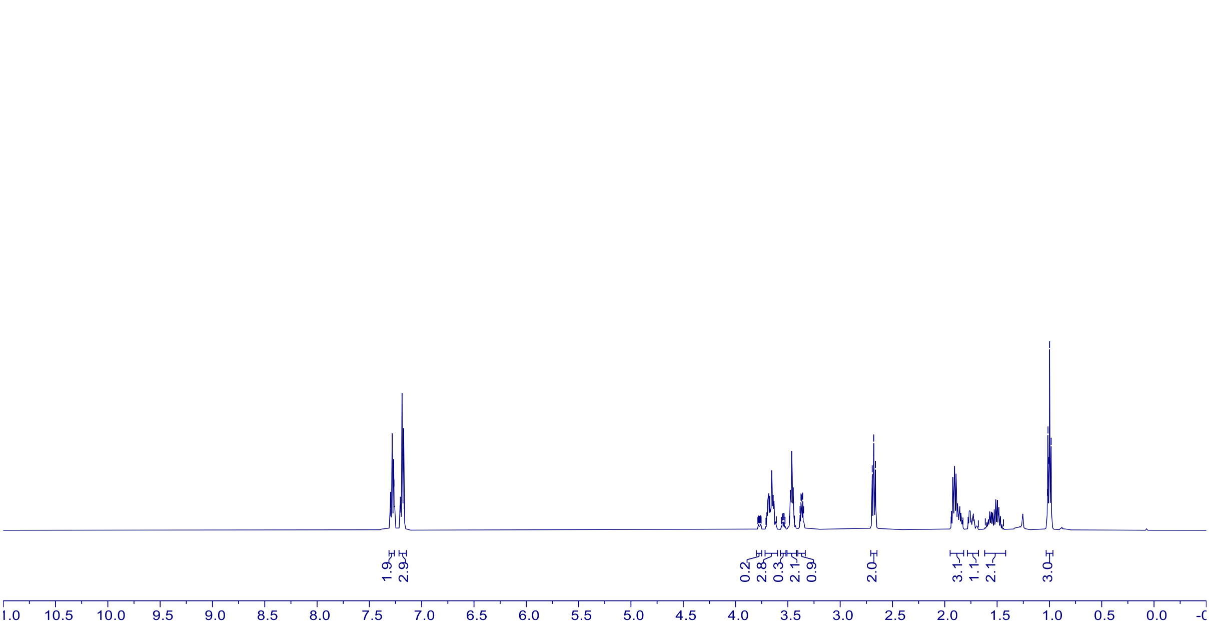

32a – ^{13}C NMR ($CDCl_3$, 126 MHz)

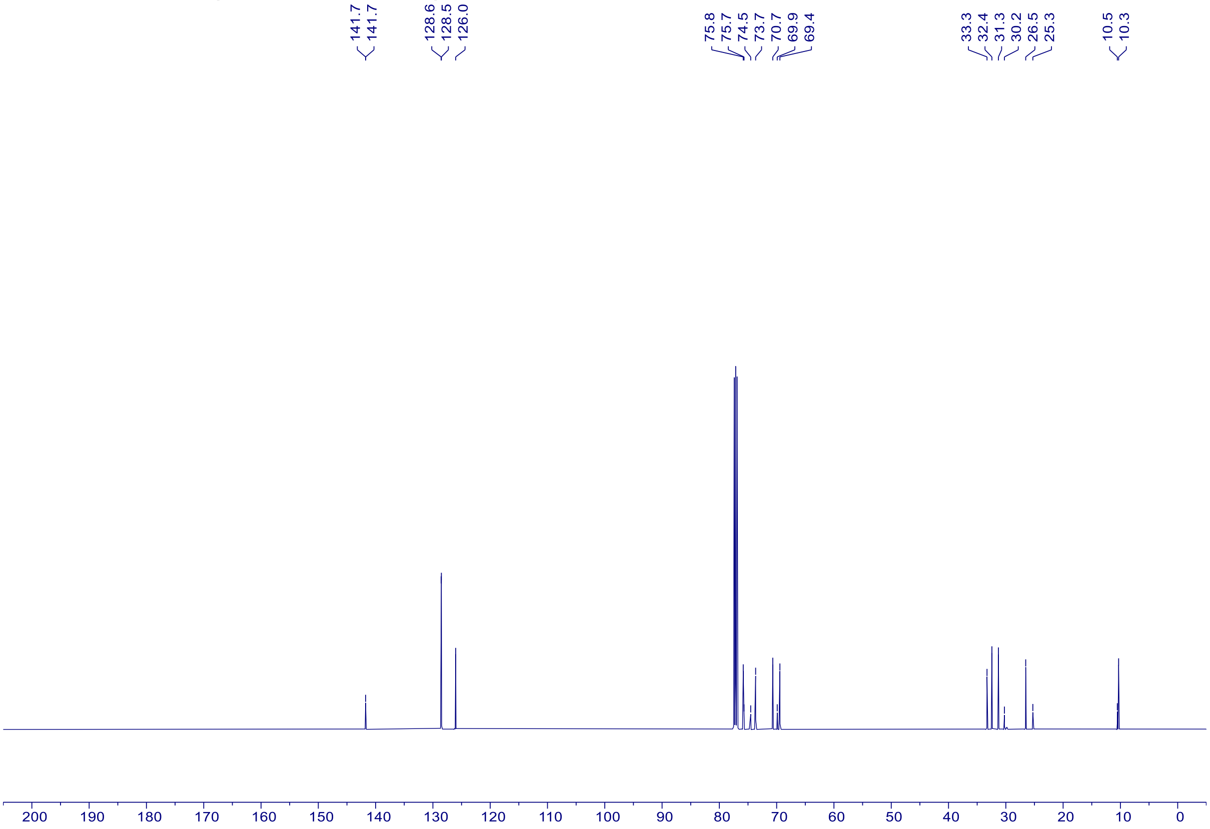

33a – ^{1}H NMR ($CDCl_3$, 500 MHz)

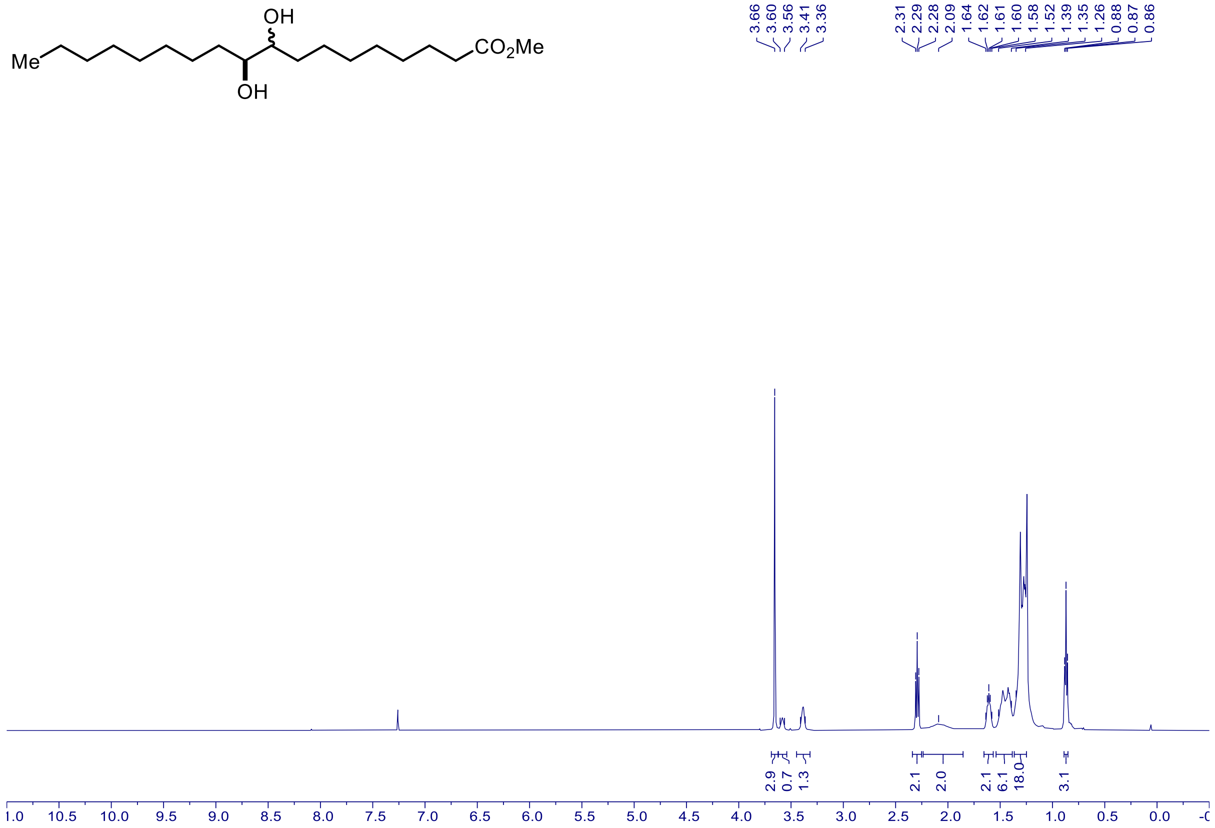

33a – ^{13}C NMR ($CDCl_3$, 126 MHz)

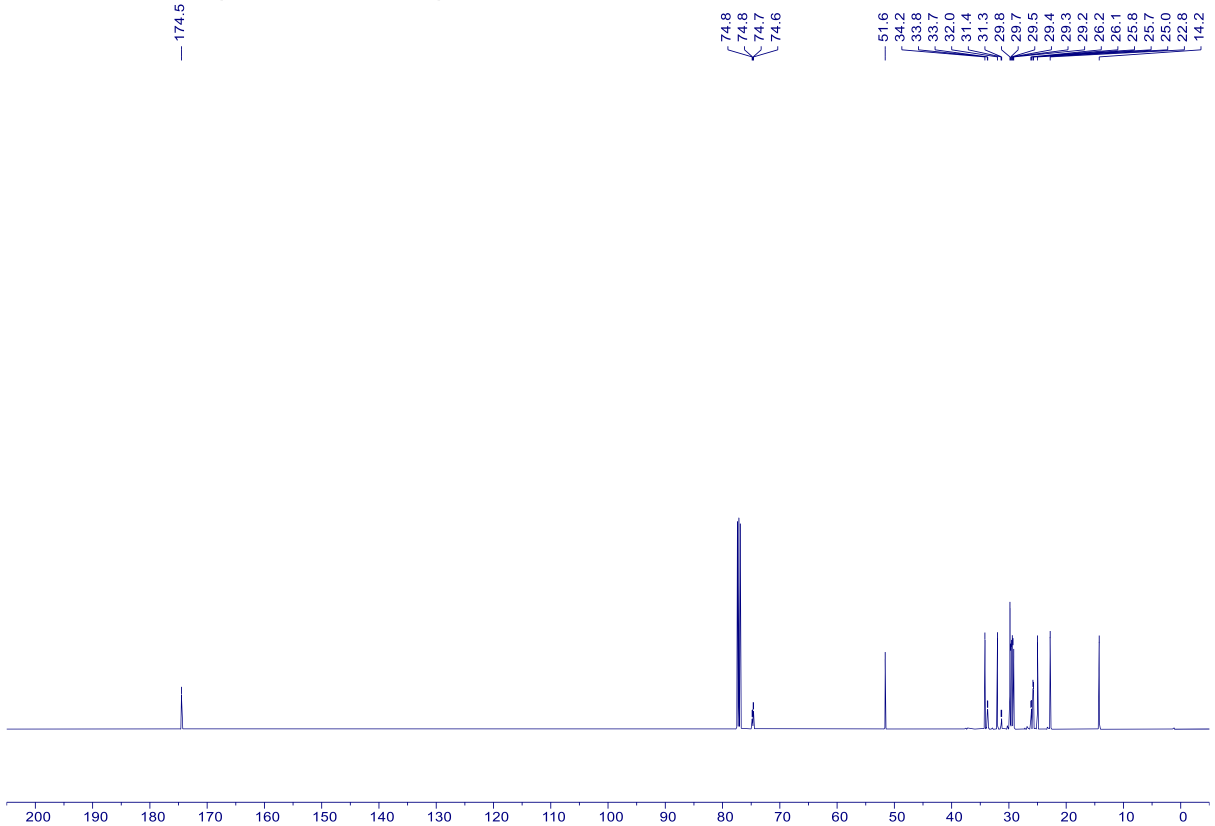

34a – ^{1}H NMR ($CDCl_3$, 400 MHz)

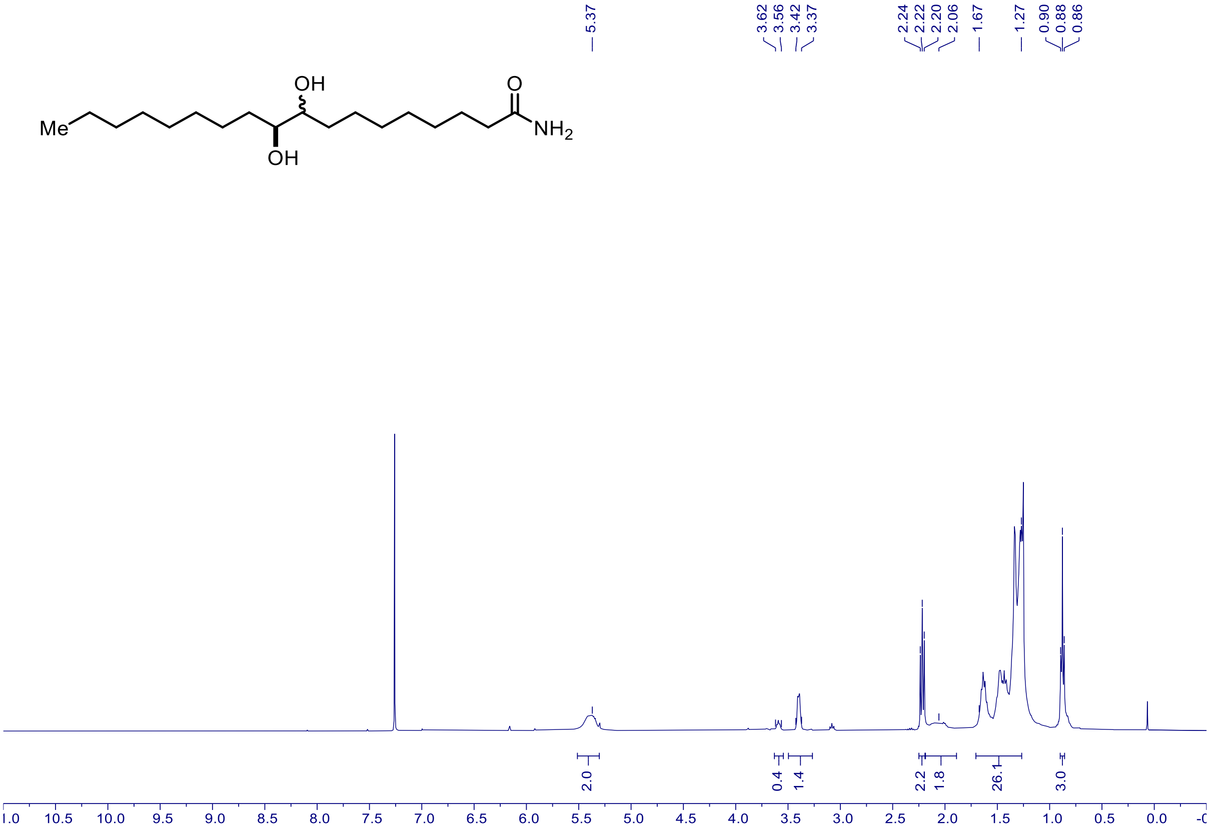

34a – ^{13}C NMR ($CDCl_3$, 101 MHz)

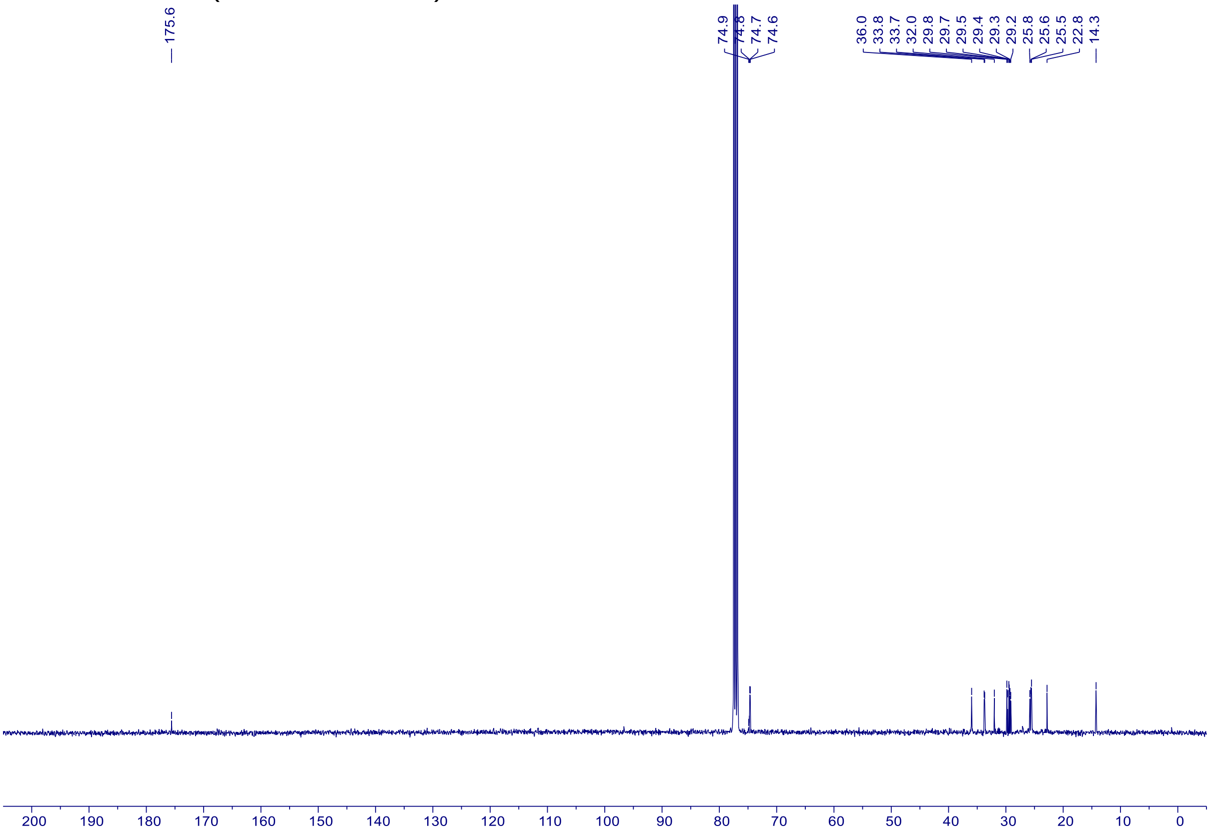

35a – ^{1}H NMR (CDCl$_3$, 400 MHz)

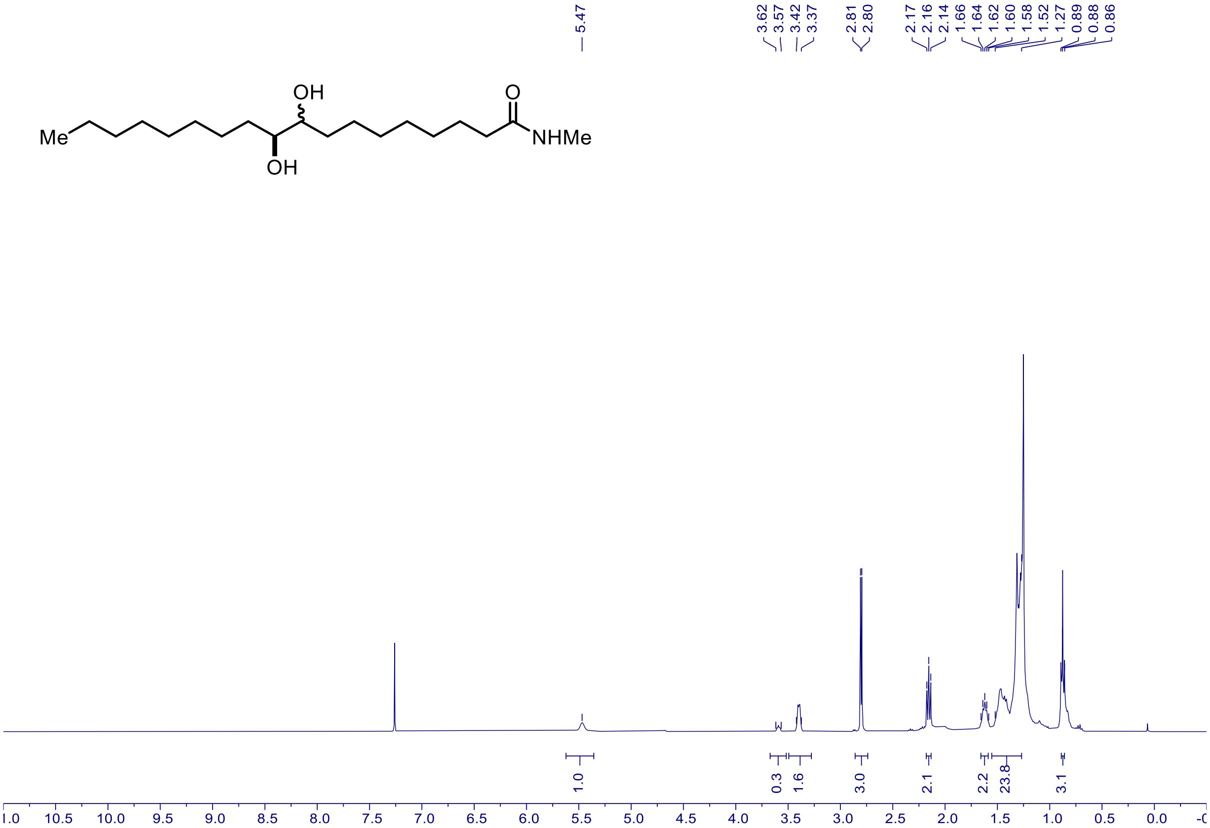

35a – ^{13}C NMR (CDCl$_3$, 101 MHz)

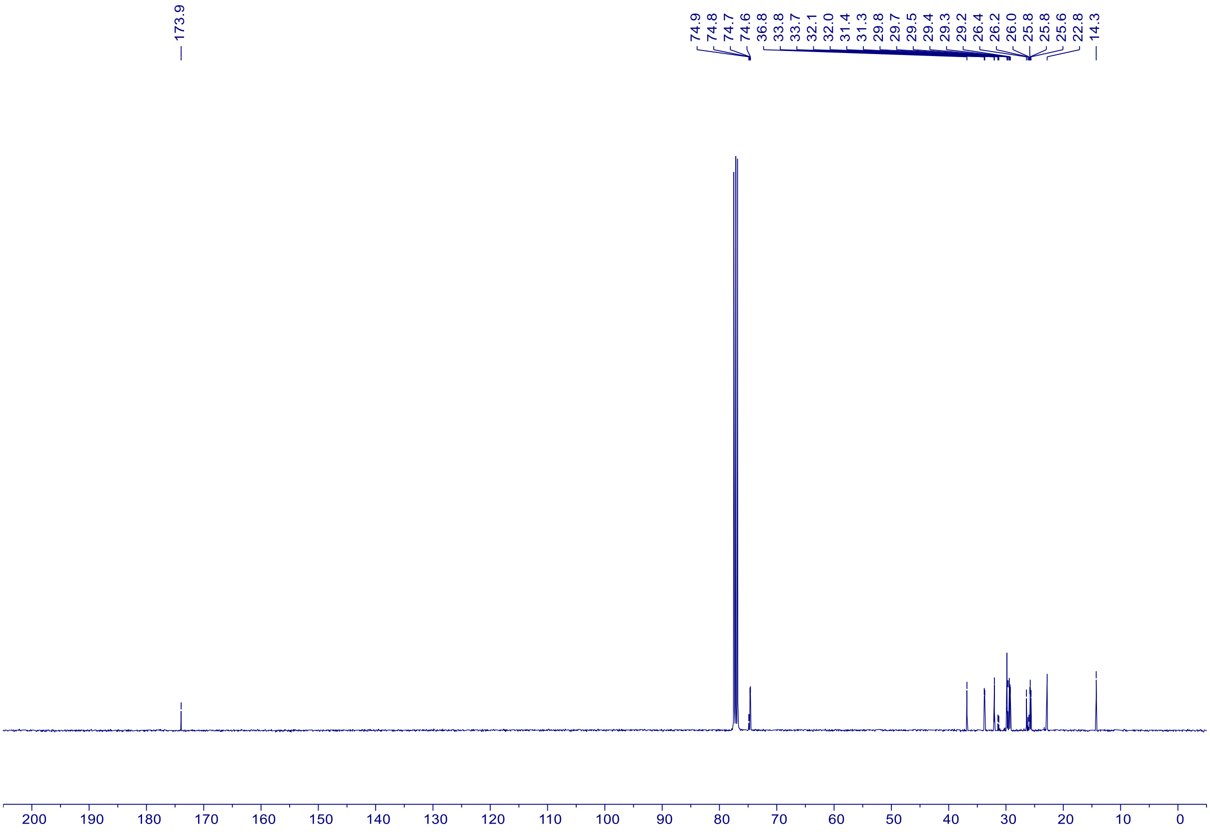

36a – ^{1}H NMR ($CDCl_3$, 400 MHz)

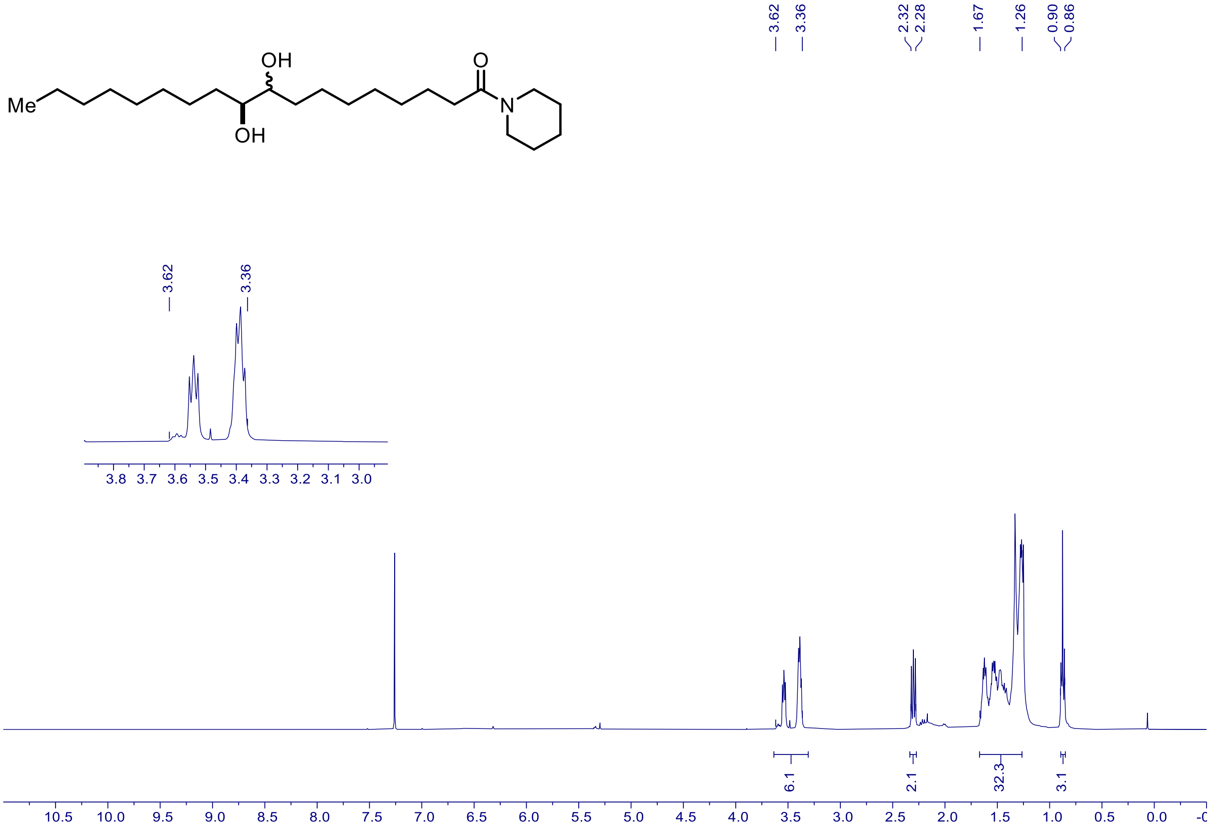

36a – ^{13}C NMR ($CDCl_3$, 101 MHz)

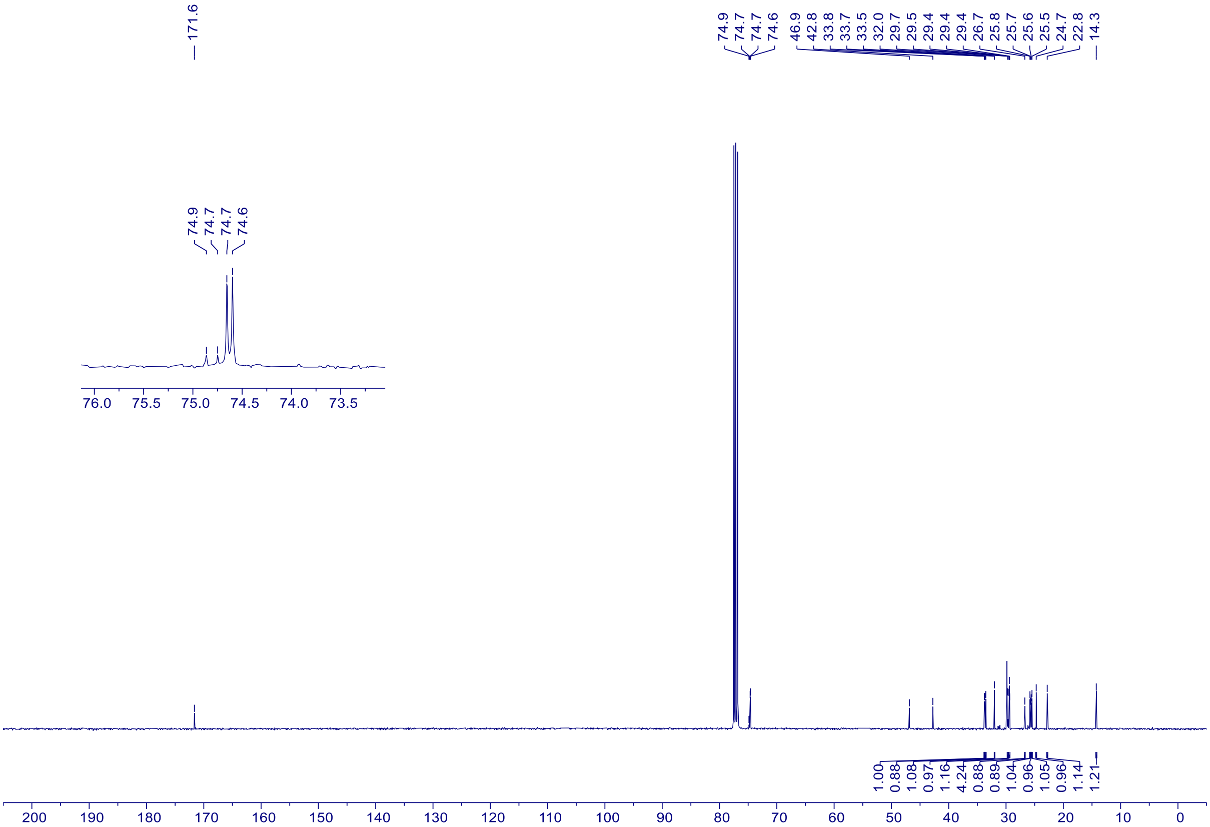

37a – ^{1}H NMR ($CDCl_3$, 400 MHz)

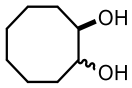

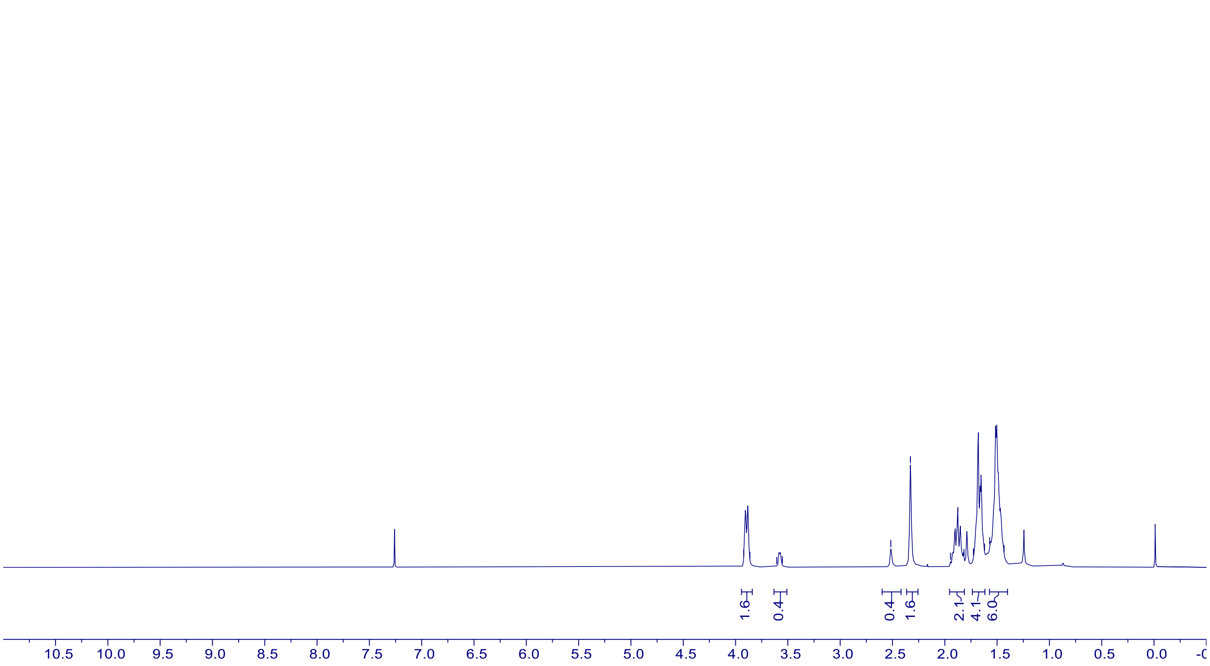

37a – ^{13}C NMR ($CDCl_3$, 126 MHz)

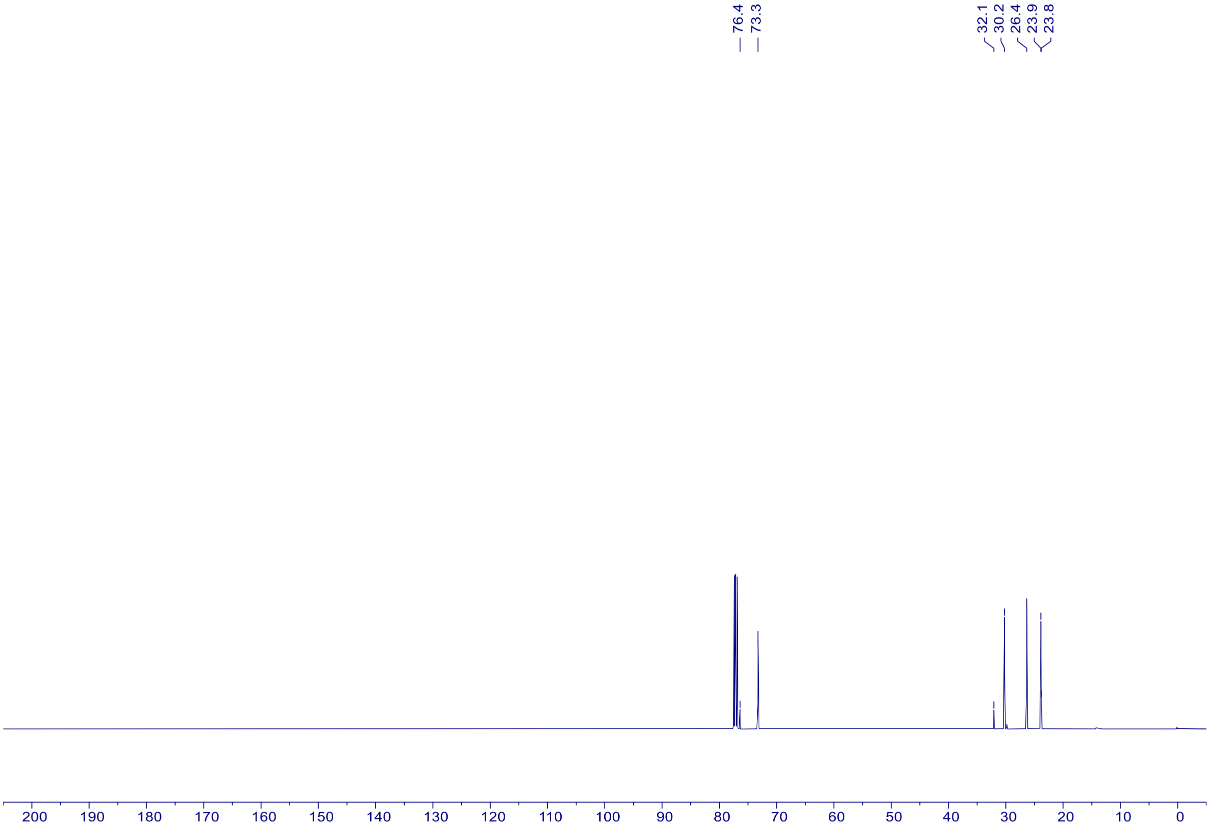

38a' – ^{1}H NMR ($CDCl_3$, 500 MHz)

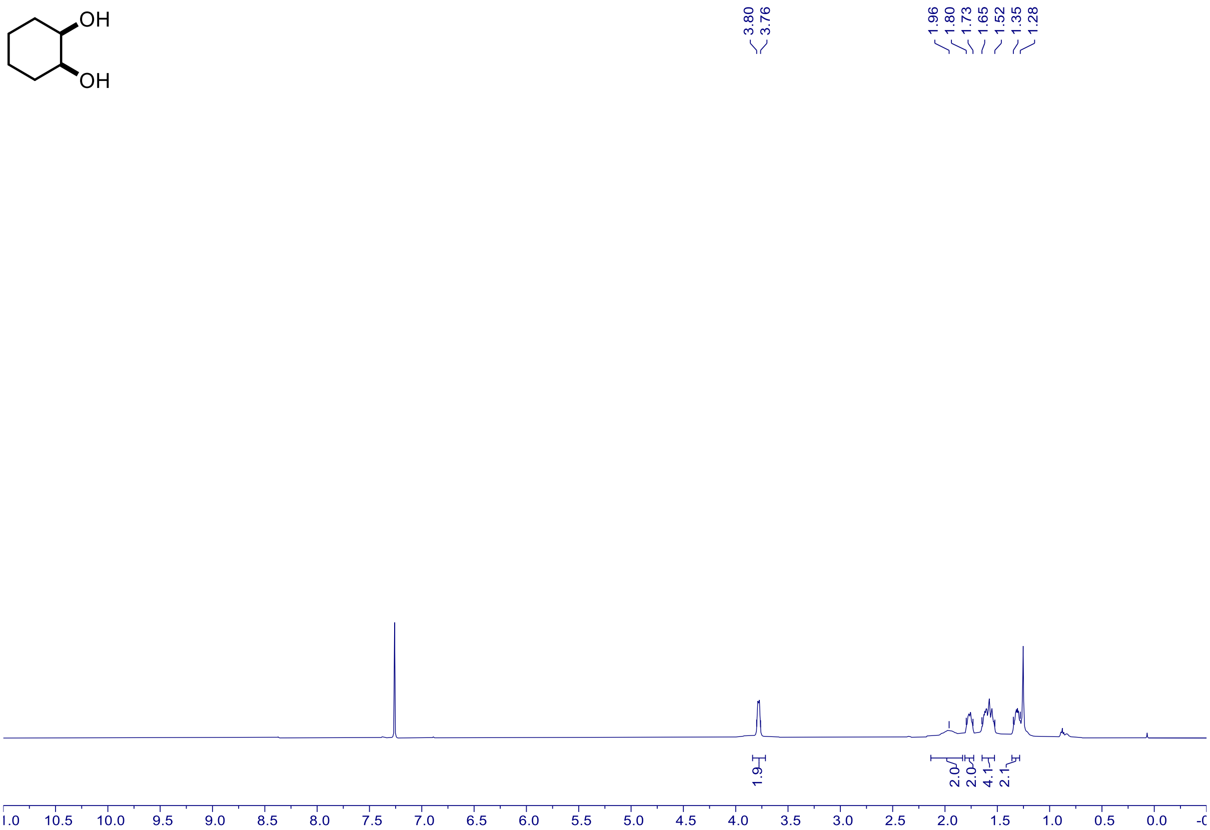

38a' – ^{13}C NMR ($CDCl_3$, 126 MHz)

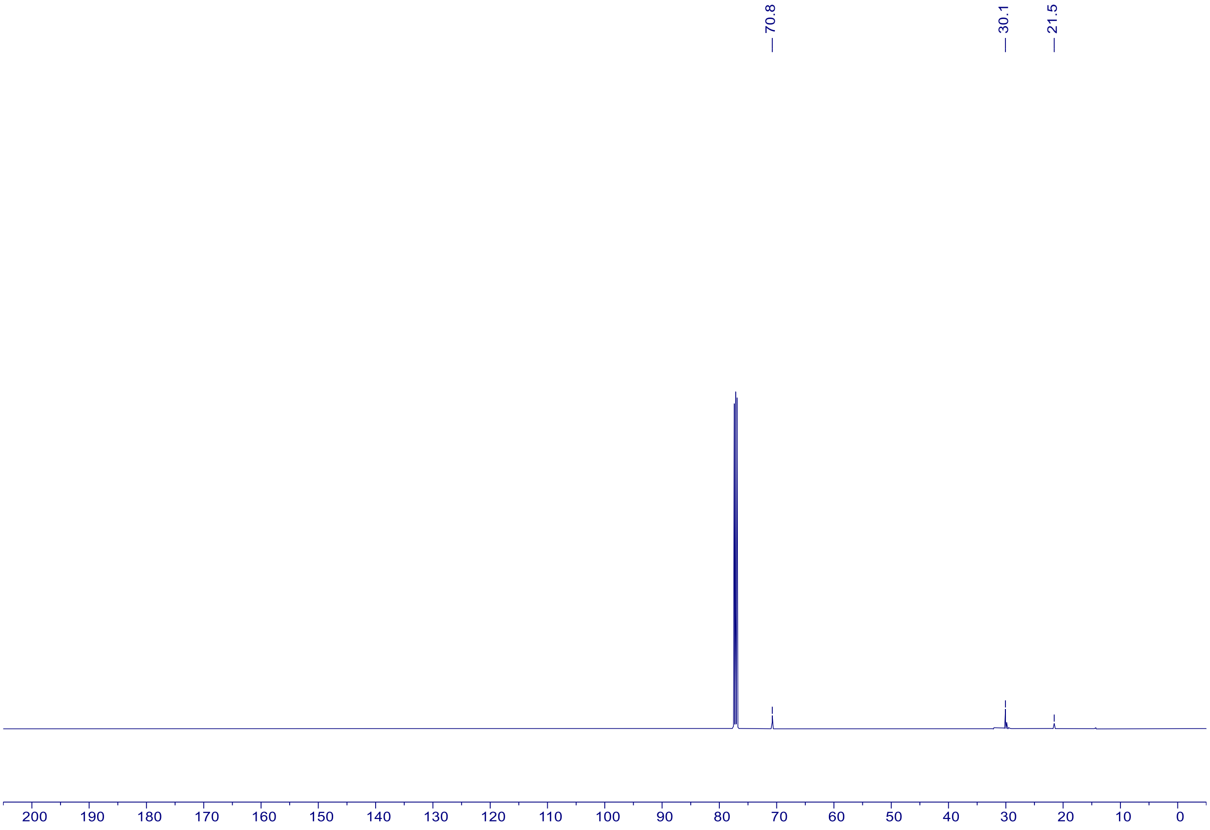

38a'' – ^{1}H NMR ($CDCl_3$, 500 MHz)

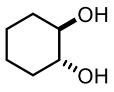

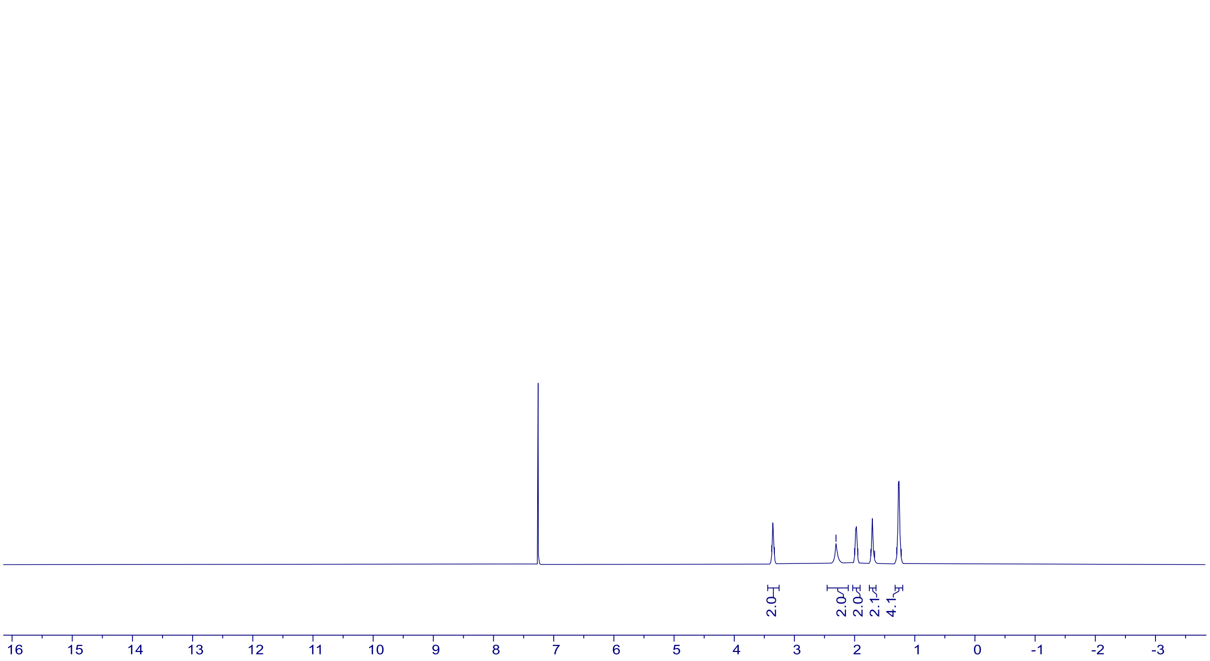

38a'' – ^{13}C NMR ($CDCl_3$, 101 MHz)

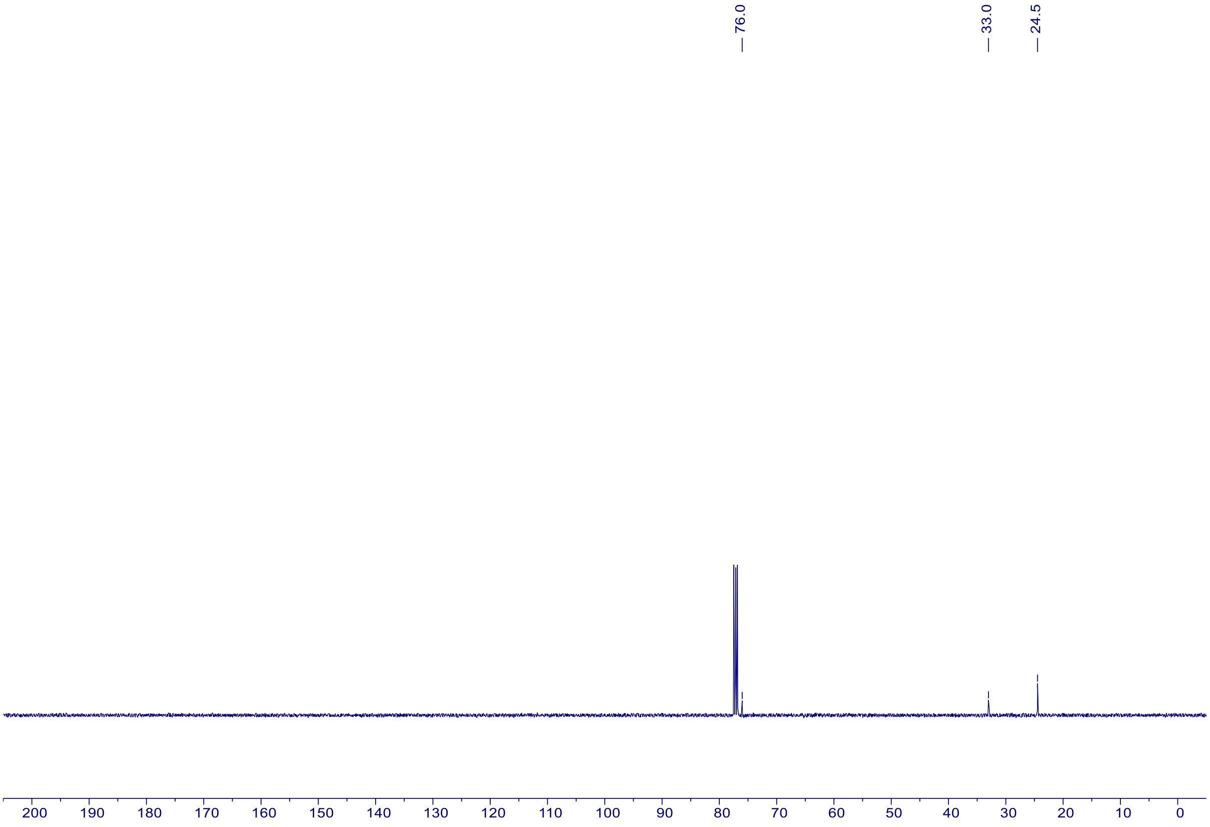

39a – ^{1}H NMR ($CDCl_3$, 500 MHz)

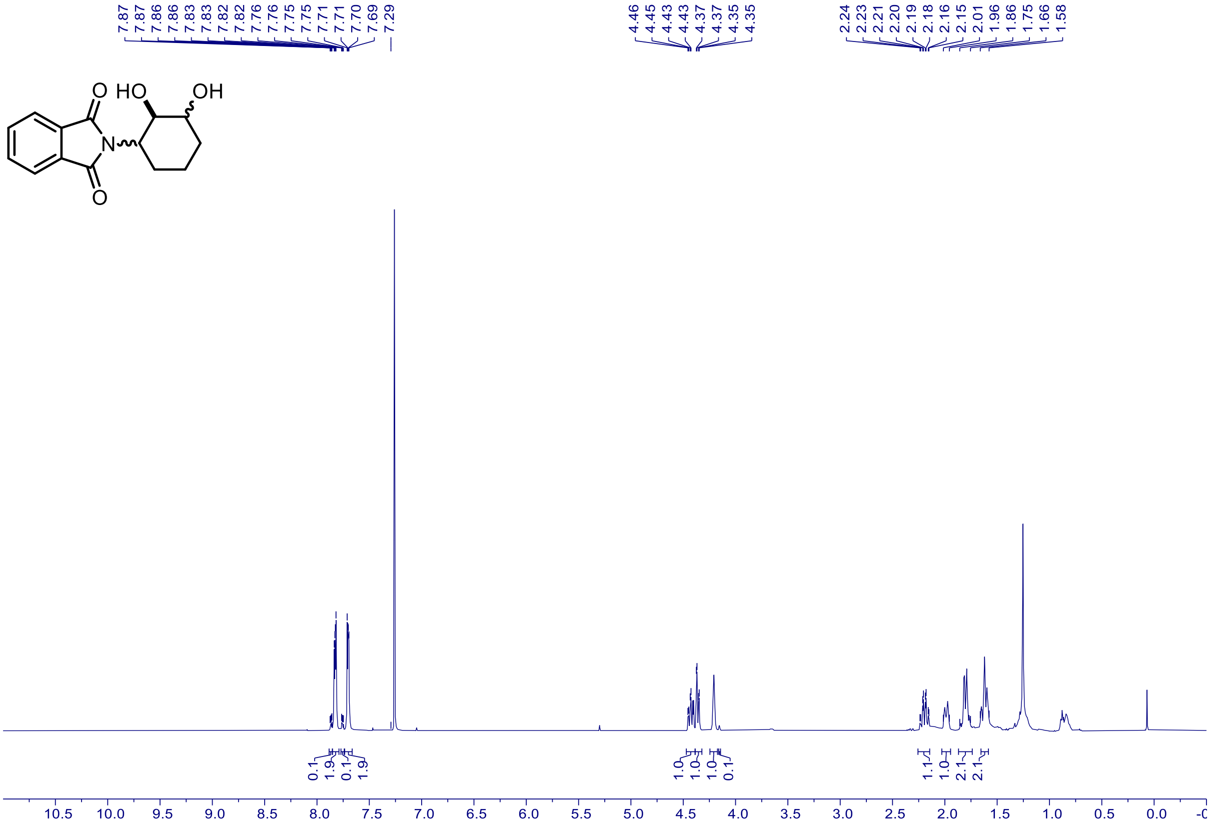

39a – ^{13}C NMR ($CDCl_3$, 126 MHz)

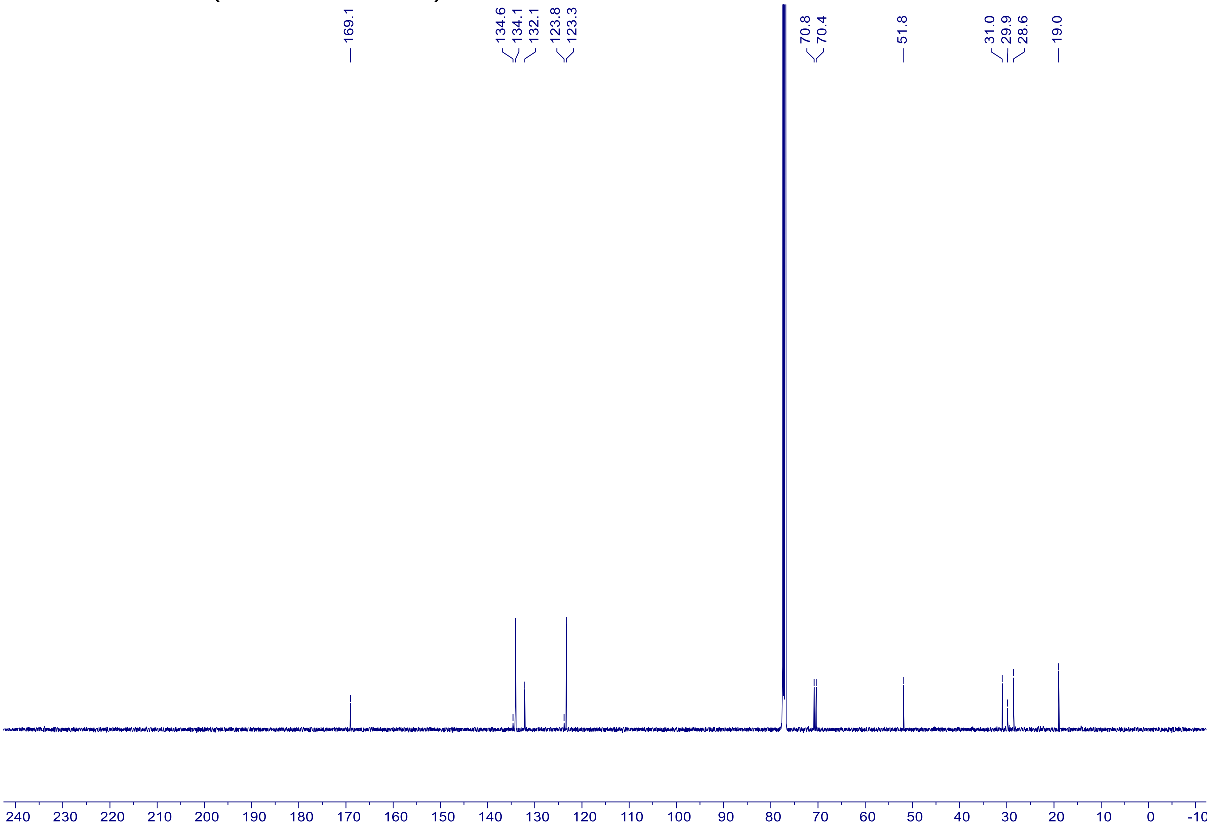

39a' – ^{1}H NMR (CDCl$_3$, 500 MHz)

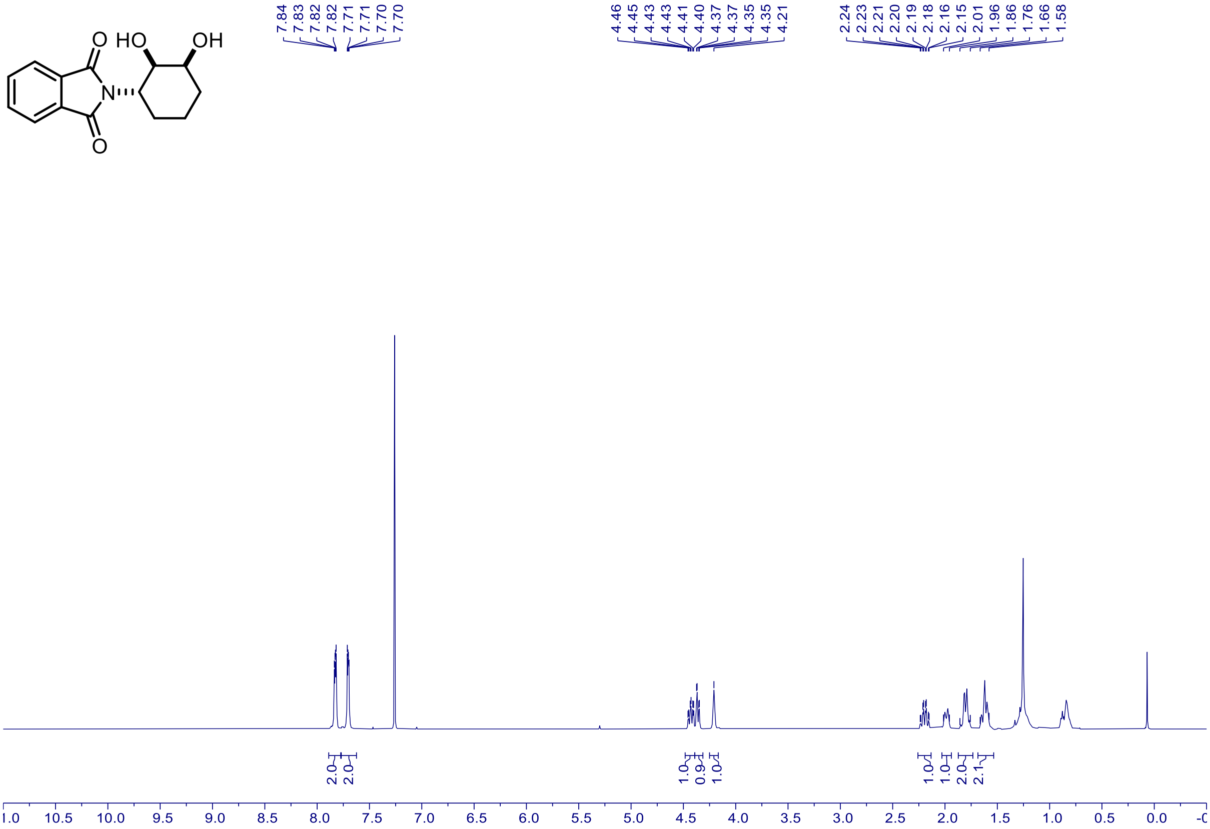

39a' – ^{13}C NMR (CDCl$_3$, 126 MHz)

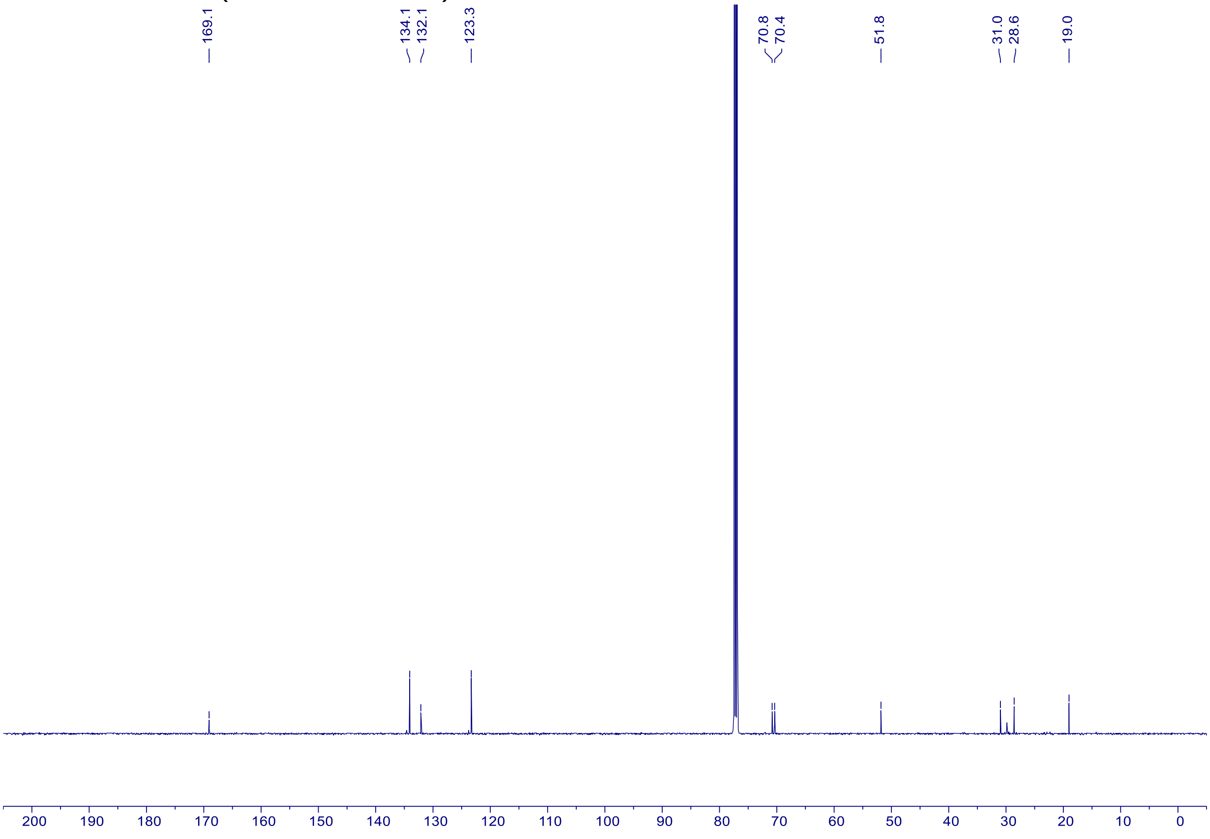

40a – ^{1}H NMR ($CDCl_3$, 400 MHz)

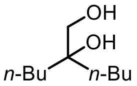

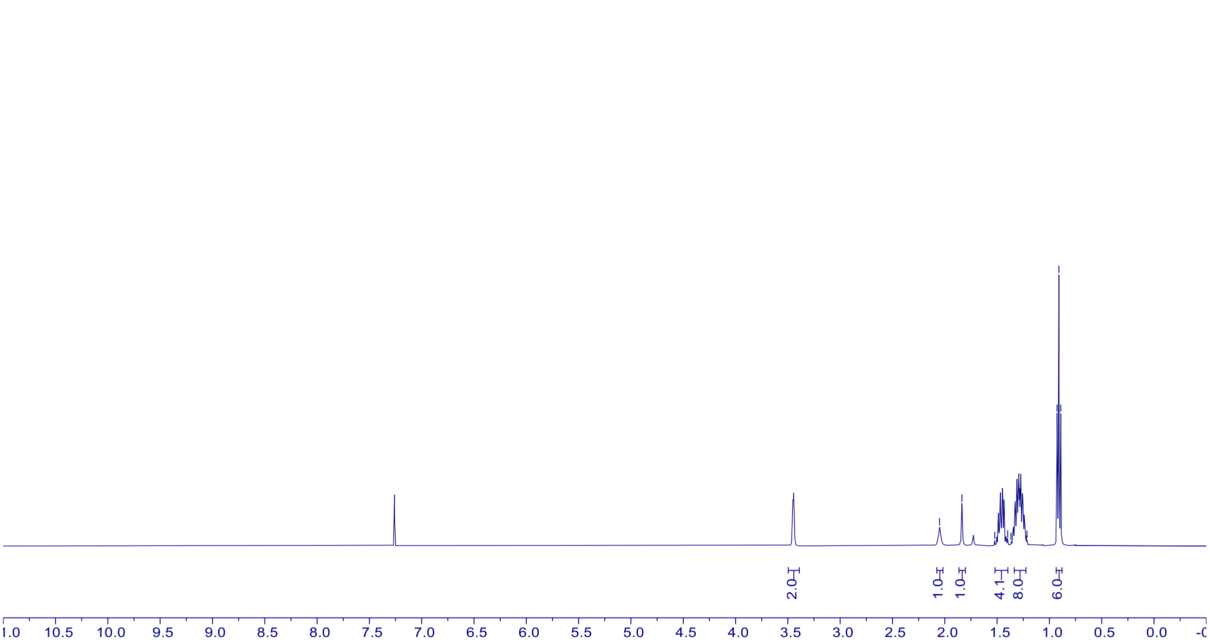

40a – ^{13}C NMR ($CDCl_3$, 101 MHz)

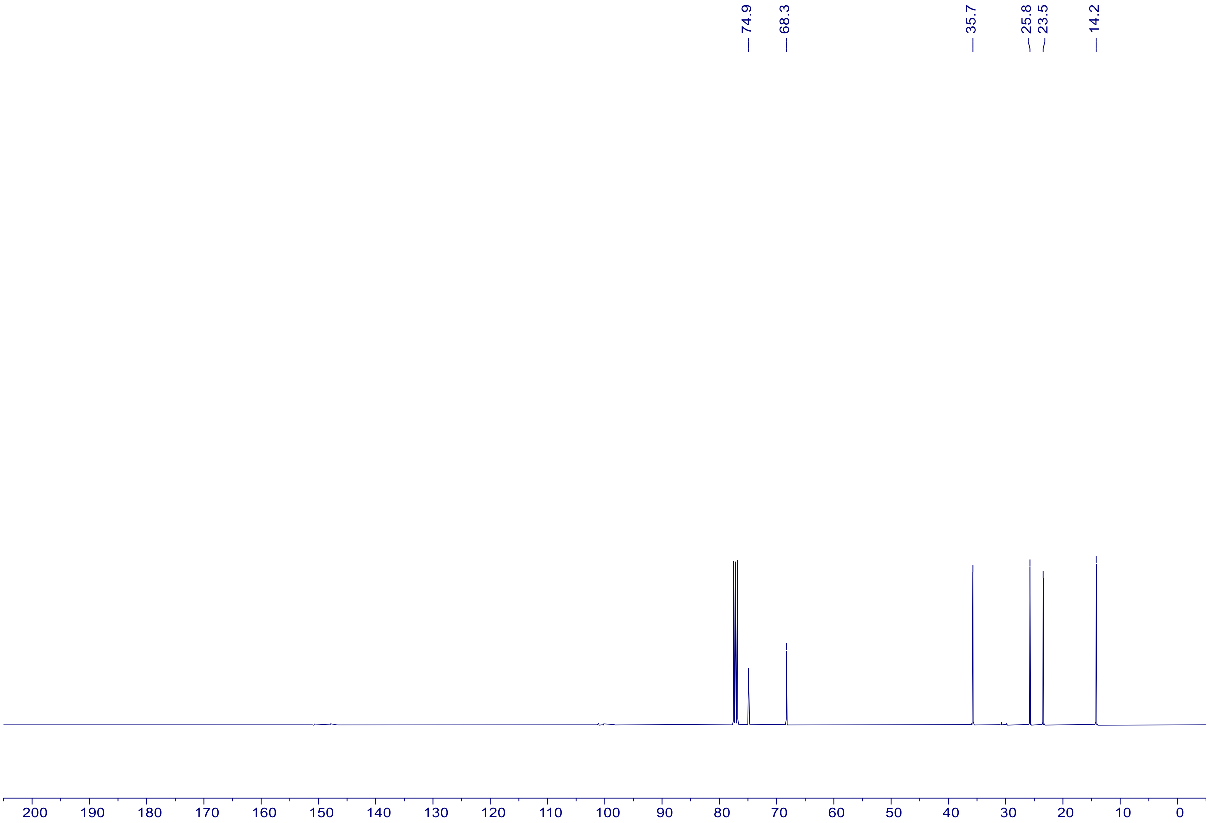

41a – ^{1}H NMR ($CDCl_3$, 400 MHz)

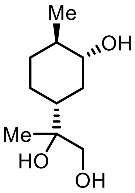

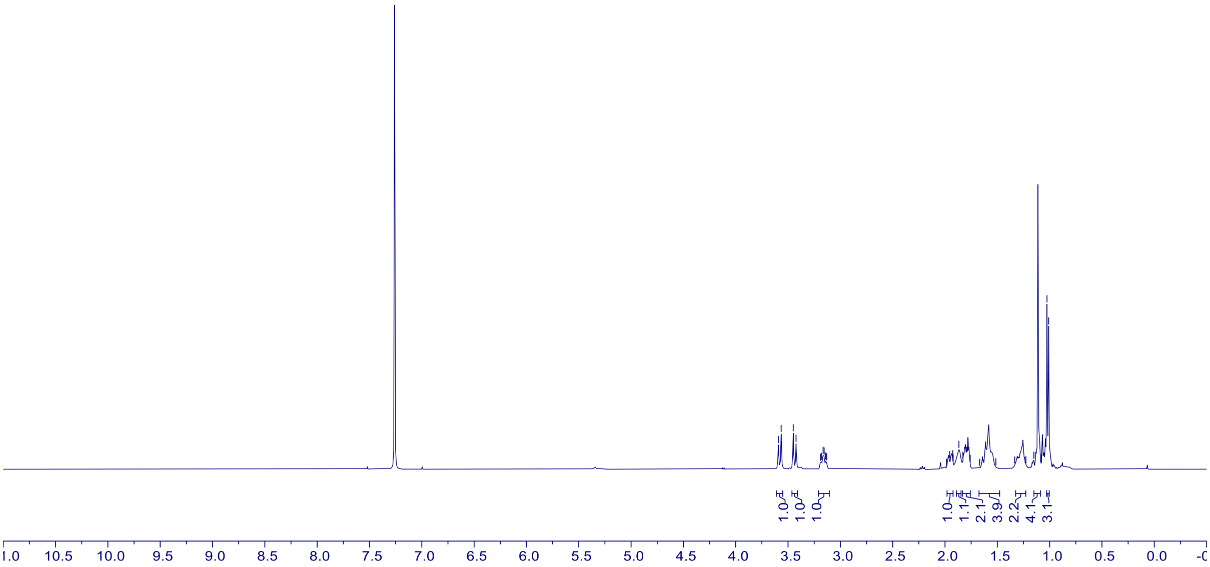

41a – ^{13}C NMR ($CDCl_3$, 101 MHz)

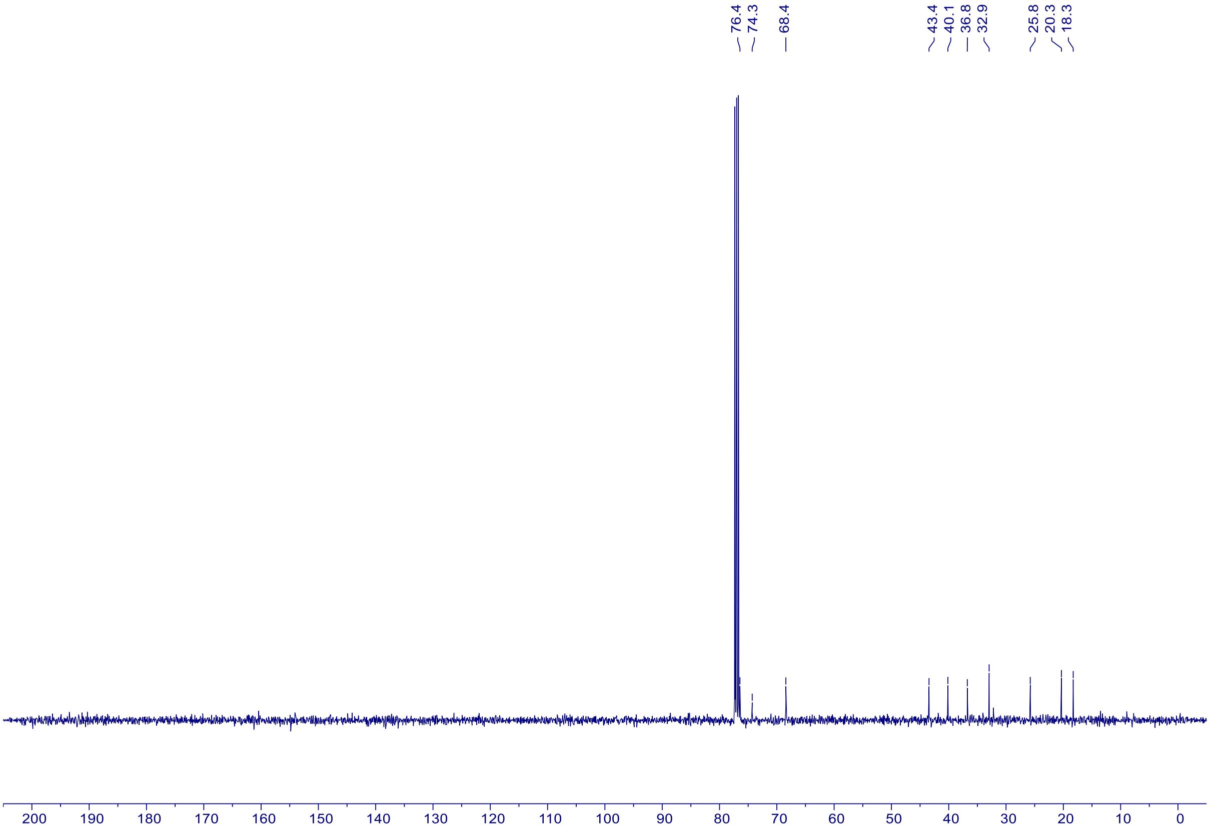

42a – ^{1}H NMR (CD_3CN, 400 MHz)

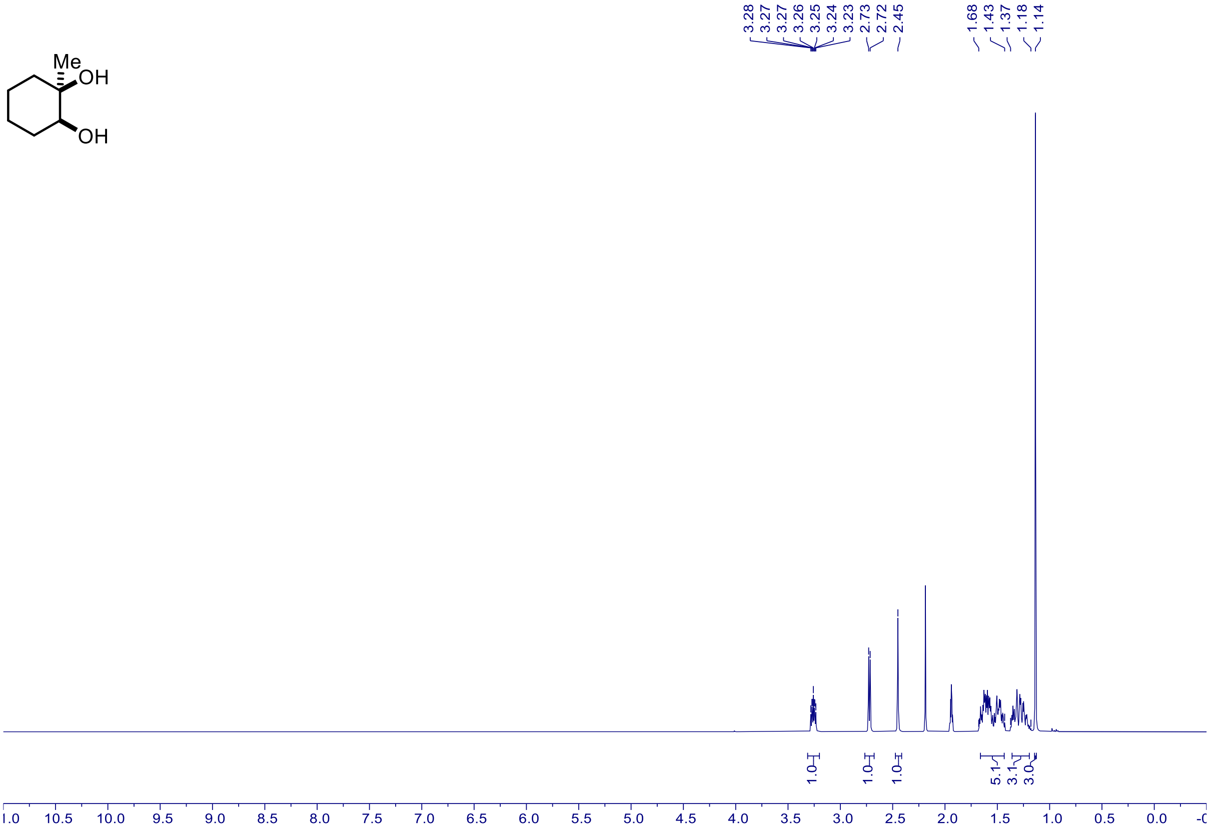

42a – ^{13}C NMR (CD_3CN, 101 MHz)

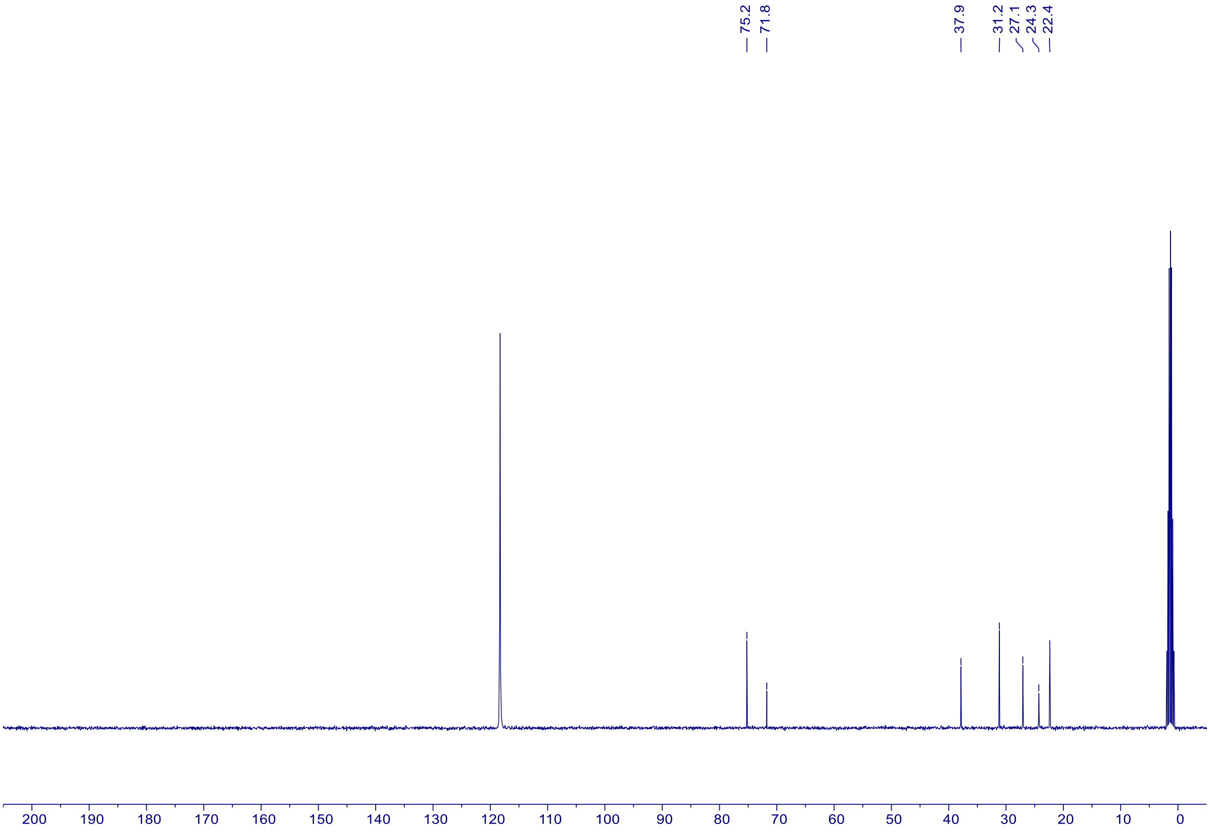

43a – ^{1}H NMR ($CDCl_3$, 400 MHz)

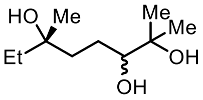

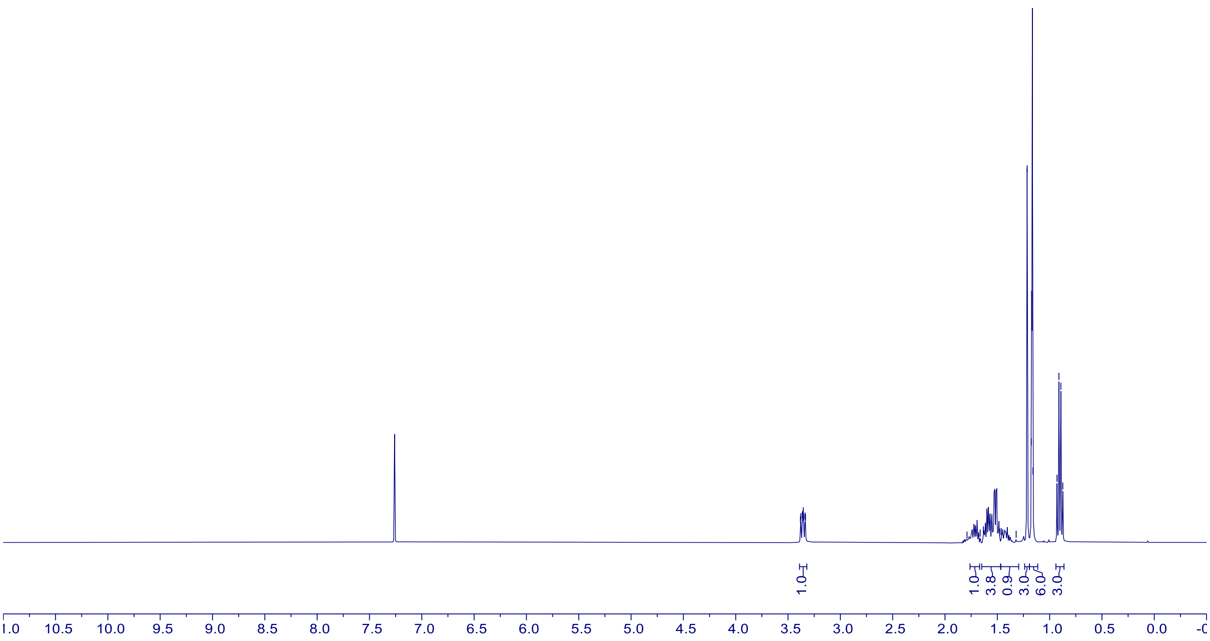

43a – ^{13}C NMR ($CDCl_3$, 101 MHz)

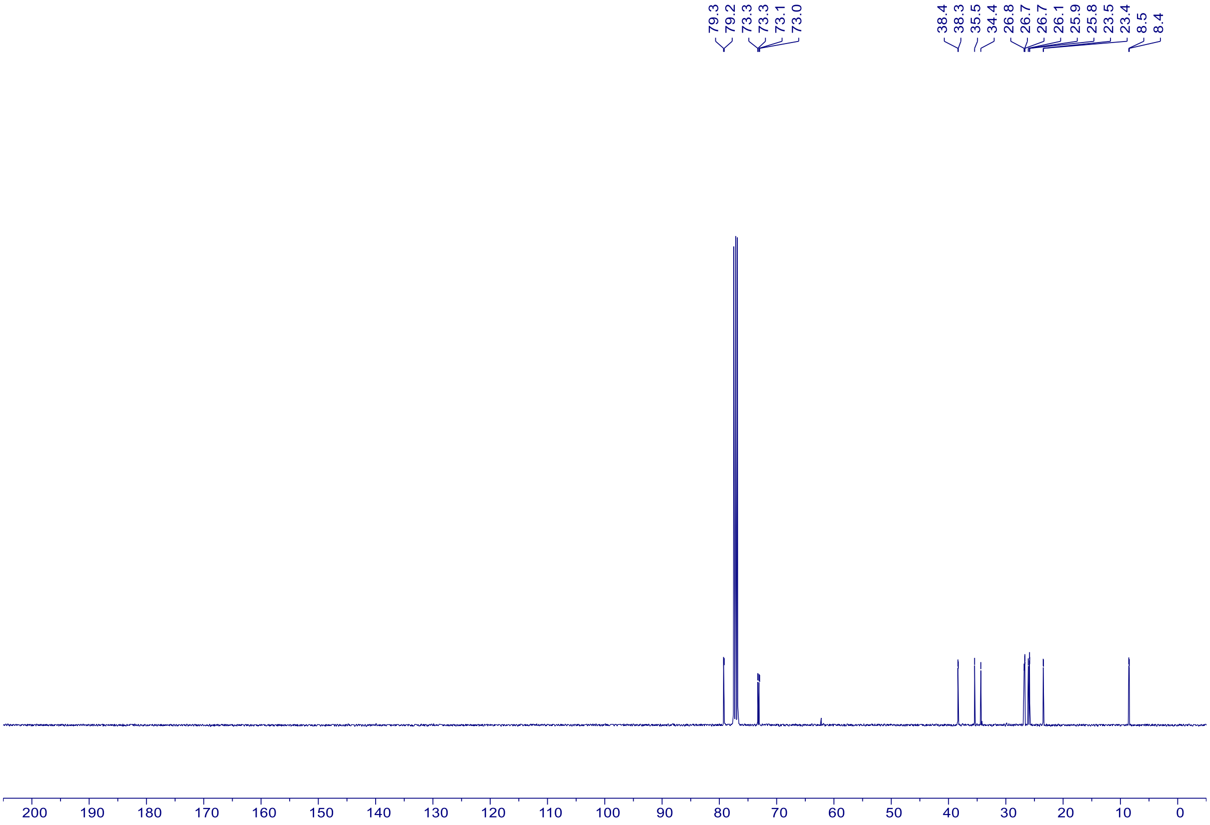

8.2 NMR Spectra of Anilines

2 – ^{1}H NMR ($CDCl_3$, 400 MHz)

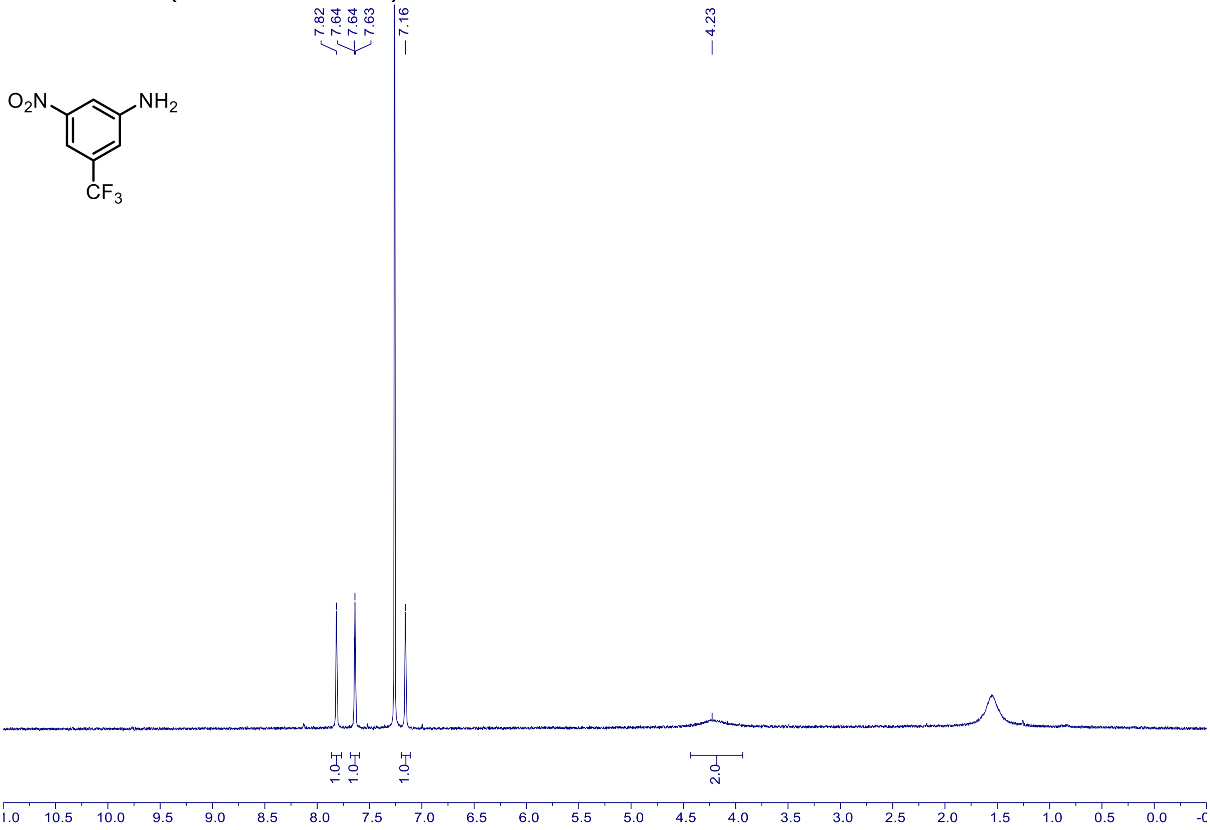

2 – ^{19}F NMR ($CDCl_3$, 376 MHz)

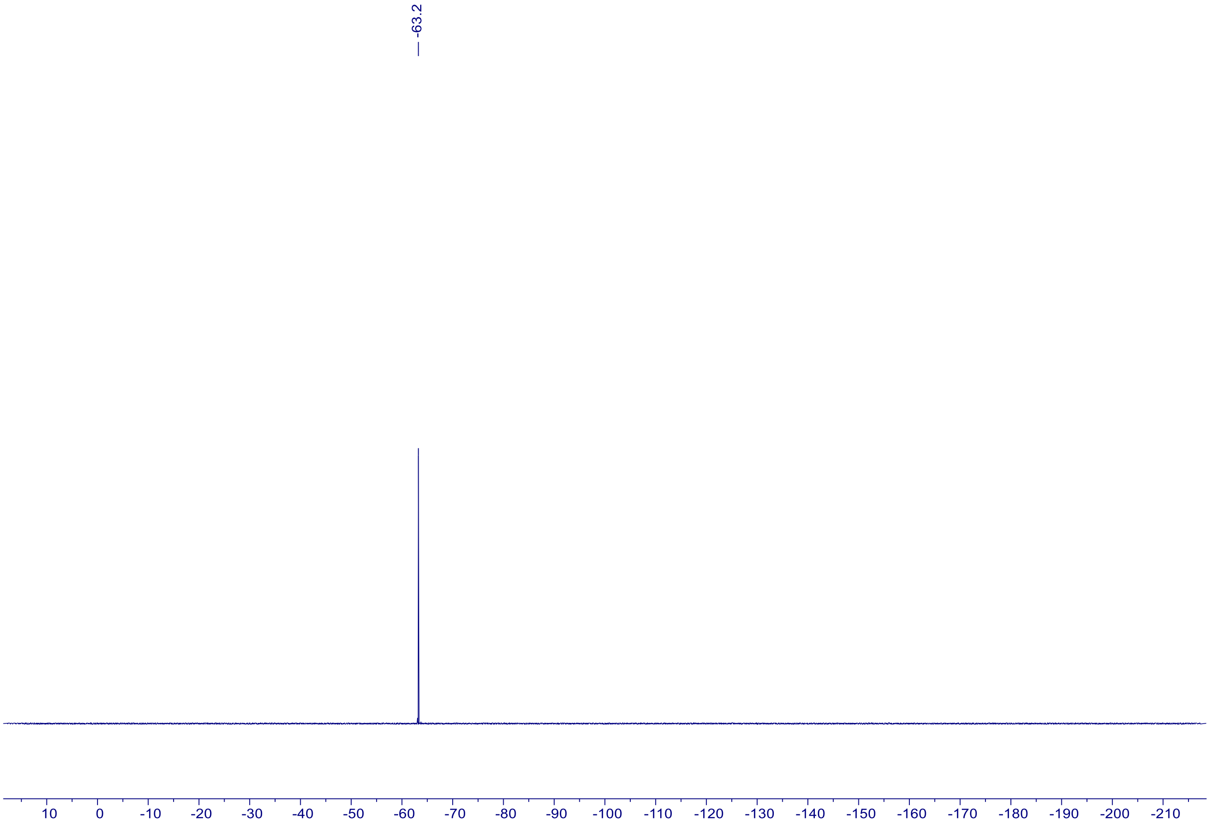

3 – ^{1}H NMR ($CDCl_3$, 400 MHz)

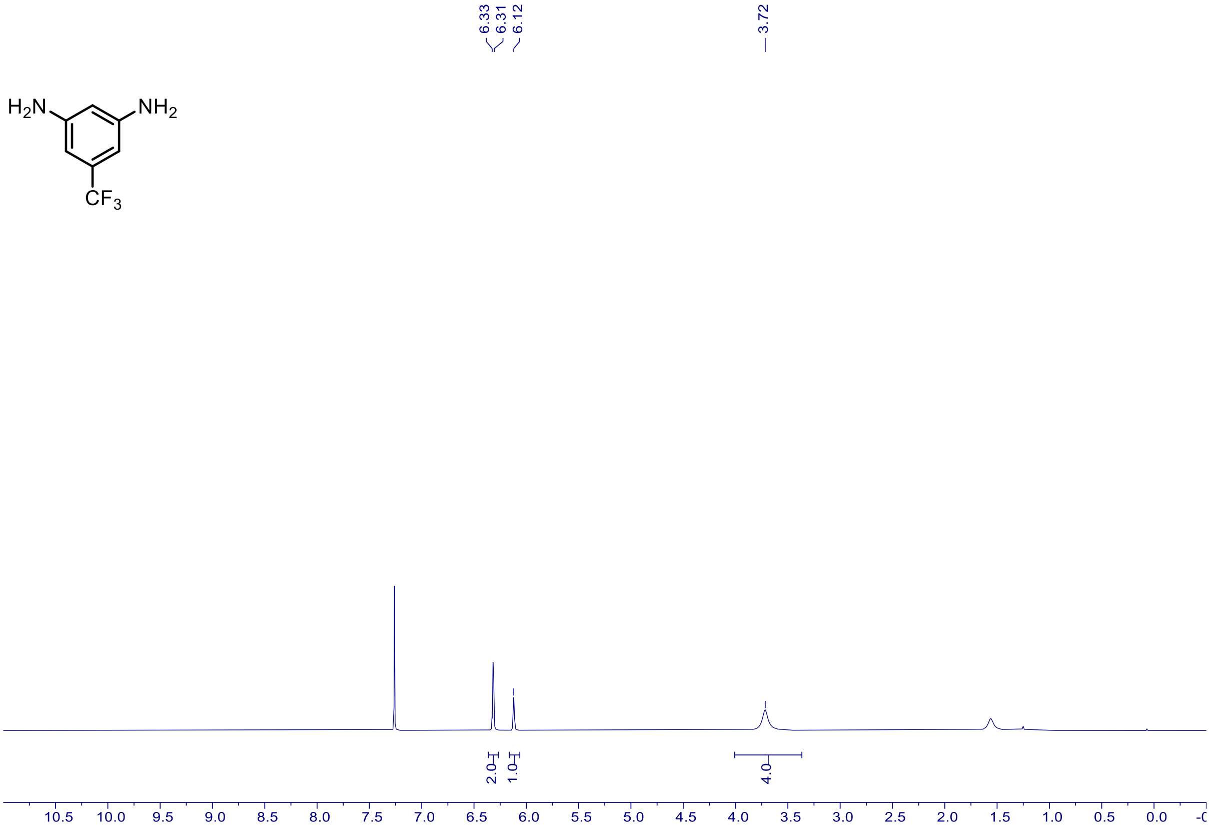

3 – ^{19}F NMR ($CDCl_3$, 376 MHz)

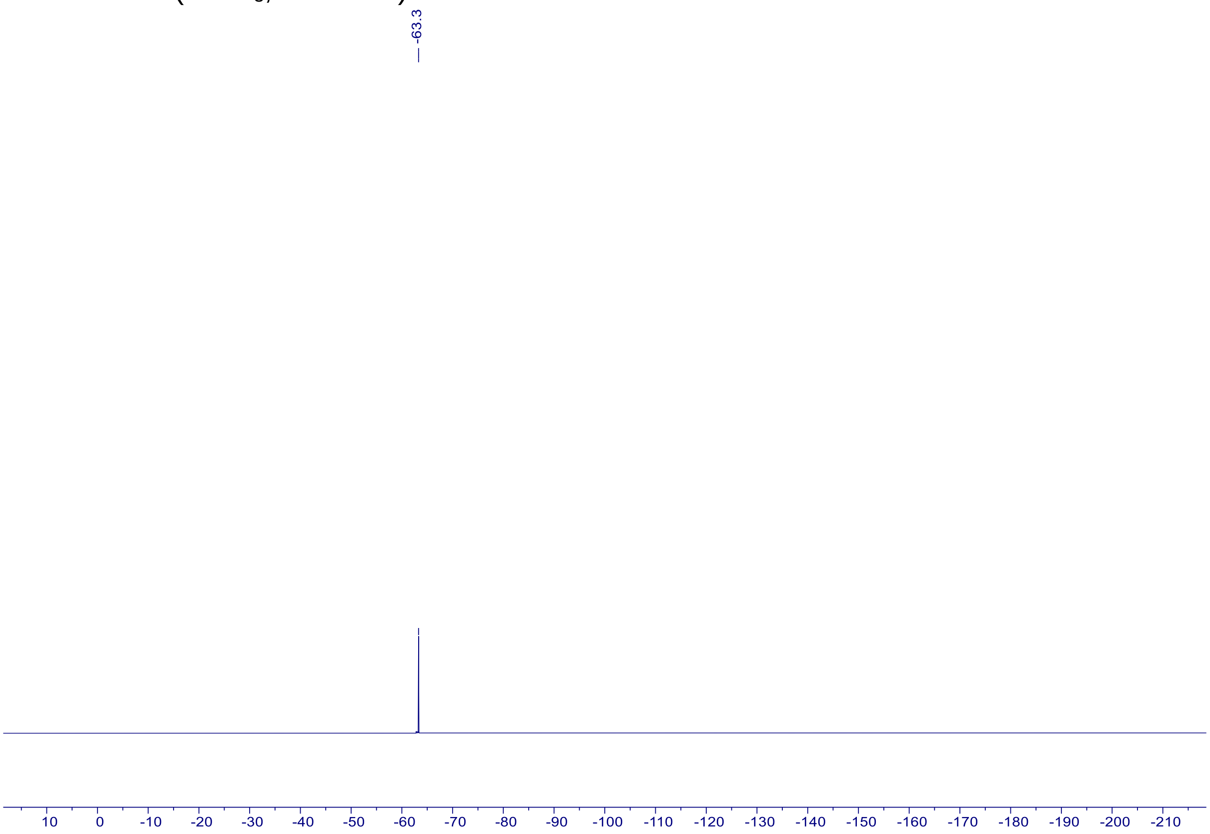

46 – ^{1}H NMR ($CDCl_3$, 400 MHz)

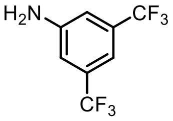

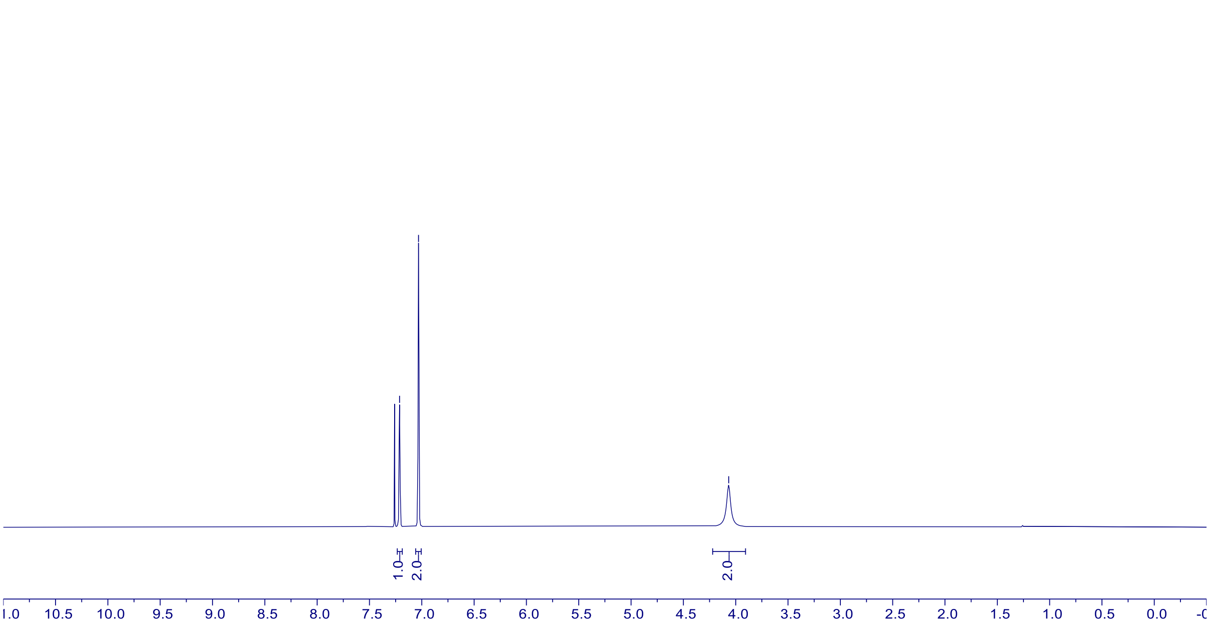

46 – ^{13}C NMR ($CDCl_3$, 101 MHz)

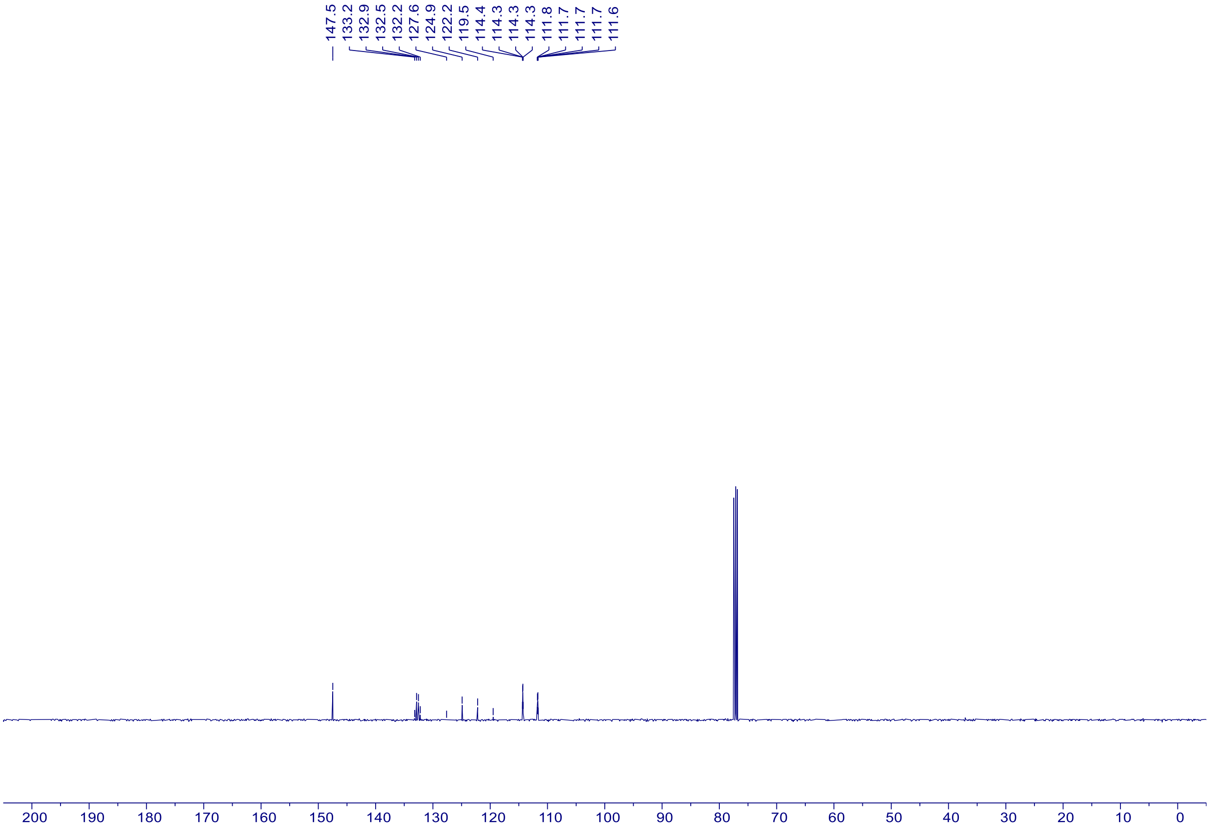

46 – ^{19}F NMR ($CDCl_3$, 471 MHz)

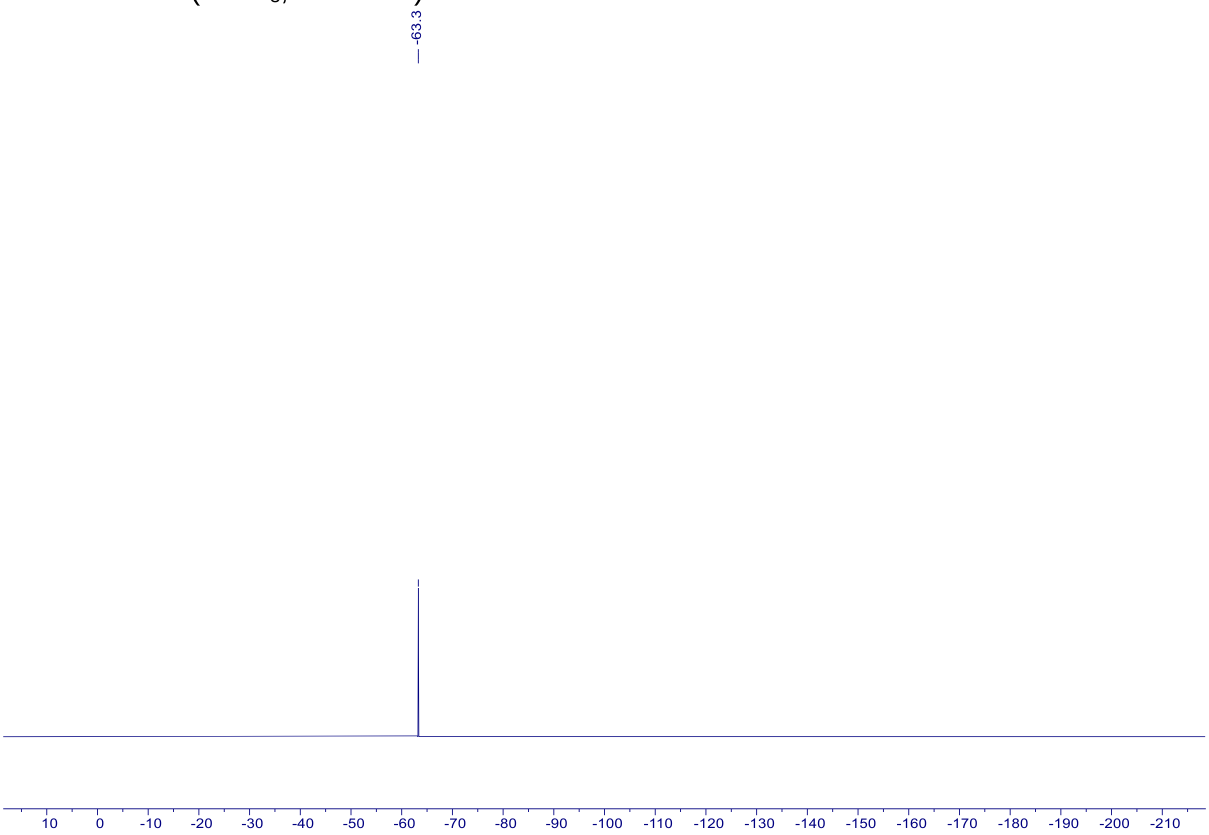

47 – ^{1}H NMR ($CDCl_3$, 400 MHz)

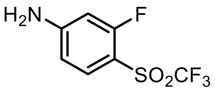

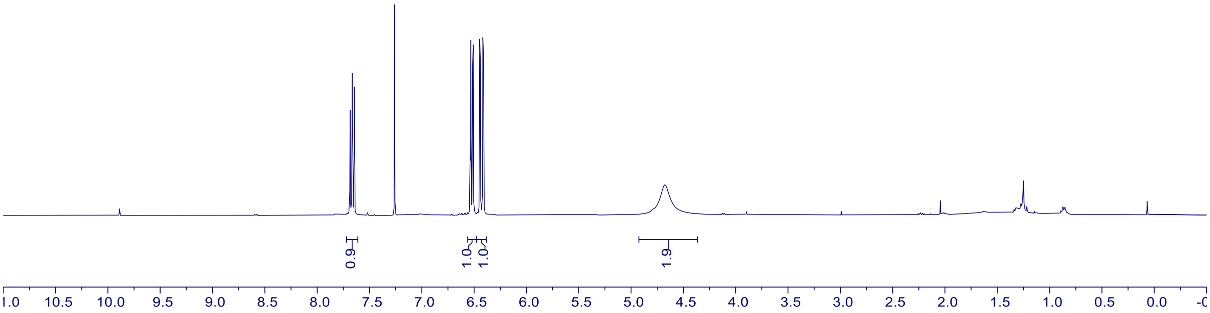

47 – ^{13}C NMR ($CDCl_3$, 101 MHz)

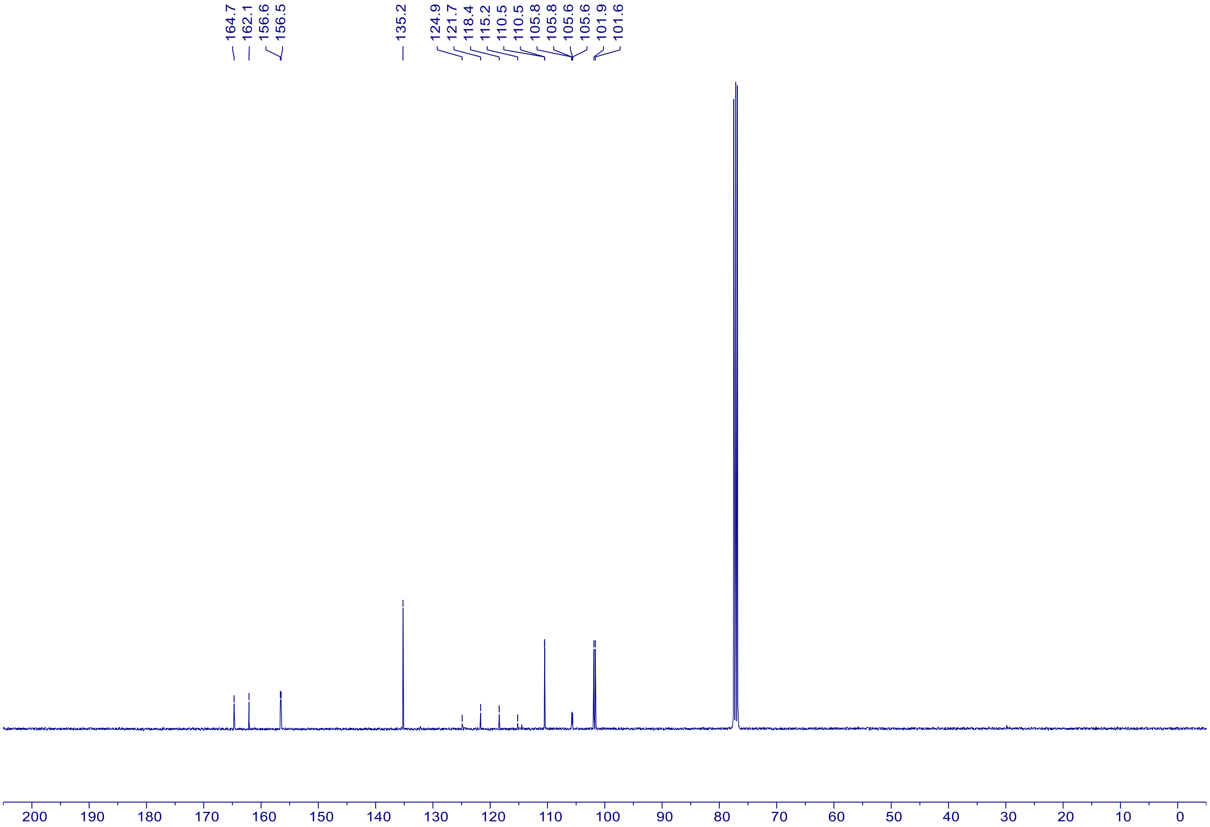

47 – $^{19}F\ \{^{1}H\}$ NMR ($CDCl_3$, 376 MHz)

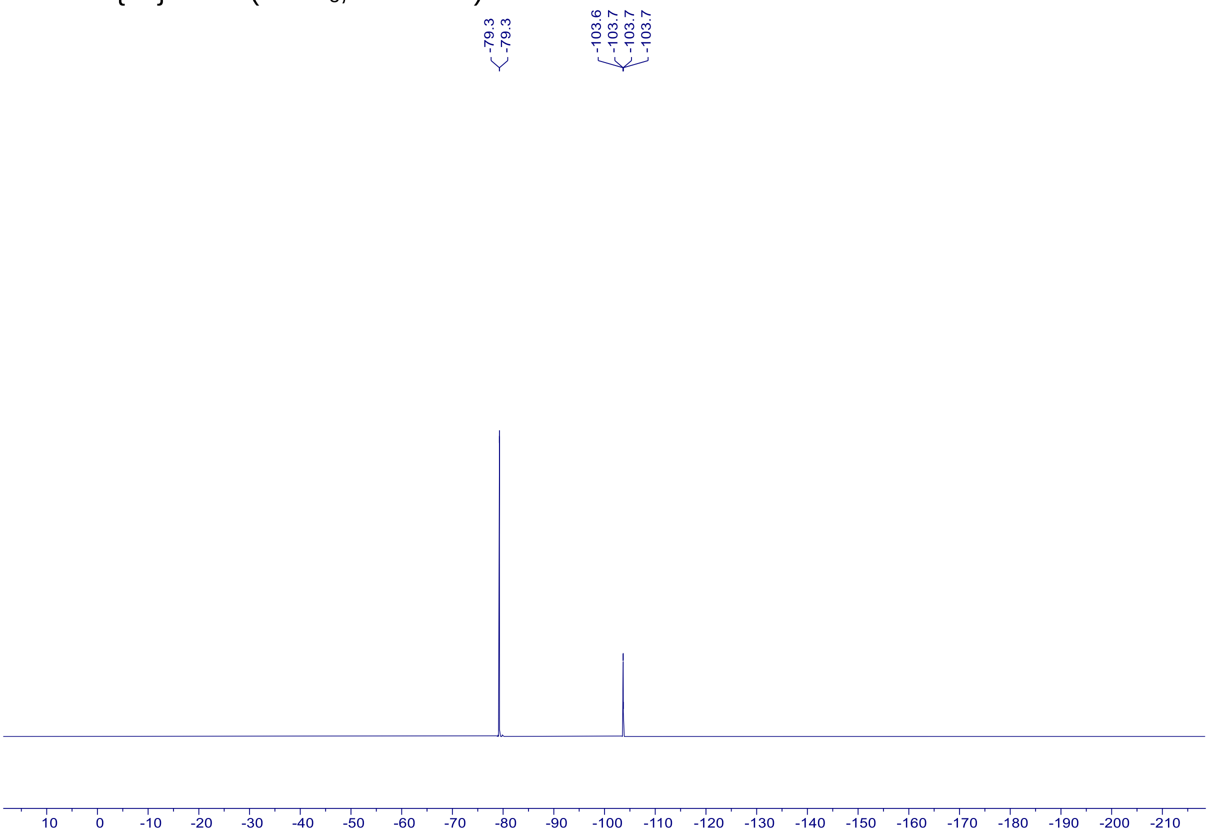

47 – ^{19}F NMR ($CDCl_3$, 376 MHz)

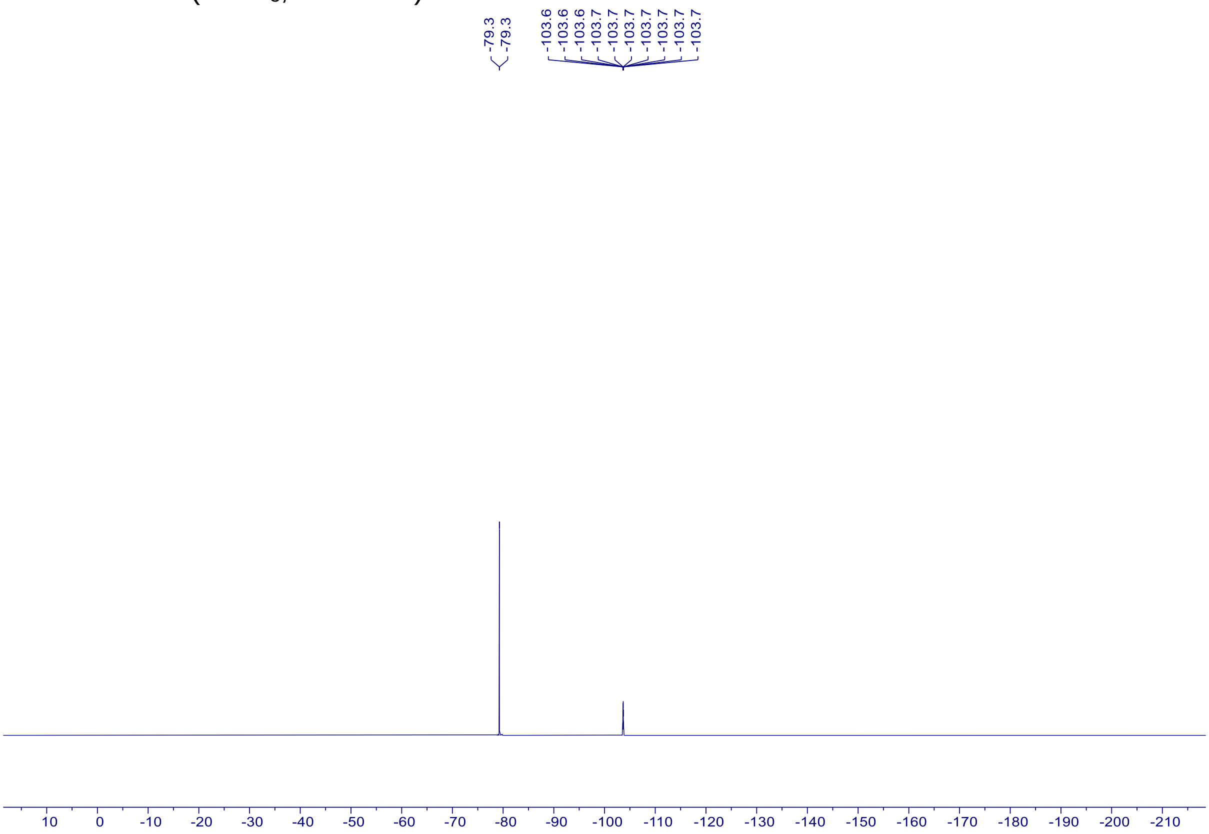

48 – ^{1}H NMR ($CDCl_3$, 400 MHz)

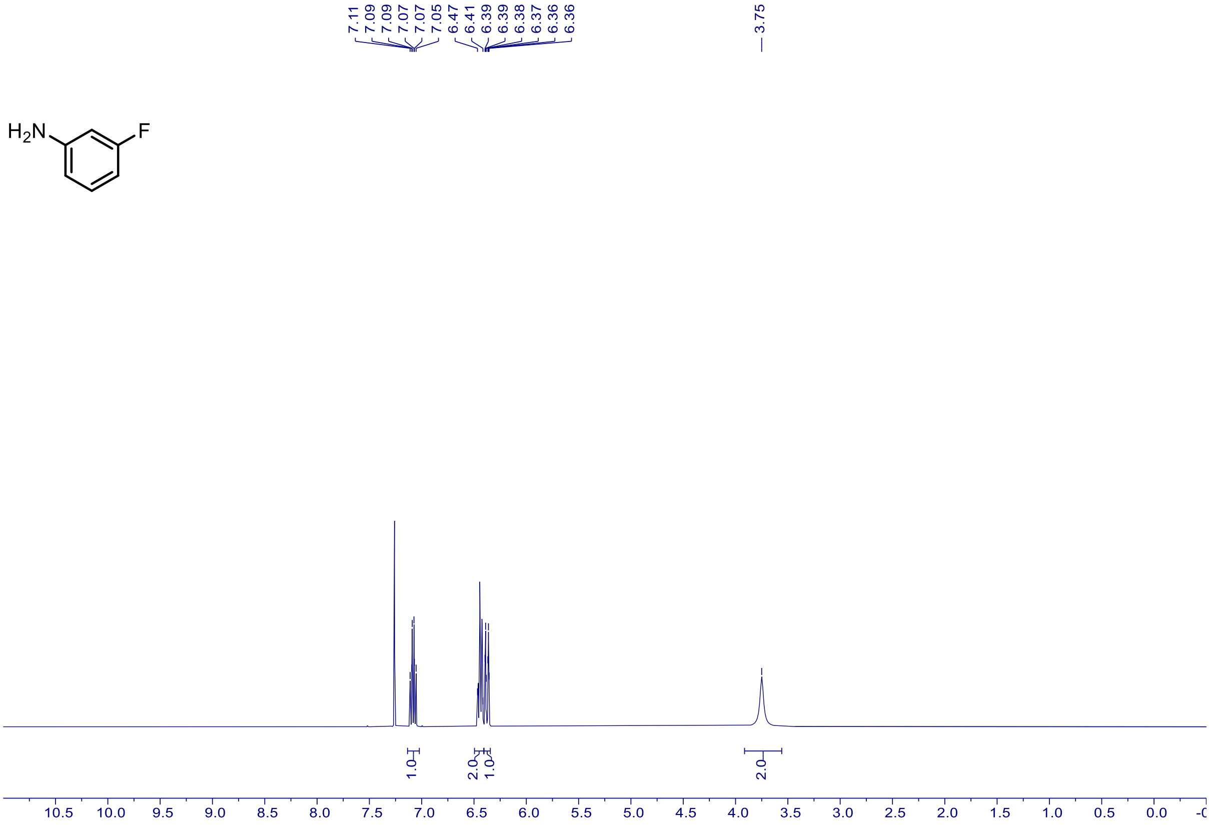

48 – ^{13}C NMR ($CDCl_3$, 101 MHz)

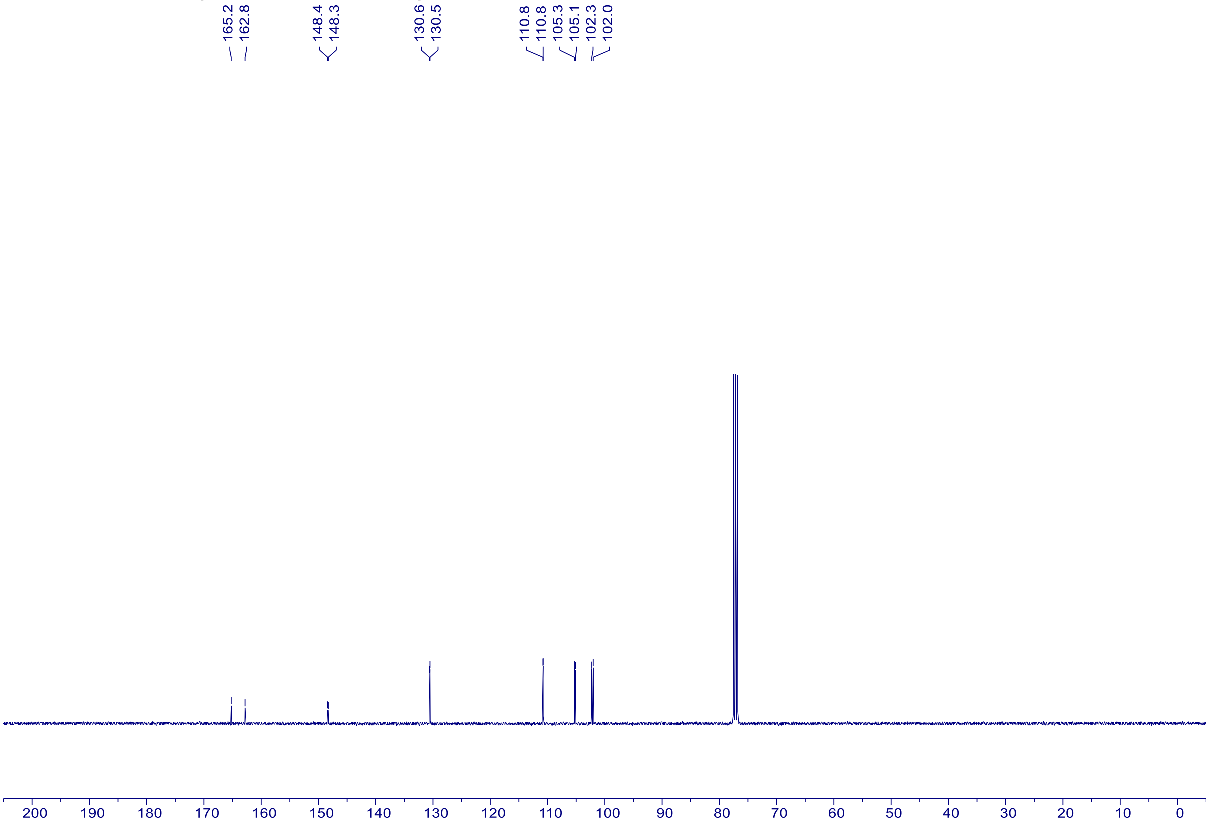

48 – ^{19}F NMR ($CDCl_3$, 376 MHz)

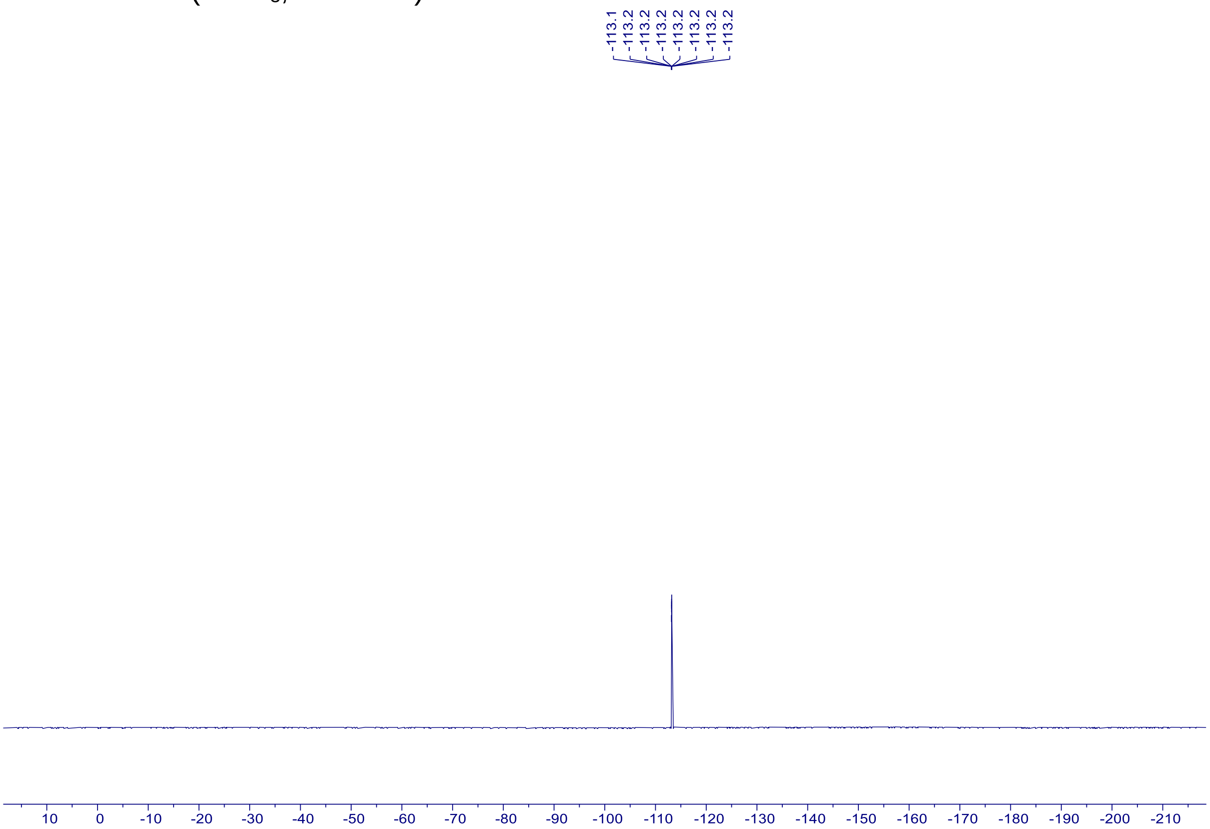

9 References

[1] A. Ruffoni, C. Hampton, M. Simonetti, D. Leonori, *Nature* **2022**, 610, 81–86.

[2] H. Lu, T. Yang, Z. Xu, X. Lin, Q. Ding, Y. Zhang, Z. Cai, K. Dong, S. Gong, W. Zhang, M. Patel, R. C. b. Copley, J. Xiang, X. Guan, P. Wren, F. Ren, *J. Med. Chem.* **2018**, *61*, 2518–2532.

[3] T. Salmi, in *Advances in Chemical Engineering: Chemical Engineering for Renewables Conversion* (Ed.: D. Y. Murzin), Academic Press, Massachusetts, **2013**, pp. 195–216.

[4] X. Wu, F. A. Cruz, V. M. Dong, *J. Am. Chem. Soc.* **2018**, *140*, 10126–10130.

[5] L. Yan, Y. Meng, F. Haeffner, R. M. Leon, M. P. Crockett, J. P. Morken, *J. Am. Chem. Soc.* **2018**, *140*, 3663–3673.

[6] J. Y. Kang, B. T. Connell, *J. Am. Chem. Soc.* **2010**, *132*, 7826–7827.

[7] S. V. Kanojia, S. Chatterjee, S. Chattopadhyay, D. Goswami, *J. Org. Chem.* **2019**, *15*, 490–496.

[8] J. Bredihhina, P. Villo, K. Andersons, L. Toom, L. Vares, *J. Org. Chem.* **2013**, *78*, 2379–2385.

[9] S. Menichetti, R. Amorati, M. G. Bartolozzi, G. F. Pedulli, A. Salvini, C. A. Viglianisi, *Eur. J. Org. Chem.* **2010**, 2218–2225.

[10] X. Gao, J. Lin, L. Zhang, X. Lou, G. Guanghui, B. Peng, H. Xu, Y. Liu, *J. Org. Chem.* **2021**, *86*, 15469–15480.

[11] B.-Y. Wei, D.-T. Xie, S.-Q. Lai, Y. Jiang, H. Fu, D. Wei, B. Han. *Angew. Chem. Int. Ed.* **2021**, *60*, 3182–3188.

[12] C. Döbler, G. M. Mehltretter, U. Sundermeier, M. Beller, *J. Organomet. Chem.* **2020**, *621*, 70–76.

[13] C. Döbler, G. M. Mehltretter, U. Sundermeier, M. Beller, *J. Am. Chem. Soc.* **2000**, *122*, 10289–10297.

[14] S. A. Valenzuela, H. S. N. Crory, C.-Y. Yao, J. R. Howard, G. Saucedo, A. Prasanna de Silva, E. V. Anslyn, *Angew. Chem. Int. Ed.* **2021**, *60*, 13819–13823.

[15] G. Fischer, *Chem. Soc. Rev.* **2000**, *29*, 119–127.

[16] Y. Zhang, P. Yao, Y. Cui, Q. Wu, D. Zhu, *Adv. Synth. Catal.* **2018**, *360*, 4191–4196.

[17] F.-L. Wu, B. P. Ross, R. P. McGeary, *Eur. J. Org. Chem.* **2010**, 1989–1998.

[18] C. J. Oswood, D. W. C. MacMillan, *J. Am. Chem. Soc.* **2021**, *144*, 93–98.

[19] I. T. Kay, J. E. D. Barton, D. J. Collins, B. Kowalczyk, G. Mitchell, J. M. Shribbs, J. M. Coz, N. J. Barnes, S. C. Smith. **2012**, US Patent US005856273A (Zeneca Limited).

[20] P. Chaignon, S. Cortial, V. Guerineau, M.-T. Adeline, C. Gianotti, G. Fan, J. Ouazzani, *Photochem. Photobiol.* **2005**, *81*, 1539–1543.

[21] F. Du, Q. Zhou, Y. Fu, Y. Chen, Y. Wu, G. Chen, *Synlett* **2019**, *30*, 2161–2168.

[22] X. Chen, X.-Y. Zhou, H. Wu, Y.-Z. Lei, J.-H. Li, *Commun* **2018**, *48*, 2475–2484.
